# Novel Hydroxamic Acids Containing Aryl-Substituted 1,2,4- or 1,3,4-Oxadiazole Backbones and an Investigation of Their Antibiotic Potentiation Activity

**DOI:** 10.3390/ijms25010096

**Published:** 2023-12-20

**Authors:** Anastasia A. Zhukovets, Vladimir V. Chernyshov, Aidar Z. Al’mukhametov, Tatiana A. Seregina, Svetlana V. Revtovich, Mariia A. Kasatkina, Yulia E. Isakova, Vitalia V. Kulikova, Elena A. Morozova, Anastasia I. Cherkasova, Timur A. Mannanov, Anastasia A. Anashkina, Pavel N. Solyev, Vladimir A. Mitkevich, Roman A. Ivanov

**Affiliations:** 1 Translational Medicine Research Center, Sirius University of Science and Technology, Olympic Ave. 1, 354340 Sochi, Russia; nastya.zhukovets0011@gmail.com (A.A.Z.); almuhametov.az@talantiuspeh.ru (A.Z.A.); km.kasatkina@gmail.com (M.A.K.); isakova.ye@talantiuspeh.ru (Y.E.I.); a-cherkasova2000@mail.ru (A.I.C.); tim.mann@yandex.ru (T.A.M.); ivanov.ra@talantiuspeh.ru (R.A.I.); 2 Engelhardt Institute of Molecular Biology of the Russian Academy of Sciences, 32 Vavilov St., 119991 Moscow, Russia; tatyana.s82@gmail.com (T.A.S.); svetla21@mail.ru (S.V.R.); vitviku@eimb.ru (V.V.K.); elmorozova@yahoo.com (E.A.M.); nastya@eimb.ru (A.A.A.); solyev@gmail.com (P.N.S.); mitkevich@gmail.com (V.A.M.)

**Keywords:** hydroxamic acids, 1,2,4-oxadiazoles, 1,3,4-oxadiazoles, antibacterial compounds, antibiotic potentiation, gram-negative bacteria, lipopolysaccharide, LpxC

## Abstract

UDP-3-*O-*(*R*-3-hydroxymyristoyl)-*N*-acetylglucosamine deacetylase (LpxC) is a zinc amidase that catalyzes the second step of the biosynthesis of lipid A, which is an outer membrane essential structural component of Gram-negative bacteria. Inhibitors of this enzyme can be attributed to two main categories, non-hydroxamate and hydroxamate inhibitors, with the latter being the most effective given the chelation of Zn^2+^ in the active site. Compounds containing diacetylene or acetylene tails and the sulfonic head, as well as oxazoline derivatives of hydroxamic acids, are among the LpxC inhibitors with the most profound antibacterial activity. The present article describes the synthesis of novel functional derivatives of hydroxamic acids—bioisosteric to oxazoline inhibitors—containing 1,2,4- and 1,3,4-oxadiazole cores and studies of their cytotoxicity, antibacterial activity, and antibiotic potentiation. Some of the hydroxamic acids we obtained (**9c**, **9d**, **23a**, **23c**, **30b**, **36**) showed significant potentiation in nalidixic acid, rifampicin, and kanamycin against the growth of laboratory-strain *Escherichia coli* MG1655. Two lead compounds (**9c**, **9d**) significantly reduced *Pseudomonas aeruginosa* ATCC 27853 growth in the presence of nalidixic acid and rifampicin.

## 1. Introduction

Human infections caused by pathogenic bacteria kill millions of people each year, many of which are caused by diarrheal diseases, bacteria-driven tuberculosis, and lower respiratory infections [1,2,3]. Severe sepsis caused by bacterial infections affects up to 18 million people worldwide each year, with mortality rates ranging from 28% to 50% [1,4]. Bacterial infections are a major health problem in the world, and the treatment of these infectious diseases is becoming increasingly difficult given the development of antibiotic resistance [5,6,7,8,9,10]. Back in 1945, Alexander Fleming, who discovered penicillin, warned that bacteria could become resistant to antibiotics in the future. The development of resistance to antibiotics is a natural evolutionary process for microorganisms. The development of antibiotic resistance is aggravated by the uncontrolled use of antibiotics in medicine and agriculture [6]. Resistant strains can multiply and spread where infection prevention and control measures are not followed. They are also found in the environment (water, soil, and air).

Policies recommended by the World Health Organization (WHO) include research on new antibiotics, but no new class of antibiotics has been discovered since daptomycin and linezolid in the 1980s, and only optimizations or combinations of already known compounds have been recently commercialized [7]. Widespread resistance to available antibiotics in clinically important pathogenic bacteria is currently a global challenge because of an ever-increasing number of strains that are resistant to multiple classes of antibiotics. This means that progress in modern medicine, which relies on the availability of effective antibacterial drugs, is at risk. Thus, the need for the development of new antibiotics, delivery systems, and bacterial diagnostics is urgent, as well as the use of “antibiotic adjuvants/potentiators” in combination with antibiotics [11].

The majority of pathogenic microorganisms that can cause various serious diseases are Gram-negative bacteria; in particular, multidrug-resistant Gram-negative bacteria (MDRGNs) have become a major threat to hospitalized patients, with mortality rates ranging from 30% to 70% [12]. For example, *E. coli* is the leading cause of urinary tract infections, *P. aeruginosa* causes hospital-acquired pneumonia and bloodstream infections, *N. gonorrhoeae* is the source of gonorrhea, and *K. pneumoniae* causes urinary tract infections and pneumonia [1,13]. In addition, it is alarming that multidrug resistance is also common among the deadliest and most human-pathogenic bacterial species, such as the plague pathogen, *Y. pestis*, which have been isolated to different parts of the world (for example, Madagascar and Mongolia) [14].

Lipopolysaccharides (LPSs) are bacterial surface glycolipids produced by Gram-negative bacteria that are present in their outer membranes. An LPS is composed of three domains: lipid A, the core oligosaccharide, and the O antigen. The main function of LPSs is structural, as they act as barriers against agents toxic to the bacteria, for instance, antibiotics [15]. Lipid A, which is the hydrophobic anchor of lipopolysaccharides (LPSs), is an attractive target for the development of both new antibiotics and potentiators of action of already known antibiotics since bacteria lacking lipid A are generally unviable, while mutants with reduced biosynthesis of lipid A have slow growth and high sensitivity to a wide range of antibiotics [1].

A Zn-dependent metalloamidase, UDP-3-*O*-(*R*-3-hydroxymyristoyl)-*N*-acetylglucosamine deacetylase (LpxC), is considered to be a promising target for the commitment step involved in catalyzing lipid A biosynthesis [16]. Deacetylase LpxC is one of the most studied enzymes involved in the biosynthesis of lipid A. Inhibitors of this enzyme can potentially be both antibiotics and potentiators of action of already known antibiotics given the key role of LpxC in the biosynthesis of lipid A and its lack of homology with mammalian proteins. Also, it is worth noting that, at the present time, inhibitors of enzyme LpxC in a variety of Gram-negative microorganisms have been described in the literature and have shown promise in the treatment of diseases caused by corresponding bacterial infections (*P. aeruginosa*, *E. coli*, *N. gonorrhoeae*, *K. pneumoniae*, *B. pseudomallei*, *B. pertussis*).

Most currently known LpxC deacetylase inhibitors fall into two main categories, namely, hydroxamate inhibitors and non-hydroxamate inhibitors. The mechanism of pharmacological action of hydroxamate inhibitors is the chelation of Zn^2+^ ions in the active site of the enzyme. Existing hydroxamate inhibitors can mainly be subdivided into those containing a butadiyne tail, those containing an acetylene tail, those containing a sulfone head, and other types of hydroxamate inhibitors [1]. The main difficulty encountered in the development of this class of LpxC deacetylase inhibitors is the toxicity of hydroxamic acids and their metabolic breakdown intermediates [17]. Studies devoted to the development of potential inhibitors of enzyme LpxC with a non-hydroxamate structure have also been published in the periodical literature [18,19,20,21]. As shown in some of them [22,23], the conversion of hydroxamic acid into carboxylic acid leads to a loss of enzyme LpxC inhibition and, as a consequence, antibacterial activity in the target compounds. When summarizing the data, the antibacterial activity of LpxC inhibitors with a non-hydroxamate structure is most often not as pronounced as compared with similar activity in hydroxamic acids [1].

**Figure 1:**
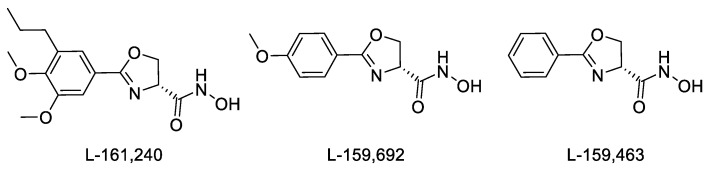
Structure of inhibitors of *E. coli* LpxC.

Another class of LpxC hydroxamate inhibitor, oxazoline inhibitors (L-161,240, L-159,692, and L-159,463 [24]), can be noted, which are some of the first compounds with high antibacterial activity [16,24,25] (Figure 1). It was found that L-161,240 and its derivatives are potent inhibitors of *E. coli* LpxC but have no effect on the growth and in vitro activity of *P. aeruginosa* [16]. It was shown in investigations of the antibacterial activity of this chemotype that replacing the oxygen atom with a sulfur atom in the oxazoline ring, as well as increasing the size of the heterocycle, leads to a loss of in vitro antibacterial activity against *P. aeruginosa* in the target compounds. However, the introduction of a trifluoromethoxy substituent into the meta- or para-position of the benzene ring, as well as the introduction of halogens into the para-position, results in micromolar concentrations of half-maximal *P. aeruginosa* growth inhibition (0.5–6.0 μM). In the para-fluorinated analogs of L-161,240, the addition of an alkyl or alkoxy group to the meta-position also has a beneficial effect on effectiveness [25].

The oxazoline LpxC inhibitors mentioned above (Figure 1) contain a hydroxamic acid functional group, which plays a key role in coordinating zinc ions in the active site of the enzyme, as well as a phenyl ring, which forms extensive interactions with hydrophobic substrate channels. The nitrogen atom of the oxazoline ring also forms interactions with the active site of the enzyme [26]. As shown previously [25], replacing the oxygen atom with another heteroatom in the oxazoline ring and increasing the size of the heterocycle lead to a loss of antibacterial activity in the inhibitors. However, in published studies devoted to oxazoline inhibitors, there are no examples of replacing the oxazoline heterocycle with another one of similar size containing nitrogen and oxygen atoms, the presence of which is considered to be mandatory. For example, oxazoline could be replaced with an oxadiazole ring, which has already established itself as a privileged heterocycle in medicinal chemistry with a wide range of pharmacological activity [27,28,29,30,31,32], including antibacterial activity [33,34,35]. We hypothesized that such a bioisosteric replacement of an oxazoline core with 1,2,4- and 1,3,4-oxadiazole would produce hydroxamic acids with a similar or improved antibacterial activity profile, particularly given the presence of an additional nitrogen atom as a hydrogen bond acceptor in the heterocyclic fragment, which may contribute to the better binding of target molecules at the enzyme site. On the other hand, replacing a non-aromatic heterocycle with an aromatic one leads to the formation of an additional planar fragment conjugated to the phenyl ring. This, in turn, greatly simplifies the synthesis and isolation of individual target hydroxamic acids, but it can also complicate the chelation of zinc ions in the active site of the enzyme because of the resulting conjugation of the heterocycle and the hydroxamate function. Therefore, we decided to synthesize hydroxamate derivatives linked to the heterocycle via an aliphatic linker in order to increase the mobility of the acid function and also obtain hydroxamic acids that are conjugated with the heterocycle directly or are linked to the heterocycle via an aromatic linker to establish further structure–activity relationships.

## 2. Results and Discussion

### 2.1. Synthesis of Hydroxamic Acids Containing a 1,2,4-Oxadiazole Nucleus

**Figure 2:**
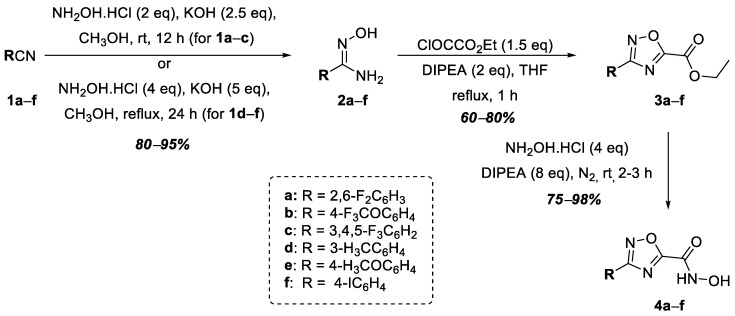
Synthesis of hydroxamic acids **4a**–**f**.

The synthesis of hydroxamic acids **4a**–**f** was carried out in three stages from commercially available benzonitriles (**1a**–**f**) (Figure 2). The following benzonitriles were selected: 4-trifluoromethoxybenzonitrile, **1b**; 3,4,5-trifluorobenzonitrile, **1c**; 3-methylbenzonitrile, **1d**; 4-iodobenzonitrile, **1f**, all of which, according to data in the literature, make it possible to obtain target products containing an aromatic substituent that has a positive effect on potential antibacterial activity, which has been shown in the case of oxazolines—that is, inhibitors of LpxC [25]. Additionally, 2,6-difluorobenzonitrile, **1a**, and 4-methoxybenzonitrile, **1e**, were chosen as the starting benzonitriles. The nucleophilic addition of hydroxylamine to the starting benzonitriles, **1a**–**f**, afforded amidoximes **2a**–**f**, which were then converted into corresponding 1,2,4-oxadiazoles, **3a**–**f**, via heterocyclization with ethyl oxalyl chloride in good yields. The subsequent interaction of esters **3a**–**f** with excess hydroxylamine in the presence of diisopropylethylamine (DIPEA) led to the formation of the target hydroxamic acids, **4a**–**f** (Figure 2).

**Figure 3:**
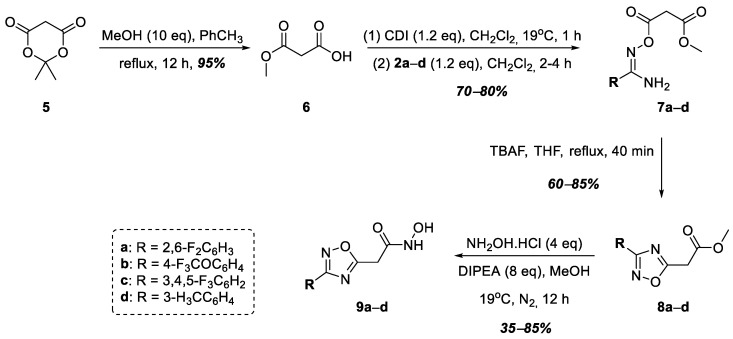
Synthesis of hydroxamic acids **9a**–**d**.

At the next stage, hydroxamic acids **9a**–**d** were synthesized, in which the acid functional group was connected to the 1,2,4-oxadiazole heterocycle at position 5 through a methylene linker (Figure 3). At the first stage, malonic acid monomethyl ester, **6**, was obtained from Meldrum’s acid, **5**, according to a known procedure [36]. Then, compound **6** was activated with *N*,*N′*-carbonyldiimidazole (CDI), after which, amidoximes **2a**–**e** were acylated to form *O*-acylamidoximes **7a**–**e** in good yields. In this case, fluorine-containing **2a**–**c** and 3-alkyl-substituted **2d** were chosen as the starting amidoximes. The heterocyclization of compounds **7a**–**d** into the target 1,2,4-oxadiazoles, **8a**–**d**, was carried out by refluxing in THF in the presence of tetrabutylammonium fluoride (TBAF) according to a known procedure [37]. The subsequent interaction of esters **8a**–**d** with excess hydroxylamine in the presence of DIPEA led to the formation of the target hydroxamic acids, **9a**–**d**, in good yields (Figure 3).

**Figure 4:**
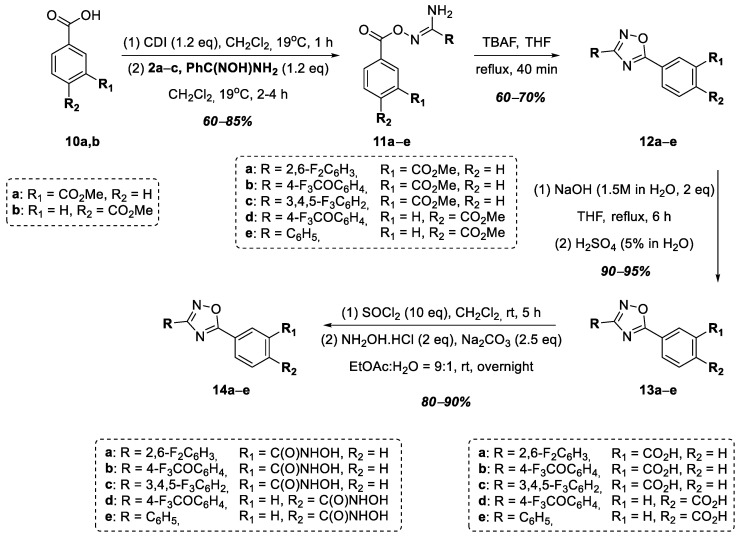
Synthesis of hydroxamic acids **14a**–**e**.

In order to study the effect of the substituent of the hydroxamic acid functional group on the antibacterial activity of the target compounds, we decided to synthesize a series of hydroxamic acids in which the hydroxamic acid functional group is directly conjugated to the benzene ring. Thus, at the next stage, a series of hydroxamic acids, **14a**–**e**, was obtained, in which the hydroxamic acid functional group connected to the 1,2,4-oxadiazole heterocycle at position 5 through a phenylene linker (Figure 4). Monomethyl esters of terephthalic and isophthalic acids, **10a**,**b**, were used as starting compounds. *O*-Acylamidoximes **11a**–**e** were obtained in good yields from acids **10a**,**b** similar to *O*-acylamidoximes **7a**–**d** using amidoximes **2a**–**c** and *N*-hydroxybenzimidamide. The cyclization of compounds **11a**–**e** in the presence of TBAF led to the formation of 1,2,4-oxadiazoles **12a**–**e**, which were further hydrolyzed to the corresponding 3- and 4-substituted benzoic acids—**13a**–**c** and **13d**–**e**. Using this synthetic scheme, we decided to obtain the target hydroxamic acids from carboxylic acids given the lack of reaction between the corresponding esters, **12a**–**e**, and a large excess of hydroxylamine. Thus, the target hydroxamic acids, **14a**–**e**, were obtained from benzoic acids **13a**–**e** in two stages: the formation of the corresponding acyl chlorides via the action of SOCl_2_ in CH_2_Cl_2_, followed by a reaction with hydroxylamine hydrochloride in the presence of Na_2_CO_3_, according to a modified known procedure [38] (Figure 4).

**Figure 5:**
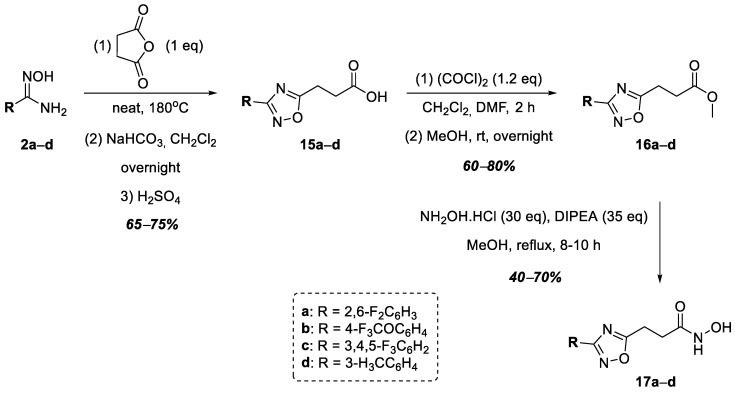
Synthesis of hydroxamic acids **17a**–**d**.

A series of hydroxamic acids containing a 1,2,4-oxadiazole ring linked at position 5 of the heterocycle through methylene and phenylene linkers was then completed with compounds **17a**–**d** containing an ethylene linker linking the heterocycle nucleus to the hydroxamic acid functional group (Figure 5). Thus, at the first stage of synthesis, aromatic amidoximes **2a**–**d** entered into a cyclocondensation reaction with succinic anhydride neatly, according to a known procedure [39]. The resulting carboxylic acids, **15a**–**d**, were converted into methyl esters **16a**–**d** in good yields. At the final stage of synthesis, esters **16a**–**d** were refluxed in methanol for 8–10 h with a large excess of hydroxylamine in the presence of DIPEA to obtain hydroxamic acids **17a**–**d** (Figure 5).

**Figure 6:**
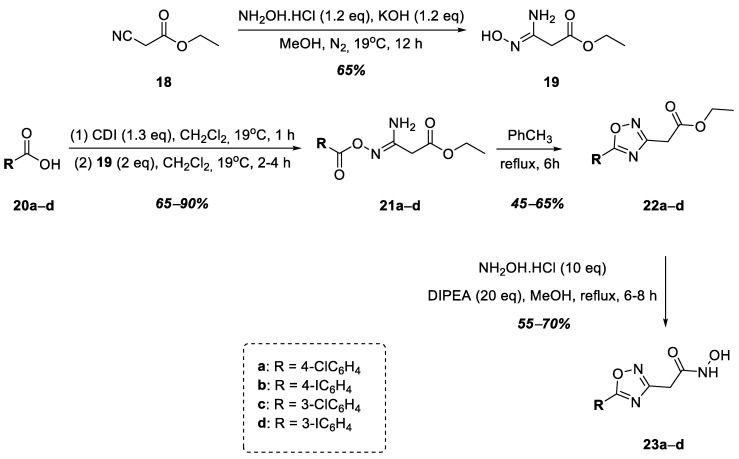
Synthesis of hydroxamic acids **23a**–**d**.

The next stage of this work was the synthesis of hydroxamic acids containing a 1,2,4-oxadiazole nucleus linked to the hydroxamic acid functional group by a methylene group at position 3 of the heterocycle (Figure 6). Thus, at the first stage, intermediate **19** was obtained via the nucleophilic addition of hydroxylamine to ethyl cyanoacetate, **18**, which was then used in the synthesis without purification. Halogen-substituted benzoic acids **20a**–**d** were activated by CDI followed by reactions with intermediate **19**, which led to the formation of a series of *O*-acylamidoximes, **21a**–**d**, in good yields. The target 1,2,4-oxadiazoles, **22a**–**e**, were obtained via the heterocyclization of *O*-acylamidoximes **21a**–**e** by refluxing their solution in toluene. Compounds **22a**–**d** were converted into hydroxamic acids **23a**–**d** in good yields under refluxing conditions using a solution of esters in methanol with an excess of hydroxylamine in the presence of DIPEA (Figure 6).

**Figure 7:**
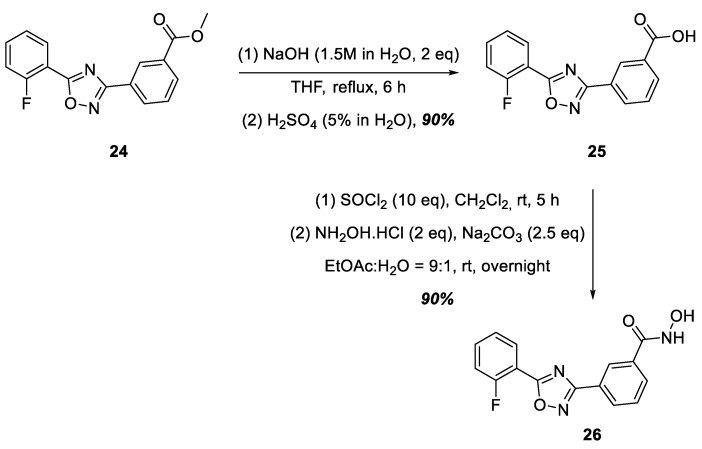
Synthesis of hydroxamic acid **26**.

A commercially available ester, **24**, was used as the starting compound for the synthesis of hydroxamic acid containing a 1,2,4-oxadiazole nucleus linked to the hydroxamic acid functional group by a phenylene group at position 3 of the heterocycle (Figure 7). Ester **24** was hydrolyzed to ataluren, **25**, which was further converted to hydroxamic acid **26** in good yield via the sequential action of SOCl_2_ in CH_2_Cl_2_ and hydroxylamine in the presence of Na_2_CO_3_ (Figure 7).

### 2.2. Synthesis of Hydroxamic Acids Containing a 1,3,4-Oxadiazole Nucleus

**Figure 8:**
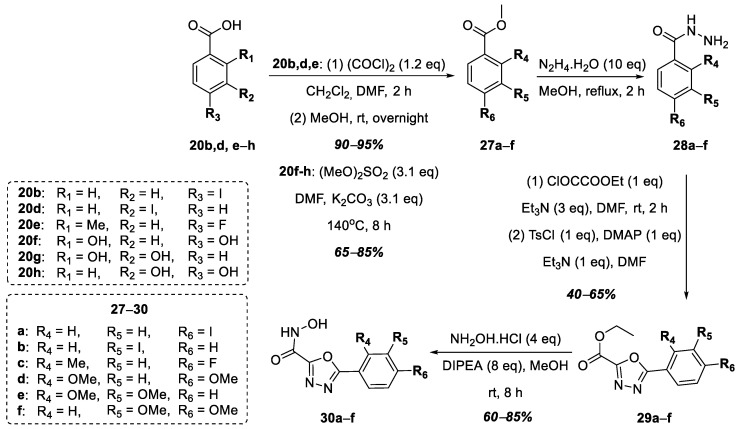
Synthesis of hydroxamic acids **30a**–**f**.

The next stage of this work was the replacement of the 1,2,4-oxadiazole nucleus with the 1,3,4-oxadiazole nucleus in the target hydroxamic acids containing the functional group of hydroxamic acid at position 2 of the heterocycle (Figure 8). In this case, the choice fell on hydroxy- and halogen-substituted benzoic acids based on the structures of the known LpxC inhibitors mentioned earlier. The synthesis began with commercially available benzoic acids, **20b**,**d**,**e**–**h**, which were converted into esters in the case of compounds **20b**,**d**,**e**, and in the case of compounds **20f**–**h**, the hydroxyl groups were at the same time methylated to form compounds **27a**–**f** in good yields. Further, a series of hydrazides, **28a**–**f**, without isolation or purification were obtained from esters **27a**–**f**, after which, compounds **28a**–**f** were converted into 1,3,4-oxadiazoles **29a**–**f**, according to a modified known procedure [40]. The target hydroxamic acids, **30a**–**f**, were obtained in good yields via the reaction of excess hydroxylamine hydrochloride with esters **29a**–**f** in the presence of DIPEA (Figure 8).

**Figure 9:**
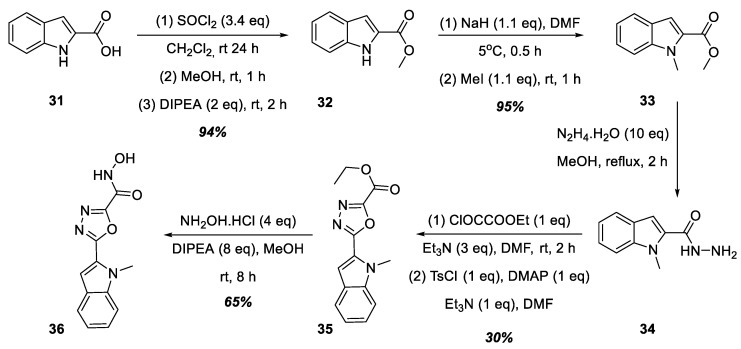
Synthesis of hydroxamic acid **36**.

To increase the lipophilicity of the aromatic substituent at position 5 of the 1,3,4-oxadiazole ring, we decided to synthesize hydroxamic acid **36**, structurally similar to acids **30a**–**f**, containing an indole nucleus at position 5 of the heterocycle (Figure 9). Thus, 1*H*-indole-2-carboxylic acid, **31**, was successively converted into its methyl ester, **32**, and then into methyl 1-methyl-1*H*-indole-2-carboxylate, **33**. Next, hydroxamic acid **36** was prepared similarly to the synthetic route shown in Figure 8: the formation of hydrazide **34** from methyl ester **33**, followed by heterocyclization into 1,3,4-oxadiazole **35** and a reaction with hydroxylamine hydrochloride in the presence of DIPEA.

### 2.3. In Vitro Studies of Potentiation of Antimicrobial Drugs via LpxC inhibitors

**Figure 10:**
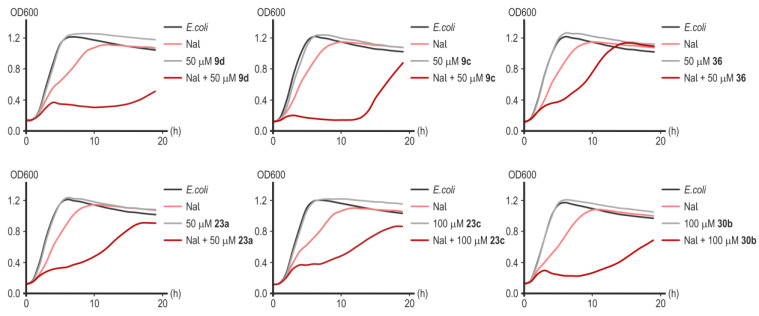
Representative growth curves of *E. coli* MG1655 in the presence of nalidixic acid (17.22 μM), without or with inhibitors of LpxC in concentrations of 50–100 µM. Data points are averages of optical density at a wavelength of 600 nm (OD600) with a <5% margin of error.

**Figure 11:**
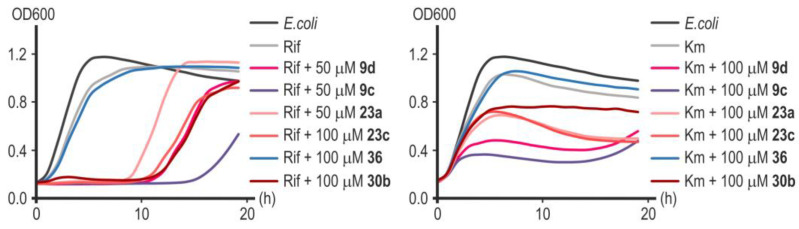
Representative growth curves of *E. coli* MG1655 in the presence of rifampicin (6.08 μM) and kanamycin (0.62 μM) with or without inhibitors of LpxC in concentrations of 50 or 100 µM. Data points are averages of optical density at a wavelength of 600 nm (OD600), with a <5% margin of error.

Preliminary screening of the synthesized substances was carried out on the laboratory strain of *E. coli* MG1655 since deacetylase LpxC has a high degree of homology in key amino acid residues of the active center of the enzyme among a wide range of Gram-negative pathogens [13]. The results of testing substances demonstrating inhibitory activity in the presence of nalidixic acid (NaI), rifampicin (Rif), and kanamycin (Km) are presented in Figure 10 and Figure 11. The greatest potentiating activity at low doses of nalidixic acid (17.22 μM) was demonstrated by substances **9d** and **9c** (Figure 10). Substances **23a**, **23c**, **36**, and **30b** inhibited bacterial growth less effectively (Figure 10).

Without antibiotics, all the target compounds synthesized were generally nontoxic to *E. coli* at all concentrations tested.

Substance **9c** showed the greatest effectiveness in potentiating the action of rifampicin and kanamycin (Figure 11). Remarkably, substances **23a** and **23c**, which turned out to be less effective in combination with nalidixic acid (Figure 10), significantly increased the sensitivity of *E. coli* cells to rifampicin and kanamycin (Figure 11). Compound **30b** significantly increased the sensitivity of cells to rifampicin but not to kanamycin (Figure 11). Rifampicin is not the antibiotic of choice for the treatment of infections caused by Gram-negative microflora, but increasing its effectiveness is potentially useful in preventing the development of opportunistic infections during anti-tuberculosis therapy [41]. Compound **36** was completely ineffective in combination with rifampicin or kanamycin (Figure 11). In the studied concentration range (50–100 µM), none of the substances d exhibited a toxic effect on cell growth in the absence of an antibiotic (Figure 10). However, despite the powerful bacteriostatic effect of compounds **9c** and **9d** in the first 10 h of incubation, we subsequently observed a resumption of culture growth, which can be explained by the metabolic transformation of the compound or by an occurrence of adaptive mutations.

Based on the results obtained for *E. coli*, two lead compounds were selected for their potentiation evaluation.

**Figure 12:**
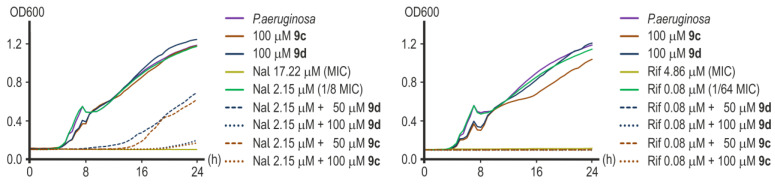
Representative growth curves of *P. aeruginosa* ATCC 27853 in the presence of nalidixic acid (**left**) and rifampicin (**right**) with or without **9c** or **9d**.

Substances **9c** and **9d** significantly reduced *P. aeruginosa* ATCC 27853 growth and lowered the MICs of the antibiotics tested when used together in combination with those products. The MIC value for nalidixic acid in the presence of 100 μM of **9c** or 100 μM of **9d** decreased approximately eight-fold from 17.22 μM to 2.15 μM (Figure 12). The combination of 50 μM of substance **9c** to 2.15 μM of nalidixic acid completely inhibited the growth of *P. aeruginosa* within 14 h, while the addition of 50 μM of substance **9d** to the same amount of nalidixic acid suppressed the growth of the culture only within 8 h (Figure 12). However, the addition of both substances at this concentration reduced the MIC of nalidixic acid by four times. Compound **9c** at a concentration of 25 μM reduced the MIC of nalidixic acid by only two times. Compound **9d** was ineffective at this concentration.

Great results were obtained when investigating the effectiveness of rifampicin potentiation. Compounds **9c** and **9d** were equally effective in reducing the MIC of rifampicin by 64 times from 4.86 μM to 0.08 μM at both concentrations of 100 μM and 50 μM (Figure 12). The addition of 25 μM of substance **9c** led to a twofold decrease in the MIC value of rifampicin, and 25 μM of compound **9d** was inactive.

Without antibiotics, both compounds were generally nontoxic to *P. aeruginosa* at all concentrations tested.

When comparing the microbiological results obtained in this work and those obtained for the oxazoline inhibitors shown in Figure 1, several points should be noted. Firstly, oxazoline inhibitors had standalone antibacterial activity against the *E. coli* cell line [24], while the compounds we synthesized did not have such activity. Secondly, in a previous work, the LpxC inhibition of oxazoline derivatives was carried out against the purified *P. aeruginosa* enzyme [25], while in the present work, the data on the inhibition of the growth of *P. aeruginosa* are presented. There are no data about the potentiating activity of oxazoline inhibitors [24,25] in the presence of antibiotics; at the same time, our work shows that oxadiazole derivatives exhibit a synergistic effect, significantly enhancing the effectiveness of antibacterial drugs. Thus, our results show a new chemotype that can be effectively utilized for potentiating the action of known antibiotics against Gram-negative bacteria resistant to these antibiotics.

In general, the results obtained during our studies of the antibiotic-potentiating activities of the target hydroxamic acids correlate with the structure–activity relationships observed for oxazoline inhibitors [25]. The presence of an *sp^3^*-hybrid carbon atom linked with the hydroxamic acid functional group appears to be extremely important, and it likely facilitates the chelation of zinc ions at the intended target. The phenylene linker between the hydroxamate function and the heterocycle core not only appears to hinder the chelation of zinc ions in the active site of LpxC but also significantly increases the size of the target molecules, which may affect their binding to the enzyme in general. The preferred positions for introducing substituents into the phenyl moiety conjugated with the heterocycle are meta- and/or para-positions. Preference is given to small substituents that do not extend outside the plane of the aromatic ring—electron-withdrawing groups (-F, -Cl) or electron-donating groups (-CH_3_).

### 2.4. Cytotoxicity Assay

The results of the cytotoxicity assay of the target hydroxamic acids against the HEK293 cell line are presented in Table 1. From the presented data regarding the cytotoxicity of the target hydroxamic acids, the following patterns can be noted. In general, hydroxamic acids directly conjugated to a 1,2,4- or 1,3,4-oxadiazole nucleus (compounds **4a**–**f**, **30a**–**f**) have high CC_50_ values, except compound **30a** (CC_50_ = 62.19 μM), which is noteworthy since its regioisomer, **4f**, has a CC_50_ value of 367 μM. The replacement of the phenyl substituent in the 1,3,4-oxadiazole ring with a 1-methylindole nucleus leads to the increased toxicity of hydroxamic acid **36**. The introduction of a methylene linker into hydroxamic acids containing a 1,2,4-oxadiazole ring at position 5 of the heterocycle leads to an increase in the toxicity of the target compounds by 2.5–3.5 times (compounds **4a**–**d** and **9a**–**d**, respectively). An increase in the number of methylene groups in the linker leads to a significant decrease in the CC_50_ values of hydroxamic acids **17a**–**d** (CC_50_ values ranged from 21.30 to 59.22 μM) compared with compounds **9a**–**d** (CC_50_ values ranged from 108.10 to 188.60 μM). At the same time, the introduction of a phenylene linker between the hydroxamic acid functional group and the 1,2,4-oxadiazole core leads to a significant increase in the toxicity of the target compounds (compounds **4a**–**c** and **14a**–**c**, respectively). It is worth noting that the position of the hydroxamic acid functional group in the phenyl substituent does not have a particular effect on the cytotoxicity of the target compounds (compounds **14b** (CC_50_ = 6.39 μM) and **14d** (CC_50_ = 5.28 μM)). Hydroxamic acids **23a**–**d** containing a methylene group between the functional group of hydroxamic acid and position 3 of the 1,2,4-oxadiazole nucleus have similar cytotoxicity indices (CC_50_ values ranging from 112.50 to 171.10 μM) as acids **9a**–**d**. Replacing the methylene linker with a phenylene one in this case leads to compound **26**, which, like other examples of *N*-hydroxybenzamides in this work, is highly toxic (CC_50_ = 21.74 μM).

### 2.5. Computer Modeling of Active Compounds

#### 2.5.1. Docking of Active Compounds to LpxC Structure

**Figure 13:**
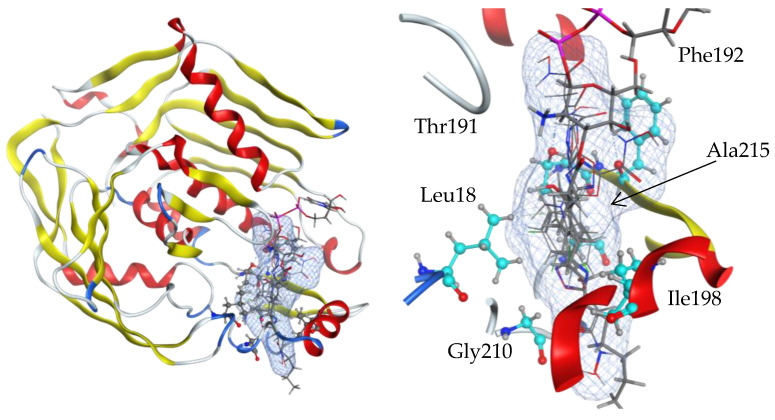
Side views of ligand-binding pockets in the structure of an LpxC protein (4mdt). Locations of **9c**, **9d, 23a, 23c, 30b**, and **36** ligand binding sites are the same as for the 4mdt ligand UDP-(3-*O*-(R-*3*-hydroxymyristoyl))-glucosamine (blue surface), and they include mostly hydrophobic residues: Leu18, Thr191, Phe192, Ile198, Gly210, and Ala215. Ligand interaction figures with the amino acid residues of the binding sites are given in Supplementary Materials Figures S1–S6. Full lists of amino acids involved in the interaction are given in Supplementary Materials Table S1.

All six active chemical compounds were docked by the MOE 2019 program to the product UDP-(3-*O*-(R-*3*-hydroxymyristoyl))-glucosamine binding site of the LpxC structure (4mdt) (Figure 13). The docking experiments showed that the locations of the **9c**, **9d, 23a, 23c, 30b**, and **36** ligand binding sites are the same as for the 4mdt ligand UDP-(3-*O*-(*R*-*3*-hydroxymyristoyl))-glucosamine (Figure 13, blue surface) and that they include mostly hydrophobic residue: Leu18, Thr191, Phe192, Ile198, Gly210, and Ala215.

#### 2.5.2. Pharmacophore Models of Active Compounds

**Figure 14:**
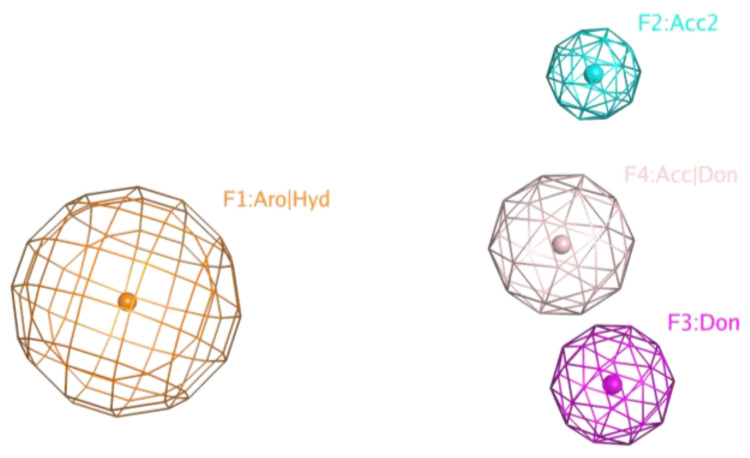
A simple pharmacophore model of four pharmacophore centers finds all compounds synthesized, both active and inactive.

Chemical compounds were pre-aligned with the flexible alignment function of the MOE 2019 program to compose the pharmacophore model. The pharmacophore centers were selected via the Consensus option according to the maximum density of atoms in the superposition. A simple model of four pharmacophore centers finds all compounds synthesized, both active and inactive (Figure 14). Since the structure of the active compounds found does not differ significantly from inactive compounds and the differences between the two active compounds are often greater than between the active and inactive compounds, a selective pharmacophore model could not be constructed.

#### 2.5.3. QSAR Models of Active Compounds

**Figure 15:**
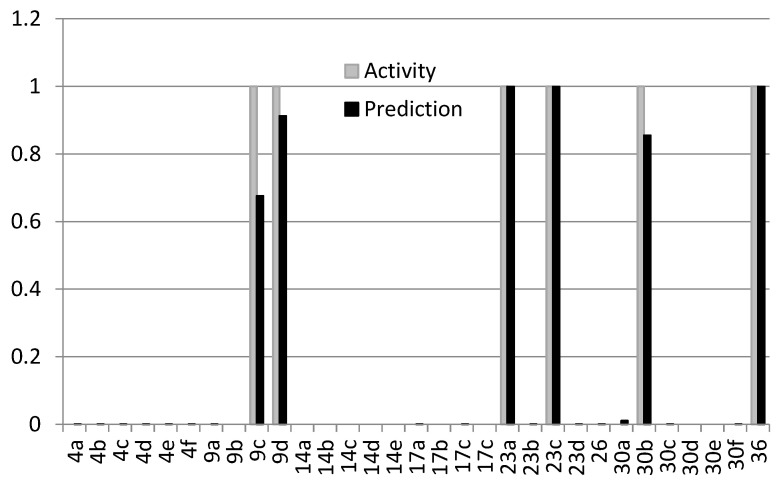
Activity (gray) and prediction (black) comparison of the best-performing QSAR model.

All compounds synthesized were marked in the MOE 2019 database as active (1) or inactive (0). In this case, the term activity was understood as the combined action together with the antibiotic. As mentioned above, the structure of the found active compounds differed from inactive compounds by one atom. We could not visually find a logical relationship between the active and inactive compounds, but this was possible using the QSAR method (Figure 15). Descriptors calculated from the structures of chemical compounds are often difficult to understand and logically analyze, but it is possible to build a highly efficient discriminative model from this application. It was found that the best-performing QSAR model is binary and includes 23 descriptors (Figure 15; Tables S2 and S3 in the Supplementary Materials).

The Binary Classification model includes, in particular, a number of aromatic atoms and a number of aromatic bonds; a number of double bonds; a number of rigid bonds; a number of rings; a number of chiral centers; a number of unconstrained chiral centers; relative negative partial charge; a polar surface area; and some less obvious descriptors. A full list of the descriptors used is in the Supplementary Materials (Table S2).

## 3. Materials and Methods

### 3.1. Materials

Mueller–Hinton agar (MHA) and Mueller–Hinton broth (MHB) were purchased from “HiMedia” (Mumbai, India); rifampicin (Rif) and nalidixic acid (Nal) were purchased from “Sigma-Aldrich” (St. Louis, MO, USA).

### 3.2. Cell Culture

The HEK293 cell line was obtained from the ECACC (European Collection of Authenticated Cell Cultures, cat. 85120602, Salisbury, UK). The cells were grown in monolayer culture in Dulbecco’s Modified Eagle Medium (DMEM) (PanEco, Moscow, Russia) supplemented with 2 mM of *L*-glutamine, 1% penicillin/streptomycin (Capricorn Scientific, Ebsdorfergrund, Germany), and 10% heat-inactivated fetal bovine serum (HyClone, Cytiva, Pasching, Austria) in a humidified atmosphere of 5% CO_2_ and at 37 °C (Binder, Tuttlingen, Germany). 

*Escherichia coli* strain MG1655 and *Pseudomonas aeruginosa* strain ATCC 27853 were obtained from the American Type Culture Collection (ATCC; Rockville, MD, USA). Subculturing and inoculum preparation were prepared according to the Clinical and Laboratory Standards Institute (CLSI) recommended method, M7-A9 [42], using MHA and MHB.

### 3.3. Generation of Growth Curves

Growth curves were obtained with a Bioscreen C automated growth analysis system. Cultures of *E. coli* MG1655 were grown overnight at 37 °C, diluted 1:100 in fresh medium, inoculated into honeycomb wells in triplicate, and grown at 37 °C with maximum shaking using the platform of the Bioscreen C instrument. When the cultures reached an optical density (OD600) of 0.2, cells were treated with nalidixic acid (17.22 μM), rifampicin (6.08 μM), or kanamycin (0.62 μM) in the presence or absence of LpxC inhibitors at a concentration of 0.01–0.1 mM and incubated at 37 °C for 19 h. OD600 values were recorded automatically at specified times, and the means of the triplicate cultures were plotted.

### 3.4. Antibiotic Activity Evaluation

Minimum inhibitory concentrations (MICs) for rifampicin and nalidixic acid were determined via the modified broth microdilution method in triplicate according to CLSI guidelines M7-A9 and M100-A30 [43]. The antibiotics were serially diluted twofold in 100 μL of MHB. The bacteria inoculum was 100 μL of a 1.0 × 10^6^ CFU/mL dilution in MHB. MICs for antibiotics in the presence of **9c** or **9d** were determined with the same method. Rifampicin or nalidixic acid ranged from 4·MIC to 1/128·MIC and included zero concentration for each antibiotic in combination with **9c** or **9d** (100 μM, 50 μM, and 25 μM) in an equal volume of 50 μL. **9c** or **9d** stock solutions were prepared in DMSO; the total DMSO concentration in a well did not exceed 1%.

MIC endpoints were read after 24 h of incubation with an iMark microplate absorbance reader (Bio-Rad, Hercules, CA, USA). Growth curves of *P. aeruginosa* ATCC 27853 in the presence of rifampicin/nalidixic acid and **9c**/**9d** were obtained using a multifunction microplate reader, SuperMax 3100 (ShanghaiFlash, Shanghai, China). Cells were grown in triplicate at 37 °C with aeration.

### 3.5. Cytotoxicity Assay

For analysis, the HEK293 cells were seeded into a 96-well plate (Corning Incorporated, Corning, NY, USA) in 100 μL of complete culture medium at a final concentration of 1 × 10^4^ cells/well and cultured for 24 h for proper attachment. Subsequently, the medium was removed, and cells were exposed to the test inhibitor with different concentrations for 72 h in 100 μL of fresh complete culture medium.

The sensitivity of the cells to test inhibitor molecules was detected using the vital dye Alamar Blue (Invitrogen, Carlsbad, CA, USA). The Alamar Blue assay was performed according to the procedure described by O’Brien et al. [44]. After incubation with test inhibitor molecules, 10 μL of Alamar Blue reagent was added to each well, cell culture plates were returned to a humidified incubator, and the fluorescence was read after 3 h. The plates were exposed to an excitation wavelength of 545 nm, and the emission at 600 nm was measured with the CLARIOstar Plus (BMG Labtech, Ortenberg, Germany, Software Version 5.70 R2). To determine corresponding 50% cellular cytotoxicity (CC_50_) values, a four-parameter sigmoidal fit curve model (GraphPad Prism 8 software) was used to measure the data.

## 4. Conclusions

In sum, a series of compounds were synthesized containing a hydroxamic acid functional group conjugated directly to 1,2,4-oxadiazole and 1,3,4-oxadiazole, as well as through a methylene, ethylene, or phenylene linker, at positions 3 and 5 of the heterocycle (in the case of 1,2,4-oxadiazole). It was shown that the target hydroxamic acids containing a phenylene linker and an ethylene linker demonstrated low CC50 values (<59.22 μM), while acids directly conjugated to the heterocycle had low cytotoxicity. Among the above-mentioned acids, only two had weak potentiating activity in the presence of nalidixic acid against laboratory-strain *E. coli* MG1655—compounds **30b** and **36**—containing a hydroxamic acid functional group directly conjugated with 1,3,4-oxadiazole. Additionally, compound **30b** significantly increased the sensitivity of *E. coli* cells to rifampicin but not to kanamycin, while compound **36** was completely ineffective in combination with these antibiotics. Among the compounds containing a hydroxamic acid functional group linked by a methylene linker to a 1,2,4-oxadiazole core, the greatest potentiating activity in the presence of nalidixic acid, rifampicin, and kanamycin against *E. coli* cells was demonstrated by substances **9c** and **9d**. At the same time, substances **23a** and **23c**, which turned out to be less effective in combination with nalidixic acid, significantly increased the sensitivity of *E. coli* cells to rifampicin and kanamycin. Lead compounds **9c** and **9d** showed the best results in nalidixic acid and rifampicin action potentiation against *P. aeruginosa* ATCC 27853 growth. All six active chemical compounds may have two partially overlapping binding sites; both are involved in interactions with the reaction product, UDP-(3-*O*-(*R*-3-hydroxymyristoyl))-glucosamine. In the foreseeable future, expanding the library of target hydroxamic acids and a more detailed study of their potentiating effect in combination with antibiotics against the growth of Gram-negative bacterial cells are expected.

## Figures and Tables

**Table 1:** Cytotoxicity of target hydroxamic acids synthesized ^1^.

Compound	CC_50_^2^ (HEK293), μM	Compound	CC_50_^2^ (HEK293), μM
**4a**	>500 ^3^	**17a**	30.83 ± 3.71
**4b**	380.20 ± 10.16	**17b**	42.40 ± 4.58
**4c**	>500 ^3^	**17c**	59.22 ± 7.27
**4d**	456.60 ± 18.15	**17d**	21.30 ± 2.53
**4e**	>250 ^3^	**23a ^4^**	112.50 ± 18.59
**4f**	367.00 ± 53.94	**23b**	168.80 ± 22.46
**9a**	187.60 ± 31.24	**23c ^4^**	142.80 ± 3.84
**9b**	108.10 ± 23.87	**23d**	171.10 ± 22.86
**9c^4^**	188.60 ± 29.54	**26**	21.74 ± 3.43
**9d^4^**	176.30 ± 17.78	**30a**	62.19 ± 9.88
**14a**	25.54 ± 3.11	**30b ^4^**	>200 ^3^
**14b**	6.39 ± 1.32	**30c**	>200 ^3^
**14c**	4.53 ± 1.22	**30d**	>600 ^3^
**14d**	5.28 ± 1.05	**30e**	770.50 ± 58.37
**14e**	15.00 ± 0.53	**30f**	478.30 ± 50.48
		**36 ^4^**	131.80 ± 23.70

^1^ The data represent mean ± SD (standard deviation) from at least 3 independent experiments, ^2^ CC_50_, cytotoxic concentration; the concentration resulting in 50% death of cells. ^3^ The compound indicated could not be tested at a concentration higher than indicated given its solubility. ^4^ Compounds that showed potentiating activity of nalidixic acid against laboratory-strain *E. coli* MG1655.

## Data Availability

Data are contained within the article and Supplementary Materials.

## Supplementary Materials

The following supporting information can be downloaded at https://www.mdpi.com/article/10.3390/ijms25010096/s1, Experimental procedures for synthesis and characterization data of all target hydroxamic acids, as well as intermediates of their synthesis, copies of their ^1^H- and ^13^C-NMR spectra, and HRMS; Tables S1–S3.
