# Supplementary material for: Novel Hydroxamic Acids Containing Aryl-Substituted 1,2,4- or 1,3,4-Oxadiazole Backbones and an Investigation of Their Antibiotic Potentiation Activity
Source: Int J Mol Sci. 2023 Dec 20;25(1):96. doi: 10.3390/ijms25010096 (PMC10779255; doi:10.3390/ijms25010096)
Supplement: Supplementary file 1 [file ijms-25-00096-s001.zip › ijms-2725539-supplementary.pdf]

Supplementary Material

Novel Hydroxamic Acids Containing Aryl-Substituted 1,2,4- or 1,3,4-Oxadiazole Backbones and an Investigation of Their Antibiotic Potentiation Activity

Anastasia A. Zhukovets [1], Vladimir V. Chernyshov [1,*], Aidar Z. Al'mukhametov [1], Tatiana A. Seregina [2], Svetlana V. Revtovich [2], Mariia A. Kasatkina [1], Yulia E. Isakova [1], Vitalia V. Kulikova [2], Elena A. Morozova [2], Anastasia I. Cherkasova [1], Timur A. Mannanov [1], Anastasia A. Anashkina [2], Pavel N. Solyev [2], Vladimir A. Mitkevich [2], and Roman A. Ivanov [1]

[1] Translational Medicine Research Center, Sirius University of Science and Technology, Olympic Ave. 1, 354340 Sochi, Russia

[2] Engelhardt Institute of Molecular Biology of the Russian Academy of Sciences, 32 Vavilov St., 119991 Moscow, Russia

* Correspondence: vladimir.chernyshov2012@yandex.ru

Experimental section

General S3

Procedures for synthesis and compounds characterization S4

Synthesis of 1,2,4-oxadiazole derivatives S4

Synthesis of 1,3,4-oxadiazole derivatives S20

References S25

^{1}H, ^{13}C NMR spectra for *O*-acylamidoximes synthesized S27

^{1}H, ^{13}C NMR spectra for ethyl 3-aryl 1,2,4-oxadiazole-5-carboxylates synthesized S49

^{1}H, ^{13}C NMR spectra for methyl 2-(3'-aryl-1',2',4'-oxadiazol-5'-yl)acetates and methyl 3-(3-aryl-1,2,4-oxadiazol-5-yl)benzoates synthesized S61

^{1}H, ^{13}C NMR spectra for ethyl 2-(5'-(aryl)-1',2',4'-oxadiazol-3'-yl)acetates synthesized S77

^{1}H, ^{13}C NMR spectra for 3-(3-aryl-1,2,4-oxadiazol-5-yl)benzoic acids synthesized S85

^{1}H, ^{13}C NMR spectra for 3-(3-aryl-1,2,4-oxadiazol-5-yl)propanoic acids synthesized S91

^{1}H, ^{13}C NMR spectra for methyl 3-(3-aryl-1,2,4-oxadiazol-5-yl)propanoates synthesized S99

^{1}H, ^{13}C NMR spectra for 3-aryl-*N*-hydroxy-1,2,4-oxadiazole-5-carboxamides synthesized S105

^{1}H, ^{13}C NMR spectra for 2-(3-aryl-1,2,4-oxadiazol-5-yl)-*N*-hydroxyacetamides synthesized S117

^{1}H, ^{13}C NMR spectrum for 3-(5-(2-fluorophenyl)-1,2,4-oxadiazol-3-yl)-*N*-hydroxybenzamide S125

^{1}H, ^{13}C NMR spectra for 3-(3-aryl-1,2,4-oxadiazol-5-yl)-*N*-hydroxybenzamides synthesized S127

^{1}H, ^{13}C NMR spectra for 2-(5-aryl-1,2,4-oxadiazol-3-yl)-*N*-hydroxyacetamides synthesized S137

^{1}H, ^{13}C NMR spectra for *N*-hydroxy-3-aryl-1,2,4-oxadiazol-5-yl)propanamides synthesized S145

^{1}H, ^{13}C NMR spectra for ethyl 5-aryl-1,3,4-oxadiazole-2-carboxylates synthesized S153

^{1}H, ^{13}C NMR spectra for *N*-Hydroxy-5-(3-iodophenyl)-1,3,4-oxadiazole-2-carboxamides synthesized S163

HRMS spectra for hydroxamic acids obtained S177

Ligand interactions (for 6 lead compounds) with amino acid residues in the predicted binding site S187

Quantitative structure–activity relationship S191

General

All solvents and reagents were obtained from commercial sources and used without further purification unless otherwise stated. ^1H and ^{13}C NMR spectra were recorded on a 300 MHz (300.1, 75.5 MHz, respectively) and 400 MHz (400.1, 100.6 MHz, respectively) spectrometers (Bruker BioSpin GmbH, Bruker Avance Neo, Germany) in $CDCl_3$, DMSO-d_6, and methanol-d_4 solutions using 0.05% Me_4Si as the external or internal standard. Chemical shifts δ are reported in parts per million (ppm); multiplicity: *s*, singlet; *d*, doublet; *t*, triplet; *dd*, double of doublets; *tt*, triplet of triplets; *dt*, double of triplet; t*d*, triplet of doublets; *ddd*, doublet of doublets of doublets; *m*, multiplet; *br*, broad; the coupling constants *J* are reported in units of Herts [Hz].The structure of the products was determined by analyzing ^1H and ^{13}C NMR spectra; assignments on a routine basis by a combination of 1D and 2D experiments (HSQC, HMBC). UPLS-MS analyses were performed on a «Vanquish Flex» chromatograph (Thermo Scientific, USA) with Diode Array Detector FG (DAD FG, Thermo Scientific, USA) combined with an ISQ EM Single Quadrupole Mass Spectrometer (Thermo Scientific, USA). A 6-minute gradient separation on an Agilent Poroshell 120 EC-C18 (100 mm·2.0 mm, particle size 1.9 μm) column was run under the following conditions: solvent A = water with 0.1% formic acid, solvent B = acetonitrile with 0.1% formic acid, from 0 to 2.5 min – gradient elution from A:B = 9:1 to A:B = 1:9, from 2.5 min to 3.5 min – elution in A:B = 1:9, from 3.5 min to 6 min – equilibration of the chromatographic column in A:B = 19:1 at a flow rate 0.5 ml/min. Column temperature was 40°C, injection volume of the sample was 1 μl. An electrospray ionization source was used to ionize the samples. Ions of positive and negative polarities were detected in the full ion current recording mode; the range of recorded masses was 10-700 *m/z*. The absorption spectra were recorded on a diode array detector at 2 wavelengths: 220 nm and 254 nm. High-resolution MS (HRMS) analyses were performed using a Bruker maXis II 4G ETD mass spectrometer and an UltiMate 3000 chromatograph equipped with Acclaim RSLC 120 C18 2.2 μm 2.1·100 mm column. Spectrum registration mode was electrospray ionization (ESI), with a full scan between *m/z* 100 and 1500, tandem MS (MS/MS) with selection of three most intense ions, collision-induced dissociation (CID) at 10–40 eV, and nitrogen as a collision gas. Melting points were determined on Melting Point Apparatus SMP50. The target substances were lyophilized using a LABCONCO FreeZone 2.5 l freeze dryer (samples were preliminarily frozen in a freezer at -80°C for 1 h, sublimation was carried out for 12 h, residual pressure 0.003 mbar). Thin-layer chromatography (TLC) was carried out on Merck silica gel 60 F254 precoated plates; compounds on TLC were visualized by illumination under UV light (254 nm) or by ninhydrin or phosphomolybdic acid staining. Column chromatography was performed on silica gel (60–200 mesh, Sisco).

Procedures for synthesis and compounds characterization

Synthesis of 1,2,4-oxadiazole derivatives

Method A. General procedure of the synthesis of amidoximes **2a-c.** A weighed portion of $NH_2OH·HCl$ (2 eq) was dissolved in 5 ml of CH_3OH in a round bottom flask. A solution of KOH (2.5 eq) in 5 ml of CH_3OH was added to the resulting solution. The reaction mixture was stirred at r.t. under N_2 atmosphere for 10 min. Then a solution of corresponding benzonitrile **1a-c** (1 eq) in 5 ml of CH_3OH was added to the reaction mixture in one portion. The reaction mixture was stirred at r.t. under N_2 atmosphere overnight. After completion of the reaction the precipitate was filtered off, the solution was evaporated to dryness using rotary evaporation, residue was dissolved in 40 ml of EtOAc. The solution obtained was washed successively with deionized water (2·15 ml) and saturated NaCl solution (1·15 ml). The organic layer was dried with anhydrous Na_2SO_4 under vigorous stirring, after which the precipitate was filtered off; the solution was evaporated to dryness to give amidoximes **2a-c** in good yields.

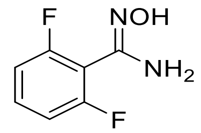

2,6-Difluoro-N'-hydroxybenzimidamide (**2a**). Light yellow powder, 460 mg (90%). **UPLC-MS (ESI+)**: found *m/z* 173.1 $[M + H]^+$; calculated for $C_7H_7F_2N_2O^+$ 173.0. Physicochemical properties of compound **2a** are in a good agreement with published data [1].

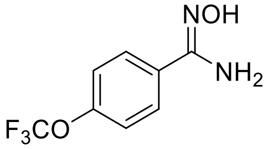

N'-Hydroxy-4-(trifluoromethoxy)benzimidamide (**2b**). White powder, 690 mg (95%). **UPLC-MS (ESI+)**: found *m/z* 221.1 $[M + H]^+$; calculated for $C_8H_8F_3N_2O_2^+$ 221.1. Physicochemical properties of compound **2b** are in a good agreement with published data [2].

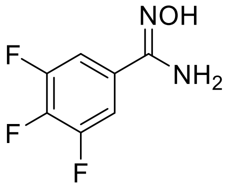

3,4,5-Trifluoro-N'-hydroxybenzimidamide (**2c**). White powder, 320 mg (90%). **UPLC-MS (ESI+)**: found *m/z* 191.1 $[M + H]^+$; calculated for $C_7H_6F_3N_2O^+$ 191.0. Physicochemical properties of compound **2c** are in a good agreement with published data [3].

Method B. General procedure of the synthesis of amidoximes **2d-f**. A weighed portion of $NH_2OH·HCl$ (4 eq) was dissolved in 10 ml of CH_3OH in a round bottom flask. A solution of KOH (5 eq) in 10 ml of CH_3OH was added to the resulting solution. The reaction mixture was stirred at r.t. under N_2 atmosphere for 10 min. Then a solution of corresponding benzonitrile **1d-f** (1 eq) in 5 ml of CH_3OH was added to the reaction mixture in one portion. The reaction mixture was refluxed for 8 h, the progress was monitored by TLC (eluent CH_2Cl_2:*iso*-C_3H_7OH = 19:1). After completion of the reaction the precipitate was filtered off, the solution was evaporated to dryness using rotary evaporation, residue was dissolved in 40 ml of EtOAc. The solution obtained was washed successively with deionized water (2·15 ml) and saturated NaCl solution (1·15 ml). The organic layer was dried with anhydrous Na_2SO_4 under vigorous stirring, after which the precipitate was filtered off; the solution was evaporated to dryness to give amidoximes **2d-f** in good yields.

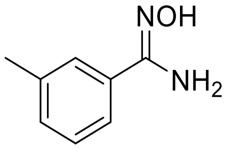

N'-Hydroxy-3-methylbenzimidamide (**2d**). Yellow amorphous substance, 143 mg (95%). **UPLC-MS (ESI+)**: found *m/z* 151.1 $[M + H]^+$; calculated for $C_8H_{11}N_2O^+$ 151.1. Physicochemical properties of compound **2d** are in a good agreement with published data [4].

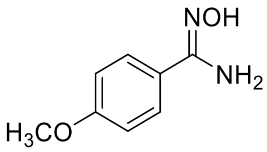

N'-Hydroxy-4-methoxybenzimidamide (**2e**). White powder, 620 mg (90%). **UPLC-MS (ESI+)**: found *m/z* 167.1 $[M + H]^+$; calculated for $C_8H_{11}N_2O_2^+$ 167.1. Physicochemical properties of compound **2e** are in a good agreement with published data [5].

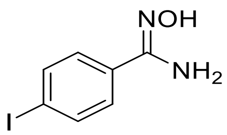

N'-Hydroxy-4-iodobenzimidamide (**2f**). White powder, 260 mg (80%). **UPLC-MS (ESI+)**: found *m/z* 263.0 $[M + H]^+$; calculated for $C_7H_8IN_2O^+$ 263.0. Physicochemical properties of compound **2e** are in a good agreement with published data [6].

Synthesis of ethyl 3-amino-3-(hydroxyimino)propanoate (**19**). A weighed portion of $NH_2OH{\cdot}HCl$ (2 g, 28.8 mmol, 1.2 eq) was dissolved in 10 ml of CH_3OH in a round bottom flask. A solution of KOH (1.6 g, 28.8 mmol, 1.2 eq) in 10 ml of CH_3OH was added to the resulting solution. The reaction mixture was stirred at r.t. under N_2 atmosphere for 10 min. After completion of the reaction the precipitate was filtered off, and a solution of ethyl 2-cyanoacetate **18** (2.55 ml, 24 mmol, 1 eq) in 5 ml of CH_3OH was added to the filtrate. The reaction mixture was stirred at r.t. under N_2 atmosphere overnight. After completion of the reaction the solution was evaporated to dryness to give ethyl 3-amino-3-(hydroxyimino)propanoate **19** as brown oil, which used in the next step without purification.

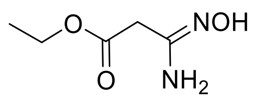

Ethyl 3-amino-3-(hydroxyimino)propanoate (**19**). Brown oil, 2.28 g (65%). **UPLC-MS (ESI+)**: found *m/z* 147.1 $[M + H]^+$; calculated for $C_5H_{11}N_2O_3^+$ 147.1. Physicochemical properties of compound **19** are in a good agreement with published data [7].

Synthesis of 3-methoxy-3-oxopropanoic acid (**6**). Obtained by a known procedure [8]. Meldrum's acid **5** (1.44 g, 10 mmol, 1 eq) was dissolved in 30 ml of toluene in a round bottom flask. Then CH_3OH (4 ml, 100 mmol, 10 eq) was added, and the resulting mixture was refluxed for 12 h, the progress was monitored by TLC (eluent CH_2Cl_2:*iso*-C_3H_7OH:HCOOH = 9:1:0.01) using Meldrum's acid (*Rf* = 0.9) and malonic acid (*Rf* = 0.1) as TLC marks. After the completion of the reaction, the solution was evaporated to dryness to give a 3-methoxy-3-oxopropanoic acid **6** (*Rf* = 0.7) as colorless liquid, 1.12 g (95%). The resulting ester **6** was further used in the next step without purification and characterization by physicochemical methods of analysis.

Method C. General procedure of the synthesis of O-acylamidoximes **7a-d**, **11a-e**, **21a-d**. The mixture of corresponding carboxylic acid **6**, **10a-d**, **20a-d** (1.0 eq) and *N,N'*-carbonyldiimidazole (CDI) (1.2 eq) were dissolved in 10 ml CH_2Cl_2, and the solution was stirred at r.t. under air atmosphere for 1.5 h. Then, the solution of the corresponding amidoxime **2a-d**, **19** (1.2 eq) in 10 ml CH_2Cl_2 was added; the resulting solution was stirred at r.t. under air atmosphere for 4-5 h, the progress was monitored by TLC (eluent CH_2Cl_2:*iso*-C_3H_7OH = 19:1). After completion of the reaction solution was evaporated to dryness and the residue was purified by column chromatography on silica gel (for eluents, see below). The combined organic fractions containing the target acylamidoximes **7a-d**, **11a-e**, **21a-d** were collected, the solution was evaporated to dryness to give corresponding *O*-acylamidoximes **7a-d**, **11a-e**, **21a-d** in good yields.

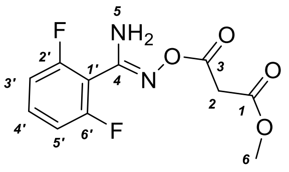

Methyl 3-(((amino(2',6'-difluorophenyl)methylene)amino)oxy)-3-oxopropanoate (**7a**). Column chromatography eluent – CH_2Cl_2:*iso*-C_3H_7OH (gradient from 100:0 to 95:5). White powder, 245 mg (70%). **^{1}H NMR (300 MHz, $CDCl_3$, δ)**: 7.47-7.35 (1H, *m*, 4'-CH), 7.02-6.93 (2H, *m*, 3'-CH, 5'-CH), 5.43 (2H, *br.s.*, 5-NH_2), 3.78 (3H, *s*, 6-CH_3), 3.60 (2H, *s*, 2-CH_2). **^{13}C NMR (75 MHz, $CDCl_3$, δ)**: 167.13 (C-1), 164.10 (C-3), 160.78 (C-2', C-6', *dd*, J^F = 254.1, 6.1 Hz), 149.37 (C-4), 132.53-131.96 (C-4', *m*), 112.28-111.75 (C-3', C-5', *m*), 109.10 (C-1'), 52.66 (C-6), 40.38 (C-2). **UPLC-MS (ESI+)**: found *m/z* 273.1 $[M + H]^+$; calculated $C_{11}H_{11}F_2N_2O_4^+$ 273.1.

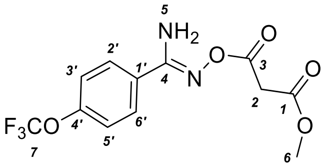

Methyl 3-(((amino(4'-(trifluoromethoxy)phenyl)methylene)amino)oxy)-3-oxopropanoate (**7b**). Column chromatography eluent – CH_2Cl_2:*iso*-C_3H_7OH (gradient from 100:0 to 95:5). Yellow powder, 250 mg (80%). **^{1}H NMR (400 MHz, $CDCl_3$, δ)**: 7.68-7.64 (2H, *m*, 2'-CH, 6'-CH), 7.20-7.17 (2H, *m*, 3'-CH, 5'-CH), 5.30 (2H, *br.s.*, 5-NH_2), 3.70 (3H, *s*, 6-CH_3), 3.53 (2H, *s*, 2-CH_2). **^{13}C NMR (101 MHz, $CDCl_3$, δ)**: 167.35 (C-1), 164.45 (C-3), 156.48 (C-4), 151.26 (C-4'), 129.29 (C-1'), 128.53 (C-2', C-6'), 120.97 (C-3', C-5'), 119.02 (C-7), 52.71 (C-6), 40.50 (C-2). **UPLC-MS (ESI+)**: found *m/z* 321.0 $[M + H]^+$; calculated $C_{12}H_{12}F_3N_2O_5^+$ 321.1.

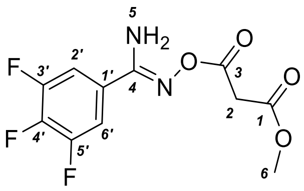

Methyl 3-(((amino(3',4',5'-trifluorophenyl)methylene)amino)oxy)-3-oxopropanoate (**7c**). Column chromatography eluent – CH_2Cl_2:*iso*-C_3H_7OH (gradient from 100:0 to 95:5). Light yellow powder, 320 mg (80%). **^{1}H NMR (300 MHz, $CDCl_3$, δ)**: 7.45-7.34 (2H, *m*, 2'-CH, 6'-CH), 5.44 (2H, *br.s*, 5-NH_2), 3.80 (3H, *s*, 6-CH_3), 3.61 (2H, *s*, 2-CH_2). **^{13}C NMR (75 MHz, $CDCl_3$, δ)**: 167.24 (C-1), 164.17 (C-3), 155.08 (C-4), 151.25 (C-3', C-5', *ddd*, J^F = 251.6, 10.1, 3.8 Hz), 141.59 (C-4', *dt*, J^F = 257.1, 15.2 Hz), 111.69-111.38 (C-2', C-6', *m*), 110.39-110.08 (C-1', *m*), 52.75 (C-6), 40.40 (C-2). **UPLC-MS (ESI+)**: found *m/z* 291.0 $[M + H]^+$; calculated $C_{11}H_{10}F_3N_2O_4^+$ 291.1.

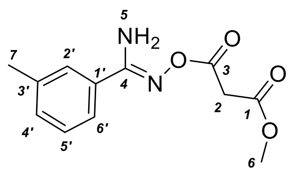

Methyl 3-(((amino(3'-methylphenyl)methylene)amino)oxy)-3-oxopropanoate (**7d**). Column chromatography eluent – CH_2Cl_2:*iso*-C_3H_7OH (gradient from 100:0 to 95:5). White powder, 190 mg (70%). **^{1}H NMR (400 MHz, $CDCl_3$, *δ*)**: 7.53 (1H, *br.s*, 2'-CH), 7.48-7.44 (1H, *m*, 5'-CH), 7.32-7.30 (2H, *m*, 4'-CH, 6'-CH), 5.31 (2H, *br.s.*, 5-NH_2), 3.79 (3H, *s*, 6-CH_3), 3.62 (2H, *s*, 2-CH_2), 2.39 (3H, *s*, 7-CH_3). **^{13}C NMR (101 MHz, $CDCl_3$, *δ*)**: 167.38 (C-1), 164.62 (C-3), 157.70 (C-4), 138.65 (C-3'), 131.97 (C-4'), 130.61 (C-1'), 128.65 (C-6'), 127.40 (C-2'), 123.66 (C-5'), 52.66 (C-6), 40.59 (C-2), 21.30 (C-7). **UPLC-MS** (**ESI+**): found *m/z* 251.1 $[M + H]^+$; calculated $C_{12}H_{15}N_2O_4^+$ 251.1.

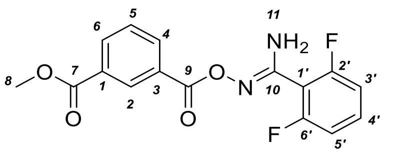

Methyl 3-((((amino(2',6'-difluorophenyl)methylene)amino)oxy) carbonyl)benzoate (**11a**). Column chromatography eluent – CH_2Cl_2:*iso*-C_3H_7OH (gradient from 100:0 to 98:2). Light yellow powder, 200 mg (60%). **^{1}H NMR (300 MHz, DMSO-*d*^{6}, *δ*)**: 8.62-8.61 (1H, *m*, 2-CH), 8.55-8.51 (1H, *m*, 4-CH), 8.25-8.21 (1H, *m*, 6-CH), 7.75-7.69 (1H, *m*, 5-CH), 7.65-7.58 (1H, *m*, 4'-CH), 7.35 (2H, *br.s*, 11-NH_2), 7.29-7.21 (2H, *m*, 3'-CH, 5'-CH), 3.92 (3H, *s*, 8-CH_3). **^{13}C NMR (75 MHz, DMSO-*d*^{6}, *δ*)**: 166.03 (C-7), 163.01 (C-9), 160.60 (C-2', C-6', *dd*, J^F = 250.0, 6.7 Hz), 150.02 (C-10), 134.54 (C-4), 133.88 (C-6), 132.96 (C-4', *t*, J^F = 10.2 Hz), 130.63 (C-1), 130.47 (C-2), 130.31 (C-3), 129.76 (C-5), 112.51-112.11 (C-3', C-5', *m*), 110.75 (C-1', *t*, J^F = 20.7 Hz), 52.90 (C-8). **UPLC-MS** (**ESI+**): found *m/z* 335.1 $[M + H]^+$; calculated $C_{16}H_{13}F_2N_2O_4^+$ 335.1.

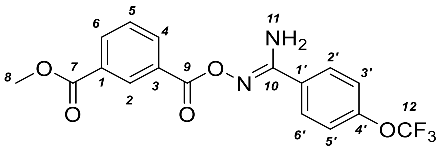

Methyl 3-((((amino(4'-(trifluoromethoxy)phenyl)methylene)amino) oxy)carbonyl)benzoate (**11b**). Column chromatography eluent – CH_2Cl_2:*iso*-C_3H_7OH (gradient from 100:0 to 98:2). White powder, 420 mg (85%). **^{1}H NMR (400 MHz, $CDCl_3$, *δ*)**: 8.64-8.63 (1H, *m*, 2-CH), 8.22-8.17 (2H, *m*, 4-CH, 6-CH), 7.75-7.72 (2H, *m*, 2'-CH, 6'-CH), 7.50 (1H, *t*, *J* = 7.8 Hz, 5-CH), 7.21-7.19 (2H, *m*, 3'-CH, 5'-CH), 5.25 (2H, *br.s.*, 11-NH_2), 3.89 (3H, *s*, 8-CH_3). **^{13}C NMR (101 MHz, $CDCl_3$, *δ*)**: 166.17 (C-7), 163.13 (C-9), 156.27 (C-10), 151.25 (C-4'), 133.96 (C-6), 133.85 (C-4), 130.71 (C-1), 130.39 (C-2), 129.91 (C-3), 129.60 (C-1'), 128.87 (C-5), 128.67 (C-2', C-6'), 120.99 (C-3', C-5'), 119.04 (C-12), 52.45 (C-8). **UPLC-MS** (**ESI+**): found *m/z* 383.1 $[M + H]^+$; calculated $C_{17}H_{14}F_3N_2O_5^+$ 383.1.

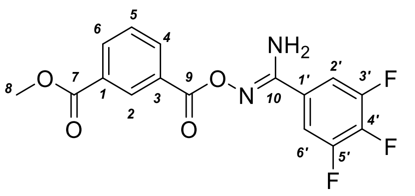

Methyl 3-((((amino(3',4',5'-trifluorophenyl)methylene)amino)oxy) carbonyl)benzoate (**11c**). Column chromatography eluent – CH_2Cl_2:*iso*-C_3H_7OH (gradient from 100:0 to 98:2). White powder, 175 mg (85%). **^{1}H NMR (300 MHz, $CDCl_3$, *δ*)**: 8.87-8.86 (1H, *m*, 2-CH), 8.39 (1H, *ddd*, *J* = 7.8, 1.8, 1.2 Hz, 4-CH), 8.32 (1H, *ddd*, *J* = 7.9, 1.7, 1.2 Hz, 6-CH), 7.92-7.81 (2H, *m*, 2'-CH, 6'-CH), 7.69 (1H, *td*, *J* = 7.8, 0.6 Hz, 5-CH), 4.02 (3H, *s*, 8-CH_3). **^{13}C NMR (75 MHz, $CDCl_3$, *δ*)**: 175.49 (C-9), 166.92 (C-10), 165.73 (C-7), 151.55 (C-3', C-5', *ddd*, J^F = 251.4, 10.3, 3.9 Hz), 141.80 (C-4', *dt*, J^F = 257.1, 15.2 Hz), 133.90 (C-6), 132.10 (C-4), 131.49 (C-1), 129.48 (C-5), 129.30 (C-2), 124.21 (C-3), 122.94-122.61 (C-1', *m*), 112.24-111.93 (C-2', C-6', *m*), 52.57 (C-8). **UPLC-MS** (**ESI+**): found *m/z* 353.1 $[M + H]^+$; calculated $C_{16}H_{12}F_3N_2O_4^+$ 353.1.

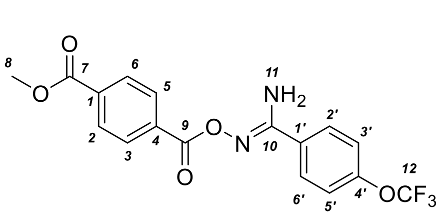

Methyl 4-((((amino(4-(trifluoromethoxy)phenyl)methylene)amino) oxy)carbonyl)benzoate (**11d**). Column chromatography eluent – CH_2Cl_2:*iso*-C_3H_7OH (gradient from 100:0 to 98:2). White powder, 100 mg (85%). **UPLC-MS** (**ESI+**): found *m/z* 383.1 $[M + H]^+$; calculated $C_{17}H_{14}F_3N_2O_5^+$ 383.1. *O*-Acylamidoxime **11d** was cyclized to 1,2,4-oxadiazole derivative without isolation and characterization.

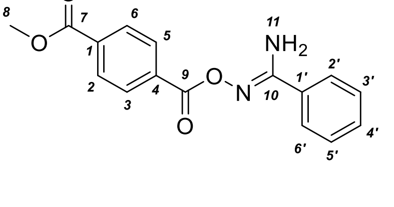

Methyl 4-((((amino(phenyl)methylene)amino)oxy)carbonyl)benzoate (**11e**). Column chromatography eluent – CH_2Cl_2:*iso*-C_3H_7OH (gradient from 100:0 to 9:1). White powder, 95 mg (80%). **UPLC-MS** (**ESI+**): found *m/z* 299.1 $[M + H]^+$; calculated $C_{16}H_{15}N_2O_4^+$ 299.1. *O*-Acylamidoxime **11e** was cyclized to 1,2,4-oxadiazole derivative without isolation and characterization.

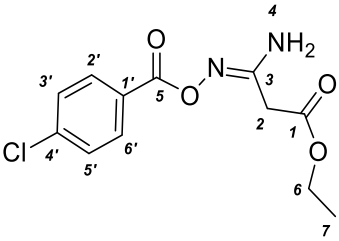

Ethyl 3-amino-3-(((4'-chlorobenzoyl)oxy)imino)propanoate (**21a**). Column chromatography eluent – CH_2Cl_2:*iso*-C_3H_7OH (gradient from 100:0 to 95:5). Light yellow powder, 720 mg (80%). **^{1}H NMR** (**400 MHz, $CDCl_3$, *δ***): 8.00-7.97 (2H, *m*, 2'-CH, 6'-CH), 7.46-7.43 (2H, *m*, 3'-CH, 5'-CH), 5.51 (2H, *br.s.*, 4-NH_2), 4.23 (2H, *q*, *J* = 7.1 Hz, 6-CH_2), 3.41 (2H, *s*, 2-CH_2), 1.31 (3H, *t*, *J* = 7.1 Hz, 7-CH_3). **^{13}C NMR** (**101 MHz, $CDCl_3$, *δ***): 168.85 (C-1), 163.06 (C-5), 153.73 (C-3), 139.59 (C-1'), 130.81 (C-2', C-6'), 128.88 (C-3', C-5'), 127.78 (C-4'), 61.88 (C-6), 35.85 (C-2), 14.05 (C-7). **UPLC-MS** (**ESI**–): found *m/z* 283.1 $[M – H]^–$; calculated $C_{12}H_{12}ClN_2O_4^–$ 283.1.

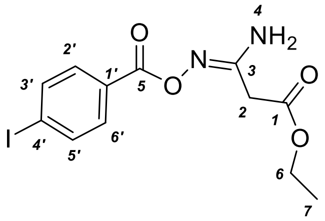

Ethyl 3-amino-3-(((4'-iodobenzoyl)oxy)imino)propanoate (**21b**). Column chromatography eluent – CH_2Cl_2:*iso*-C_3H_7OH (gradient from 100:0 to 95:5). White powder, 140 mg (65%). **^{1}H NMR** (**400 MHz, $CDCl_3$, *δ***): 7.76-7.74 (2H, *m*, 3'-CH, 5'-CH), 7.68-7.66 (2H, *m*, 2'-CH, 6'-CH), 5.39 (2H, *br.s.*, 4-NH_2), 4.15 (2H, *q*, *J* = 7.1 Hz, 6-CH_2), 3.33 (2H, *s*, 2-CH_2), 1.23 (3H, *t*, *J* = 7.1 Hz, 7-CH_3). **^{13}C NMR** (**101 MHz, $CDCl_3$, *δ***): 168.87 (C-1), 163.39 (C-5), 153.73 (C-3), 137.88 (C-3', C-5'), 130.82 (C-2', C-6'), 128.82 (C-1'), 100.84 (C-4'), 61.89 (C-6), 35.81 (C-2), 14.06 (C-7). **UPLC-MS** (**ESI**+): found *m/z* 377.0 $[M + H]^+$; calculated $C_{12}H_{14}IN_2O_4^+$ 377.0.

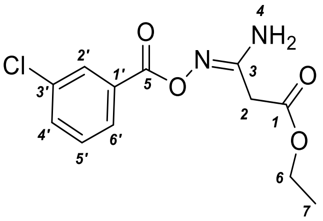

Ethyl 3-amino-3-(((3'-chlorobenzoyl)oxy)imino)propanoate (**21c**). Column chromatography eluent – CH_2Cl_2:*iso*-C_3H_7OH (gradient from 100:0 to 95:5). Light yellow powder, 740 mg (80%). **^{1}H NMR** (**300 MHz, $CDCl_3$, *δ***): 8.03-8.01 (1H, *m*, 2'-CH), 7.96-7.92 (1H, *m*, 6'-CH), 7.58-7.54 (1H, *m*, 4'-CH), 7.44-7.39 (1H, *m*, 5'-CH), 5.52 (2H, *br.s.*, 4-NH_2), 4.24 (2H, *q*, *J* = 7.1 Hz, 6-CH_2), 3.41 (2H, *s*, 2-CH_2), 1.31 (3H, *t*, *J* = 7.2 Hz, 7-CH_3). **^{13}C NMR** (**75 MHz, $CDCl_3$, *δ***): 168.81 (C-1), 162.69 (C-5), 153.80 (C-3), 134.67 (C-3'), 133.12 (C-4'), 131.11 (C-1'), 129.85 (C-2'), 129.42 (C-5'), 127.60 (C-6'), 61.88 (C-6), 35.82 (C-2), 14.03 (C-7). **UPLC-MS** (**ESI**+): found *m/z* 285.1 $[M + H]^+$; calculated $C_{12}H_{14}ClN_2O_4^+$ 285.1.

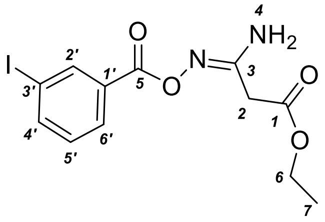

Ethyl 3-amino-3-(((3'-iodobenzoyl)oxy)imino)propanoate (**21d**). Column chromatography eluent – CH_2Cl_2:*iso*-C_3H_7OH (gradient from 100:0 to 95:5). White powder, 480 mg (90%). **^{1}H NMR** (**300 MHz, $CDCl_3$, *δ***): 8.37 (1H, *t*, *J* = 1.7 Hz, 2'-CH), 8.02 (1H, *dt*, *J* = 7.8, 1.4 Hz, 6'-CH), 7.92 (1H, *ddd*, *J* = 7.9, 1.8, 1.1 Hz, 4'-CH), 7.22 (1H, *t*, *J* = 7.9 Hz, 5'-CH), 5.49 (2H, *br.s*, 4-NH_2), 4.24 (2H, *q*, *J* = 7.2 Hz, 6-CH_2), 3.42 (2H, *s*, 2-CH_2), 1.32 (3H, *t*, *J* = 7.1 Hz, 7-CH_3). **^{13}C NMR** (**75 MHz, $CDCl_3$, *δ***): 168.84 (C-1), 162.37 (C-7), 153.78 (C-3), 141.93 (C-4'), 138.17 (C-2'), 131.30 (C-1'), 130.17 (C-5'), 128.62 (C-6'), 93.89 (C-3'), 61.89 (C-6), 35.80 (C-2), 14.05 (C-7). **UPLC-MS** (**ESI**+): found *m/z* 377.0 $[M + H]^+$; calculated $C_{12}H_{14}IN_2O_4^+$ 377.0.

Method D. General procedure of the synthesis of ethyl 3-aryl 1,2,4-oxadiazole-5-carboxylates **3a-f**. The corresponding amidoxime **2a-f** (1 eq) was dissolved in 15 ml of THF with cooling and stirring in a round bottom flask. Then, a solution of ethyl oxalyl chloride (1.5 eq) in 15 ml of THF was added dropwise under cooling, after which DIPEA (2 eq) was added in one portion. The reaction mixture was stirred for 30 min with cooling, then 1 h at r.t. After that, the reaction mixture was refluxed with vigorous stirring until complete conversion of the starting amidoxime **2a-f**, the progress was monitored by TLC (eluent – hexane:EtOAc = 3:1). After completion of the reaction solution was evaporated to dryness using rotary evaporation, the residue was dissolved in EtOAc, washed with deionized water (3·15 ml) and saturated NaCl solution (3·15 ml). The organic layer was dried with anhydrous Na_2SO_4 under vigorous stirring, after which the precipitate was filtered off; the solution was evaporated to dryness and the residue was purified by column chromatography on silica gel (for eluents, see below). The combined organic fractions containing the target ethyl 3-aryl 1,2,4-oxadiazole-5-carboxylates **3a-f** were collected, the solution was evaporated to dryness to give corresponding compounds **3a-f** in good yields.

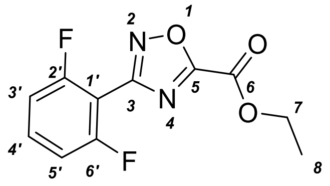

Ethyl 3-(2',6'-difluorophenyl)-1,2,4-oxadiazole-5-carboxylate (**3a**). Column chromatography eluent – CH_2Cl_2:*iso*-C_3H_7OH (gradient from 96:4 to 85:15). Light yellow powder, 320 mg (60%). **^{1}H NMR** (**300 MHz, $CDCl_3$, *δ***): 7.59-7.49 (1H, *m*, 4'-CH), 7.13-7.06 (2H, *m*, 3'-CH, 5'-CH), 4.59 (2H, *q*, *J* = 7.1 Hz, 7-CH_2), 1.50 (3H, *t*, *J* = 7.1 Hz, 8-CH_3). **^{13}C NMR** (**75 MHz, $CDCl_3$, *δ***): 166.77 (C-3), 162.07 (C-5), 160.93 (C-2', C-6', *dd*, J^F = 257.6, 5.5 Hz), 153.83 (C-6), 133.38 (C-4', *t*, J^F = 10.3 Hz), 112.16 (C-3', C-5', *dd*, J^F = 21.7, 3.2 Hz), 104.65 (C-1'), 64.08 (C-7), 14.01 (C-8). **UPLC-MS** (**ESI**+): found *m/z* 255.1 $[M + H]^+$; calculated $C_{11}H_9F_2N_2O_3^+$ 255.1.

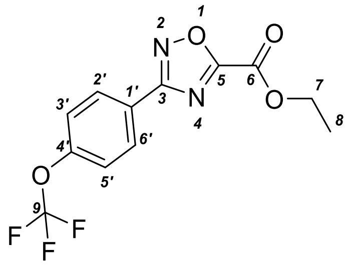

Ethyl 3-(4'-(trifluoromethoxy)phenyl)-1,2,4-oxadiazole-5-carboxylate [9] (**3b**). Column chromatography eluent – hexane:EtOAc (gradient from 9:1 to 5:1). Light yellow powder, 350 mg (80%). **^{1}H NMR** (**300 MHz, DMSO-*d*^{6}, *δ***): 8.22-8.18 (2H, *m*, 2'-CH, 6'-CH), 7.62-7.58 (2H, *m*, 3'-CH, 5'-CH), 4.48 (2H, *q*, *J* = 7.1 Hz, 7-CH_2), 1.39 (3H, *t*, *J* = 7.1 Hz, 8-CH_3). **^{13}C NMR** (**75 MHz, DMSO-*d*^{6}, *δ***): 168.00 (C-3), 167.62 (C-5), 154.03 (C-6), 151.24 (C-4'), 129.98 (C-2', C-6'), 124.96 (C-9), 122.20 (C-3', C-5'), 118.70 (C-1'), 63.85 (C-7), 14.24 (C-8). **UPLC-MS** (**ESI**+): found *m/z* 303.1 $[M + H]^+$; calculated $C_{12}H_{10}F_3N_2O_4^+$ 303.1.

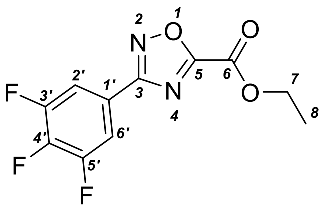

Ethyl 3-(3',4',5'-trifluorophenyl)-1,2,4-oxadiazole-5-carboxylate (**3c**). Column chromatography eluent – hexane:EtOAc = 3:1. Light yellow powder, 200 mg (60%). **^{1}H NMR** (**300 MHz, DMSO-*d*^{6}, *δ***): 8.04-7.93 (2H, *m*, 2'-CH, 6'-CH), 4.48 (2H, *q*, *J* = 7.1 Hz, 7-CH_2), 1.38 (3H, *t*, *J* = 7.1 Hz, 8-CH_3). **^{13}C NMR** (**75 MHz, DMSO-*d*^{6}, *δ***): 167.90 (C-3), 166.94 (C-5), 153.87 (C-6), 151.31 (C-3', C-5', *ddd*, J^F = 249.7, 10.2, 3.9 Hz), 141.75 (C-4', *dt*, J^F = 254.8, 15.3 Hz), 122.52-122.21 (C-1', *m*), 113.06-112.75 (C-2', C-6', *m*), 63.94 (C-7), 14.24 (C-8). **UPLC-MS** (**ESI**+): found *m/z* 273.0 $[M + H]^+$; calculated $C_{11}H_8F_3N_2O_3^+$ 273.0.

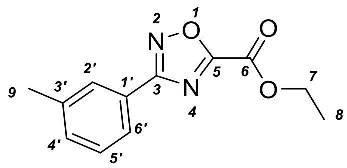

Ethyl 3-(3'-methylphenyl)-1,2,4-oxadiazole-5-carboxylate (**3d**). Column chromatography eluent – hexane:EtOAc = 19:1. Light yellow powder, 260 mg (65%). **^{1}H NMR** (**300 MHz, $CDCl_3$, *δ***): 8.00-7.96 (2H, *m*, 2'-CH, 6'-CH), 7.44-7.35 (2H, *m*, 4'-CH, 5'-CH), 4.59 (2H, *q*, *J* = 7.1 Hz, 7-CH_2), 2.45 (3H, *s*, 9-CH_3), 1.51 (3H, *t*, *J* = 7.1 Hz, 8-CH_3). **^{13}C NMR** (**75 MHz, $CDCl_3$, *δ***): 169.56 (C-3), 166.53 (C-5), 154.21 (C-6), 138.87 (C-3'), 132.61 (C-4'), 128.88 (C-5'), 128.23 (C-2'), 125.51 (C-1'), 124.79 (C-6'), 63.92 (C-7), 21.26 (C-9), 14.04 (C-8). **UPLC-MS** (**ESI**+): found *m/z* 233.1 $[M + H]^+$; calculated $C_{12}H_{13}N_2O_3^+$ 233.1.

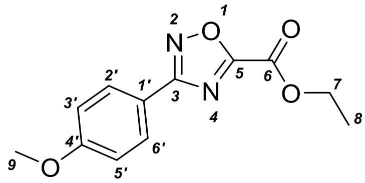

Ethyl 3-(4'-methoxyphenyl)-1,2,4-oxadiazole-5-carboxylate [10] (**3e**). Column chromatography eluent – hexane:EtOAc (gradient from 99:1 to 0:100). Light yellow powder, 400 mg (75%). **^{1}H NMR** (**300 MHz, $CDCl_3$, *δ***): 8.13-8.08 (2H, *m*, 3'-CH, 5'-CH), 7.04-6.99 (2H, *m*, 2'-CH, 6'-CH), 4.58 (2H, *q*, *J* = 7.1 Hz, 7-CH_2), 3.89 (3H, *s*, 9-CH_3), 1.50 (3H, *t*, *J* = 7.1 Hz, 8-CH_3). **^{13}C NMR** (**75 MHz, $CDCl_3$, *δ***): 169.16 (C-3), 166.34 (C-5), 162.44 (C-4'), 154.27 (C-6), 129.36 (C-3', C-5'), 118.06 (C-1'), 114.39 (C-2', C-6'), 63.86 (C-7), 55.41 (C-9), 14.04 (C-8). **UPLC-MS** (**ESI**+): found *m/z* 249.1 $[M + H]^+$; calculated $C_{12}H_{13}N_2O_4^+$ 249.1.

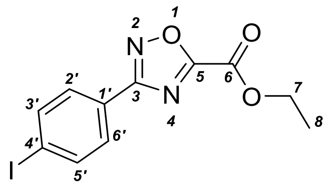

Ethyl 3-(4'-iodophenyl)-1,2,4-oxadiazole-5-carboxylate [9] (**3f**). Column chromatography eluent – hexane:EtOAc (gradient from 98:2 to 95:5). Light yellow powder, 400 mg (60%). **^{1}H NMR** (**300 MHz, $CDCl_3$, *δ***): 7.92-7.86 (4H, *m*, 2'-CH, 3'-CH, 5'-CH, 6'-CH), 4.59 (2H, *q*, *J* = 7.1 Hz, 7-CH_2), 1.51 (3H, *t*, *J* = 7.1 Hz, 8-CH_3). **^{13}C NMR** (**75 MHz, $CDCl_3$, *δ***): 168.93 (C-3), 166.76 (C-5), 154.02 (C-6), 138.29 (C-2', C-6'), 129.09 (C-3', C-5'), 125.14 (C-1'), 98.80 (C-4'), 64.04 (C-7), 14.04 (C-8). **UPLC-MS** (**ESI**+): found *m/z* 345.0 $[M + H]^+$; calculated $C_{11}H_{10}IN_2O_3^+$ 345.0.

Method E. General procedure of the synthesis of methyl 2-(3-aryl-1,2,4-oxadiazol-5-yl)acetates **8a-d** *and methyl 3-(3-aryl-1,2,4-oxadiazol-5-yl)benzoates* **12a-e**. The corresponding *O*-acylamidoxime **7a-d** and **11a-e** (1 eq) was dissolved in 20 ml THF in a round bottom flask. Then, a solution of tetrabutylammonium fluoride (TBAF, 1M solution in THF, 0.5 eq) was added in one portion to the resulting colorless solution. The reaction mixture was refluxed with vigorous stirring for 40–50 min, the progress was monitored by TLC (eluent – CH_2Cl_2:*iso*-C_3H_7OH = 19:1). After completion of the reaction solution was evaporated to dryness using rotary evaporation, the residue was dissolved in 30 ml of EtOAc, washed successively with deionized water (2·15 ml) and saturated NaCl solution (1·15 ml). The

organic layer was dried with anhydrous Na_2SO_4 under vigorous stirring, after which the precipitate was filtered off; the solution was evaporated to dryness and the residue was purified by column chromatography on silica gel (eluent – CH_2Cl_2:*iso*-C_3H_7OH (gradient from 100:0 to 99:1) for methyl 2-(3-aryl-1,2,4-oxadiazol-5-yl)acetates **8a-d**; eluent – CH_2Cl_2:*iso*-C_3H_7OH (gradient from 100:0 to 9:1) for methyl 3-(3-aryl-1,2,4-oxadiazol-5-yl)benzoates **12a-e**). The combined organic fractions containing the target 3,5-disubstituted 1,2,4-oxadiazoles were collected, the solution was evaporated to dryness to give corresponding compounds **7a-d** and **11a-e** in good yields.

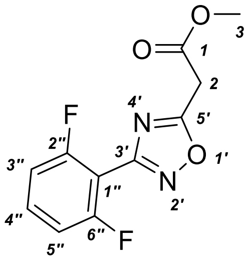

Methyl 2-(3'-(2'',6''-difluorophenyl)-1',2',4'-oxadiazol-5'-yl)acetate (**8a**). Light yellow oil, 130 mg (60%). **^{1}H NMR** (**300 MHz, $CDCl_3$, *δ***): 7.55-7.46 (1H, *m*, 4''-CH), 7.12-7.04 (2H, *m*, 3''-CH, 5''-CH), 4.13 (2H, *s*, 2-CH_2), 3.82 (3H, *s*, 3-CH_3). **^{13}C NMR** (**75 MHz, $CDCl_3$, *δ***): 172.76 (C-5'), 165.88 (C-1), 161.34 (C-3'), 160.98 (C-2'', C-6'', *dd*, J^F = 257.1, 5.7 Hz), 132.81 (C-4'', *t*, J^F = 10.4 Hz), 112.30-111.96 (C-3'', C-5'', *m*), 105.33 (C-1''), 53.06 (C-3), 32.90 (C-2). **UPLC-MS** (**ESI**+): found *m/z* 255.1 $[M + H]^+$; calculated $C_{11}H_9F_2N_2O_3^+$ 255.1.

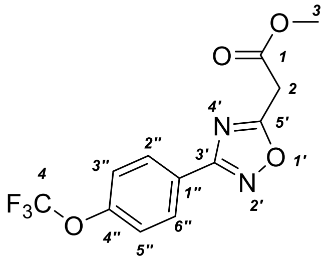

Methyl 2-(3'-(4''-trifluoromethoxy)phenyl)-1',2',4'-oxadiazol-5'-yl)acetate (**8b**). Light yellow oil, 170 mg (75%). **^{1}H NMR** (**300 MHz, $CDCl_3$, *δ***): 8.18-8.13 (2H, *m*, 2''-CH, 6''-CH), 7.36-7.33 (2H, *m*, 3''-CH, 5''-CH), 4.08 (2H, *s*, 2-CH_2), 3.82 (3H, *s*, 3-CH_3). **^{13}C NMR** (**75 MHz, $CDCl_3$, *δ***): 172.84 (C-5'), 167.69 (C-3'), 166.03 (C-1), 151.39 (C-4''), 129.22 (C-2'', C-6''), 125.07 (C-4), 121.07 (C-3'', C-5''), 118.64 (C-1''), 53.05 (C-3), 32.92 (C-2). **UPLC-MS** (**ESI**+): found *m/z* 302.9 $[M + H]^+$; calculated $C_{12}H_{10}F_3N_2O_4^+$ 303.1.

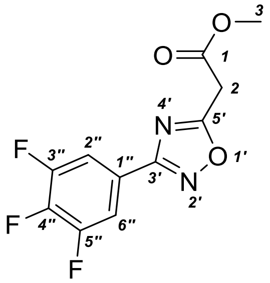

Methyl 2-(3'-(3'',4'',5''-trifluorophenyl)-1',2',4'-oxadiazol-5'-yl)acetate (**8c**). Light yellow oil, 180 mg (60%). **^{1}H NMR (300 MHz, DMSO-*d*^{6}, *δ*)**: 7.95-7.85 (2H, *m*, 2''-CH, 6''-CH), 4.42 (2H, *s*, 2-CH_2), 3.72 (3H, *s*, 3-CH_3). **^{13}C NMR (75 MHz, DMSO-*d*^{6}, *δ*)**: 175.14 (C-5'), 167.08 (C-1), 166.31 (C-3'), 151.25 (C-3'', C-5'', *ddd*, J^F = 249.0, 10.1, 3.8 Hz), 141.48 (C-4'', *dt*, J^F = 254.3, 15.4 Hz), 123.05-122.74 (C-1'', *m*), 112.79-112.48 (C-2'', C-6'', *m*), 53.16 (C-3), 32.91 (C-2). **UPLC-MS** (**ESI–**): found *m/z* 271.0 $[M – H]^–$; calculated $C_{11}H_6F_3N_2O_3^–$ 271.0.

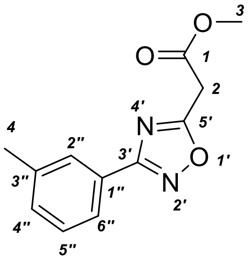

Methyl 2-(3'-(3''-methylphenyl)-1',2',4'-oxadiazol-5'-yl)acetate (**8d**). Light yellow oil, 120 mg (70%). **^{1}H NMR** (**400 MHz, $CDCl_3$, *δ***): 7.93-7.89 (2H, *m*, 2''-CH, 6''-CH), 7.41-7.33 (2H, *m*, 5''-CH, 4''-CH), 4.08 (2H, *s*, 2-CH_2), 3.82 (3H, *s*, 3-CH_2), 2.44 (3H, *s*, 4-CH_3). **^{13}C NMR** (**101 MHz, $CDCl_3$, *δ***): 172.43 (C-5'), 168.80 (C-3'), 166.16 (C-1), 138.69 (C-3''), 132.11 (C-4''), 128.78 (C-5''), 128.03 (C-2''), 126.29 (C-1''), 124.59 (C-6''), 53.03 (C-3), 32.97 (C-2), 21.31 (C-4). **UPLC-MS** (**ESI+**): found *m/z* 233.0 $[M + H]^+$; calculated $C_{12}H_{13}N_2O_3^+$ 233.1.

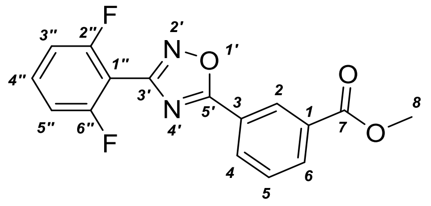

Methyl 3-(3'-(2'',6''-difluorophenyl)-1',2',4'-oxadiazol-5'-yl)benzoate (**12a**). White powder, 130 mg (70%). **^{1}H NMR (400 MHz, $CDCl_3$, *δ*)**: 8.81-8.80 (1H, *m*, 2-CH), 8.33 (1H, *ddd*, J = 7.8, 1.8, 1.2 Hz, 4-CH), 8.23 (1H, *ddd*, J = 7.9, 1.7, 1.2 Hz, 6-CH), 7.59 (1H, *td*, J = 7.8, 0.6 Hz, 5-CH), 7.44 (1H, *tt*, J = 8.5, 6.2 Hz, 4''-CH), 7.05-6.99 (2H, *m*, 3''-CH, 5''-CH), 3.91 (3H, *s*, 8-CH_3). **^{13}C NMR (101 MHz, $CDCl_3$, *δ*)**: 175.14 (C-5'), 165.80 (C-3'), 161.72 (C-7), 161.06 (C-2'', C-6'', *dd*, J^F = 256.8, 5.8 Hz), 133.82 (C-6), 132.79 (C-4'', *t*, J^F = 10.3 Hz), 132.23 (C-4), 131.45 (C-1), 129.45 (C-5), 129.34 (C-2), 124.34 (C-3), 112.26-112.01 (C-3'', C-5'', *m*), 104.97-104.00 (C-1'', *m*), 52.52 (C-8). **UPLC-MS** (**ESI+**): found *m/z* 317.1 $[M + H]^+$; calculated $C_{16}H_{11}F_2N_2O_3^+$ 317.1.

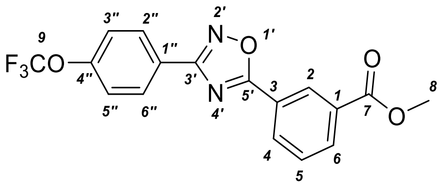

Methyl 3-(3'-(4''-(trifluoromethoxy)phenyl)-1',2',4'-oxadiazol-5'-yl) benzoate (**12b**). White powder, 400 mg (65%). **^{1}H NMR** (**300 MHz, $CDCl_3$, *δ***): 8.90-8.89 (1H, *m*, 2-CH), 8.43-8.40 (1H, *m*, 4-CH), 8.33-8.29 (1H, *m*, 6-CH), 8.28-8.23 (2H, *m*, 2''-CH, 6''-CH), 7.68 (1H, *t*, J = 7.8 Hz, 5-CH), 7.40-7.37 (2H, *m*, 3''-CH, 5''-CH), 4.02 (3H, *s*, 8-CH_3). **^{13}C NMR** (**75 MHz, $CDCl_3$, *δ***): 175.11 (C-5'), 168.08 (C-3'), 165.82 (C-7), 151.39 (C-4''), 133.67 (C-6), 132.11 (C-4), 131.42 (C-1), 129.40 (C-5), 129.30 (C-2'', C-6'', C-2), 125.37 (C-9), 124.54 (C-3), 121.09 (C-3'', C-5''), 118.67 (C-1''), 52.53 (C-8). **UPLC-MS** (**ESI+**): found *m/z* 365.1 $[M + H]^+$; calculated $C_{17}H_{12}F_3N_2O_4^+$ 365.1.

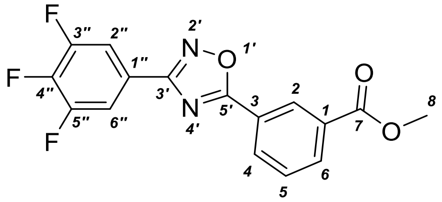

Methyl 3-(3'-(3'',4'',5''-trifluorophenyl)-1',2',4'-oxadiazol-5'-yl) benzoate (**12c**). White powder, 100 mg (60%). **UPLC-MS** (**ESI+**): found *m/z* 335.1 $[M + H]^+$; calculated $C_{16}H_{10}F_3N_2O_3^+$ 335.1. The ester **12c** was hydrolyzed without isolation and characterization.

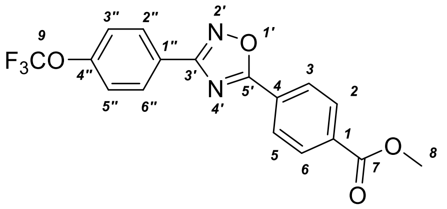

Methyl 4-(3'-(4''-(trifluoromethoxy)phenyl)-1',2',4'-oxadiazol-5'-yl) benzoate (**12d**). White powder, 67 mg (70%). **^{1}H NMR** (**300 MHz, DMSO-*d*^{6}, *δ***): 8.35-8.31 (2H, *m*, 2''-CH, 6''-CH), 8.25-8.19 (4H, *m*, 3-CH, 5-CH, 2-CH, 6-CH), 7.61-7.56 (2H, *m*, 3''-CH, 5''-CH), 3.94 (3H, *s*, 8-CH_3). **^{13}C NMR** (**75 MHz, DMSO-*d*^{6}, *δ***): 175.40 (C-5'), 168.07 (C-7), 165.81 (C-3'), 151.19 (C-4''), 134.19 (C-1), 130.59 (C-2, C-6), 129.91 (C-3, C-5), 128.81 (C-2'', C-6''), 127.55 (C-9), 125.68 (C-4), 121.98 (C-3'', C-5''), 52.93 (C-8). **UPLC-MS** (**ESI+**): found *m/z* 365.1 $[M + H]^+$; calculated $C_{17}H_{12}F_3N_2O_4^+$ 365.1.

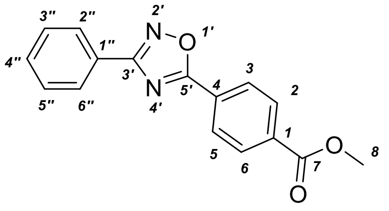

Methyl 4-(3'-phenyl-1',2',4'-oxadiazol-5'-yl)benzoate (**12e**). White powder, 185 mg (75%). **^{1}H NMR** (**400 MHz, DMSO-*d*^{6}, *δ***): 8.32-8.30 (2H, *m*, 2-CH, 6-CH), 8.20-8.18 (2H, *m*, 3-CH, 5-CH), 8.11-8.09 (2H, *m*, 2''-CH, 6''-CH), 7.63-7.61 (3H, *m*, 3''-CH, 4''-CH, 5''-CH), 3.91 (3H, *s*, 8-CH_3). **^{13}C NMR** (**101 MHz, DMSO-*d*^{6}, *δ***): 175.03 (C-5'), 168.91 (C-7), 165.78 (C-3'), 133.85 (C-1), 132.25 (C-4''), 130.61 (C-3, C-5), 129.77 (C-3'', C-5''), 128.79 (C-2, C-6), 127.81 (C-4), 127.59 (C-2'', C-6''), 126.41 (C-1''), 53.05 (C-8). **UPLC-MS** (**ESI+**): found *m/z* 281.1 $[M + H]^+$; calculated $C_{16}H_{13}N_2O_3^+$ 281.1.

Method F. General procedure of the synthesis of ethyl 2-(5-aryl-1,2,4-oxadiazol-3-yl)acetates **22a-d**. *O*-Acylated amidoximes **21a-d** were dissolved in 20 ml of toluene in a round bottom flask and then were refluxed for 8–10 h using a Dean-Stark trap; the progress was monitored by TLC (eluent – CH_2Cl_2:*iso*-C_3H_7OH = 19:1). After completion of the reaction solution was evaporated to dryness using rotary evaporation and the residue was purified by column chromatography on silica gel (for eluents see below). The combined organic fractions containing the target ethyl 2-(5-aryl-1,2,4-oxadiazol-3-yl)acetates **22a-d** were collected, the solution was evaporated to dryness to give corresponding esters **22a-d** in good yields.

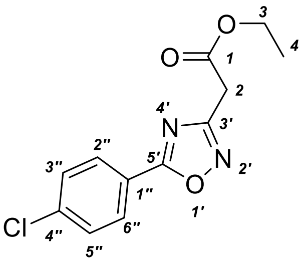

Ethyl 2-(5'-(4"-chlorophenyl)-1',2',4'-oxadiazol-3'-yl)acetate (**22a**). Column chromatography eluent – CH_2Cl_2:*iso*-C_3H_7OH (gradient from 100:0 to 97:3). White powder, 440 mg (65%). **^{1}H NMR** (**300 MHz, $CDCl_3$, *δ***): 8.12-8.07 (2H, *m*, 2"-CH, 6"-CH), 7.55-7.50 (2H, *m*, 3"-CH, 5"-CH), 4.26 (2H, *q*, *J* = 7.1 Hz, 3-CH_2), 3.89 (2H, *s*, 2-CH_2), 1.31 (3H, *t*, *J* = 7.1 Hz, 4-CH_3). **^{13}C NMR** (**75 MHz, $CDCl_3$, *δ***): 175.16 (C-5'), 167.55 (C-1), 165.35 (C-3'), 139.34 (C-1"), 129.51 (C-3", C-5"), 129.43 (C-2", C-6"), 122.47 (C-4"), 61.75 (C-3), 32.38 (C-2), 14.09 (C-4). **UPLC-MS** (**ESI**+): found *m/z* 267.1 $[M + H]^+$; calculated $C_{12}H_{12}ClN_2O_3^+$ 267.1.

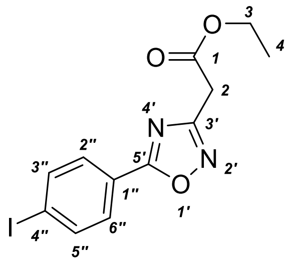

Ethyl 2-(5'-(4"-iodophenyl)-1',2',4'-oxadiazol-3'-yl)acetate (**22b**). Column chromatography eluent – CH_2Cl_2:*iso*-C_3H_7OH (gradient from 100:0 to 99:1). Light yellow powder, 80 mg (60%). **^{1}H NMR** (**400 MHz, $CDCl_3$, *δ***): 7.83-7.76 (4H, *m*, 2"-CH, 6"-CH, 3"-CH, 5"-CH), 4.17 (2H, *q*, *J* = 7.1 Hz, 3-CH_2), 3.81 (2H, *s*, 2-CH_2), 1.22 (3H, *t*, *J* = 7.1 Hz, 4-CH_3). **^{13}C NMR** (**101 MHz, $CDCl_3$, *δ***): 175.46 (C-5'), 167.56 (C-1), 165.36 (C-3'), 138.46 (C-3", C-5"), 129.40 (C-2", C-6"), 123.41 (C-1"), 100.31 (C-4"), 61.78 (C-3), 32.39 (C-2), 14.10 (C-4). **UPLC-MS** (**ESI**+): found *m/z* 359.0 $[M + H]^+$; calculated $C_{12}H_{12}IN_2O_3^+$ 359.0.

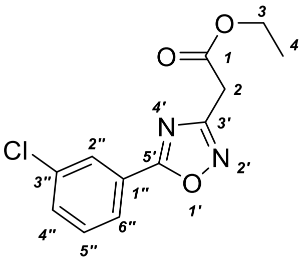

Ethyl 2-(5'-(3"-chlorophenyl)-1',2',4'-oxadiazol-3'-yl)acetate (**22c**). Column chromatography eluent – CH_2Cl_2:*iso*-C_3H_7OH (gradient from 100:0 to 97:3). Light yellow oil, 350 mg (50%). **^{1}H NMR** (**300 MHz, $CDCl_3$, *δ***): 8.16-8.15 (1H, *m*, 2"-CH), 8.04 (1H, *dt*, *J* = 7.7, 1.4 Hz, 6"-CH), 7.59 (1H, *ddd*, *J* = 8.1, 2.1, 1.2 Hz, 4"-CH), 7.51-7.46 (1H, *m*, 5"-CH), 4.27 (2H, *q*, *J* = 7.1 Hz, 3-CH_2), 3.90 (2H, *s*, 2-CH_2), 1.32 (3H, *t*, *J* = 7.1 Hz, 4-CH_3). **^{13}C NMR** (**75 MHz, $CDCl_3$, *δ***): 174.84 (C-5'), 167.50 (C-1), 165.39 (C-3'), 135.31 (C-1"), 132.88 (C-4"), 130.44 (C-5"), 128.18 (C-2"), 126.18 (C-6"), 125.58 (C-3"), 61.78 (C-3), 32.38 (C-2), 14.09 (C-4). **UPLC-MS** (**ESI**+): found *m/z* 267.1 $[M + H]^+$; calculated $C_{12}H_{12}ClN_2O_3^+$ 267.1.

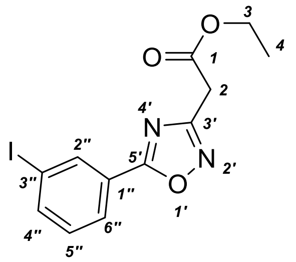

Ethyl 2-(5'-(3"-iodophenyl)-1',2',4'-oxadiazol-3'-yl)acetate (**22d**). Column chromatography eluent – hexane:EtOAc = 8:1. Yellow powder, 200 mg (45%). **^{1}H NMR** (**300 MHz, $CDCl_3$, *δ***): 8.53-8.51 (1H, *m*, 2"-CH), 8.12 (1H, *ddd*, *J* = 7.9, 1.7, 1.1 Hz, 6"-CH), 7.95 (1H, *ddd*, *J* = 7.9, 1.8, 1.1 Hz, 4"-CH), 7.31-7.26 (1H, *m*, 5"-CH), 4.27 (2H, *q*, *J* = 7.1 Hz, 3-CH_2), 3.90 (2H, *s*, 2-CH_2), 1.32 (3H, *t*, *J* = 7.1 Hz, 4-CH_3). **^{13}C NMR** (**75 MHz, $CDCl_3$, *δ***): 174.52 (C-5'), 167.51 (C-1), 165.35 (C-3'), 141.71 (C-4"), 136.84 (C-2"), 130.66 (C-5"), 127.16 (C-6"), 125.77 (C-1"), 94.31 (C-3"), 61.79 (C-3), 32.38 (C-2), 14.10 (C-4). **UPLC-MS** (**ESI**+): found *m/z* 359.0 $[M + H]^+$; calculated $C_{12}H_{12}IN_2O_3^+$ 359.0.

Method G. General procedure of the synthesis of 3-(3-aryl-1,2,4-oxadiazol-5-yl)benzoic acids **13a-e** and *3-(5-(2-fluorophenyl)-1,2,4-oxadiazol-3-yl)benzoic acid* **25**. The corresponding esters **12a-e** and **24** (1 eq) were dissolved in 5 ml THF in a round bottom flask. Then, a solution of NaOH (1.5 M solution in H_2O, 2 eq) was added in one portion to the resulting colorless solution. The reaction mixture was refluxed with vigorous stirring for 6 h, the progress was monitored by TLC (eluent – CH_2Cl_2:*iso*-C_3H_7OH = 9:1). After completion of the reaction, the aqueous layer was separated from the organic layer; the organic layer was further washed with deionized H_2O (2·10 ml). The combined aqueous phase was acidified with 5% solution of H_2SO_4 to pH 3-4. The target 3-(3-aryl-1,2,4-oxadiazol-5-yl)benzoic acids **13a-e** and 3-(5-(2-fluorophenyl)-1,2,4-oxadiazol-3-yl)benzoic acid **25** were obtained in excellent yields as a white precipitate which was filtered off and dried in air overnight.

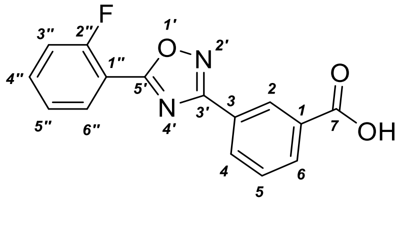

3-(5-(2-Fluorophenyl)-1,2,4-oxadiazol-3-yl)benzoic acid [11] (**25**). White powder, 300 mg (90%). **UPLC-MS** (**ESI–**): found *m/z* 283.0 $[M – H]^-$; calculated $C_{15}H_8FN_2O_3^-$ 283.1. Physicochemical properties of compound **25** are in a good agreement with published data [11].

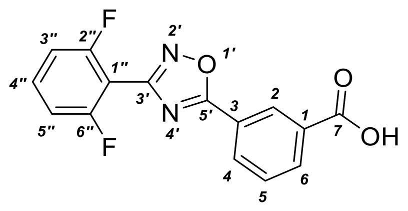

3-(3'-(2'',6''-Difluorophenyl)-1',2',4'-oxadiazol-5'-yl)benzoic acid (**13a**). White powder, 110 mg (95%). **^{1}H NMR (400 MHz, DMSO-*d*^{6}, *δ*)**: 8.66-8.65 (1H, *m*, 2-CH), 8.43-8.40 (1H, *m*, 4-CH), 8.29-8.26 (1H, *m*, 6-CH), 7.82 (1H, *t*, *J* = 7.8 Hz, 5-CH), 7.79-7.73 (1H, *m*, 4''-CH), 7.42-7.37 (2H, *m*, 3''-CH, 5''-CH). **^{13}C NMR (101 MHz, DMSO-*d*^{6}, *δ*)**: 175.42 (C-5'), 166.61 (C-3'), 161.51 (C-7), 160.54 (C-2'', C-6'', *dd*, J^F = 254.7, 5.8 Hz), 134.85 (C-4'', *t*, J^F = 10.6 Hz), 134.39 (C-6), 132.57 (C-1), 132.43 (C-4), 130.78 (C-5), 128.95 (C-2), 123.89 (C-3), 113.29-113.05 (C-3'', C-5'', *m*), 104.97-104.00 (C-1'', *m*). **UPLC-MS** (**ESI–**): found *m/z* 301.1 [M – H]$^-$; calculated $C_{15}H_7F_2N_2O_3^-$ 301.1.

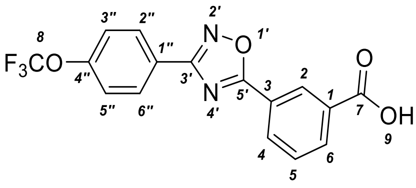

3-(3'-(4''-(Trifluoromethoxy)phenyl)-1',2',4'-oxadiazol-5'-yl)benzoic acid (**13b**). White powder, 90 mg (95%). **^{1}H NMR (300 MHz, DMSO-*d*^{6}, *δ*)**: 13.35 (1H, *br.s.*, 9-OH), 8.67-8.66 (1H, *m*, 2-CH), 8.40-8.36 (1H, *m*, 4-CH), 8.27-8.26 (1H, *m*, 6-CH), 8.25-8.20 (2H, *m*, 2''-CH, 6''-CH), 7.79 (1H, *t*, *J* = 7.8 Hz, 5-CH), 7.59-7.56 (2H, *m*, 3''-CH, 5''-CH). **^{13}C NMR (75 MHz, DMSO-*d*^{6}, *δ*)**: 175.46 (C-5'), 167.80 (C-3'), 166.68 (C-7), 150.99 (C-4''), 134.20 (C-6), 132.90 (C-4), 132.16 (C-1), 130.57 (C-5), 129.88 (C-2'', C-6'') 128.95 (C-2), 125.61 (C-8), 124.07 (C-3), 122.05 (C-3'', C-5''), 118.72 (C-1''). **UPLC-MS** (**ESI–**): found *m/z* 349.1 [M – H]$^-$; calculated $C_{16}H_8F_3N_2O_4^-$ 349.1.

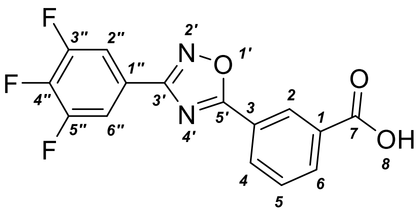

3-(3'-(3'',4'',5''-Trifluorophenyl)-1',2',4'-oxadiazol-5'-yl)benzoic acid (**13c**). White powder, 85 mg (90%). **^{1}H NMR (300 MHz, DMSO-*d*^{6}, *δ*)**: 13.46 (1H, *br.s.*, 8-OH), 8.63-8.61 (1H, *m*, 2-CH), 8.38-8.34 (1H, *m*, 4-CH), 8.26-8.23 (1H, *m*, 6-CH), 8.00-7.90 (2H, *m*, 2''-CH, 6''-CH), 7.79 (1H, *t*, *J* = 7.8 Hz, 5-CH). **^{13}C NMR (75 MHz, DMSO-*d*^{6}, *δ*)**: 175.71 (C-5'), 166.73 (C-3'), 166.54 (C-7), 151.21 (C-3'', C-5'', *ddd*, J^F = 249.3, 10.1, 3.8 Hz), 141.46 (C-4'', *dt*, J^F = 254.5, 15.4 Hz), 134.33 (C-6), 132.54 (C-4), 132.29 (C-1), 130.62 (C-5), 128.95 (C-2), 123.83 (C-3), 123.10-122.86 (C-1'', *m*), 112.84-112.53 (C-2'', C-6'', *m*). **UPLC-MS** (**ESI–**): found *m/z* 319.0 [M – H]$^-$; calculated $C_{15}H_6F_3N_2O_3^-$ 319.0.

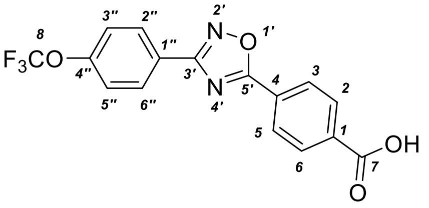

4-(3'-(4''-(Trifluoromethoxy)phenyl)-1',2',4'-oxadiazol-5'-yl)benzoic acid (**13d**). White powder, 61 mg (95%). **UPLC-MS** (**ESI–**): found *m/z* 349.1 [M – H]$^-$; calculated $C_{16}H_8F_3N_2O_4^-$ 349.1. Benzoic acid **13d** was converted to target hydroxamic acid without isolation and characterization.

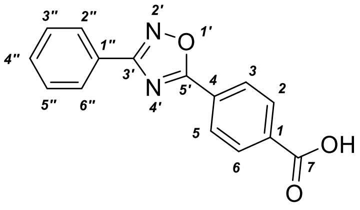

4-(3-phenyl-1,2,4-oxadiazol-5-yl)benzoic acid (**13e**). White powder, 60 mg (95%). **UPLC-MS** (**ESI–**): found *m/z* 265.1 [M – H]$^-$; calculated $C_{15}H_9N_2O_3^-$ 265.1. Benzoic acid **13e** was converted to target hydroxamic acid without isolation and characterization.

Method H. General procedure of the synthesis of 3-(3-aryl-1,2,4-oxadiazol-5-yl)propanoic acids **15a-d**. The corresponding amidoxime **2a-d** (1 eq) and succinic anhydride (1.5 eq) were mixed in a round bottom flask. The resulting mixture was refluxed without the solvent for 1.5 h until a dark brown color appeared. Then, the reaction mixture was cooled to r.t. and 10 ml of CH_2Cl_2 and 10 ml of $NaHCO_3$ saturated aqueous solution were added successively. The reaction mixture was left stirring at r.t. overnight. After completion of the reaction, the aqueous layer was separated from the organic layer; the organic layer was further washed with deionized H_2O (2·10 ml). The combined aqueous phase was acidified with a 5% solution of H_2SO_4 to pH = 3; then extraction with EtOAc (4·20 ml) was carried out. The organic layer was dried with anhydrous Na_2SO_4 under vigorous stirring, after which the precipitate was filtered off; the solution was evaporated to dryness to give 3-(3-aryl-1,2,4-oxadiazol-5-yl)propanoic acids **15a-d** in good yields.

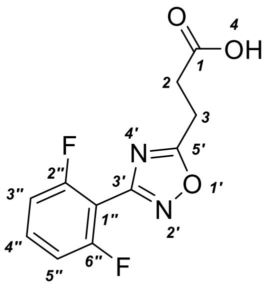

3-(3'-(2",6"-difluorophenyl)-1',2',4'-oxadiazol-5'-yl)propanoic acid (**15a**). Orange amorphous substance, 330 mg (75%). **^{1}H NMR** (**300 MHz, DMSO-*d*^{6}, *δ***): 12.32 (1H, *br.s*, 4-OH), 7.78-7.68 (1H, *m*, 4"-CH), 7.38-7.31 (2H, *m*, 3"-CH, 5"-CH), 3.25 (2H, *t*, *J* = 6.9 Hz, 3-CH_2), 2.84 (2H, *t*, *J* = 6.9 Hz, 2-CH_2). **^{13}C NMR** (**75 MHz, DMSO-*d*^{6}, *δ***): 180.41 (C-5'), 174.01 (C-3'), 173.13 (C-1), 160.46 (C-2", C-6", *dd*, J^F = 254.0, 5.9 Hz), 134.50 (C-4", *t*, J^F = 10.5 Hz), 113.18-112.86 (C-3", C-5", *m*), 105.22 (C-1", *t*, *J* = 18.0 Hz), 30.24 (C-2), 22.13 (C-3). **UPLC-MS** (**ESI**+): found *m/z* 255.1 [M + H]$^+$; calculated $C_{11}H_9F_2N_2O_3^+$ 255.1.

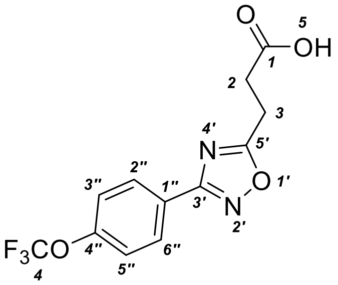

3-(3'-(4"-(trifluoromethoxy)phenyl)-1',2',4'-oxadiazol-5'-yl)propanoic acid (**15b**) [12]. White powder, 380 mg (70%). **^{1}H NMR** (**300 MHz, DMSO-*d*^{6}, *δ***): 12.34 (1H, *br.s*, 5-OH), 8.15-8.10 (2H, *m*, 2"-CH, 6"-CH), 7.59-7.53 (2H, *m*, 3"-CH, 5"-CH), 3.22 (2H, *t*, *J* = 6.9 Hz, 3-CH_2), 2.85 (2H, *t*, *J* = 6.9 Hz, 2-CH_2). **^{13}C NMR** (**75 MHz, DMSO-*d*^{6}, *δ***): 180.52 (C-5'), 174.00 (C-3'), 173.18 (C-1), 150.82 (C-4"), 129.67 (C-2", C-6"), 125.84 (C-1"), 122.08 (C-3", C-5"), 118.72 (C-4), 30.34 (C-2), 22.19 (C-3). **UPLC-MS** (**ESI**+): found *m/z* 303.0 [M + H]$^+$; calculated for $C_{12}H_{10}F_3N_2O_4^+$ 303.1.

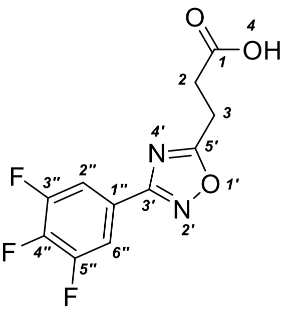

3-(3'-(3",4",5"-trifluorophenyl)-1',2',4'-oxadiazol-5'-yl)propanoic acid (**13c**). White powder, 340 mg (70%). **^{1}H NMR** (**300 MHz, DMSO-*d*^{6}, *δ***): 12.32 (1H, *br.s*, 4-OH), 7.92-7.82 (2H, *m*, 2"-CH, 6"-CH), 3.22 (2H, *t*, *J* = 6.9 Hz, 3-CH_2), 2.85 (2H, *t*, *J* = 6.9 Hz, 2-CH_2). **^{13}C NMR** (**75 MHz, DMSO-*d*^{6}, *δ***): 180.99 (C-5'), 174.00 (C-3'), 173.13 (C-1), 151.22 (C-3", C-5", *ddd*, J^F = 249.3, 10.4, 3.9 Hz), 141.34 (C-4", *dt*, J^F = 253.6, 15.4 Hz), 123.41-123.11 (C-1", *m*), 112.65-112.35 (C-2", C-6", *m*), 30.30 (C-2), 22.21 (C-3). **UPLC-MS** (**ESI**+): found *m/z* 273.0 [M + H]$^+$; calculated for $C_{11}H_8F_3N_2O_3^+$ 273.0.

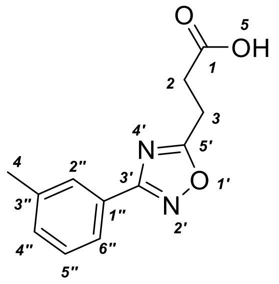

3-(3'-(3"-methylphenyl)-1',2',4'-oxadiazol-5'-yl)propanoic acid (**15d**) [13]. White powder, 370 mg (65%). **^{1}H NMR** (**300 MHz, DMSO-*d*^{6}, *δ***): 12.46 (1H, *br.s*, 5-OH), 7.82-7.77 (2H, *m*, 2"-CH, 6"-CH), 7.47-7.38 (2H, *m*, 4"-CH, 5"-CH), 3.20 (2H, *t*, *J* = 6.9 Hz, 3-CH_2), 2.85 (2H, *t*, *J* = 6.9 Hz, 2-CH_2), 2.40 (3H, *s*, 4-CH_3). **^{13}C NMR** (**75 MHz, DMSO-*d*^{6}, *δ***): 180.04 (C-5'), 173.21 (C-1), 167.96 (C-3'), 139.07 (C-3"), 132.56 (C-4"), 129.58 (C-5"), 127.80 (C-2"), 126.65 (C-1"), 124.58 (C-6"), 30.41 (C-2), 22.19 (C-3), 21.33 (C-4). **UPLC-MS** (**ESI**+): found *m/z* 233.2 [M + H]$^+$; calculated for $C_{12}H_{13}N_2O_3^+$ 233.1.

Method I. General procedure of the synthesis of methyl 3-(3-aryl-1,2,4-oxadiazol-5-yl)propanoates **16a-d**. Two drops of DMF were added to the suspension of the corresponding acid **15a-d** (1 eq) in CH_2Cl_2. The mixture was cooled in an ice bath and when oxalyl chloride was added in one portion (1.2 eq). Then the reaction mixture was intensively stirred for several hours at r.t., the progress was monitored with TLC (eluent – CH_2Cl_2:*iso*-C_3H_7OH = 9:1). After completion of the reaction, the solution was evaporated to dryness using rotary evaporation and the residue was dissolved in CH_3OH and left to stir overnight. Purification of esters **16a-d** obtained was carried out by column chromatography on silica gel, eluent – hexane:EtOAc = 3:1. The combined organic fractions containing the target methyl 3-(3-aryl-1,2,4-oxadiazol-5-yl)propanoates **16a-d** were collected, the solution was evaporated to dryness to give corresponding esters **16a-d** in good yields.

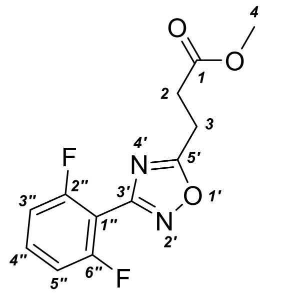

Methyl 3-(3'-(2'',6''-difluorophenyl)-1',2',4'-oxadiazol-5'-yl) propanoate (**16a**). Light yellow amorphous substance, 200 mg (60%). **^{1}H NMR (400 MHz, $CDCl_3$, δ)**: 7.40 (1H, *tt*, *J* = 8.5, 6.2 Hz, 4''-CH), 7.00-6.94 (2H, *m*, 3''-CH, 5''-CH), 3.66 (3H, *s*, 4-CH_3), 3.25 (2H, *t*, *J* = 7.4 Hz, 3-CH_2), 2.88 (2H, *t*, *J* = 7.4 Hz, 2-CH_2). **^{13}C NMR (101 MHz, $CDCl_3$, δ)**: 178.52 (C-5'), 171.59 (C-1), 160.98 (C-2'', C-6'', *dd*, J^F = 256.6, 5.9 Hz), 160.93 (C-3'), 132.62 (C-4'', *t*, J^F = 10.4 Hz), 112.20-111.95 (C-3'', C-5'', *m*), 105.70 (C-1''), 52.11 (C-4), 30.24 (C-2), 22.08 (C-3). **UPLC-MS (ESI+)**: found *m/z* 269.0 $[M + H]^+$; calculated for $C_{12}H_{11}F_2N_2O_3^+$ 269.1.

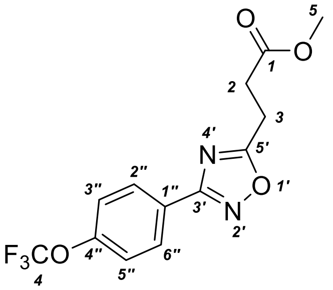

Methyl 3-(3'-(4''-(trifluoromethoxy)phenyl)-1',2',4'-oxadiazol-5'-yl) propanoate (**16b**). Light yellow amorphous substance, 320 mg (80%). **^{1}H NMR (300 MHz, $CDCl_3$, δ)**: 8.15-8.11 (2H, *m*, 2''-CH, 6''-CH), 7.36-7.31 (2H, *m*, 3''-CH, 5''-CH), 3.76 (3H, *s*, 5-CH_3), 3.29 (2H, *t*, *J* = 7.3 Hz, 3-CH_2), 2.97 (2H, *t*, *J* = 7.3 Hz, 2-CH_2). **^{13}C NMR (75 MHz, $CDCl_3$, δ)**: 178.61 (C-5'), 171.65 (C-1), 167.29 (C-3'), 151.25 (C-4''), 129.16 (C-2'', C-6''), 125.38 (C-1''), 121.04 (C-3'', C-5''), 118.65 (C-4), 52.10 (C-5), 30.25 (C-2), 22.05 (C-3). **UPLC-MS (ESI+)**: found *m/z* 317.0 $[M + H]^+$; calculated for $C_{13}H_{12}F_3N_2O_4^+$ 317.1.

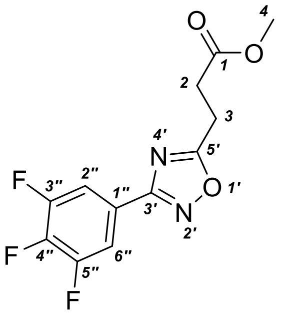

Methyl 3-(3'-(4''-(trifluoromethoxy)phenyl)-1',2',4'-oxadiazol-5'-yl) propanoate (**16c**). Yellow amorphous substance, 285 mg (80%). **^{1}H NMR (400 MHz, $CDCl_3$, δ)**: 7.69-7.61 (2H, *m*, 2''-CH, 6''-CH), 3.67 (3H, *s*, 4-CH_3), 3.19 (2H, *t*, *J* = 7.2 Hz, 3-CH_2), 2.87 (2H, *t*, *J* = 7.2 Hz, 2-CH_2). **^{13}C NMR (101 MHz, $CDCl_3$, δ)**: 179.12 (C-5'), 171.59 (C-1), 166.20 (C-3'), 151.50 (C-3'', C-5'', *ddd*, J^F = 251.4, 10.4, 3.9 Hz), 141.68 (C-4'', *dt*, J^F = 257.1, 15.2 Hz), 122.88-122.71 (C-1'', *m*), 112.07-111.84 (C-2'', C-6'', *m*), 52.15 (C-4), 30.14 (C-2), 22.02 (C-3). **UPLC-MS (ESI+)**: found *m/z* 287.1 $[M + H]^+$; calculated for $C_{12}H_{10}F_3N_2O_3^+$ 287.1.

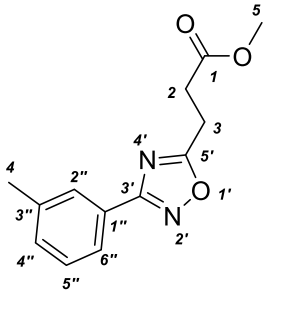

Methyl 3-(3'-(3''-methylphenyl)-1',2',4'-oxadiazol-5'-yl)propanoate (**16d**). Yellow amorphous substance, 310 mg (80%). **UPLC-MS (ESI+)**: found *m/z* 247.1 $[M + H]^+$; calculated for $C_{13}H_{15}N_2O_3^+$ 247.1. The ester **16d** was converted to the target hydroxamic acid without isolation and characterization.

Method J. General procedure of the synthesis of 3-aryl-N-hydroxy-1,2,4-oxadiazole-5-carboxamides **4a-f** and *2-(3-aryl-1,2,4-oxadiazol-5-yl)-N-hydroxyacetamides* **9a-d**. A weighed portion of $NH_2OH{\cdot}HCl$ (4 eq) was dissolved in 15 ml of CH_3OH in a round bottom flask. Then, DIPEA (8 eq) was added in one portion, the resulting solution was stirred in N_2 atmosphere at r.t. for 5-10 min, after which a solution of the corresponding ester **3a-f** and **8a-d** in 10 ml of CH_3OH was added dropwise. The reaction mixture was stirred in N_2 atmosphere at r.t. for 6-10 h, the progress was monitored with TLC (eluent – CH_2Cl_2:*iso*-C_3H_7OH:HCOOH = 19:1:0.1). After completion of the reaction, the 5% solution of H_2SO_4 in deionized H_2O was added to pH = 2; then extraction with EtOAc (2·30 ml) was carried out. Organic layer was additionally washed with saturated solution of NaCl (20 ml), and then was dried with anhydrous Na_2SO_4 under vigorous stirring, after which the precipitate was filtered off; the solution was evaporated to dryness and the residue was purified by column chromatography on silica gel (for eluents, see below). The combined organic fractions containing the target 3-aryl-*N*-hydroxy-1,2,4-oxadiazole-5-carboxamides **4a-f** and 2-(3-aryl-1,2,4-oxadiazol-5-yl)-*N*-hydroxyacetamides were collected, the solution was evaporated to dryness to give corresponding compounds **9a-d** in good yields. The resulting hydroxamic acids **4a-f** and **9a-d** were lyophilized immediately before biological studies.

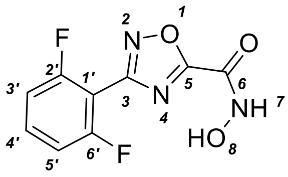

3-(2',6'-Difluorophenyl)-N-hydroxy-1,2,4-oxadiazole-5-carboxamide (**4a**). Column chromatography eluent – CH_2Cl_2:*iso*-C_3H_7OH (gradient from 99:1 to 9:1). Light yellow powder, 270 mg (90%), m.p. 117.4°C (d). **^{1}H NMR** (**300 MHz, DMSO-*d*^{6}, *δ***): 10.53 (1H, *br.s*, 8-OH, 7-NH), 7.82-7.72 (1H, *m*, 4'-CH), 7.47-7.32 (2H, *m*, 3'-CH, 5'-CH). **^{13}C NMR** (**75 MHz, DMSO-*d*^{6}, *δ***): 169.86 (C-3), 160.92 (C-5), 160.45 (C-2', C-6', *dd*, J^F = 254.9, 5.7 Hz), 150.89 (C-6), 135.03 (C-4', *t*, J^F = 10.6 Hz), 113.14 (C-3', C-5', *dd*, J^F = 21.5, 2.8 Hz), 104.49 (C-1', *t*, J^F = 17.7 Hz). **HRMS** (**ESI**–): found *m/z* 240.0221 $[M - H]^-$; calculated $C_9H_4F_2N_3O_3^-$ 240.0226. **UPLC-MS** (**ESI**–): found *m/z* 240.1 $[M - H]^-$; calculated $C_9H_4F_2N_3O_3^-$ 240.0.

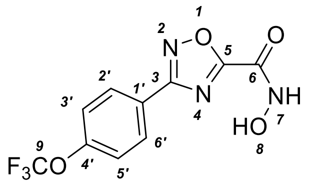

N-Hydroxy-3-(4'-(trifluoromethoxy)phenyl)-1,2,4-oxadiazole-5-carboxamide (**4b**). Column chromatography eluent – CH_2Cl_2:*iso*-C_3H_7OH (gradient from 99:1 to 9:1). White powder, 250 mg (75%), m.p. 108.6°C (d). **^{1}H NMR** (**300 MHz, DMSO-*d*^{6}, *δ***): 10.45 (2H, *br.s*, 7-NH, 8-OH), 8.20-8.16 (2H, *m*, 2'-CH, 6'-CH), 7.64-7.59 (2H, *m*, 3'-CH, 5'-CH). **^{13}C NMR** (**75 MHz, DMSO-*d*^{6}, *δ***): 169.55 (C-3), 167.43 (C-5), 151.14 (C-6), 150.87 (C-4'), 129.96 (C-2', C-6'), 125.19 (C-9), 122.18 (C-3', C-5'), 118.71 (C-1'). **HRMS** (**ESI**–): found *m/z* 288.0233 $[M - H]^-$; calculated $C_{10}H_5F_3N_3O_4^-$ 288.0238. **UPLC-MS** (**ESI**–): found *m/z* 288.1 $[M - H]^-$; calculated $C_{10}H_5F_3N_3O_4^-$ 288.0.

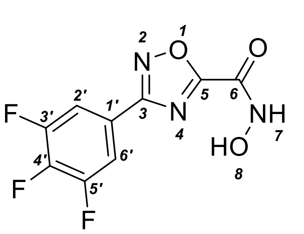

N-Hydroxy-3-(3',4',5'-trifluorophenyl)-1,2,4-oxadiazole-5-carboxamide (**4c**). An analytically pure product was obtained without chromatographic purification. Light yellow powder, 170 mg (90%), m.p. 133.6°C (d). **^{1}H NMR** (**300 MHz, DMSO-*d*^{6}, *δ***): 12.26 (1H, *br.s*, 8-OH), 10.04 (1H, *br.s*, 7-NH), 7.99-7.89 (2H, *m*, 2'-CH, 6'-CH). **^{13}C NMR** (**75 MHz, DMSO-*d*^{6}, *δ***): 169.48 (C-3), 166.40 (C-5), 151.27 (C-3', C-5', *ddd*, J^F = 249.2, 10.1, 3.8 Hz), 150.38 (C-6), 141.66 (C-4', *dt*, J^F = 254.7, 15.4 Hz), 122.69-122.38 (C-1', *m*), 113.02-112.71 (C-2', C-6', *m*). **HRMS** (**ESI**–): found *m/z* 258.0136 $[M - H]^-$; calculated $C_9H_3F_3N_3O_3^-$ 258.0132. **UPLC-MS** (**ESI**–): found *m/z* 258.0 $[M - H]^-$; calculated $C_9H_3F_3N_3O_3^-$ 258.0.

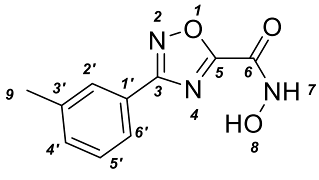

N-Hydroxy-3-(3'-methylphenyl)-1,2,4-oxadiazole-5-carboxamide (**4d**). An analytically pure product was obtained without chromatographic purification. White powder, 240 mg (98%), m.p. 106.0°C (d). **^{1}H NMR** (**300 MHz, DMSO-*d*^{6}, *δ***): 12.25 (1H, *br.s*, 8-OH), 9.92 (1H, *br.s*, 7-NH), 7.89-7.83 (2H, *m*, 2'-CH, 6'-CH), 7.52-7.43 (2H, *m*, 4'-CH, 5'-CH), 2.42 (3H, *s*, 9-CH_3). **^{13}C NMR** (**75 MHz, DMSO-*d*^{6}, *δ***): 168.93 (C-3), 168.50 (C-5), 150.84 (C-6), 139.27 (C-3'), 133.10 (C-4'), 129.74 (C-5'), 128.05 (C-2'), 125.86 (C-1'), 124.82 (C-6'), 21.33 (C-9). **HRMS** (**ESI**–): found *m/z* 218.0565 $[M - H]^-$; calculated $C_{10}H_8N_3O_3^-$ 218.0571. **UPLC-MS** (**ESI**–): found *m/z* 218.1 $[M - H]^-$; calculated $C_{10}H_8N_3O_3^-$ 218.1.

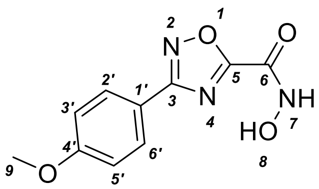

N-Hydroxy-3-(4'-methoxyphenyl)-1,2,4-oxadiazole-5-carboxamide (**4e**). Column chromatography eluent – CH_2Cl_2:*iso*-C_3H_7OH (gradient from 99:1 to 9:1). Light yellow powder, 340 mg (90%), m.p. 144.0°C (d). **^{1}H NMR** (**300 MHz, DMSO-*d*^{6}, *δ***): 11.54-10.29 (2H, *br.s*, 8-OH, 7-NH), 8.01-7.97 (2H, *m*, 3'-CH, 5'-CH), 7.17-7.13 (2H, *m*, 2'-CH, 6'-CH), 3.85 (3H, *s*, 9-CH_3). **^{13}C NMR** (**75 MHz, DMSO-*d*^{6}, *δ***): 169.05 (C-3), 168.12 (C-5), 162.48 (C-4'), 151.11 (C-6), 129.37 (C-3', C-5'), 118.22 (C-1'), 115.25 (C-2', C-6'), 55.93 (C-9). **HRMS** (**ESI**–): found *m/z* 234.0515 $[M - H]^-$; calculated $C_{10}H_8N_3O_4^-$ 234.0520. **UPLC-MS** (**ESI**–): found *m/z* 234.1 $[M - H]^-$; calculated $C_{10}H_8N_3O_4^-$ 234.1.

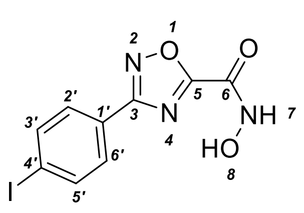

N-Hydroxy-3-(4'-iodophenyl)-1,2,4-oxadiazole-5-carboxamide (**4f**). An analytically pure product was obtained without chromatographic purification. White powder, 380 mg (98%), m.p. 181.3°C (d). **^{1}H NMR** (**300 MHz, DMSO-*d*^{6}, *δ***): 12.27 (1H, *br.s*, 8-OH), 9.95 (1H, *br.s*, 7-NH), 8.02-7.98 (2H, *m*, 3'-CH, 5'-CH), 7.84-7.80 (2H, *m*, 2'-CH, 6'-CH). **^{13}C NMR** (**75 MHz, DMSO-*d*^{6}, *δ***): 169.11 (C-3), 168.01 (C-5), 150.68 (C-6), 138.78 (C-2', C-6'), 129.37 (C-3', C-5'), 125.42 (C-1'), 100.02 (C-4'). **HRMS** (**ESI**–): found *m/z* 329.9372 $[M - H]^-$; calculated $C_9H_5IN_3O_3^-$ 329.9381. **UPLC-MS** (**ESI**+): found *m/z* 332.0 $[M + H]^+$; calculated $C_9H_7IN_3O_3^+$ 332.0.

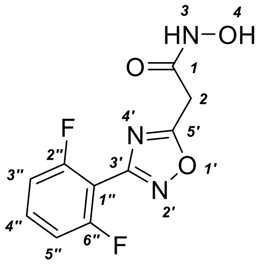

2-(3'-(2'',6''-Difluorophenyl)-1',2',4'-oxadiazol-5'-yl)-N-hydroxyacetamide (**9a**). Column chromatography eluent – CH_2Cl_2:*iso*-C_3H_7OH (gradient from 99:1 to 9:1). Light yellow powder, 40 mg (35%), m.p. 149.7°C (d). **^{1}H NMR (300 MHz, DMSO-*d*^{6}, *δ*)**: 10.94 (1H, *br.s*, 4-OH), 9.17 (1H, *s*, 3-NH), 7.79-7.69 (1H, *m*, 4''-CH), 7.40-7.32 (2H, *m*, 3''-CH, 5''-CH), 3.96 (2H, *s*, 2-CH_2). **^{13}C NMR (75 MHz, DMSO-*d*^{6}, *δ*)**: 175.85 (C-5'), 161.90 (C-1), 160.78 (C-3'), 160.47 (C-2'', C-6'', *dd*, J^F = 254.3, 6.0 Hz), 134.63 (C-4'', *t*, J^F = 10.5 Hz), 113.24-112.92 (C-3'', C-5'', *m*), 105.00 (C-1'', *t*, J^F = 17.8 Hz), 31.88 (C-2). **HRMS** (**ESI–**): found *m/z* 254.0380 [M – H]$^-$; calculated $C_{10}H_6F_2N_3O_3^-$ 254.0383 **UPLC-MS** (**ESI–**): found *m/z* 254.0 [M – H]$^-$; calculated $C_{10}H_6F_2N_3O_3^-$ 254.0.

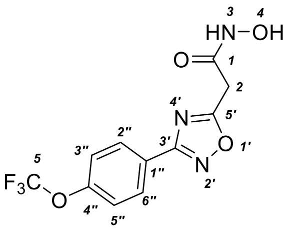

N-Hydroxy-2-(3'-(4''-(trifluoromethoxy)phenyl)-1',2',4'-oxadiazol-5'-yl)acetamide (**9b**). Purified by recrystallization from CH_2Cl_2. White powder, 140 mg (85%), m.p. 177.0°C (d). **^{1}H NMR (300 MHz, DMSO-*d*^{6}, *δ*)**: 10.93 (1H, *br.s.*, 4-OH), 9.17 (1H, *br.s.*, 3-NH), 8.16-8.11 (2H, *m*, 2''-CH, 6''-CH), 7.60-7.55 (2H, *m*, 3''-CH, 5''-CH), 3.93 (2H, *s*, 2-CH_2). **^{13}C NMR (75 MHz, DMSO-*d*^{6}, *δ*)**: 175.96 (C-5'), 167.26 (C-3'), 161.99 (C-1), 150.90 (C-4''), 129.70 (C-2'', C-6''), 125.67 (C-4), 122.10 (C-3'', C-5''), 118.72 (C-1''), 115.31 (C-5), 53.05 (C-3), 32.92 (C-2). **HRMS** (**ESI–**): found *m/z* 302.0393 [M – H]$^-$; calculated $C_{11}H_7F_3N_3O_4^-$ 302.0394. **UPLC-MS** (**ESI–**): found *m/z* 301.9 [M – H]$^-$; calculated $C_{11}H_7F_3N_3O_4^-$ 302.0.

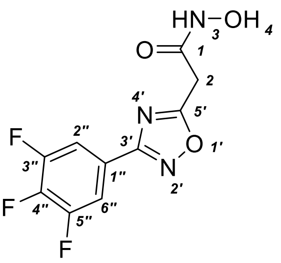

N-Hydroxy-2-(3'-(3'',4'',5''-trifluorophenyl)-1',2',4'-oxadiazol-5'-yl)acetamide (**9c**). Column chromatography eluent – CH_2Cl_2:*iso*-C_3H_7OH (gradient from 99:1 to 9:1). Light yellow powder, 120 mg (70%), m.p. 159.2°C (d). **^{1}H NMR (300 MHz, DMSO-*d*^{6}, *δ*)**: 10.93 (1H, *br.s.*, 4-OH), 9.17 (1H, *s*, 3-NH), 7.94-7.83 (2H, *m*, 2''-CH, 6''-CH), 3.94 (2H, *s*, 2-CH_2). **^{13}C NMR (75 MHz, DMSO-*d*^{6}, *δ*)**: 176.38 (C-5'), 166.21 (C-3'), 161.85 (C-1), 151.24 (C-3'', C-5'', *ddd*, J^F = 249.0, 9.8, 3.8 Hz), 141.42 (C-4'', *dt*, J^F = 254.4, 15.5 Hz), 123.22-122.92 (C-1'', *m*), 112.71-112.40 (C-2'', C-6'', *m*), 31.93 (C-2). **HRMS** (**ESI–**): found *m/z* 272.0289 [M – H]$^-$; calculated $C_{10}H_5F_3N_3O_3^-$ 272.0288. **UPLC-MS** (**ESI–**): found *m/z* 272.0 [M – H]$^-$; calculated $C_{10}H_5F_3N_3O_3^-$ 272.0.

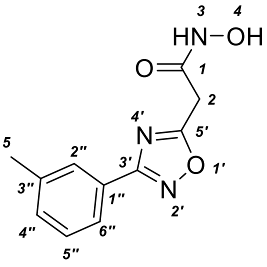

N-Hydroxy-2-(3''-methylphenyl)-1',2',4'-oxadiazol-5'-yl)acetamide (**9d**). Column chromatography eluent – CH_2Cl_2:*iso*-C_3H_7OH (gradient from 99:1 to 9:1). White powder, 70 mg (65%), m.p. 165.4°C (d). **^{1}H NMR (300 MHz, DMSO-*d*^{6}, *δ*)**: 10.94 (1H, *br.s.*, 4-OH), 9.17 (1H, *br.s.*, 3-NH), 7.84-7.82 (1H, *m*, 2''-CH), 7.82-7.78 (1H, *m*, 6''-CH), 7.49-7.42 (1H, *m*, 5''-CH), 7.42-7.39 (1H, *m*, 4''-CH), 3.90 (2H, *s*, 2-CH_2), 2.40 (3H, *s*, 5-CH_3). **^{13}C NMR (75 MHz, DMSO-*d*^{6}, *δ*)**: 175.51 (C-5'), 168.27 (C-3'), 162.08 (C-1), 139.14 (C-3''), 132.68 (C-4''), 129.64 (C-5''), 127.83 (C-2''), 126.46 (C-1''), 124.57 (C-6''), 31.94 (C-2), 21.33 (C-5). **HRMS** (**ESI–**): found *m/z* 232.0724 [M – H]$^-$; calculated $C_{11}H_{10}N_3O_3^-$ 232.0728. **UPLC-MS** (**ESI–**): found *m/z* 232.0 [M – H]$^-$; calculated $C_{11}H_{10}N_3O_3^-$ 232.1.

Method K. General procedure of the synthesis of 3-(3-aryl-1,2,4-oxadiazol-5-yl)-N-hydroxybenzamides **14a-c**, *4-(3-aryl-1,2,4-oxadiazol-5-yl)-N-hydroxybenzamides* **14d-e** and *3-(5-(2-fluorophenyl)-1,2,4-oxadiazol-3-yl)-N-hydroxybenzamide* **26**. The corresponding carboxylic acids **13a-e** and **25** (1 eq) were dissolved in 10 ml of CH_2Cl_2 in a round bottom flask. Then, $SOCl_2$ (10 eq) were added to the resulting solution with vigorous stirring. After the addition of $SOCl_2$ was completed, the reaction mixture was kept at r.t. for 5 h, the progress was monitored by TLC (eluent – CH_2Cl_2:*iso*-C_3H_7OH = 9:1). After completion of the reaction, the solution was evaporated to dryness and the residue was dissolved in 20 ml of an EtOAc:H_2O = 9:1 (v/v) solution containing $NH_2OH{\cdot}HCl$ (2 eq) and Na_2CO_3 (2.5 eq). The reaction mixture was left at r.t. overnight, the formed precipitate of hydroxamic acids **14a-e** and **26** was filtered off and washed with cold EtOAc:H_2O = 4:1. The resulting hydroxamic acids **14a-e** and **26** were lyophilized immediately before biological studies.

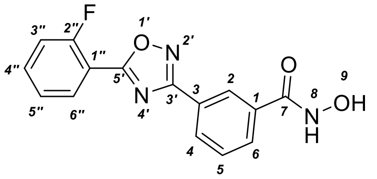

3-(5'-(2''-Fluorophenyl)-1',2',4'-oxadiazol-3'-yl)-N-hydroxybenzamide (**26**). Red powder, 280 mg (90%), m.p. 199.3°C (d). **^{1}H NMR (300 MHz, DMSO-d^6, δ)**: 11.48 (1H, *br.s.*, 9-OH), 9.18 (1H, *br.s.*, 8-NH), 8.50 (1H, *s*, 2-CH), 8.28-8.22 (2H, *m*, 6''-CH, 6-CH), 8.00 (1H, *d*, J = 7.3 Hz, 5-CH), 7.84-7.78 (1H, *m*, 4''-CH), 7.70 (1H, *t*, J = 7.5 Hz, 4-CH), 7.59-7.48 (2H, *m*, 3''-CH, 5''-CH). **^{13}C NMR (75 MHz, DMSO-d^6, δ)**: 173.16 (C-5', *d*, J^F = 4.0 Hz), 168.07 (C-3'), 163.77 (C-7), 160.45 (C-2'', *d*, J^F = 257.8 Hz), 136.25 (C-4'', *d*, J^F = 8.8 Hz), 134.28 (C-1), 131.38 (C-6), 130.33 (C-6''), 130.07 (C-5), 130.00 (C-4), 126.70 (C-3), 126.30 (C-2), 125.97 (C-5'', *d*, J^F = 3.3 Hz), 117.78 (C-3'', *d*, J^F = 20.6 Hz), 112.19 (C-1'', *d*, J^F = 11.0 Hz). **HRMS** (**ESI–**): found *m/z* 298.0622 [M – H]$^-$; calculated $C_{15}H_9FN_3O_3^-$ 298.0633. **UPLC-MS** (**ESI–**): found *m/z* 298.0 [M – H]$^-$; calculated $C_{15}H_9FN_3O_3^-$ 298.1.

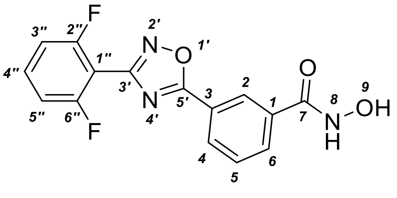

3-(3-(2'',6''-Difluorophenyl)-1',2',4'-oxadiazol-5'-yl)-N-hydroxybenzamide (**14a**). White powder, 100 mg (90%), m.p. 204.2°C (d). **^{1}H NMR (300 MHz, DMSO-d^6, δ)**: 11.47 (1H, *br.s.*, 9-OH), 9.35 (1H, *br.s.*, 8-NH), 8.57-8.56 (1H, *m*, 2-CH), 8.34-8.30 (1H, *m*, 4-CH), 8.14-8.10 (1H, *m*, 6-CH), 7.83-7.73 (2H, *m*, 4''-CH, 5-CH), 7.44-7.36 (2H, *m*, 3''-CH, 5''-CH). **^{13}C NMR (75 MHz, DMSO-d^6, δ)**: 175.62 (C-5'), 163.05 (C-3'), 161.49 (C-7), 160.55 (C-2'', C-6'', *dd*, J^F = 254.5, 5.8 Hz), 134.81 (C-4'', *t*, J^F = 10.5 Hz), 134.50 (C-1), 132.15 (C-6), 130.86 (C-5), 130.42 (C-4), 126.88 (C-2), 123.67 (C-3), 113.14 (C-3'', C-5'', *dd*, J^F = 21.6, 3.1 Hz), 104.97 (C-1'', *t*, J^F = 17.7 Hz). **HRMS** (**ESI–**): found *m/z* 316.0534 [M – H]$^-$; calculated $C_{15}H_8F_2N_3O_3^-$ 316.0539. **UPLC-MS** (**ESI–**): found *m/z* 316.0 [M – H]$^-$; calculated $C_{15}H_8F_2N_3O_3^-$ 316.1.

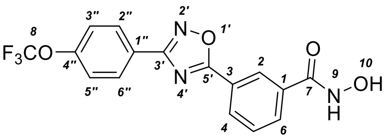

N-Hydroxy-3-(3'-(4''-(trifluoromethoxy)phenyl)-1',2',4'-oxadiazol-5'-yl) benzamide (**14b**). White powder, 80 mg (90%), m.p. 191.0°C (d). **^{1}H NMR (300 MHz, DMSO-d^6, δ)**: 11.54 (1H, *br.s.*, 10-OH), 9.23 (1H, *br.s.*, 9-NH), 8.58-8.57 (1H, *m*, 2-CH), 8.34-8.31 (1H, *m*, 4-CH), 8.27-8.22 (2H, *m*, 2''-CH, 6''-CH), 8.12-8.08 (1H, *m*, 6-CH), 7.77 (1H, *t*, J = 7.8 Hz, 5-CH), 7.63-7.58 (2H, *m*, 3''-CH, 5''-CH). **^{13}C NMR (75 MHz, DMSO-d^6, δ)**: 175.64 (C-5'), 167.84 (C-3'), 163.26 (C-7), 151.02 (C-4''), 134.42 (C-1), 131.97 (C-6), 130.86 (C-4), 130.35 (C-5), 129.89 (C-2'', C-6''), 126.93 (C-2), 125.66 (C-8), 123.95 (C-3), 122.11 (C-3'', C-5''), 118.73 (C-1''). **HRMS** (**ESI–**): found *m/z* 364.0547 [M – H]$^-$; calculated $C_{16}H_9F_3N_3O_4^-$ 364.0551. **UPLC-MS** (**ESI–**): found *m/z* 364.0 [M – H]$^-$; calculated $C_{16}H_9F_3N_3O_4^-$ 364.1.

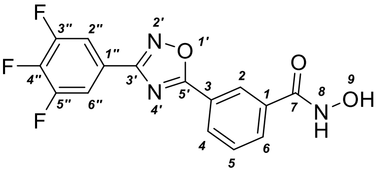

N-Hydroxy-3-(3'-(3'',4'',5''-trifluorophenyl)-1',2',4'-oxadiazol-5'-yl) benzamide (**14c**). White powder, 70 mg (80%), m.p. 188.5°C (d). **^{1}H NMR (300 MHz, DMSO-d^6, δ)**: 11.49 (1H, *br.s.*, 9-OH), 9.25 (1H, *br.s.*, 8-NH), 8.57-7.77 (6H, *m*, 2-CH, 4-CH, 6-CH, 2''-CH, 6''-CH, 5-CH). **^{13}C NMR (75 MHz, DMSO-d^6, δ)**: 175.96 (C-5'), 166.78 (C-3'), 163.03 (C-7), 151.27-149.57 (C-3'', C-5'', *m*), 143.39-139.72 (C-4'', *m*), 134.42 (C-6), 132.07 (C-4), 130.86 (C-1), 130.37 (C-5), 126.98 (C-2), 123.72 (C-3), 123.30-122.90 (C-1'', *m*), 112.88-112.62 (C-2'', C-6'', *m*). **HRMS** (**ESI–**): found *m/z* 334.0441 [M – H]$^-$; calculated $C_{15}H_7F_3N_3O_3^-$ 334.0445. **UPLC-MS** (**ESI–**): found *m/z* 334.0 [M – H]$^-$; calculated $C_{15}H_7F_3N_3O_3^-$ 334.0.

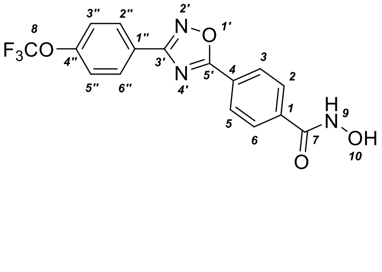

N-Hydroxy-4-(3'-(4''-(trifluoromethoxy)phenyl)-1',2',4'-oxadiazol-5'-yl) benzamide (**14d**). White powder, 50 mg (80%), m.p. 236.0°C (d). **^{1}H NMR (300 MHz, DMSO-d^6, δ)**: 11.35 (1H, *br.s*, 10-OH), 9.32 (1H, *br.s*, 9-NH), 8.29-8.22 (4H, *m*, 2''-CH, 6''-CH, 2-CH, 6-CH), 8.02 (2H, *d*, J = 8.1 Hz, 3-CH, 5-CH), 7.64-7.61 (2H, *m*, 3''-CH, 5''-CH). **^{13}C NMR (75 MHz, DMSO-d^6, δ)**: 175.59 (C-5'), 167.89 (C-3'), 163.25 (C-7), 151.03 (C-4''), 137.53 (C-1), 129.91 (C_{Ar}), 128.57 (C_{Ar}), 128.47 (C_{Ar}), 125.69 (C_{Ar}), 122.16 (C_{Ar}). **HRMS** (**ESI–**): found *m/z* 364.0544 [M – H]$^-$; calculated $C_{16}H_9F_3N_3O_4^-$ 364.0551. **UPLC-MS** (**ESI–**): found *m/z* 364.0 [M – H]$^-$; calculated $C_{16}H_9F_3N_3O_4^-$ 364.1.

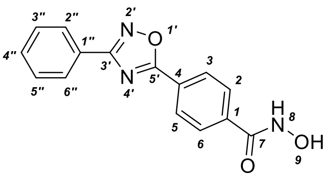

N-Hydroxy-4-(3'-phenyl-1',2',4'-oxadiazol-5'-yl)benzamide (**14e**). White powder, 50 mg (80%), m.p. 265.4°C (d). **^{1}H NMR (300 MHz, DMSO-*d*^{6}, *δ*)**: 11.73 (1H, *br.s*, 9-OH), 10.01 (1H, *br.s*, 8-NH), 8.10-8.07 (4H, *m*, 2-CH, 6-CH, 3-CH, 5-CH), 7.71-7.62 (5H, *m*, 2"-CH, 6"-CH, 3"-CH, 4"-CH, 5"-CH). **^{13}C NMR (75 MHz, DMSO-*d*^{6}, *δ*)**: 175.31 (C-5'), 168.87 (C-7), 163.47 (C-3'), 137.35 (C-1), 132.24 (C-4"), 129.79 (C-3", C-5"), 128.53 (C-2, C-3, C-5, C-6), 127.61 (C-2", C-6"), 126.51 (C-4), 125.92 (C-1"). **HRMS** (**ESI–**): found *m/z* 280.0709 [M – H]$^{–}$; calculated $C_{15}H_{10}N_3O_3^{-}$ 280.0728. **UPLC-MS** (**ESI–**): found *m/z* 280.1 [M – H]$^{–}$; calculated $C_{15}H_{10}N_3O_3^{-}$ 280.1.

Method K. General procedure of the synthesis of 2-(5-aryl-1,2,4-oxadiazol-3-yl)-N-hydroxyacetamides **23a-d**. A weighed portion of $NH_2OH{\cdot}HCl$ (10 eq) was dissolved in 10 ml of CH_3OH in a round bottom flask. Then, DIPEA (20 eq) was added in one portion, the resulting solution was stirred in N_2 atmosphere at r.t. for 10 min, after which a solution of the corresponding esters **22a-d** in 10 ml of CH_3OH was added in one portion. The resulting solution was refluxed with vigorous stirring for 6-8 hours, the progress was monitored with TLC (eluent – CH_2Cl_2:*iso*-C_3H_7OH:HCOOH = 19:1:0.1). After completion of the reaction, the 5% solution of H_2SO_4 in deionized H_2O was added to pH = 2; then extraction with methyl EtOAc (2·20 ml) was carried out. Organic layer was additionally washed with deionized H_2O (2·10 ml) and saturated solution of NaCl (2·10 ml), and then was dried with anhydrous Na_2SO_4 under vigorous stirring, after which the precipitate was filtered off; the solution was evaporated to dryness. The residue was washed twice with a small amount of CH_2Cl_2 (1-2 ml) followed by decantation of the solution. Target hydroxamic acids **23a-d** were obtained by precipitation with *n*-hexane from a saturated solution of the insoluble residue after washing with CH_2Cl_2. The resulting precipitate was filtered off, washed with *n*-hexane, and dried in air to give corresponding 2-(5-aryl-1,2,4-oxadiazol-3-yl)-*N*-hydroxyacetamides in good yields. The resulting hydroxamic acids **23a-d** were lyophilized immediately before biological studies.

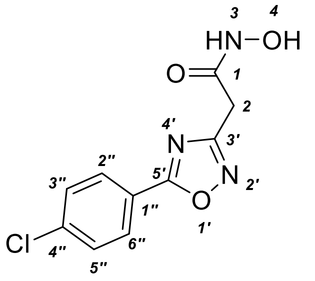

2-(5'-(4"-Chlorophenyl)-1',2',4'-oxadiazol-3'-yl)-N-hydroxyacetamide (**23a**). White powder, 250 mg (60%), m.p. 174.0°C (d). **^{1}H NMR** (**300 MHz, DMSO-*d*^{6}, *δ***): 10.86 (1H, *br.s*, 4-OH), 9.06 (1H, *br.s*, 3-NH), 8.13-8.08 (2H, *m*, 2"-CH, 6"-CH), 7.73-7.68 (1H, *m*, 3"-CH, 5"-CH), 3.62 (2H, *s*, 2-CH_2). **^{13}C NMR** (**75 MHz, DMSO-*d*^{6}, *δ***): 174.61 (C-5'), 166.94 (C-3'), 163.46 (C-1), 138.59 (C-4"), 130.23 (C-3", C-5"), 130.03 (C-2", C-6"), 122.62 (C-1"), 30.95 (C-2). **HRMS** (**ESI**–): found *m/z* 252.0178 [M – H]$^{–}$; calculated $C_{10}H_7ClN_3O_3^{-}$ 252.0181. **UPLC-MS** (**ESI**+): found *m/z* 254.0 [M + H]$^{+}$; calculated $C_{10}H_9ClN_3O_3^{+}$ 254.0.

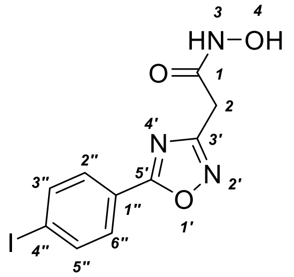

N-Hydroxy-2-(5'-(4"-iodophenyl)-1',2',4'-oxadiazol-3'-yl)acetamide (**23b**). White powder, 50 mg (70%), m.p. 211.6°C (d). **^{1}H NMR** (**300 MHz, DMSO-*d*^{6}, *δ***): 11.03 (1H, *br.s.*, 4-OH), 9.08 (1H, *br.s.*, 3-NH), 8.05-8.01 (2H, *m*, 3"-CH, 5"-CH), 7.87-7.83 (2H, *m*, 2"-CH, 6"-CH), 3.64 (2H, *s*, 2-CH_2). **^{13}C NMR** (**75 MHz, DMSO-*d*^{6}, *δ***): 175.00 (C-5'), 166.99 (C-3'), 163.44 (C-1), 138.98 (C-3", C-5"), 129.76 (C-2", C-6"), 123.14 (C-1"), 102.11 (C-4"), 30.93 (C-2). **HRMS** (**ESI**–): found *m/z* 343.9530 [M – H]$^{–}$; calculated $C_{10}H_7IN_3O_3^{-}$ 343.9538. **UPLC-MS** (**ESI**+): found *m/z* 345.8 [M + H]$^{+}$; calculated $C_{10}H_9IN_3O_3^{+}$ 346.0.

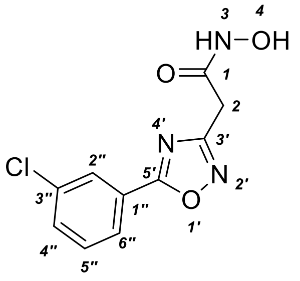

2-(5'-(3"-Chlorophenyl)-1',2',4'-oxadiazol-3'-yl)-N-hydroxyacetamide (**23c**). White powder, 230 mg (70%), m.p. 146.0°C (d). **^{1}H NMR** (**300 MHz, DMSO-*d*^{6}, *δ***): 10.91 (1H, *br.s*, 4-OH), 10.33 (1H, *br.s*, 3-NH), 8.09-8.04 (2H, *m*, 2"-CH, 6"-CH), 7.81-7.77 (1H, *m*, 4"-CH), 7.70-7.65 (1H, *m*, 5"-CH), 3.64 (2H, *s*, 2-CH_2). **^{13}C NMR** (**75 MHz, DMSO-*d*^{6}, *δ***): 174.23 (C-5'), 166.99 (C-3'), 163.40 (C-1), 134.64 (C-3"), 133.55 (C-4"), 132.10 (C-5"), 127.67 (C-2"), 126.93 (C-6"), 125.62 (C-1"), 30.94 (C-2). **HRMS** (**ESI**–): found *m/z* 252.0176 [M – H]$^{–}$; calculated $C_{10}H_7ClN_3O_3^{-}$ 252.0181. **UPLC-MS** (**ESI**+): found *m/z* 254.0 [M + H]$^{+}$; calculated $C_{10}H_9ClN_3O_3^{+}$ 254.0.

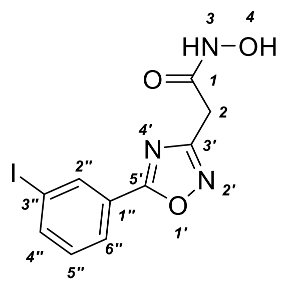

N-Hydroxy-2-(5'-(3''-iodophenyl)-1',2',4'-oxadiazol-3'-yl)acetamide (**23d**). White powder, 100 mg (55%), m.p. 156.3°C (d). **^{1}H NMR (300 MHz, DMSO-*d*^{6}, *δ*)**: 10.83 (1H, *br.s*, 4-OH), 9.05 (1H, *br.s*, 3-NH), 8.39-8.37 (1H, *m*, 6''-CH), 8.12-8.06 (1H, *m*, 2''-CH, 4''-CH), 7.44 (1H, *t*, *J* = 7.9 Hz, 5''-CH), 3.62 (2H, *s*, 2-CH_2). **^{13}C NMR (75 MHz, DMSO-*d*^{6}, *δ*)**: 174.05 (C-5'), 166.91 (C-3'), 163.42 (C-1), 142.20 (C-2''), 136.20 (C-6''), 132.07 (C-5''), 127.48 (C-4''), 125.66 (C-1''), 95.94 (C-3''), 30.96 (C-2). **HRMS** (**ESI**–): found *m/z* 343.9530 [M – H]$^{–}$; calculated $C_{10}H_7IN_3O_3^{–}$ 343.9538. **UPLC-MS** (**ESI**+): found *m/z* 345.9 [M + H]$^{+}$; calculated $C_{10}H_9IN_3O_3^{+}$ 346.0.

Method L. General procedure of the synthesis of N-hydroxy-3-aryl-1,2,4-oxadiazol-5-yl)propanamides **17a-d**. A weighed portion of $NH_2OH{\cdot}HCl$ (30 eq) was dissolved in 40 ml of CH_3OH with a vigorous stirring in a round bottom flask. Then, DIPEA (35 eq) was added in one portion, the resulting solution was stirred in N_2 atmosphere at r.t. for 10 min, after which a solution of the corresponding esters **16a-d** in 10 ml of CH_3OH was added in one portion. The reaction mixture was refluxed for 8-10 h with a vigorous stirring, the progress was monitored with TLC (eluent – CH_2Cl_2:*iso*-C_3H_7OH:HCOOH = 19:1:0.1). After completion of the reaction, the 5% solution of H_2SO_4 in deionized H_2O was added to pH = 3; then extraction with EtOAc (2·20 ml) was carried out. Organic layer was additionally washed with deionized H_2O (2·10 ml) and saturated solution of NaCl (2·10 ml), and then was dried with anhydrous Na_2SO_4 under vigorous stirring, after which the precipitate was filtered off; the solution was evaporated to dryness. For purification of *N*-hydroxy-3-aryl-1,2,4-oxadiazol-5-yl)propanamides obtained see below. The resulting hydroxamic acids **17a-d** were lyophilized immediately before biological studies.

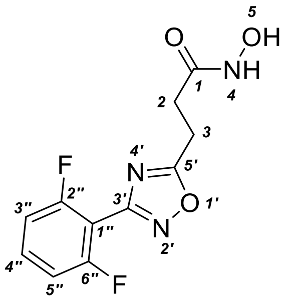

3-(3'-(2'',6''-Difluorophenyl)-1',2',4'-oxadiazol-5'-yl)-N-hydroxypropanamide (**17a**). Purification: column chromatography on silica gel, eluent – CH_2Cl_2:*iso*-C_3H_7OH (gradient from 100:0 to 75:25). Orange powder, 85 mg (45%), m.p. 99.0°C (d). **^{1}H NMR (400 MHz, DMSO-*d*^{6}, *δ*)**: 10.53 (1H, *s*, 5-OH), 8.81 (1H, *s*, 4-NH), 7.73 (1H, *tt*, *J* = 8.6, 6.5 Hz, 4''-CH), 7.38-7.32 (2H, *m*, 3''-CH, 5''-CH), 3.26 (2H, *t*, *J* = 7.1 Hz, 3-CH_2), 2.58 (2H, *t*, *J* = 7.1 Hz, 2-CH_2). **^{13}C NMR (101 MHz, DMSO-*d*^{6}, *δ*)**: 180.45 (C-5'), 167.41 (C-1), 160.47 (C-3'), 160.46 (C-2'', C-6'', *dd*, J^F = 254.0, 5.9 Hz), 134.51 (C-4'', *t*, J^F = 10.4 Hz), 113.17-112.92 (C-3'', C-5'', *m*), 105.43 (C-1''), 28.62 (C-2), 22.23 (C-3). **HRMS** (**ESI**–): found *m/z* 268.0530 [M – H]$^{–}$; calculated $C_{11}H_8F_2N_3O_3^{–}$ 268.0539. **UPLC-MS** (**ESI**+): found *m/z* 270.1 [M + H]$^{+}$; calculated for $C_{11}H_{10}F_2N_3O_3^{+}$ 270.1.

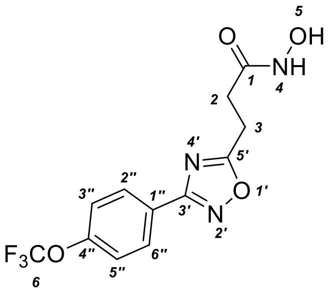

N-Hydroxy-3-(3'-(4''-(trifluoromethoxy)phenyl)-1',2',4'-oxadiazol-5'-yl) propanamide (**17b**). Purification: precipitation with *n*-C_6H_{14} from saturated solution in CH_2Cl_2. Light yellow powder, 160 mg (50%), m.p. 120.0°C (d). **^{1}H NMR (400 MHz, DMSO-*d*^{6}, *δ*)**: 10.66 (1H, *s*, 5-OH), 8.83 (1H, *s*, 4-NH), 8.14-8.10 (2H, *m*, 2''-CH, 6''-CH), 7.58-7.56 (2H, *m*, 3''-CH, 5''-CH), 3.22 (2H, *t*, *J* = 7.21 Hz, 3-CH_2), 2.59 (2H, *t*, *J* = 7.2 Hz, 2-CH_2). **^{13}C NMR (101 MHz, DMSO-*d*^{6}, *δ*)**: 180.59 (C-5'), 167.45 (C-1), 166.94 (C-3'), 150.81 (C-4''), 129.69 (C-2'', C-6''), 125.91 (C-1''), 122.09 (C-3'', C-5''), 121.71 (C-6), 28.80 (C-2), 22.34 (C-3). **HRMS** (**ESI**–): found *m/z* 316.0543 [M – H]$^{–}$; calculated $C_{12}H_9F_3N_3O_4^{–}$ 316.0551. **UPLC-MS** (**ESI**+): found *m/z* 318.0 [M + H]$^{+}$; calculated for $C_{12}H_{11}F_3N_3O_4^{+}$ 318.1.

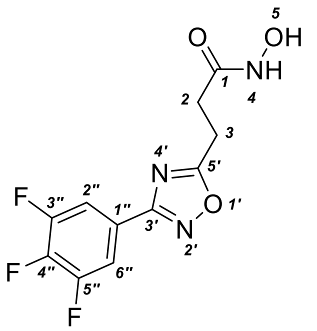

N-Hydroxy-3-(3'-(3'',4'',5''-trifluorophenyl)-1',2',4'-oxadiazol-5'-yl) propanamide (**17c**). Purification: column chromatography on silica gel, eluent – CH_2Cl_2:*iso*-C_3H_7OH (gradient from 100:0 to 75:25). Beige powder, 140 mg (50%), m.p. 122.0°C (d). **^{1}H NMR (400 MHz, DMSO-*d*^{6}, *δ*)**: 10.55 (1H, *s*, 5-OH), 8.81 (1H, *s*, 4-NH), 7.92-7.84 (2H, *m*, 2''-CH, 6''-CH), 3.23 (2H, *t*, *J* = 7.1 Hz, 3-CH_2), 2.58 (2H, *t*, *J* = 7.1 Hz, 2-CH_2). **^{13}C NMR (101 MHz, DMSO-*d*^{6}, *δ*)**: 181.04 (C-5'), 167.44 (C-1), 165.90 (C-3'), 151.23 (C-3'', C-5'', *ddd*, J^F = 248.9, 10.2, 3.9 Hz), 141.34 (C-4'', *dt*, J^F = 254.0, 15.3 Hz), 123.37-123.25 (C-1'', *m*), 112.63-112.40 (C-2'', C-6'', *m*), 28.73 (C-2), 22.34 (C-3). **HRMS** (**ESI**–): found *m/z* 286.0438 [M – H]$^{–}$; calculated $C_{11}H_7F_3N_3O_3^{–}$ 286.0445. **UPLC-MS** (**ESI**+): found *m/z* 288.0 [M + H]$^{+}$; calculated for $C_{11}H_9F_3N_3O_3^{+}$ 288.0.

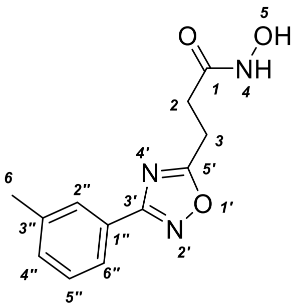

N-Hydroxy-3-(3''-(3''-methylphenyl)-1',2',4'-oxadiazol-5'-yl)propanamide (**17d**). Purification: precipitation with *n*-C_6H_{14} from saturated solution in CH_2Cl_2. Beige powder, 125 mg (40%), m.p. 115.8°C (d). **^{1}H NMR (300 MHz, DMSO-*d*^{6}, *δ*)**: 10.54 (1H, *br.s*, 5-OH), 8.81 (1H, *br.s*, 4-NH), 7.83-7.80 (1H, *m*, 2''-CH), 7.79-7.77 (1H, *m*, 6''-CH), 7.45 (1H, *t*, *J* = 7.3 Hz, 5''-CH), 7.43-7.38 (1H, *m*, 4''-CH), 3.21 (2H, *t*, *J* = 7.2 Hz, 3-CH_2), 2.58 (2H, *t*, *J* = 7.2 Hz, 2-CH_2), 2.40 (3H, *s*, 6-CH_3). **^{13}C NMR (75 MHz, DMSO-*d*^{6}, *δ*)**: 180.07 (C-5'), 167.96 (C-3'), 167.51 (C-1), 139.06 (C-3''), 132.55 (C-4''), 129.58 (C-5''), 127.82 (C-2''), 126.69 (C-1''), 124.58 (C-6''), 28.81 (C-2), 22.29 (C-3), 21.34 (C-6). **HRMS** (**ESI**–): found *m/z* 246.0875 [M – H]$^-$; calculated $C_{12}H_{12}N_3O_3^-$ 246.0884. **UPLC-MS** (**ESI**+): found *m/z* 248.1 [M + H]$^+$; calculated for $C_{12}H_{14}N_3O_3^+$ 248.1.

Synthesis of 1,3,4-oxadiazole derivatives

Method M. General procedure of the synthesis of methoxy-substituted methyl benzoates **27d-f**. To a 50 ml round bottom flask, equipped with a magnetic stir bar and condenser were added dihydroxycarboxylic acid **20f-h** (1 eq), K_2CO_3 (3.1 eq) and $(MeO)_2SO_2$ (3.1 eq) in 30 ml of dry DMF. The suspension was stirred for 8 h at 140°C. Then, the reaction mixture was cooled to r.t., diluted with 10 ml of water and extracted with EtOAc (3·30 ml). The combined organic fractions were washed with brine (3·50 ml) and dried over anhydrous Na_2SO_4. Thereafter, the solvent was distilled off on rotary evaporator. Purification was carried out using column chromatography on silica gel, eluent – hexane:EtOAc = 3:1.

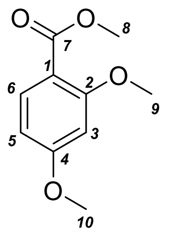

Methyl 2,4-dimethoxybenzoate [15] (**27d**). Colorless oil, 535 mg (85%). **UPLC-MS (ESI+)**: found *m/z* 197.0 [M + H]$^+$; calculated for $C_{10}H_{13}O_4^+$ 197.1. Physicochemical properties of compound **27d** are in a good agreement with published data [15].

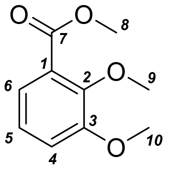

Methyl 2,3-dimethoxybenzoate [16] (**27e**). Colorless solid, 428 mg (65%). **UPLC-MS (ESI+)**: found *m/z* 197.0 [M + H]$^+$; calculated for $C_{10}H_{13}O_4^+$ 197.1. Physicochemical properties of compound **27e** are in a good agreement with published data [16].

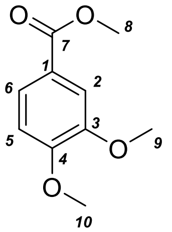

Methyl 3,4-dimethoxybenzoate [14] (**27f**). White solid, 1.08 g (85%). **UPLC-MS (ESI+)**: found *m/z* 197.0 [M + H]$^+$; calculated for $C_{10}H_{13}O_4^+$ 197.1. Physicochemical properties of compound **27f** are in a good agreement with published data [14].

Synthesis of methyl 1H-indole-2-carboxylate (**32**). To a solution of 1*H*-indole-2-carboxylic acid **31** (2 g, 12.34 mmol, 1 eq) in 5 ml of dry DCM was added $SOCl_2$ (3 ml, 41.97 mmol, 3.4 eq), and the resulting mixture was stirred for 24 h at r.t. Then, the solvent and excess of $SOCl_2$ was distilled off on rotary evaporator and 5 ml of dry MeOH was added. After being stirred for 2 h, DIPEA (4.3 ml, 24.69 mmol, 2 eq) was added and the reaction mixture stirred for another 1 h. Thereafter, was added 10 ml of deionized water, product was extracted with EtOAc (3·30 ml), combined organic fractions were washed with brine (3·10 ml) and dried over anhydrous Na_2SO_4. The solvent was distilled off on rotary evaporator. Purification was carried out using column chromatography on silica gel, eluent – hexane: EtOAc = 3:1.

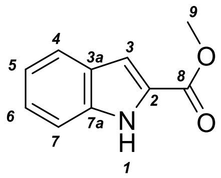

Methyl 1H-indole-2-carboxylate [17] (**32**). Brown solid, 2.04 g (94%). **UPLC-MS (ESI+)**: found *m/z* 176.2 [M+H]$^+$; calculated for $C_{10}H_{10}NO_2^+$ 176.1. Physicochemical properties of compound **32** are in a good agreement with published data [17].

Synthesis of methyl 1-methyl-1H-indole-2-carboxylate (**33**). In a flame-dried, nitrogen-purged 100 ml two-necked round bottom flask, equipped with a magnetic stir bar and dropping funnel, was suspended NaH (513 mg, 12.82 mmol, 1.1 eq) in 10 ml of dry DMF at +5°C. Then, the solution of methyl 1*H*-indole-2-carboxylate **32** (2.04 g, 11.66 mmol, 1 eq) in 12 ml of DMF was added dropwise and the reaction mixture stirred at the same temperature for 30 min. Then, to the resulting solution was added MeI (799 µl, 12.82 mmol, 1.1 eq) and the reaction stirred at r.t. for 1 h. Then 10 ml of deionized water was added and resultant extracted with EtOAc (3·50 ml). The combined organic layers were washed with brine and dried under anhydrous Na_2SO_4. The solvent was distilled off on rotary evaporator. Purification was carried out using column chromatography on silica gel, eluent – hexane: EtOAc = 5:1.

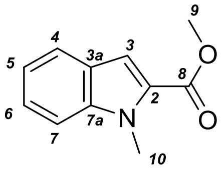

Methyl 1-methyl-1H-indole-2-carboxylate [18] (**33**). White solid, 2.1 g (95%). **UPLC-MS (ESI+)**: found *m/z* 190.1 [M + H]$^+$; calculated for $C_{11}H_{12}NO_2^+$ 190.1. Physicochemical properties of compound **33** are in a good agreement with published data [18].

Method N. General procedure of the synthesis of hydrazides **28 a-f**, **34**. To a solution of methyl benzoates **27a-f** and **33** (1 eq) in 5 ml of MeOH was added $N_2H_4 \cdot H_2O$ (50-60% water solution, 10 eq). The reaction mixture was refluxed for 2 h, the progress was monitored by TLC (eluent – hexane:EtOAc = 3:1), cooled to r.t., concentrated under *vacuo*, dried with toluene (3·10 ml) and lyophilized. The products were used in the next stage of synthesis without purification.

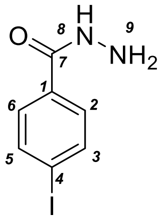

4-Iodobenzohydrazide [23] (**28a**). **UPLC-MS (ESI+)**: found *m/z* 263.0 [M + H]$^+$; calculated for $C_7H_8IN_2O^+$ 263.0. Physicochemical properties of compound **28a** are in a good agreement with published data [23].

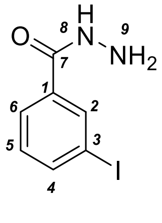

3-Iodobenzohydrazide [23] (**28b**). **UPLC-MS (ESI+)**: found *m/z* 263.1 [M + H]$^+$; calculated for $C_7H_8IN_2O^+$ 263.1. Physicochemical properties of compound **28b** are in a good agreement with published data [23].

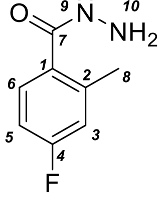

4-Fluoro-2-methylbenzohydrazide [24] (**28c**). **UPLC-MS (ESI+)**: found *m/z* 169.1 [M + H]$^+$; calculated for $C_8H_{10}FN_2O^+$ 169.1. Physicochemical properties of compound **28c** are in a good agreement with published data [24].

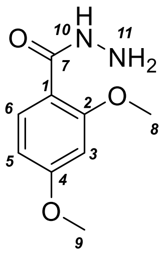

2,4-Dimethoxybenzohydrazide [20] (**28d**). **UPLC-MS (ESI+)**: found *m/z* 197.1 [M + H]$^+$; calculated for $C_9H_{13}N_2O_3^+$ 197.1. Physicochemical properties of compound **28d** are in a good agreement with published data [20].

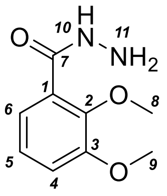

2,3-Dimethoxybenzohydrazide [21] (**28e**). **UPLC-MS (ESI+)**: found *m/z* 197.1 [M + H]$^+$; calculated for $C_9H_{13}N_2O_3^+$ 197.1. Physicochemical properties of compound **28e** are in a good agreement with published data [21].

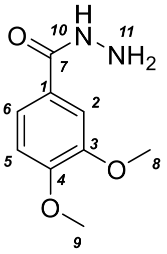

3,4-Dimethoxybenzohydrazide [19] (**28f**). **UPLC-MS (ESI+)**: found *m/z* 197.1 [M + H]$^+$; calculated for $C_9H_{13}N_2O_3^+$ 197.1. Physicochemical properties of compound **28e** are in a good agreement with published data [19].

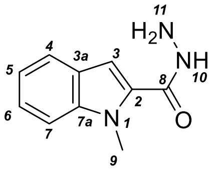

1-Methyl-1H-indole-2-carbohydrazide [22] (**34**). **UPLC-MS (ESI+)**: found *m/z* 190.2 [M + H]$^+$; calculated for $C_{10}H_{12}N_3O^+$ 190.1. Physicochemical properties of compound **34** are in a good agreement with published data [22].

Method O. General procedure of the synthesis of 1,3,4-oxadiazole derivatives **29a-f** and **35**. To a flame-dried, nitrogen-purged 50 ml two-necked round bottom flask, equipped with a magnetic stir bar and dropping funnel, were added corresponding hydrazide **28a-f** and **34** (1 eq), 10 ml of DMF, Et_3N (3 eq), the mixture was stirred for 10 min. Then, the mixture was cooled to +5°C, and a solution of ethyl oxalyl chloride (1 eq) in 10 ml of DMF was added dropwise. The resulting mixture was stirred for 2 h, the progress was monitored by TLC (eluent – hexane:EtOAc = 1:1) and UPLC (ESI+). Then, Et_3N (1 eq), DMAP (1 eq) and solution of TsCl (1 eq) in 5 ml of DMF was added. After being stirred for 8 h, the reaction mixture was diluted with deionized water, extracted with EtOAc. The combined organic layers were washed with brine and dried under anhydrous Na_2SO_4. The solvent was distilled off on rotary evaporator. Purification was carried out using column chromatography on silica gel, eluent – hexane:EtOAc = 3:1.

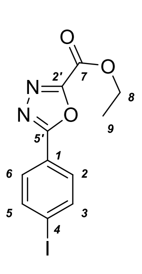

Ethyl 5-(4-iodophenyl)-1,3,4-oxadiazole-2-carboxylate [26] (**29a**). Purple solid, 136 mg (55%). **^{1}H NMR (300 MHz, DMSO-*d*^{6}, δ)**: 8.06-8.00 (2H, *m*, 3-CH, 5-CH), 7.85-7.80 (2H, *m*, 2-CH, 6-CH), 4.47 (2H, *q*, *J* = 7.1 Hz, 8-CH_2), 1.38 (3H, *t*, *J* = 7.1 Hz, 9-CH_3). **^{13}C NMR (75 MHz, DMSO-*d*^{6}, δ)**: 165.44 (C-7), 157.00 (C-2' or C-5'), 154.42 (C-5' or C-2'), 138.99 (C-3, C-5), 129.16 (C-2, C-6), 122.46, (C-4), 101.29 (C-1), 63.45 (C-8), 14.34 (C-9). **UPLC-MS (ESI$^+$)**: found *m/z* 345.1 [M + H]$^+$; calculated for $C_{11}H_{10}IN_2O_3$ 345.1. Physicochemical properties of compound **29a** are in a good agreement with published data [26].

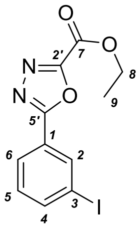

Ethyl 5-(3-iodophenyl)-1,3,4-oxadiazole-2-carboxylate (**29b**). White solid, 385 mg (60%). **^{1}H NMR (400 MHz, DMSO-*d*^{6}, δ)**: 8.33 (1H, *t*, *J* = 1.7 Hz, 2-CH), 8.09-8.05 (2H, *m*, 4-CH, 6-CH), 7.45 (1H, *t*, *J* = 7.9 Hz, 5-CH), 4.47 (2H, *q*, *J* = 7.1 Hz, 8-CH$_2$), 1.38 (3H, *t*, *J* = 7.1 Hz, 9-CH$_3$). **^{13}C NMR (101 MHz, DMSO-*d*^{6}, δ)**: 164.44 (C-7), 157.10 (C-2' or C-5'), 154.40 (C-5' or C-2'), 141.82 (C-4), 135.50 (C-2), 132.10 (C-5), 126.93 (C-6), 125.02 (C-1), 95.94 (C-3), 63.50 (C-8), 14.35 (C-9). **UPLC-MS (ESI$^+$)**: found *m/z* 345.0 [M + H]$^+$; calculated for $C_{11}H_{10}IN_2O_3^+$ 345.0

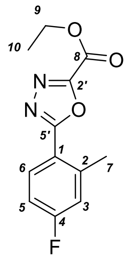

Ethyl 5-(4-fluoro-2-methylphenyl)-1,3,4-oxadiazole-2-carboxylate (**29c**). White solid, 175 mg (45%). **^{1}H NMR (300 MHz, DMSO-*d*^{6}, δ)**: 8.00 (1H, *dd*, *J* = 8.7, 5.9 Hz, 6-CH), 7.41-7.36 (1H, *m*, 3-CH), 7.34-7.27 (1H, *m*, 5-CH), 4.47 (2H, *q*, *J* = 7.1 Hz, 9-CH$_2$), 2.66 (3H, *s*, 7-CH$_3$), 1.38 (3H, *t*, *J* = 7.1 Hz, 10-CH$_3$). **^{13}C NMR (75 MHz, DMSO-*d*^{6}, δ)**: 165.99 (C-8), 164.32 (C-4, *d*, J^F = 251.2 Hz), 156.54 (C-2' or C-5'), 154.55 (C-5' or C-2'), 142.46 (C-1, *d*, J^F = 9.3 Hz), 142.40 (C-2), 132.43 (C-6, *d*, J^F = 9.8 Hz), 119.06 (C-3, *d*, J^F = 21.9 Hz), 114.38 (C-5, *d*, J^F = 22.2 Hz), 63.40 (C-9), 21.69 (C-7), 14.34 (C-10). **UPLC-MS (ESI+)**: found *m/z* 251.1 [M + H]$^+$; calculated for $C_{12}H_{12}FN_2O_3^+$ 251.1.

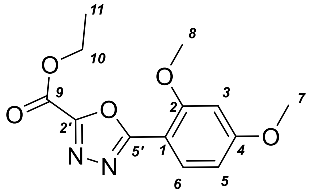

Ethyl 5-(2,4-dimethoxyphenyl)-1,3,4-oxadiazole-2-carboxylate [25] (**29d**). White solid, 385 mg (40%). **^{1}H NMR (300 MHz, DMSO-*d*^{6}, δ)**: 7.81-7.75 (1H, *m*, 6-CH), 6.67-6.60 (2H, *m*, 5-CH, 3-CH), 4.30 (2H, *q*, *J* = 7.1 Hz, 10-CH$_2$), 3.92 (3H, *s*, 7-CH$_3$ or 8-CH$_3$), 3.85 (3H, *s*, 8-CH$_3$ or 7-CH$_3$), 1.31 (3H, *t*, *J* = 7.1 Hz, 11-CH$_3$). **^{13}C NMR (75 MHz, DMSO-*d*^{6}, δ)**: 163.95 (C-9), 163.93 (C-2' or C-5'), 160.43 (C-2 or C-4), 159.34 (C-5' or C-2'), 156.64 (C-4 or C-2), 133.05 (C-6), 113.34 (C-1), 106.36 (C-5), 98.91 (C-3), 62.67 (C-10), 56.47 (C-8), 56.05 (C-7), 14.28 (C-11). **UPLC-MS (ESI+)**: found *m/z* 279.1 [M + H]$^+$; calculated for $C_{13}H_{15}N_2O_5^+$ 279.1. Physicochemical properties of compound **29d** are in a good agreement with published data [25].

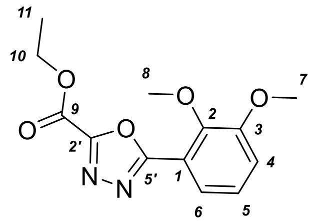

Ethyl 5-(2,3-dimethoxyphenyl)-1,3,4-oxadiazole-2-carboxylate (**29e**). White solid, 135 mg (65%). **^{1}H NMR (300 MHz, DMSO-*d*^{6}, δ)**: 7.49 (1H, *dd*, *J* = 7.7, 1.7 Hz, 6-CH), 7.39 (1H, *dd*, *J* = 8.3, 1.7 Hz, 4-CH), 7.31 (1H, *dd*, *J* = 8.2, 7.7 Hz, 5-CH), 4.46 (2H, *q*, *J* = 7.1 Hz, 10-CH$_2$), 3.90 (3H, *s*, 7-CH$_3$ or 8-CH$_3$), 3.86 (3H, *s*, 8-CH$_3$ or 7-CH$_3$), 1.38 (3H, *t*, *J* = 7.1 Hz, 11-CH$_3$). **^{13}C NMR (75 MHz, DMSO-*d*^{6}, δ)**: 164.35 (C-9), 157.03 (C-2 or C-3), 154.52 (C-3 or C-2), 153.91 (C-2', C-5'), 148.12 (C-1), 125.52 (C-5), 121.51 (C-6), 117.59 (C-4), 63.35 (C-10), 61.43 (C-7 or C-8), 56.56 (C-8 or C-7), 14.32 (C-11). **UPLC-MS (ESI+)**: found *m/z* 279.1 [M + H]$^+$; calculated for $C_{13}H_{15}N_2O_5^+$ 279.1.

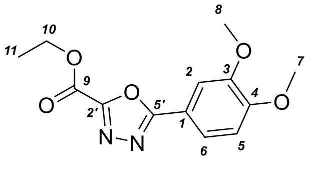

Ethyl 5-(3,4-dimethoxyphenyl)-1,3,4-oxadiazole-2-carboxylate (**29f**). Pinkish solid, 96 mg (50%). **^{1}H NMR (400 MHz, DMSO-*d*^{6}, δ)**: 7.65 (1H, *dd*, *J* = 8.4, 2.1 Hz, 5-CH), 7.53 (1H, *d*, *J* = 2.0 Hz, 2-CH), 7.20 (1H, *d*, *J* = 8.5 Hz, 6-CH), 4.46 (2H, *q*, *J* = 7.1 Hz, 10-CH$_2$), 3.88 (3H, *s*, 7-CH$_3$ or 8-CH$_3$), 3.88 (3H, *s*, 8-CH$_3$ or 7-CH$_3$), 1.38 (3H, *t*, *J* = 7.1 Hz, 11-CH$_3$). **^{13}C NMR (101 MHz, DMSO-*d*^{6}, δ)**: 165.93 (C-9), 156.59 (C-2' or C-5'), 154.55 (C-5' or C-2'), 153.13 (C-3), 149.68 (C-4), 121.46 (C-5), 115.10 (C-1), 112.64 (C-6), 109.99 (C-2), 63.34 (C-10), 56.29 (C-7 or C-8), 56.22 (C-8 or C-7), 14.36 (C-11). **UPLC-MS (ESI+):** found *m/z* 279.1 [M + H]$^+$; calculated for $C_{13}H_{15}N_2O_5^+$ 279.1.

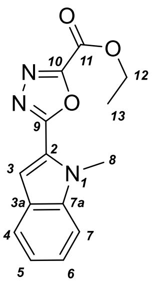

Ethyl 5-(1-methyl-1H-indol-2-yl)-1,3,4-oxadiazole-2-carboxylate (**35**). White solid, 110 mg (30%). **^{1}H NMR (300 MHz, DMSO-*d*^{6}, δ)**: 7.74 (1H, *dt*, *J* = 8.0, 1.0 Hz, 7-CH), 7.66-7.63 (1H, *m*, 4-CH), 7.42-7.36 (2H, *m*, 5-CH, 3-CH), 7.19 (1H, *ddd*, *J* = 7.9, 6.9, 0.9 Hz, 6-CH), 4.48 (2H, *q*, *J* = 7.1 Hz, C-12), 4.17 (3H, *s*, C-8), 1.39 (3H, *t*, *J* = 7.1 Hz, 13-CH$_3$). **^{13}C NMR (75 MHz, DMSO-*d*^{6}, δ)**: 160.89 (C-11), 156.00 (C-9 or C-10), 154.42 (C-10 or C-9), 139.89 (C-7a), 126.73 (C-2), 125.50 (C-5), 122.49 (C-7), 122.33 (C-3a), 121.31 (C-6), 111.38 (C-4), 108.20 (C-3), 63.46 (C-12), 32.63 (C-8), 14.36 (C-13). **UPLC-MS (ESI+)**: found *m/z* 272.1 [M + H]$^+$; calculated for $C_{14}H_{14}N_3O_3^+$ 272.1.

Method P. General procedure of the synthesis of hydroxamic acids **30a-f** and **36**. To a flame-dried, nitrogen-purged 50 ml two-necked round bottom flask, equipped with a magnetic stir bar and dropping funnel, were added dry MeOH, $NH_2OH{\cdot}HCl$ (4 eq), DIPEA (8 eq) and stirred for 5 min at r.t. Then, to the cooled to +5°C reaction mixture was added a solution of corresponding ester **29a-f** and **35** (1 eq) in a dry MeOH dropwise. After being stirred for 8 h at the r.t., the resultant was diluted with 10% H_2SO_4 aqueous solution to pH 5-6, precipitate was filtered, washed with water (3·1 ml), dry MeOH (3·1 ml) and lyophilized.

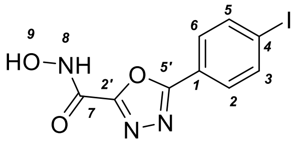

N-Hydroxy-5-(4-iodophenyl)-1,3,4-oxadiazole-2-carboxamide (**30a**). White solid, m.p. 184.4°C (d), 110 mg (85%). **^{1}H NMR (300 MHz, DMSO-*d^6*, δ)**: 12.15 (1H, *br.s*, 9-OH), 9.76 (1H, *br.s*, 8-NH), 8.05-8.01 (2H, *m*, 3-CH, 5-CH), 7.87-7.82 (2H, *m*, 2-CH, 6-CH). **^{13}C NMR (75 MHz, DMSO-*d^6*, δ)**: 164.82 (C-7), 157.96 (C-5'), 151.06 (C-2'), 138.89 (C-3, C-5), 129.10 (C-2, C-6), 122.70 (C-1), 100.88 (C-4). **HRMS (ESI$^-$)**: found *m/z* 329.9367 [M – H]$^-$; calculated for $C_9H_5IN_3O_3^-$ 329.9381. **UPLC-MS** (**ESI**+): found *m/z* 332.0 [M + H]$^+$; calculated $C_9H_7IN_3O_3^+$ 332.0.

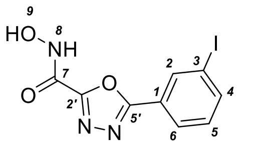

N-Hydroxy-5-(3-iodophenyl)-1,3,4-oxadiazole-2-carboxamide (**30b**). White solid, m.p. 274.9°C (d), 110 mg (85%). **^{1}H NMR (300 MHz, DMSO-*d^6*, δ)**: 8.37 (1H, *s*, 8-NH), 8.27 (1H, *t*, *J* = 1.7 Hz, 2-CH), 8.01-7.97 (2H, *m*, 4-CH, 6-CH), 7.41 (1H, *t*, *J* = 7.9 Hz, 5-CH). **^{13}C NMR (75 MHz, DMSO-*d^6*, δ)**: 163.81 (C-7), 161.78 (C-2'), 154.30 (C-5'), 140.56 (C-4), 134.86 (C-2), 131.95 (C-5), 126.23 (C-6), 126.10 (C-1), 95.80 (C-3). **HRMS (ESI$^-$)**: found *m/z* 329.9363 [M – H]$^-$; calculated for $C_9H_5IN_3O_3^-$ 329.9381. **UPLC-MS** (**ESI**+): found *m/z* 332.0 [M + H]$^+$; calculated $C_9H_7IN_3O_3^+$ 332.0.

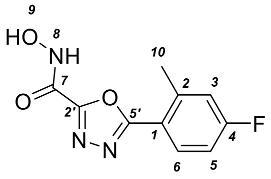

5-(4-Fluoro-2-methylphenyl)-N-hydroxy-1,3,4-oxadiazole-2-carboxamide (**30c**). Pinkish solid, m.p 259.6°C (d), 89 mg (60%). **^{1}H NMR (300 MHz, DMSO-*d^6*, δ)**: 8.50 (1H, *br.s*, 8-NH), 7.93 (1H, *dd*, *J* = 8.7, 6.0 Hz, 6-CH), 7.34 (1H, *dd*, *J* = 10.1, 2.7 Hz, 3-CH), 7.26 (1H, *td*, *J* = 8.5, 2.8 Hz, 5-CH), 2.64 (3H, *s*, 10-CH_3). **^{13}C NMR (75 MHz, DMSO-*d^6*, δ)**: 163.69 (C-4, *d*, J^F = 249.3 Hz), 162.84 (C-7), 162.59 (C-2'), 154.12 (C-5'), 141.54 (C-2, *d*, J^F = 8.8 Hz), 131.63 (C-6, *d*, J^F = 9.5 Hz), 120.09 (C-1, *d*, J^F = 2.7 Hz), 118.79 (C-3, *d*, J^F = 22.0 Hz), 114.07 (C-5, *d*, J^F = 22.0 Hz), 21.81 (C-10). **HRMS (ESI$^-$)**: found *m/z* 236.0463 [M – H]$^-$; calculated for $C_{10}H_7FN_3O_3^-$ 236.0477. **UPLC-MS** (**ESI**+): found *m/z* 238.1 [M + H]$^+$; calculated $C_{10}H_9FN_3O_3^+$ 238.1.

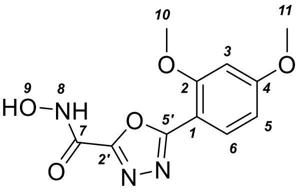

5-(2,4-Dimethoxyphenyl)-N-hydroxy-1,3,4-oxadiazole-2-carboxamide (**30d**). White solid, m.p. 197.3°C (d), 185 mg (65%). **^{1}H NMR (300 MHz, DMSO-*d^6*, δ)**: 12.05 (1H, *br.s*, 9-OH), 9.68 (1H, *s*, 8-NH), 7.86 (1H, *d*, *J* = 8.6 Hz, 6-CH), 6.79 (1H, *d*, *J* = 2.2 Hz, 3-CH), 6.75 (1H, *dd*, *J* = 8.7, 2.3 Hz, 5-CH), 3.92 (3H, *s*, 10-CH_3 or 11-CH_3), 3.88 (3H, *s*, 11-CH_3 or 10-CH_3). **^{13}C NMR (75 MHz, DMSO-*d^6*, δ)**: 164.50 (C-7), 164.14 (C-2' or C-5'), 159.79 (C-2, C-4), 157.32 (C-5' or C-2'), 132.20 (C-6), 106.88 (C-5), 104.61 (C-1), 99.55 (C-3), 56.59 (C-10 or C-11), 56.20 (C-11 or C-10). **HRMS (ESI$^-$)**: found *m/z* 264.0615 [M – H]$^-$; calculated for $C_{11}H_{10}N_3O_5^-$ 264.0626. **UPLC-MS** (**ESI–**): found *m/z* 264.0 [M – H]$^-$; calculated $C_{11}H_{10}N_3O_5^-$ 264.1.

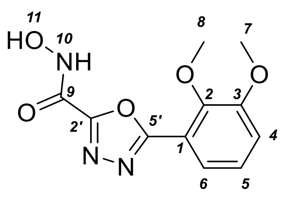

5-(2,3-Dimethoxyphenyl)-N-hydroxy-1,3,4-oxadiazole-2-carboxamide (**30e**). Pinkish solid, m.p. 166.7°C (d), 48 mg (85%). **^{1}H NMR (300 MHz, DMSO-*d^6*, δ)**: 12.11 (1H, *br.s*, 11-OH), 9.74 (1H, *br.s*, 10-NH), 7.49 (1H, *dd*, *J* = 7.6, 1.7 Hz, 6-CH), 7.39-7.27 (2H, *m*, 4-CH, 5-CH), 3.90 (3H, *s*, 7-CH_3 or 8-CH_3), 3.85 (3H, *s*, 8-CH_3 or 7-CH_3). **^{13}C NMR (75 MHz, DMSO-*d^6*, δ)**: 163.65 (C-9), 157.98 (C-2' or C-5'), 153.85 (C-5' or C-2'), 151.29 (C-2 or C-3), 148.04 (C-3 or C-2), 125.41 (C-5), 121.58 (C-6), 117.80 (C-1), 117.31 (C-4), 61.42 (C-7 or C-8), 56.55 (C-8 or C-7). **HRMS (ESI$^-$)**: found *m/z* 264.0614 [M – H]$^-$; calculated for $C_{11}H_{10}N_3O_5^-$ 264.0626. **UPLC-MS** (**ESI–**): found *m/z* 264.1 [M – H]$^-$; calculated $C_{11}H_{10}N_3O_5^-$ 264.1.

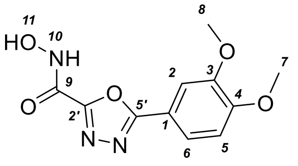

5-(3,4-Dimethoxyphenyl)-N-hydroxy-1,3,4-oxadiazole-2-carboxamide (**30f**). White solid, m.p. 130.7°C (d), 183 mg (65%).**^{1}H NMR (400 MHz, DMSO-*d^6*, δ)**: 11.81 (1H, *br.s*, 11-OH), 9.85 (1H, *br.s*, 10-NH), 7.68 (1H, *dd*, *J* = 8.4, 2.0 Hz, 6-CH), 7.56 (1H, *d*, *J* = 2.0 Hz, 2-CH), 7.20 (1H, *d*, *J* = 8.5 Hz, 5-CH), 3.88 (3H, *s*, 7-CH_3 or 8-CH_3), 3.87 (3H, *s*, 8-CH_3 or 7-CH_3). **^{13}C NMR (101 MHz, DMSO-*d^6*, δ)**: 165.22 (C-9), 157.51 (C-2' or C-5'), 152.85 (C-5' or C-2'), 151.23 (C-4), 149.60 (C-3), 121.26 (C-6), 115.32 (C-1), 112.58 (C-5), 110.01 (C-2), 56.26 (C-7 or C-8), 56.20 (C-8 or C-7). **HRMS (ESI$^-$)**: found *m/z* 264.0610 [M – H]$^-$;

calculated for $C_{11}H_{10}N_3O_5^-$ 264.0626. **UPLC-MS** (**ESI–**): found *m/z* 264.0 $[M-H]^-$; calculated $C_{11}H_{10}N_3O_5^-$ 264.1.

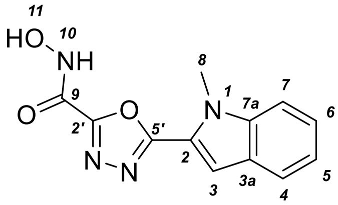

N-Hydroxy-5-(1-methyl-1H-indol-2-yl)-1,3,4-oxadiazole-2-carboxamide (**36**). White solid, m.p 198°C (d), 73 mg (65%). **^{1}H NMR (400 MHz, DMSO-d^6, δ)**: 12.18 (1H, *s*, 11-OH), 9.78 (1H, *s*, 10-NH), 7.75-7.72 (1H, *m*, 7-CH), 7.65-7.63 (1H, *m*, 4-CH), 7.40-7.36 (2H, *m*, 3-CH, 5-CH), 7.20-7.16 (1H, *m*, 6-CH), 4.17 (3H, *s*, 8-CH_3). **^{13}C NMR (101 MHz, DMSO-d^6, δ)**: 160.30 (C-2' or C-5'), 156.98 (C-5' or C-2'), 151.09 (C-9), 139.79 (C-7a), 126.73 (C-2), 125.28 (C-5), 122.63 (C-3a), 122.39 (C-7), 121.24 (C-6), 111.34 (C-4), 107.84 (C-3), 32.59 (C-8). **HRMS (ESI^-)**: found *m/z* 257.0663 $[M-H]^-$; calculated for $C_{12}H_9N_4O_3^-$ 257.0680. **UPLC-MS** (**ESI–**): found *m/z* 259.1 $[M+H]^+$; calculated $C_{12}H_{11}N_4O_3^+$ 259.1.

References

1. Li, Y.; Zhu, H.; Chen, K.; Liu, R.; Khallaf, A.; Zhang, X.; Ni, J. Synthesis, Insecticidal Activity, and Structure-Activity Relationship (SAR) of Anthranilic Diamides Analogs Containing Oxadiazole Rings. *Org. Biomol. Chem.* **2013**, *11*, 3979, doi:10.1039/c3ob40345a.
2. García-Álvarez, R.; Díaz-Álvarez, A.E.; Borge, J.; Crochet, P.; Cadierno, V. Ruthenium-Catalyzed Rearrangement of Aldoximes to Primary Amides in Water. *Organometallics* **2012**, *31*, 6482–6490, doi:10.1021/om3006917.
3. Yang, X.; Ren, H.; Zhou, S.; Li, C.; Liu, C.; Zhou, Y.; He, G.; Liu, H. Rh(III)-Catalyzed Synthesis of Substituted Isoindoles through a Direct C–H Activation/[4 + 1] Annulation and Acyl Migration Cascade of Oxadiazolones with Diazo Compounds. *Org. Lett.* **2023**, *25*, 3195–3199, doi:10.1021/acs.orglett.3c00547.
4. Lacbay, C.M.; Menni, M.; Bernatchez, J.A.; Götte, M.; Tsantrizos, Y.S. Pharmacophore Requirements for HIV-1 Reverse Transcriptase Inhibitors That Selectively "Freeze" the Pre-Translocated Complex during the Polymerization Catalytic Cycle. *Bioorg. Med. Chem.* **2018**, *26*, 1713–1726, doi:10.1016/j.bmc.2018.02.017.
5. Lin, C.-C.; Hsieh, T.-H.; Liao, P.-Y.; Liao, Z.-Y.; Chang, C.-W.; Shih, Y.-C.; Yeh, W.-H.; Chien, T.-C. Practical Synthesis of N-Substituted Cyanamides via Tiemann Rearrangement of Amidoximes. *Org. Lett.* **2014**, *16*, 892–895, doi:10.1021/ol403645y.
6. Shabalin, D.A.; Dunsford, J.J.; Ngwerume, S.; Saunders, A.R.; Gill, D.M.; Camp, J.E. Synthesis of 2,4-Disubstituted Imidazoles via Nucleophilic Catalysis. *Synlett* **2020**, *31*, 797–800, doi:10.1055/s-0039-1690832.
7. Bianchini, G.; Tomassetti, M.; Lillini, S.; Sirico, A.; Bovolenta, S.; Za, L.; Liberati, C.; Novelli, R.; Aramini, A. Discovery of Novel TRPM8 Blockers Suitable for the Treatment of Somatic and Ocular Painful Conditions: A Journey through pK_a and LogD Modulation. *J. Med. Chem.* **2021**, *64*, 16820–16837, doi:10.1021/acs.jmedchem.1c01647.
8. Kumar, N.; Ghosh, S.; Bhunia, S.; Bisai, A. Synthesis of 2-Oxindoles via "transition-Metal-Free" Intramolecular Dehydrogenative Coupling (IDC) of sp^2 C-H and sp^3 C-H Bonds. *Beilstein J. Org. Chem.* **2016**, *12*, 1153–1169, doi:10.3762/bjoc.12.111.
9. Tsuji, A.; Akao, T.; Masuya, T.; Murai, M.; Miyoshi, H. IACS-010759, a Potent Inhibitor of Glycolysis-Deficient Hypoxic Tumor Cells, Inhibits Mitochondrial Respiratory Complex I through a Unique Mechanism. *J. Biol. Chem.* **2020**, *295*, 7481–7491, doi:10.1074/jbc.RA120.013366.
10. Galave, S.A.; Kadam, K.S.; Sonawane, A.D.; Pansare, V.R.; Garud, D.R. A Metal-Free Isoamyl Nitrite Mediated Efficient Synthesis of 1,2,4-Oxadiazoles. *Tetrahedron Lett.* **2023**, *125*, 154616, doi:10.1016/j.tetlet.2023.154616.
11. Welch, E.M.; Barton, E.R.; Zhuo, J.; Tomizawa, Y.; Friesen, W.J.; Trifillis, P.; Paushkin, S.; Patel, M.; Trotta, C.R.; Hwang, S.; et al. PTC124 Targets Genetic Disorders Caused by Nonsense Mutations. *Nature* **2007**, *447*, 87–91, doi:10.1038/nature05756.
12. Beaton, G.; Moree, W.J.; Rueter, J.K.; Dahl, R.S.; Mcelligott, D.L.; Goldman, P.; Demaggio, A.J.; Christenson, E.; Herendeen, D.; Fowler, K.W.; et al. 2*H*-Phthalazin-1-Ones and Methods for Use Thereof 2001, 1–229.
13. Srivastava, R.M.; Seabra, G.M. Preparation and Reactions of 3-[3-(Aryl)-1,2,4-oxadiazol-5-yl]propionic Acids. *J. Braz. Chem. Soc.* **1997**, *8*, 397–405, doi:10.1590/S0103-50531997000400013.
14. Cheng, J.; Zhu, M.; Wang, C.; Li, J.; Jiang, X.; Wei, Y.; Tang, W.; Xue, D.; Xiao, J. Chemoselective Dehydrogenative Esterification of Aldehydes and Alcohols with a Dimeric Rhodium(II) Catalyst. *Chem. Sci.* **2016**, *7*, 4428–4434, doi:10.1039/C6SC00145A.
15. Van Otterlo, W.A.L.; Michael, J.P.; De Koning, C.B. Alternatives to *N,N*-Diethyl-2,4-dimethoxybenzamide as a Precursor for the Synthesis of 6,8-Dimethoxy-3-Methyl-3,4-Dihydro-1*H*-Isochromen-1-One.

Synth. Commun. **2007**, *37*, 3611–3621, doi:10.1080/00397910701557655.

16. Nishimoto, Y.; Babu, S.A.; Yasuda, M.; Baba, A. Esters as Acylating Reagent in a Friedel-Crafts Reaction: Indium Tribromide Catalyzed Acylation of Arenes Using Dimethylchlorosilane. *J. Org. Chem.* **2008**, *73*, 9465–9468, doi:10.1021/jo801914x.
17. Whiting, A.L.; Hof, F. Binding Trimethyllysine and Other Cationic Guests in Water with a Series of Indole-Derived Hosts: Large Differences in Affinity from Subtle Changes in Structure. *Org. Biomol. Chem.* **2012**, *10*, 6885–6892, doi:10.1039/c2ob25882j.
18. Li, Z.; Hong, J.; Weng, L.; Zhou, X. Facile Synthesis of Sulfenyl-Substituted Isocoumarins, Heterocycle-Fused Pyrones and 3-(Inden-1-Ylidene)Isobenzofuranones by $FeCl_3$-Promoted Regioselective Annulation of *o*-(1-Alkynyl)Benzoates and *o*-(1-Alkynyl)Heterocyclic Carboxylates with Disulfides. *Tetrahedron* **2012**, *68*, 1552–1559, doi:10.1016/j.tet.2011.12.003.
19. Silalahi, I.H.; Prokhorov, A.M.; Tanner, T.; Cowling, S.J.; Whitwood, A.C.; Bruce, D.W. The Liquid-Crystalline and Luminescence Properties of Polycatenar Diphenylpyridine Complexes of Palladium(II). *J. Organomet. Chem.* **2022**, *977*, 0–7, doi:10.1016/j.jorganchem.2022.122455.
20. Taha, M.; Ismail, N.H.; Imran, S.; Rokei, M.Q. Bin; Saad, S.M.; Khan, K.M. Synthesis of New Oxadiazole Derivatives as α-Glucosidase Inhibitors. *Bioorganic Med. Chem.* **2015**, *23*, 4155–4162, doi:10.1016/j.bmc.2015.06.060.
21. Kerzare, D.; Chikhale, R.; Bansode, R.; Amnerkar, N.; Karodia, N.; Paradkar, A.; Khedekar, P. Design, Synthesis, Pharmacological Evaluation and Molecular Docking Studies of Substituted Oxadiazolyl-2-Oxoindolinylidene Propane Hydrazide Derivatives. *J. Braz. Chem. Soc.* **2016**, *27*, 1998–2010, doi:10.5935/0103-5053.20160090.
22. Gokhale, N.; Panathur, N.; Dalimba, U.; Kumsi, M. Indole-3-Carbinol and 1,3,4-Oxadiazole Hybrids: Synthesis and Study of Anti-Proliferative and Anti-Microbial Activity. *Aust. J. Chem.* **2015**, *68*, 1603, doi:10.1071/CH15116.
23. Zhang, Y.; Zuniga, C.; Kim, S.J.; Cai, D.; Barlow, S.; Salman, S.; Coropceanu, V.; Brédas, J.L.; Kippelen, B.; Marder, S. Polymers with Carbazole-Oxadiazole Side Chains as Ambipolar Hosts for Phosphorescent Light-Emitting Diodes. *Chem. Mater.* **2011**, *23*, 4002–4015, doi:10.1021/cm201562p.
24. Su, S.; Zhou, X.; Zhou, Y.; Liao, G.; Shi, L.; Yang, X.; Zhang, X.; Jin, L. Synthesis and Biological Evaluation of Novel Sulfone Derivatives Containing 1,3,4-Oxadiazole Moiety. *World J. Org. Chem.* **2014**, 10, doi:10.12691/wjoc-2-1-3.
25. Li, J.; Lu, X.C.; Xu, Y.; Wen, J.X.; Hou, G.Q.; Liu, L. Photoredox Catalysis Enables Decarboxylative Cyclization with Hypervalent Iodine(III) Reagents: Access to 2,5-Disubstituted 1,3,4-Oxadiazoles. *Org. Lett.* **2020**, *22*, 9621–9626, doi:10.1021/acs.orglett.0c03663.
26. Huang, H.; Zou, X.; Cao, S.; Peng, Z.; Peng, Y.; Wang, X. N-Heterocyclic Carbene-Catalyzed Cyclization of Aldehydes with α-Diazo Iodonium Triflate: Facile Access to 2,5-Disubstituted 1,3,4-Oxadiazoles. *Org. Lett.* **2021**, *23*, 4185–4190, doi:10.1021/acs.orglett.1c01128.

^{1}H, ^{13}C NMR spectra for *O*-acylamidoximes synthesized

^{1}H NMR spectrum of compound **7a**

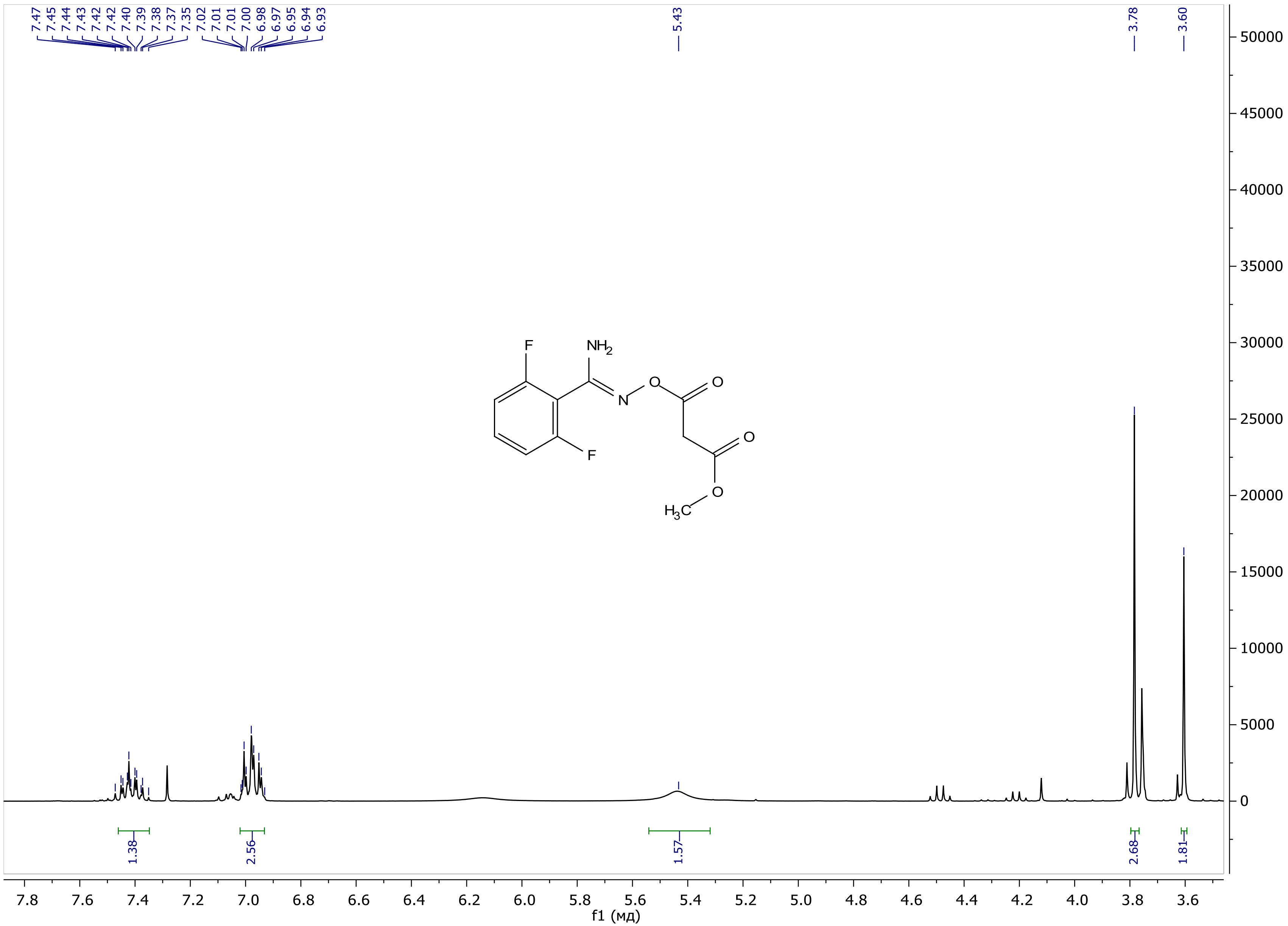

^{13}C NMR spectrum of compound **7a**

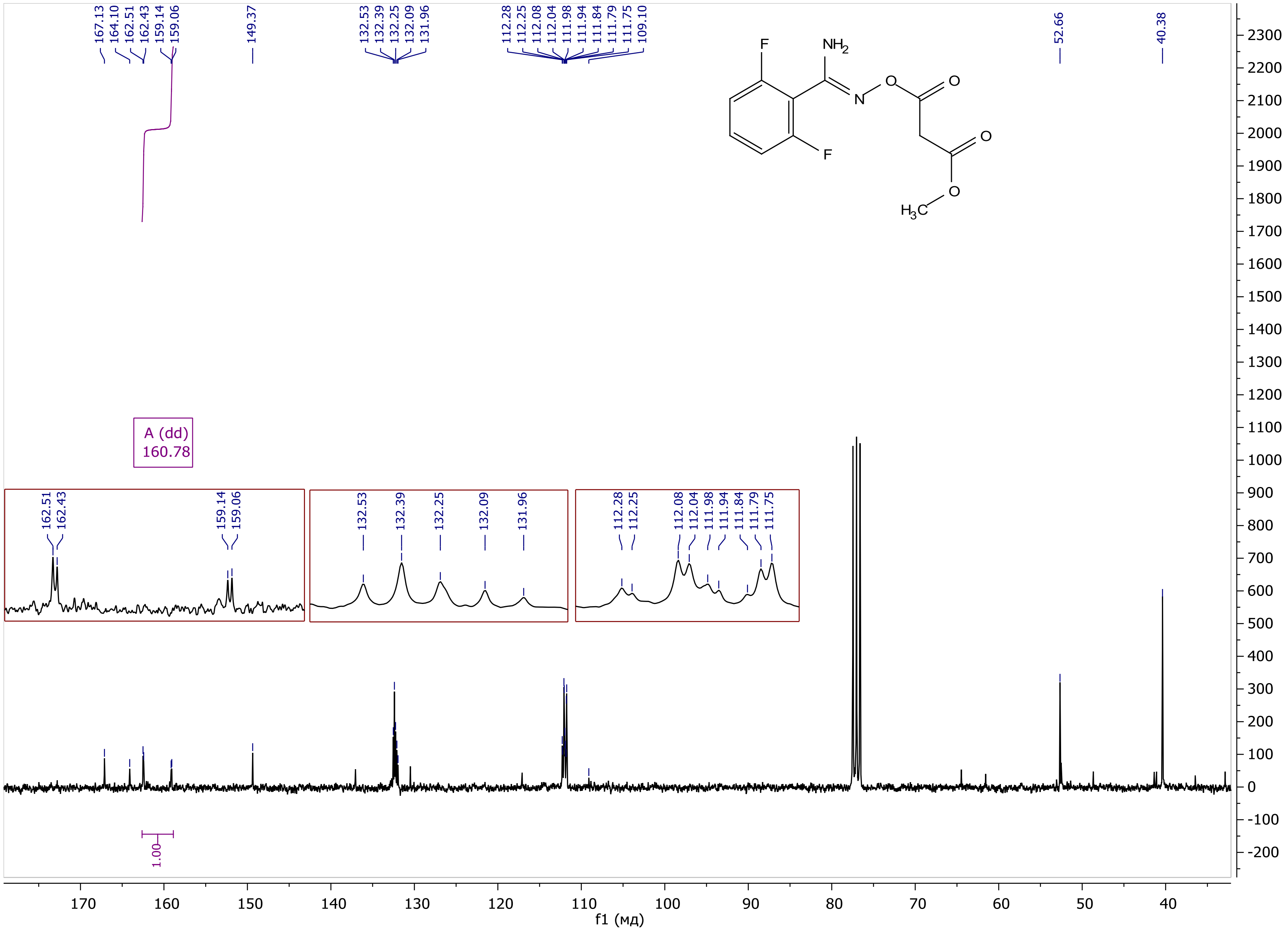

^{1}H NMR spectrum of compound **7b**

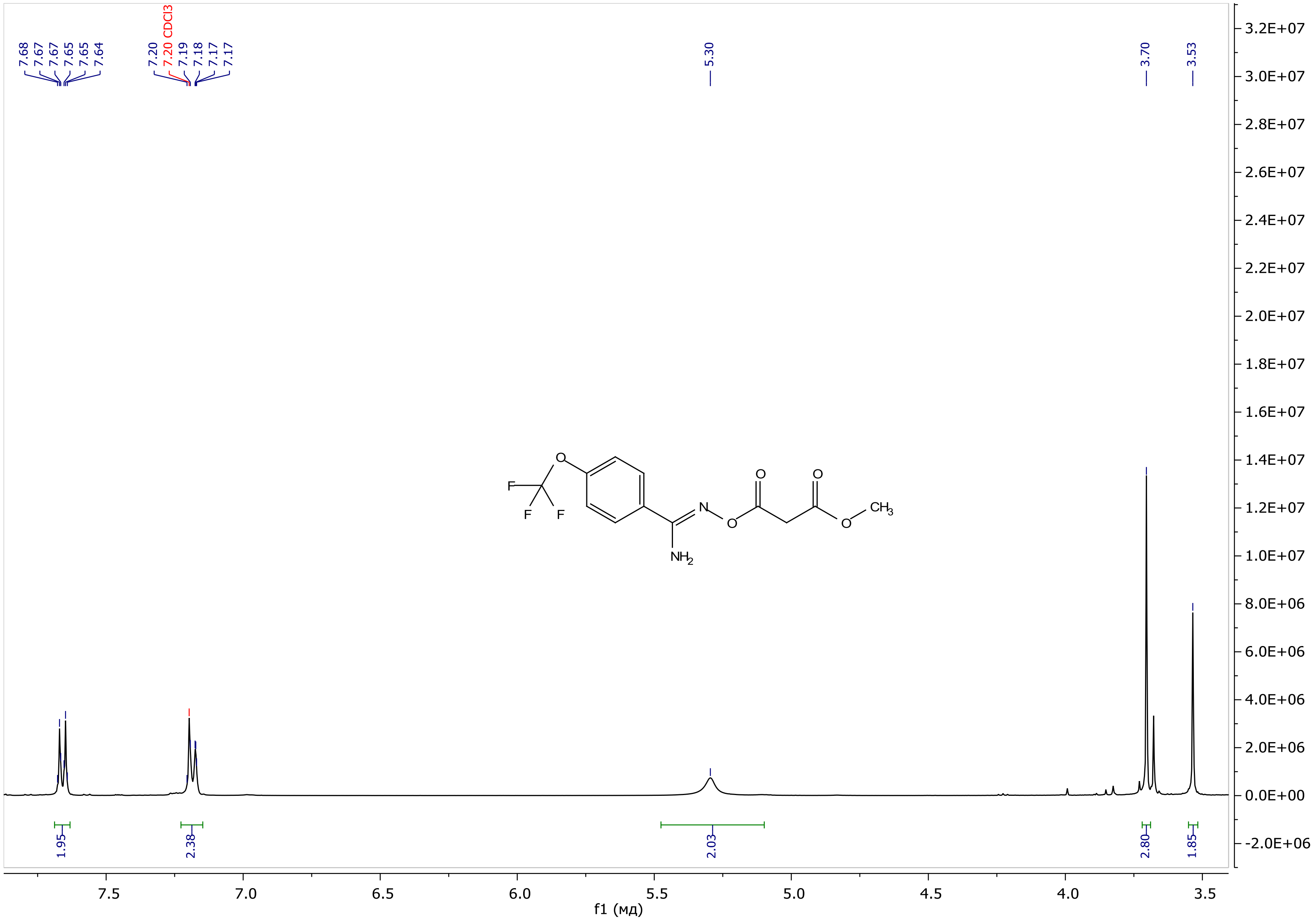

^{13}C NMR spectrum of compound **7b**

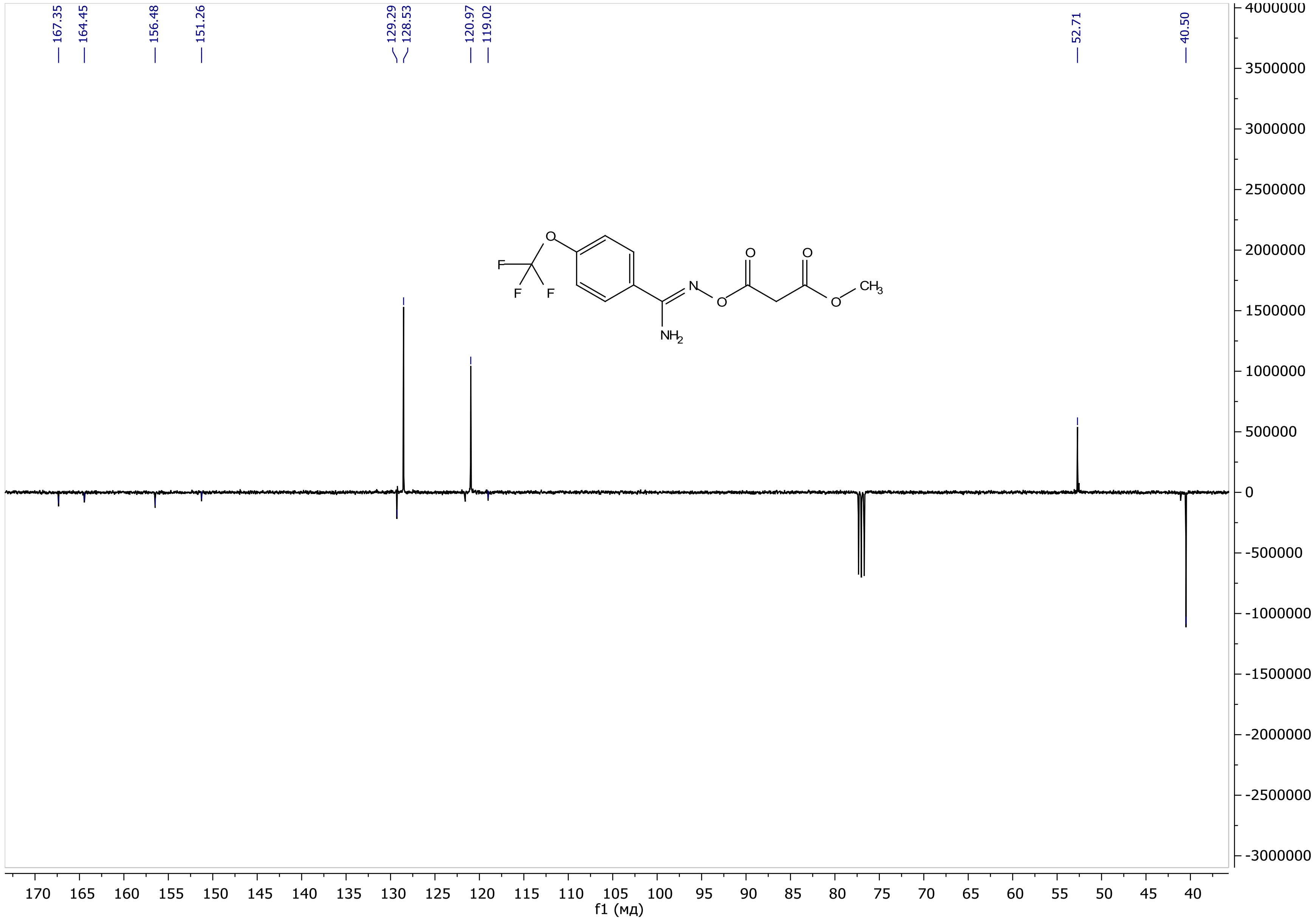

^{1}H NMR spectrum of compound **7c**

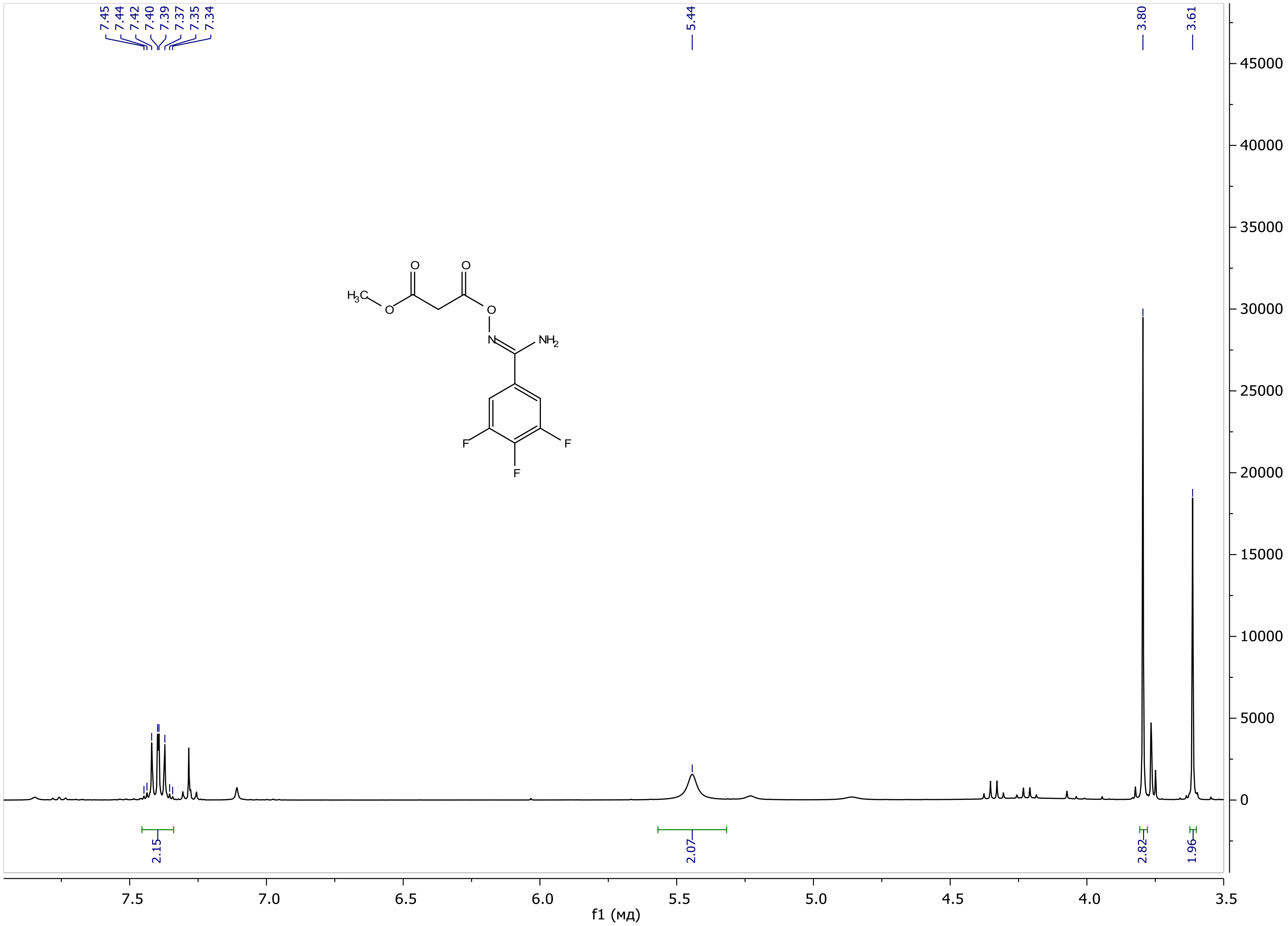

^{13}C NMR spectrum of compound **7c**

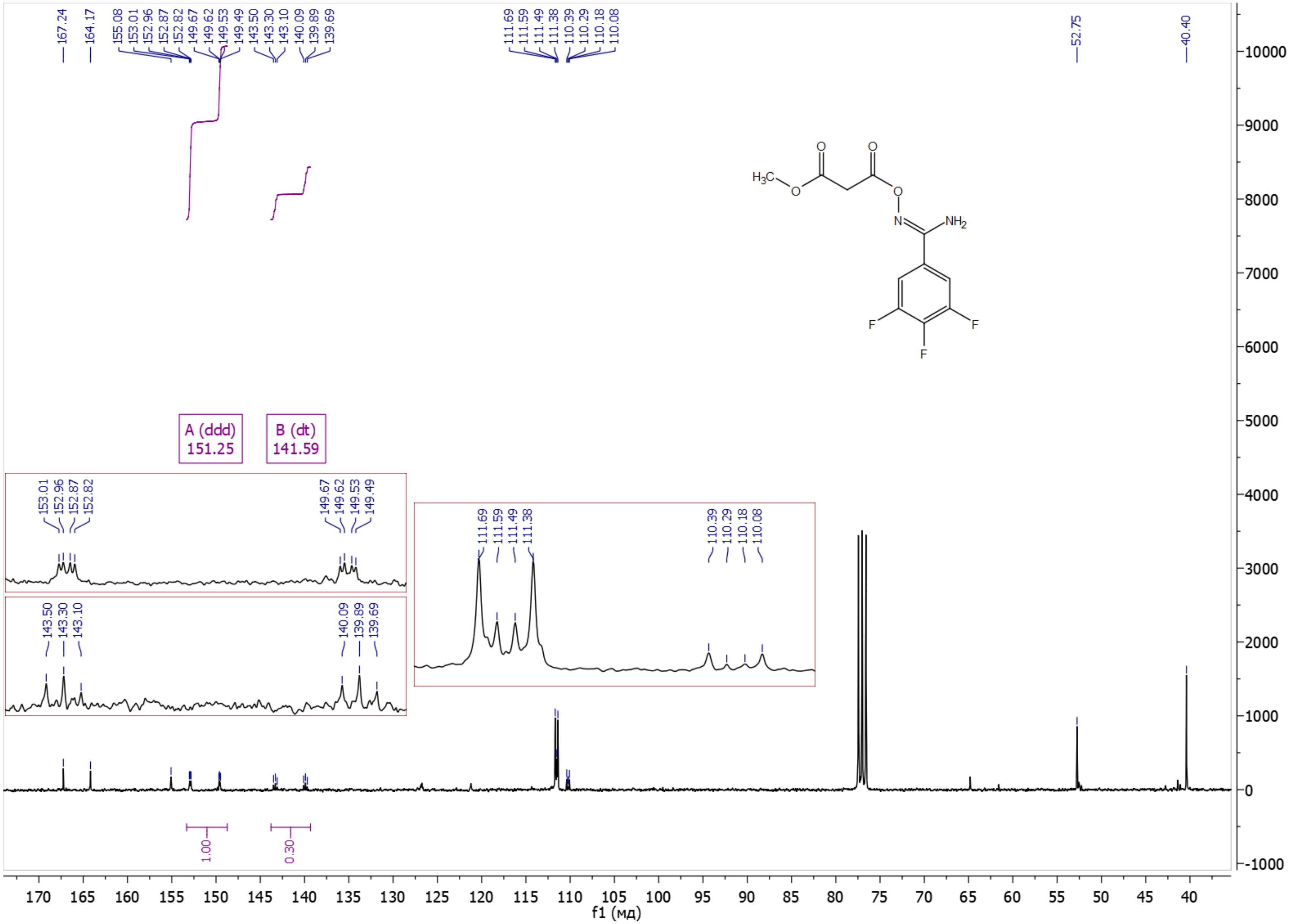

^{1}H NMR spectrum of compound **7d**

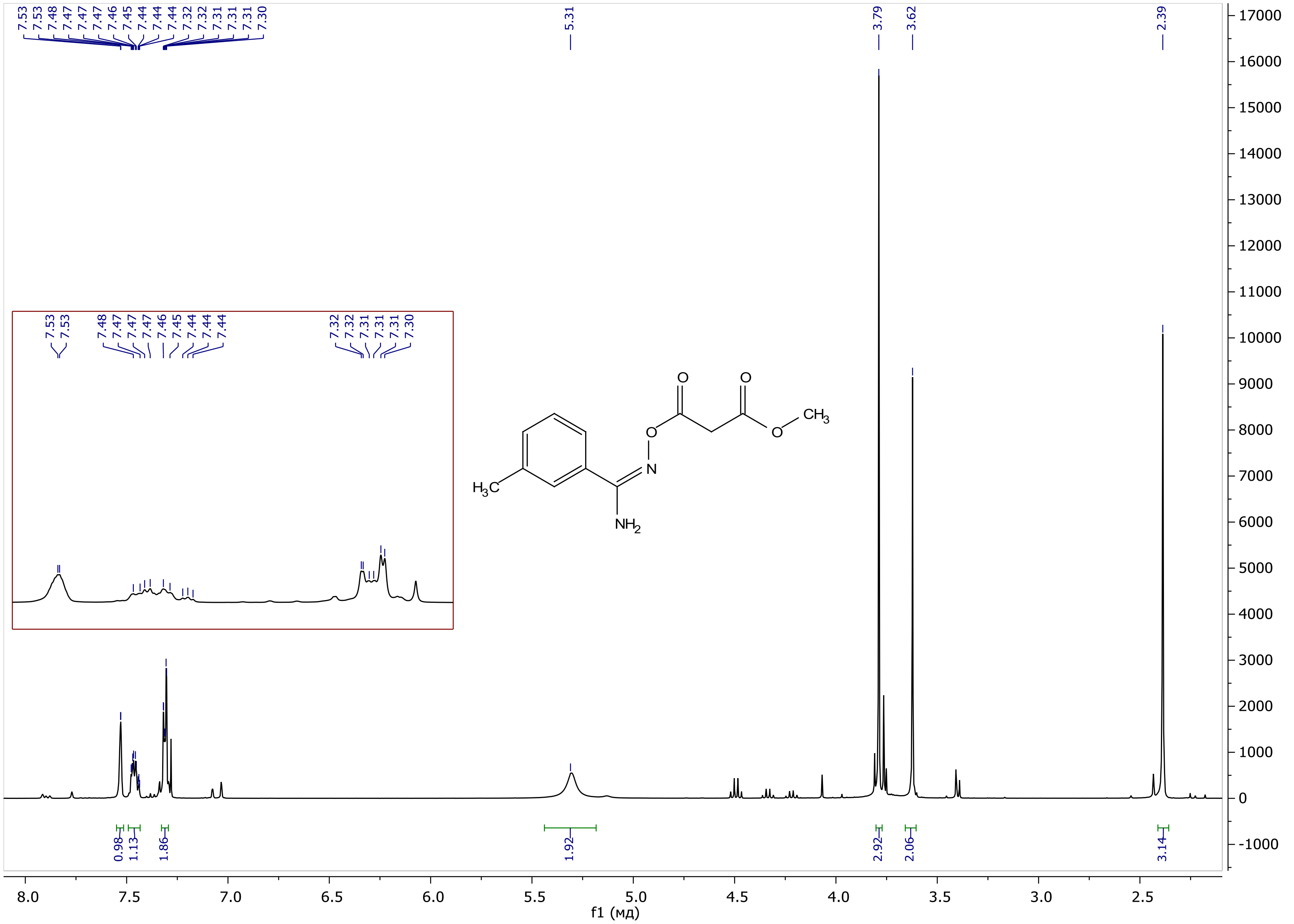

^{13}C NMR spectrum of compound **7d**

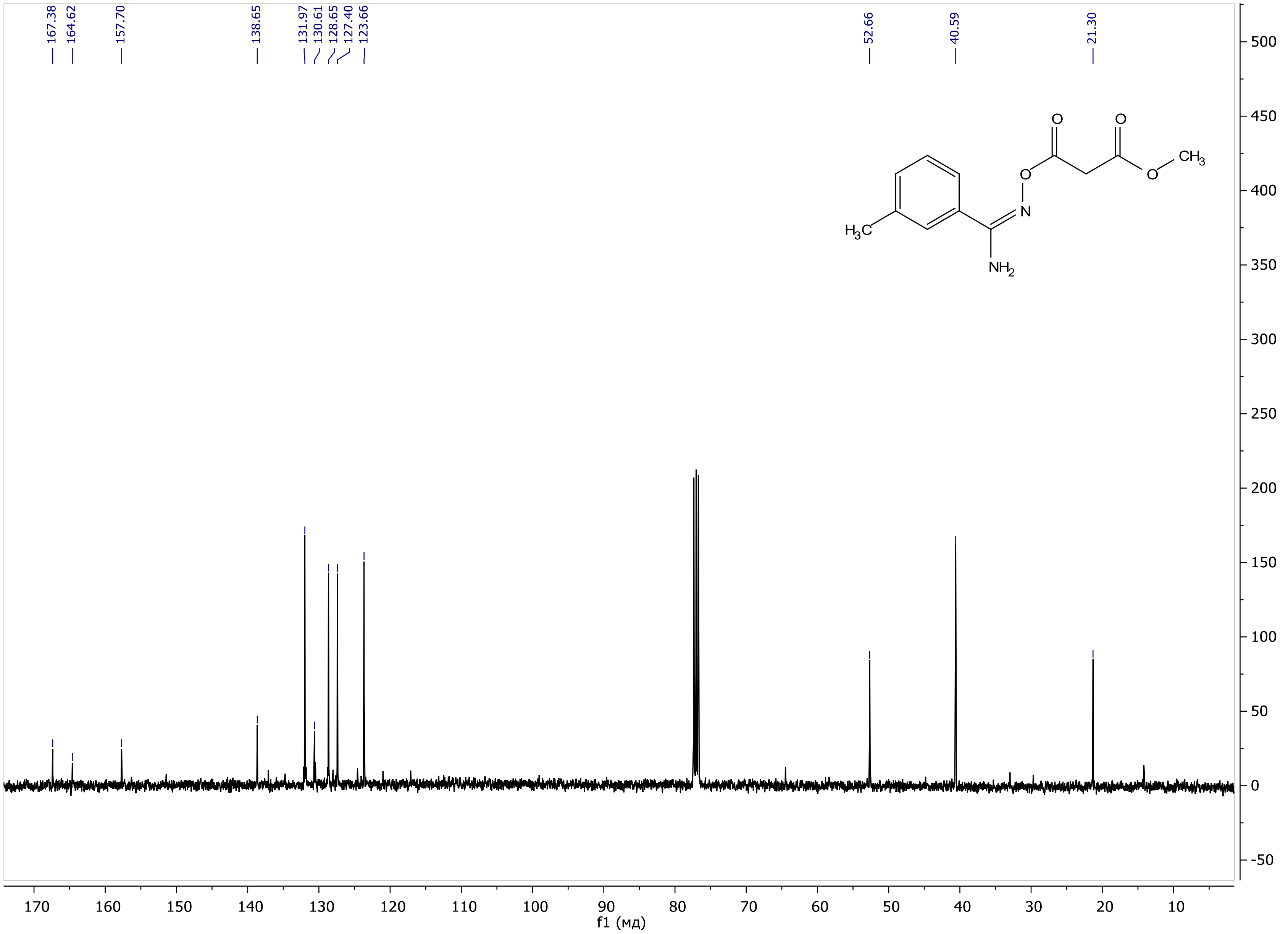

^{1}H NMR spectrum of compound **11a**

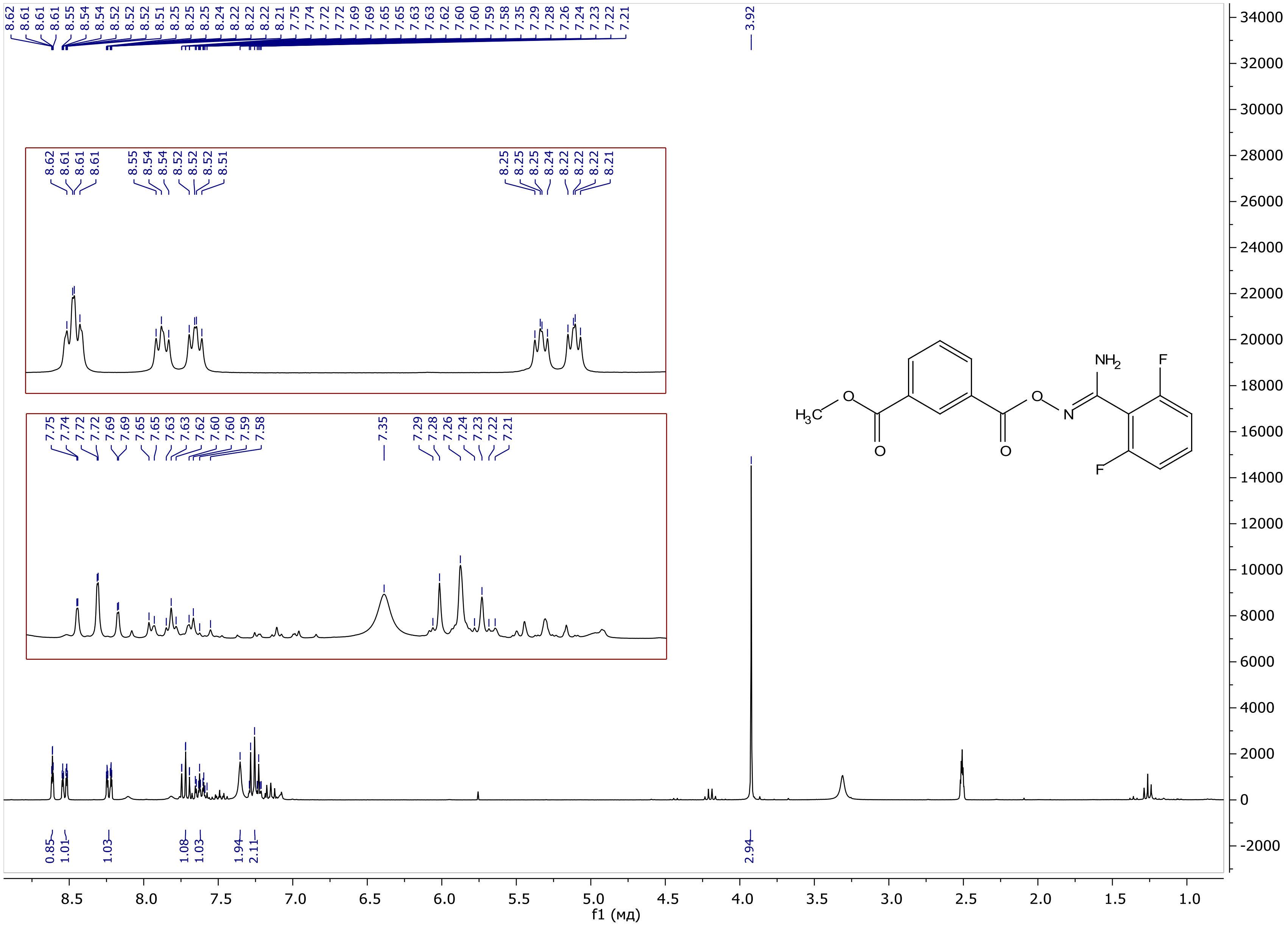

^{13}C NMR spectrum of compound **11a**

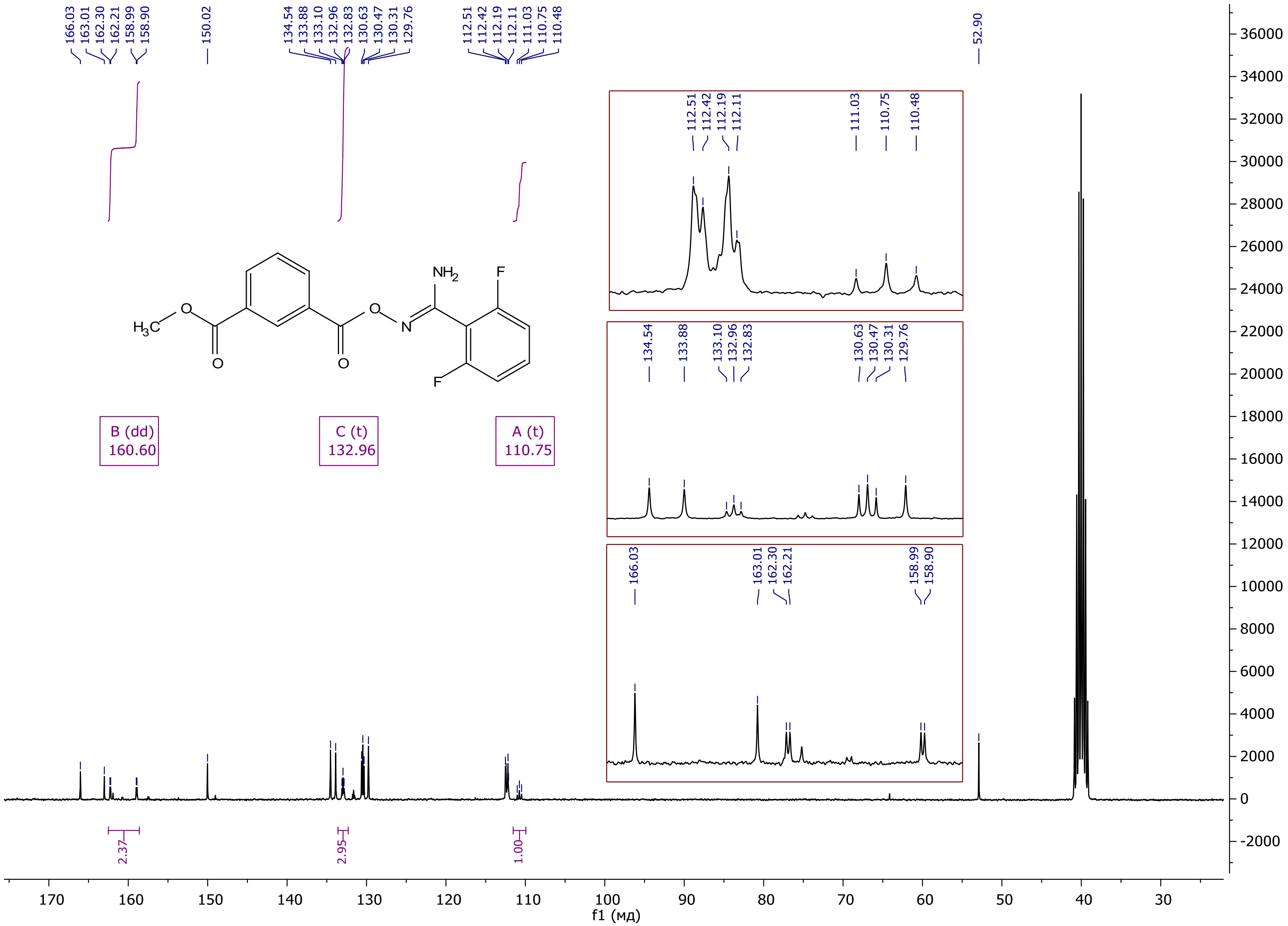

^{1}H NMR spectrum of compound **11b**

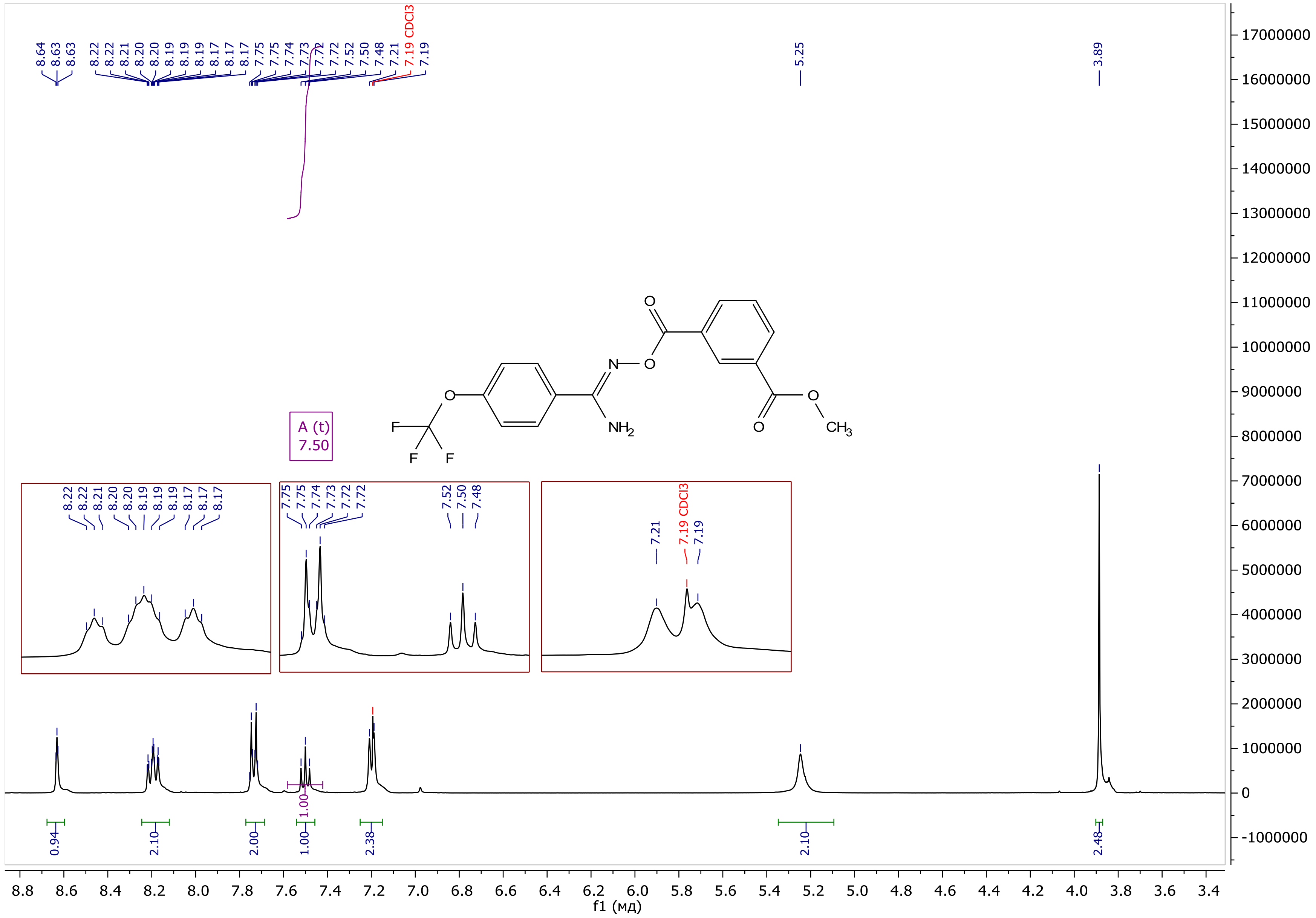

^{13}C NMR spectrum of compound **11b**

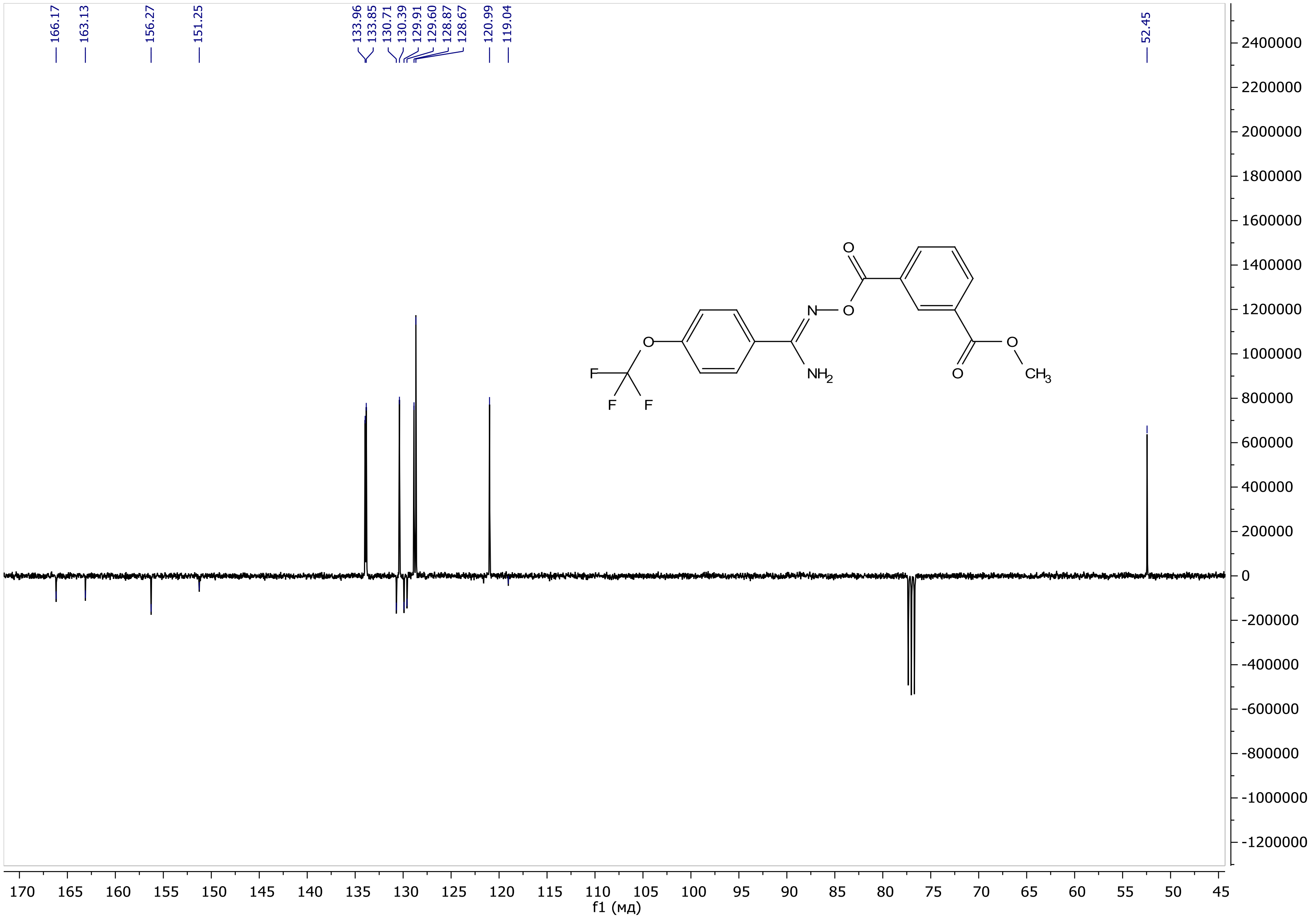

^{1}H NMR spectrum of compound **11c**

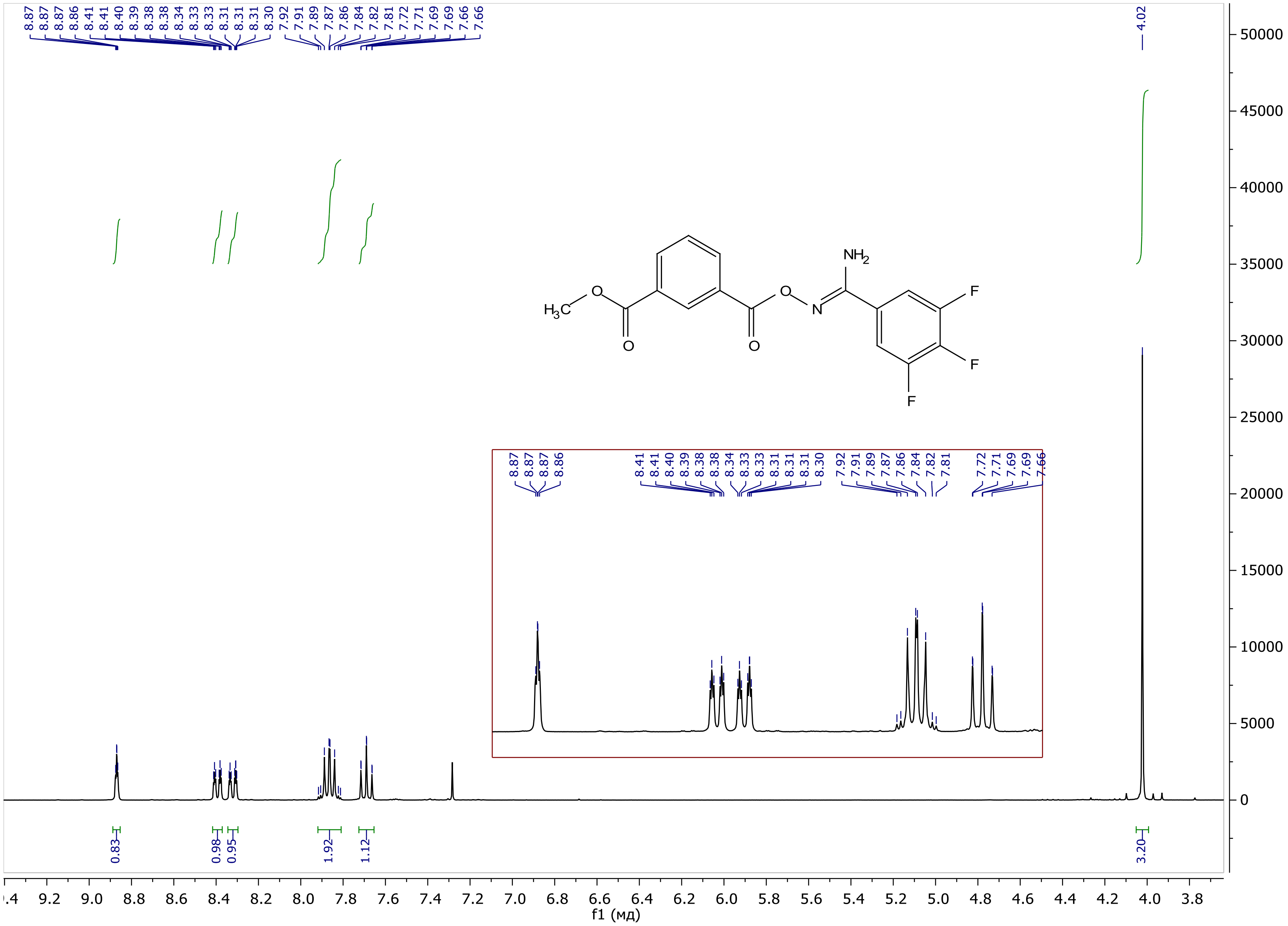

^{13}C NMR spectrum of compound **11c**

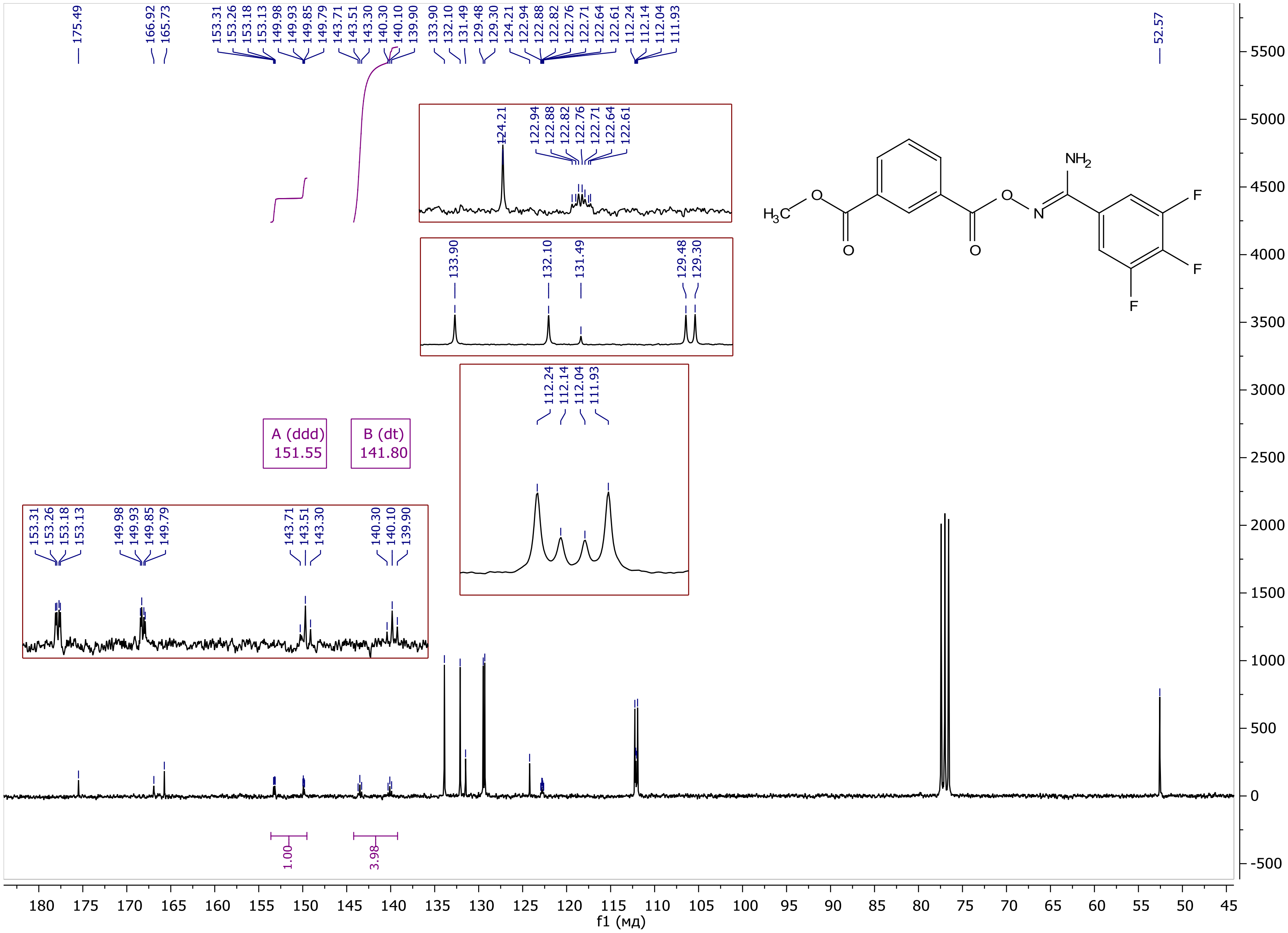

^{1}H NMR spectrum of compound **21a**

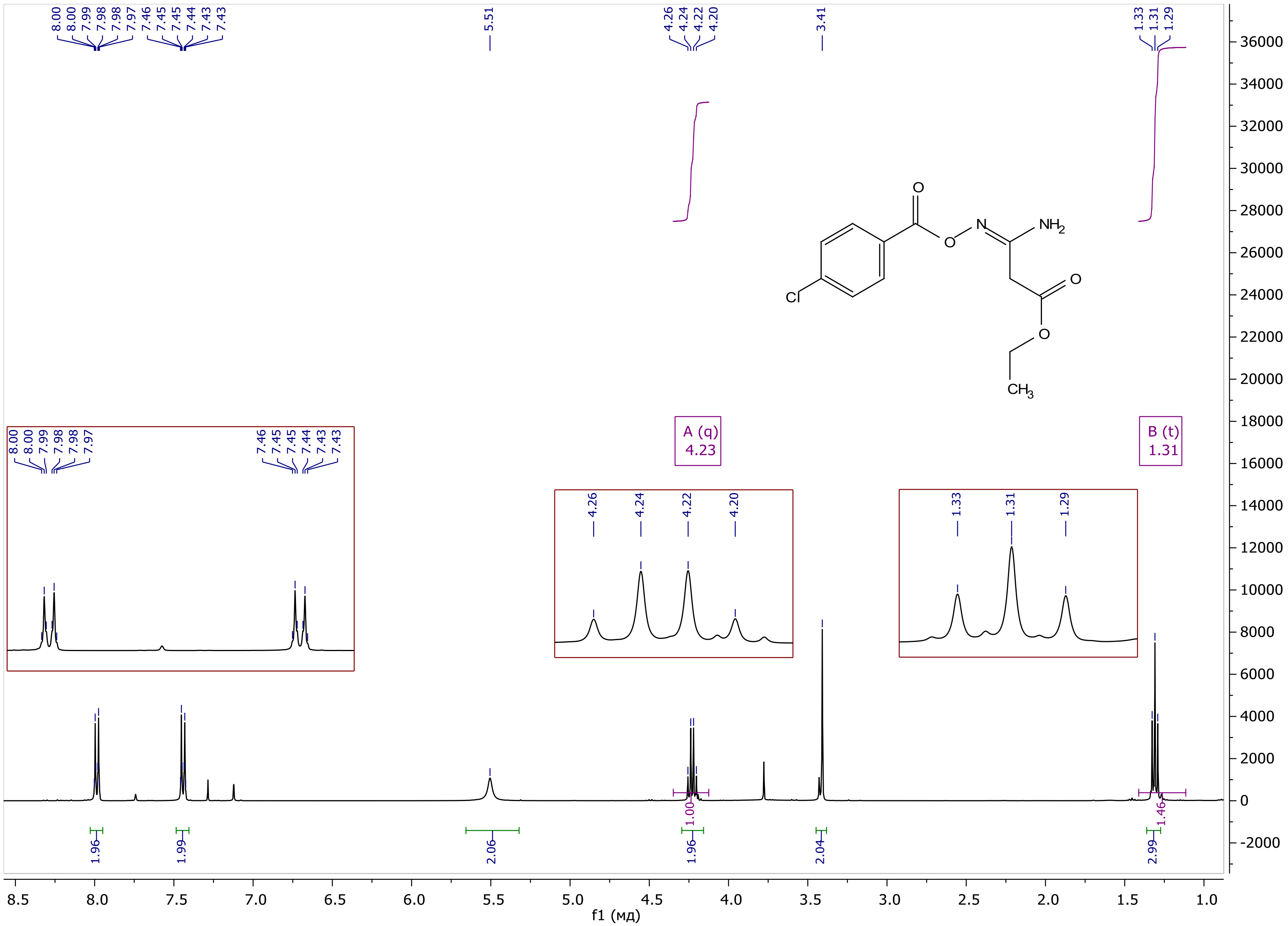

^{13}C NMR spectrum of compound **21a**

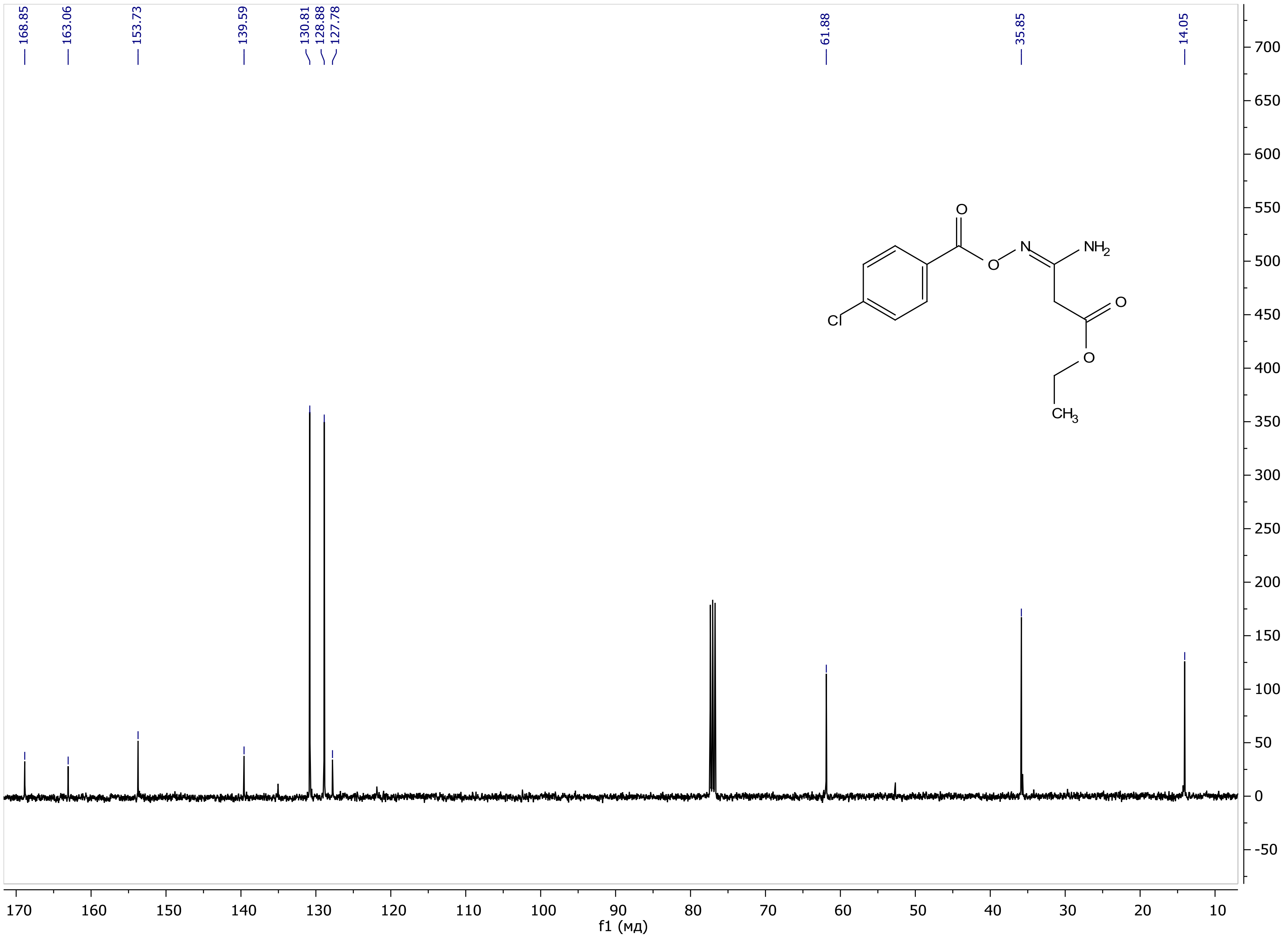

^{1}H NMR spectrum of compound **21b**

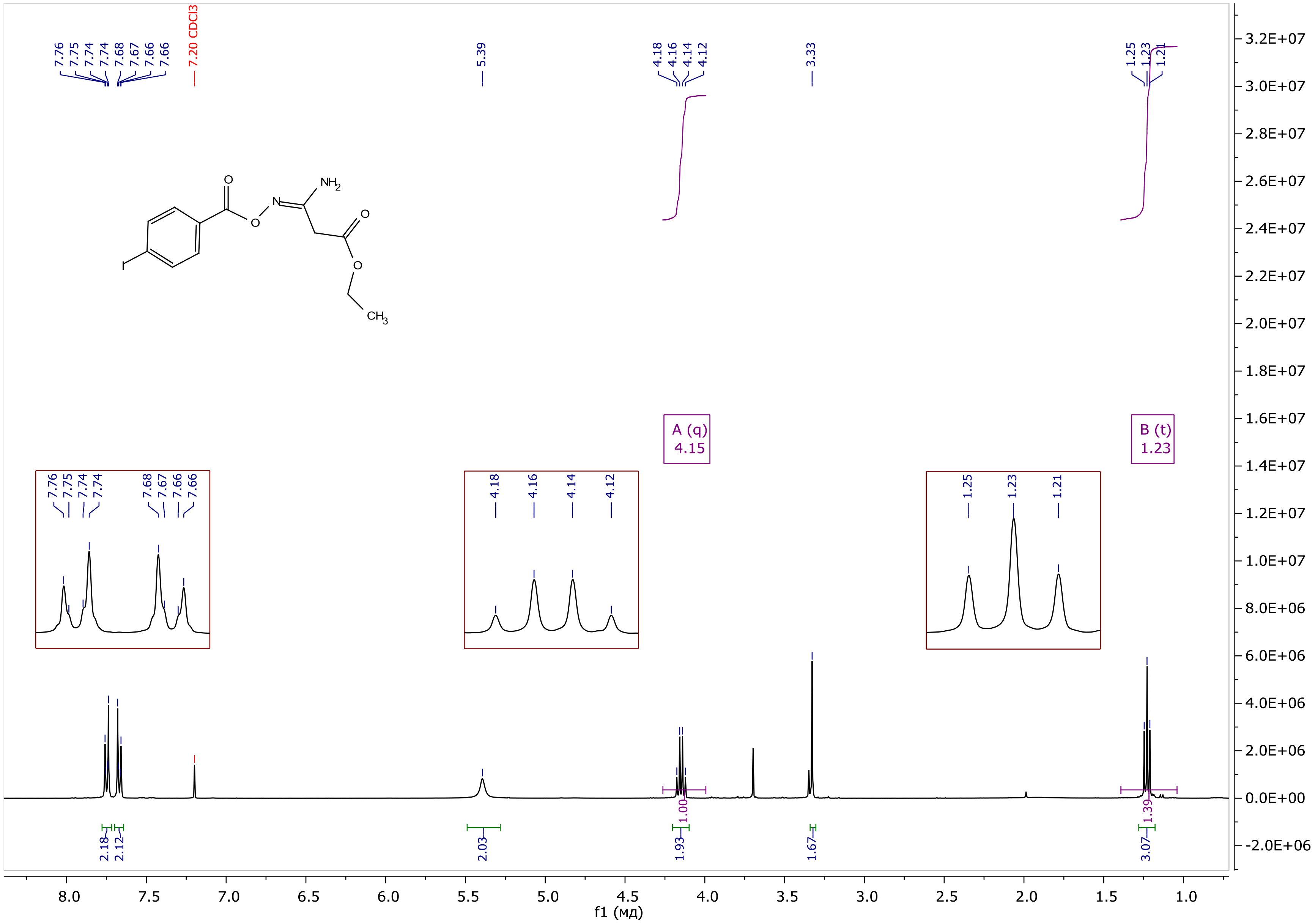

^{13}C NMR spectrum of compound **21b**

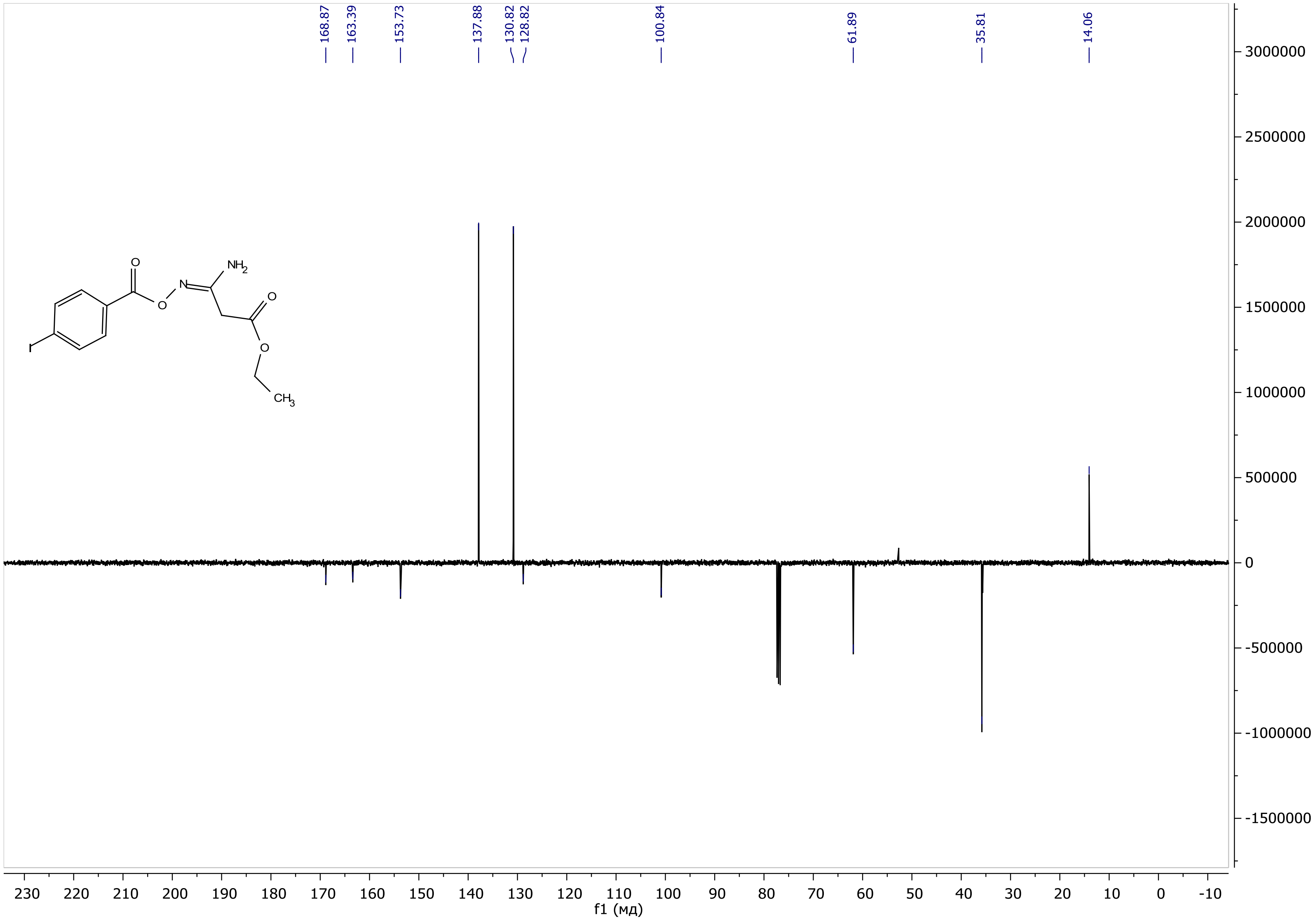

^{1}H NMR spectrum of compound **21c**

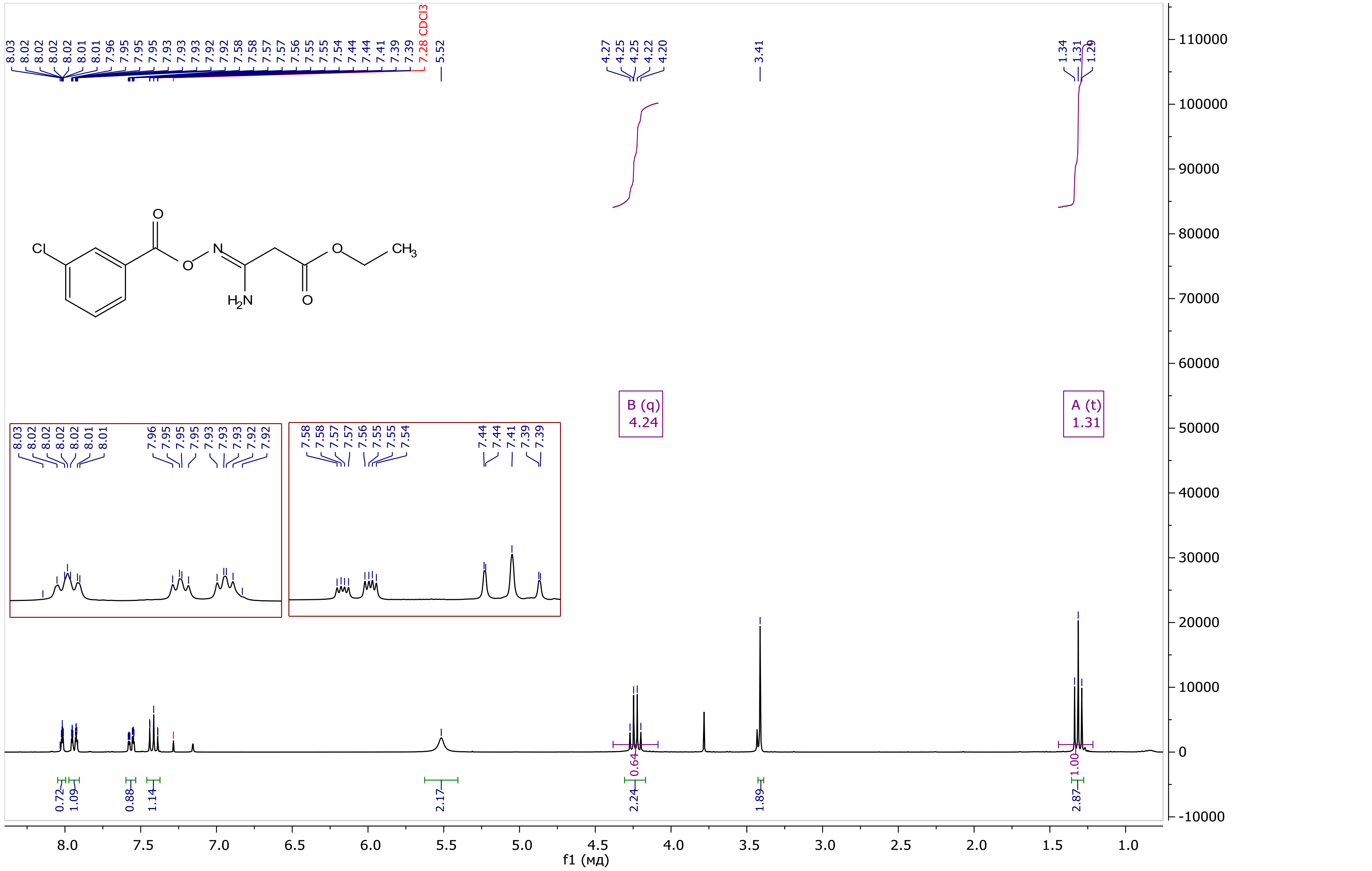

^{13}C NMR spectrum of compound **21c**

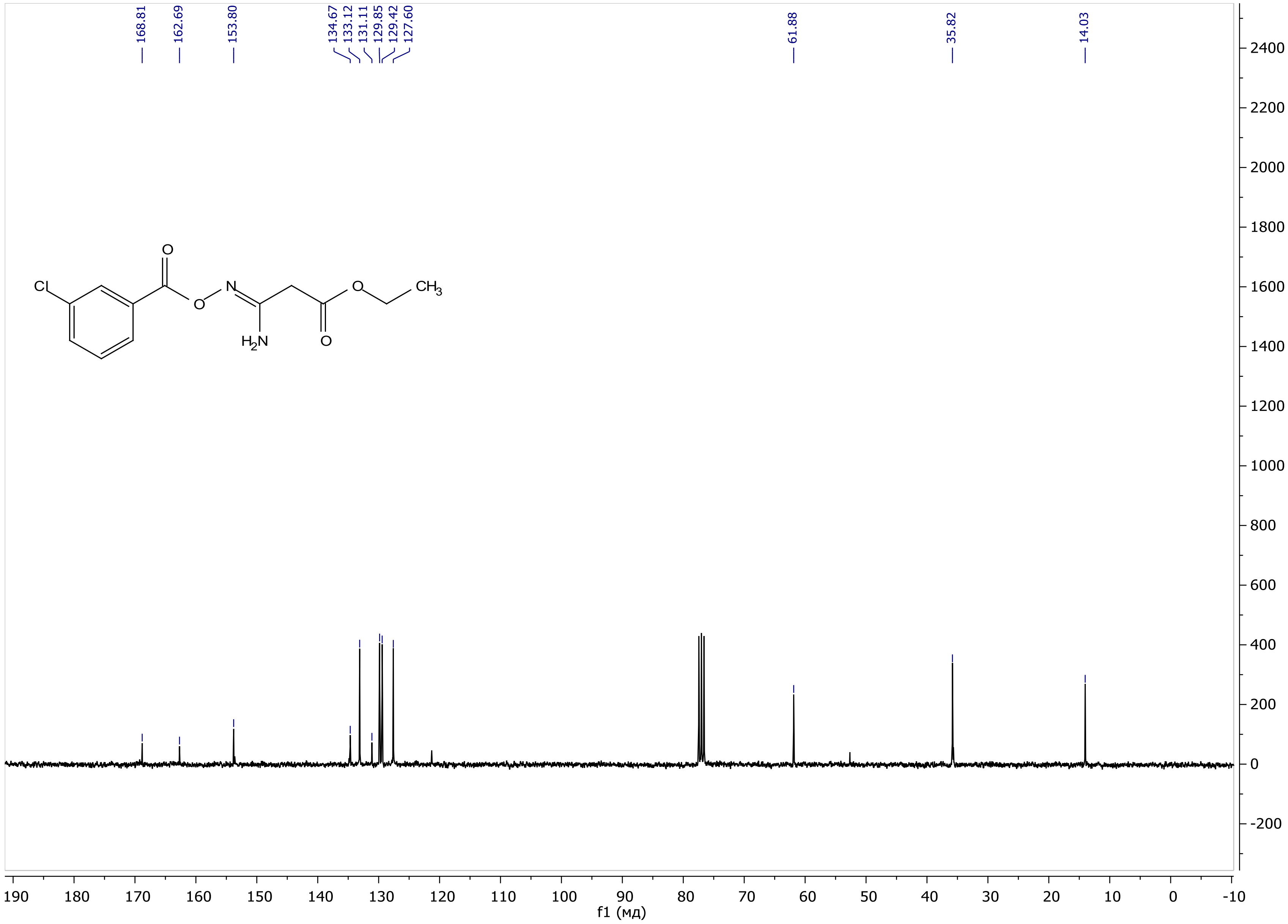

^{1}H NMR spectrum of compound **21d**

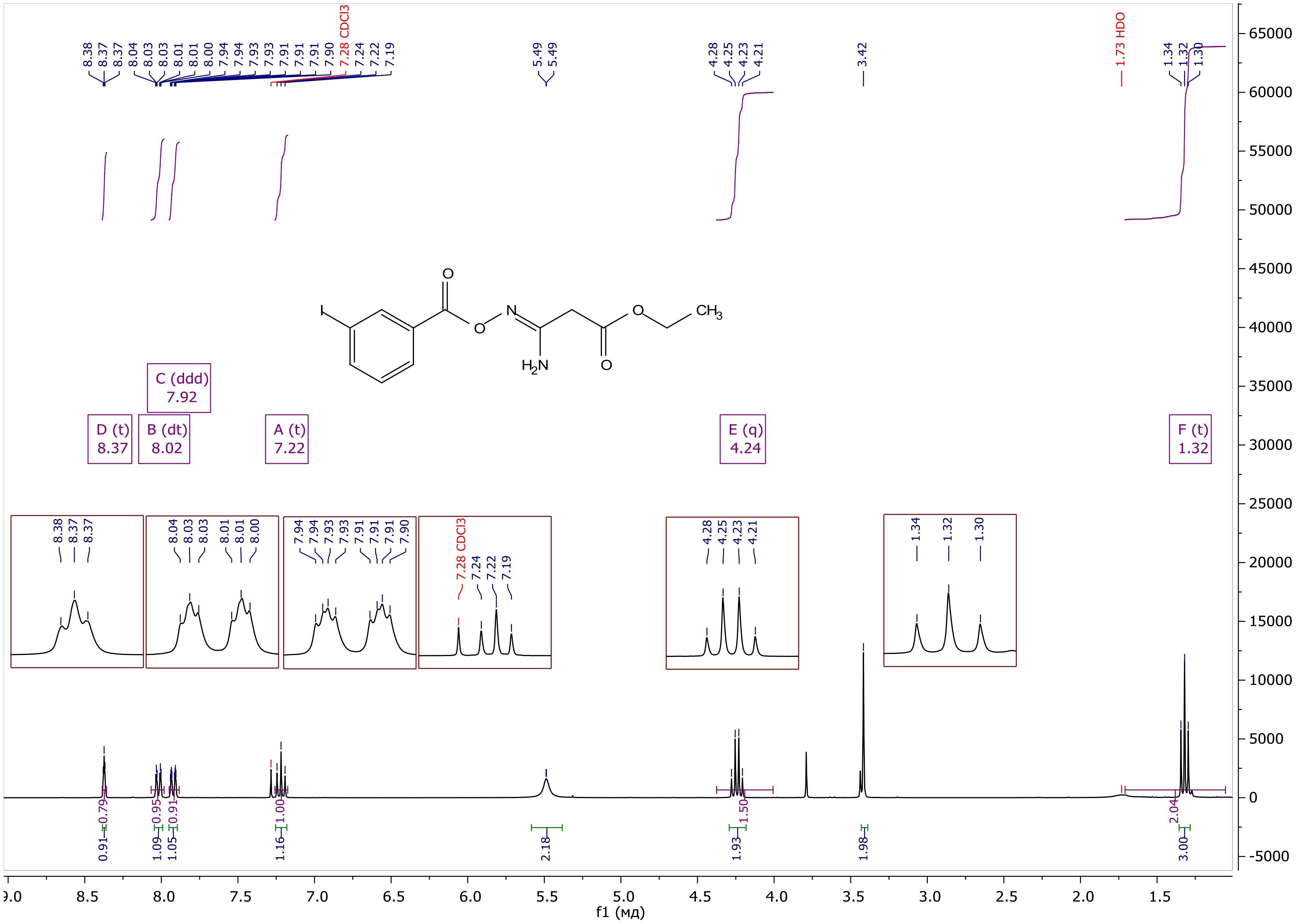

^{13}C NMR spectrum of compound **21d**

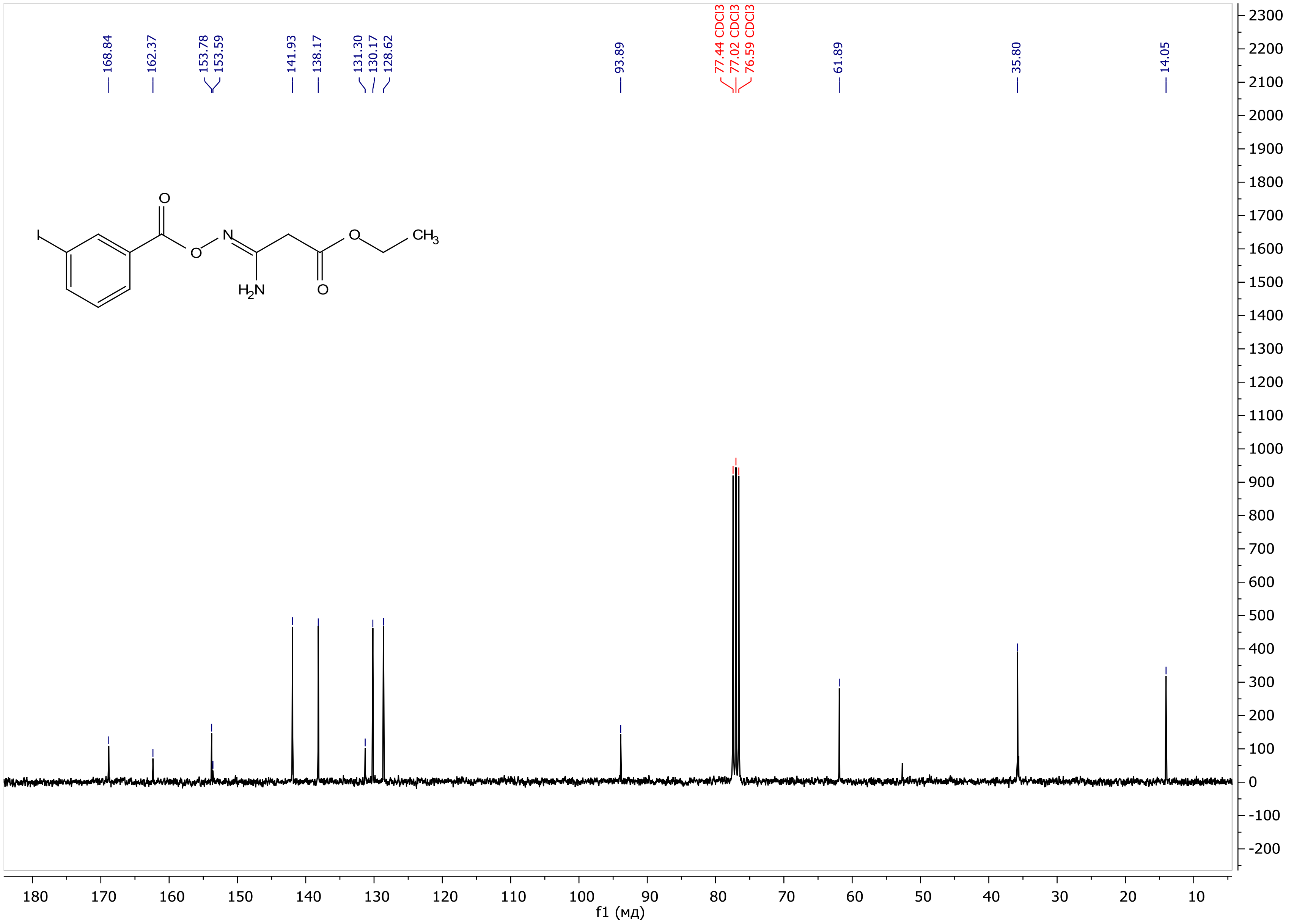

^{1}H, ^{13}C NMR spectra for ethyl 3-aryl 1,2,4-oxadiazole-5-carboxylates synthesized

^{1}H NMR spectrum of compound **3a**

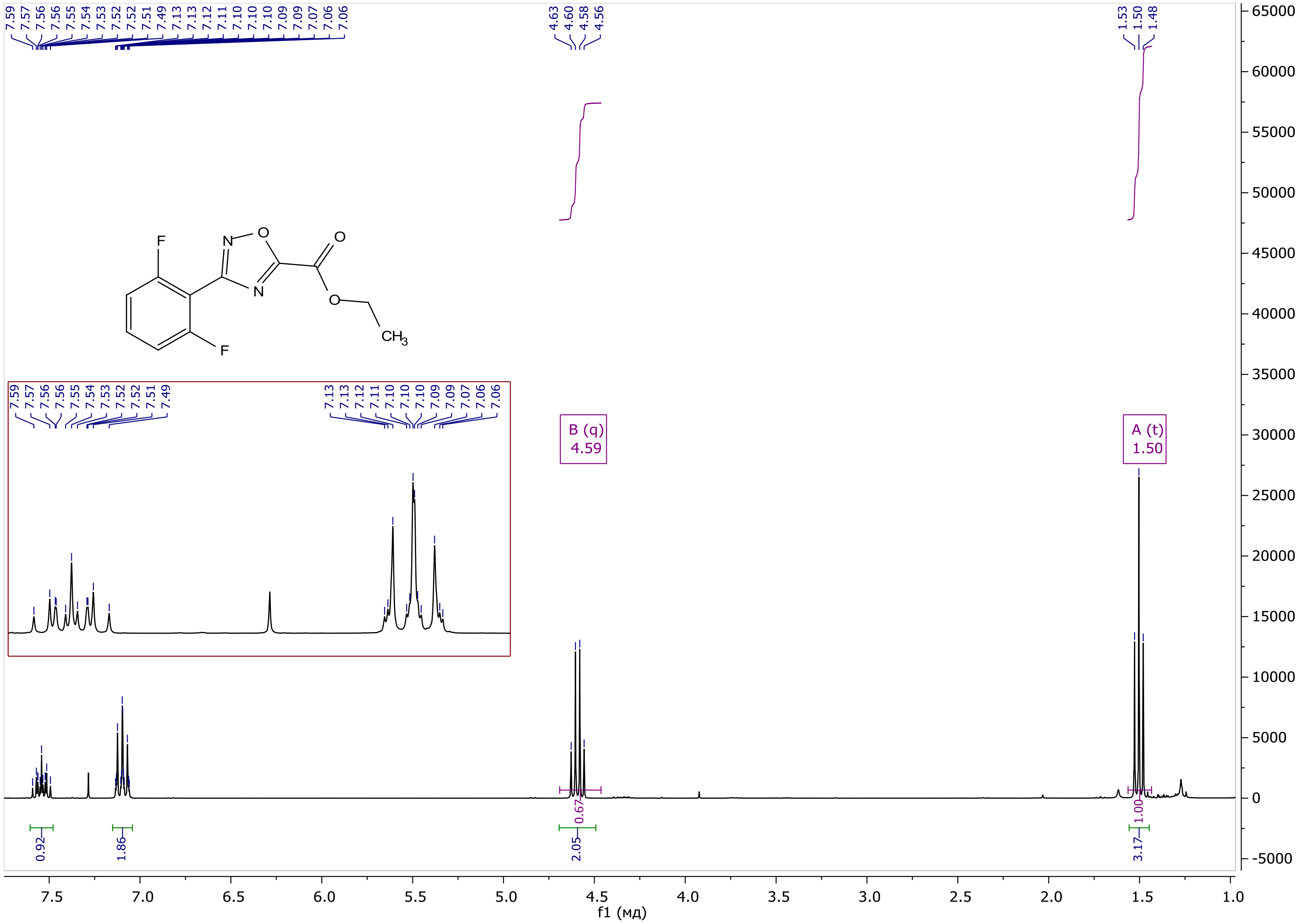

^{13}C NMR spectrum of compound **3a**

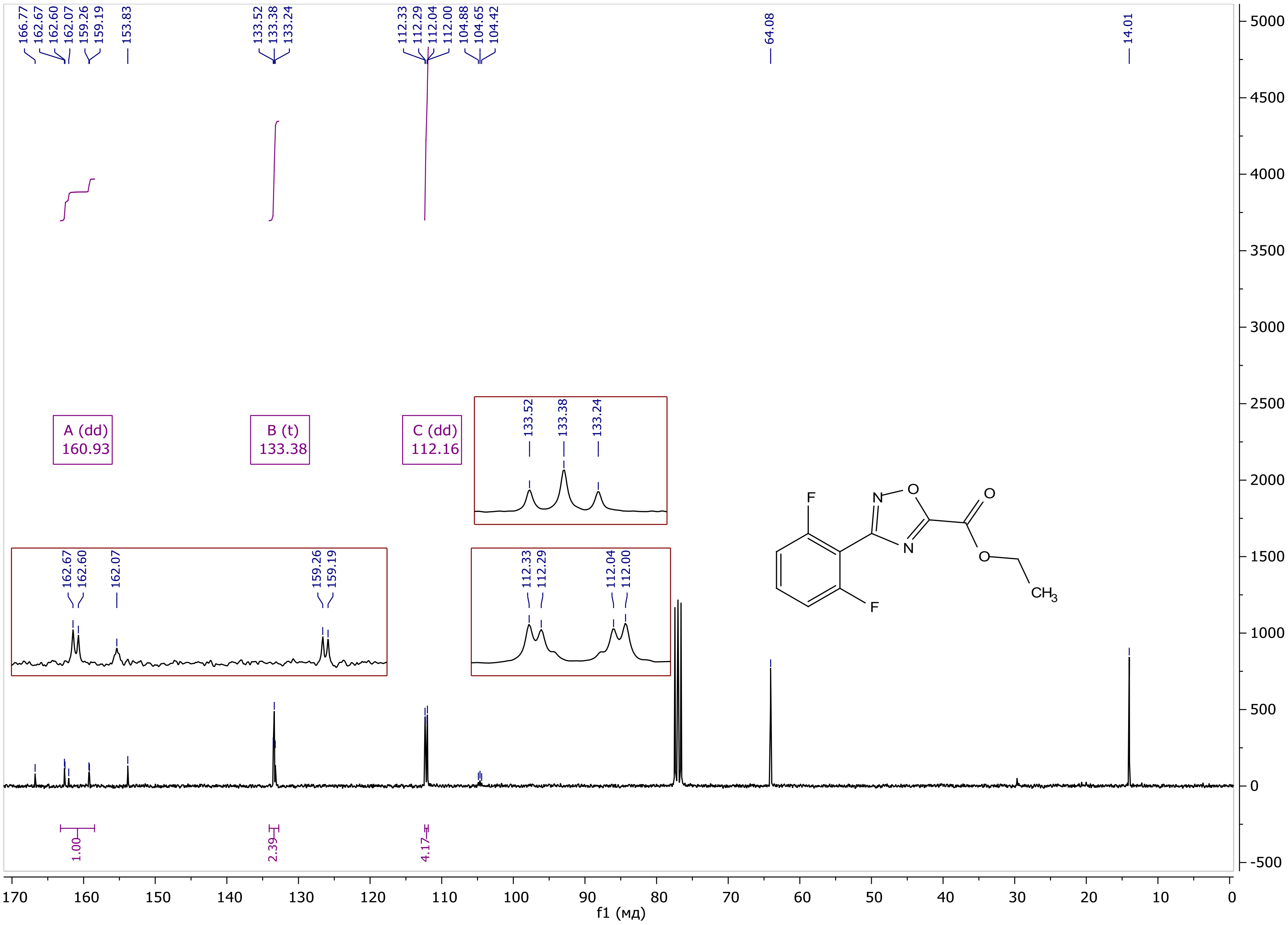

^{1}H NMR spectrum of compound **3b**

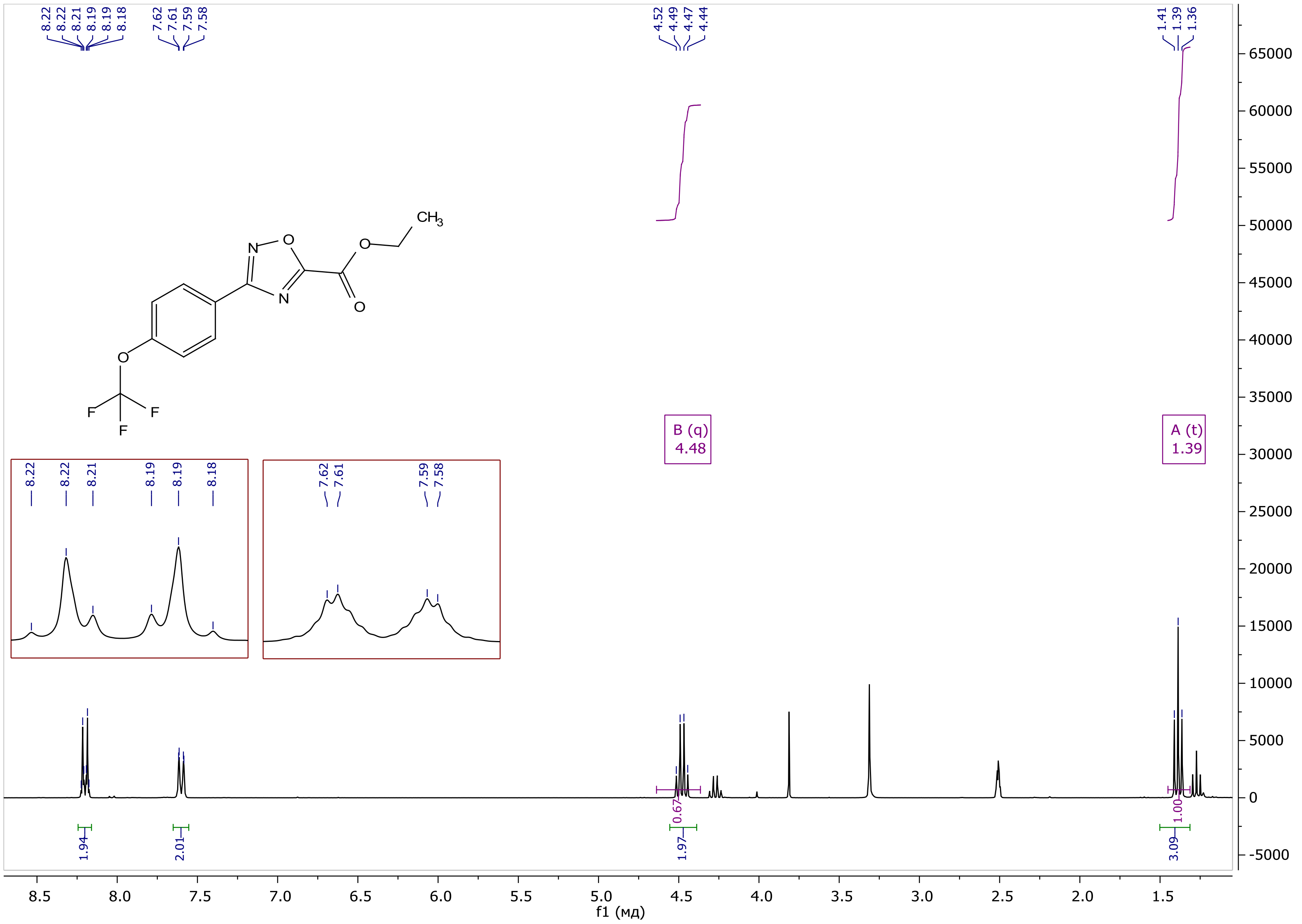

^{13}C NMR spectrum of compound **3b**

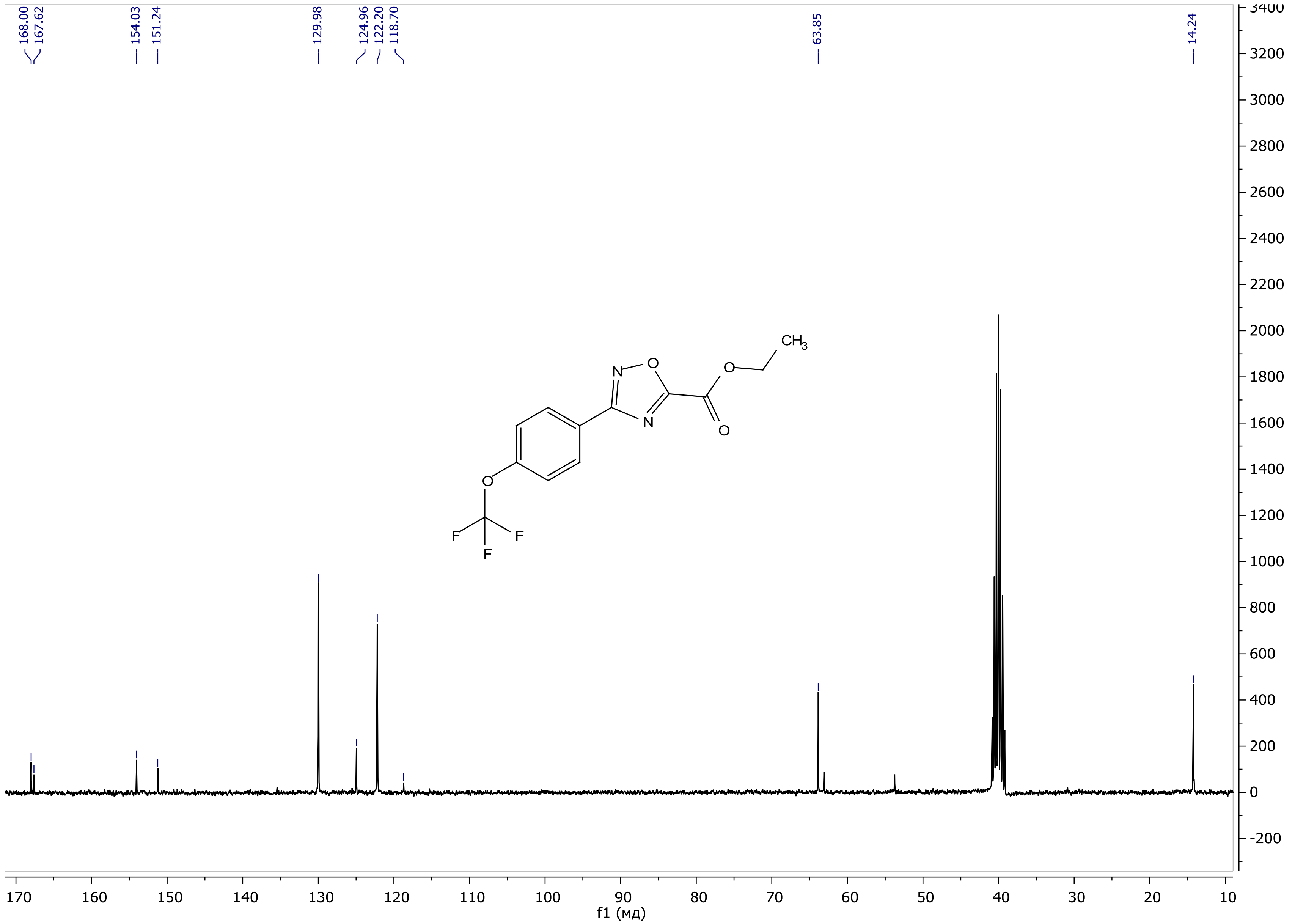

^{1}H NMR spectrum of compound **3c**

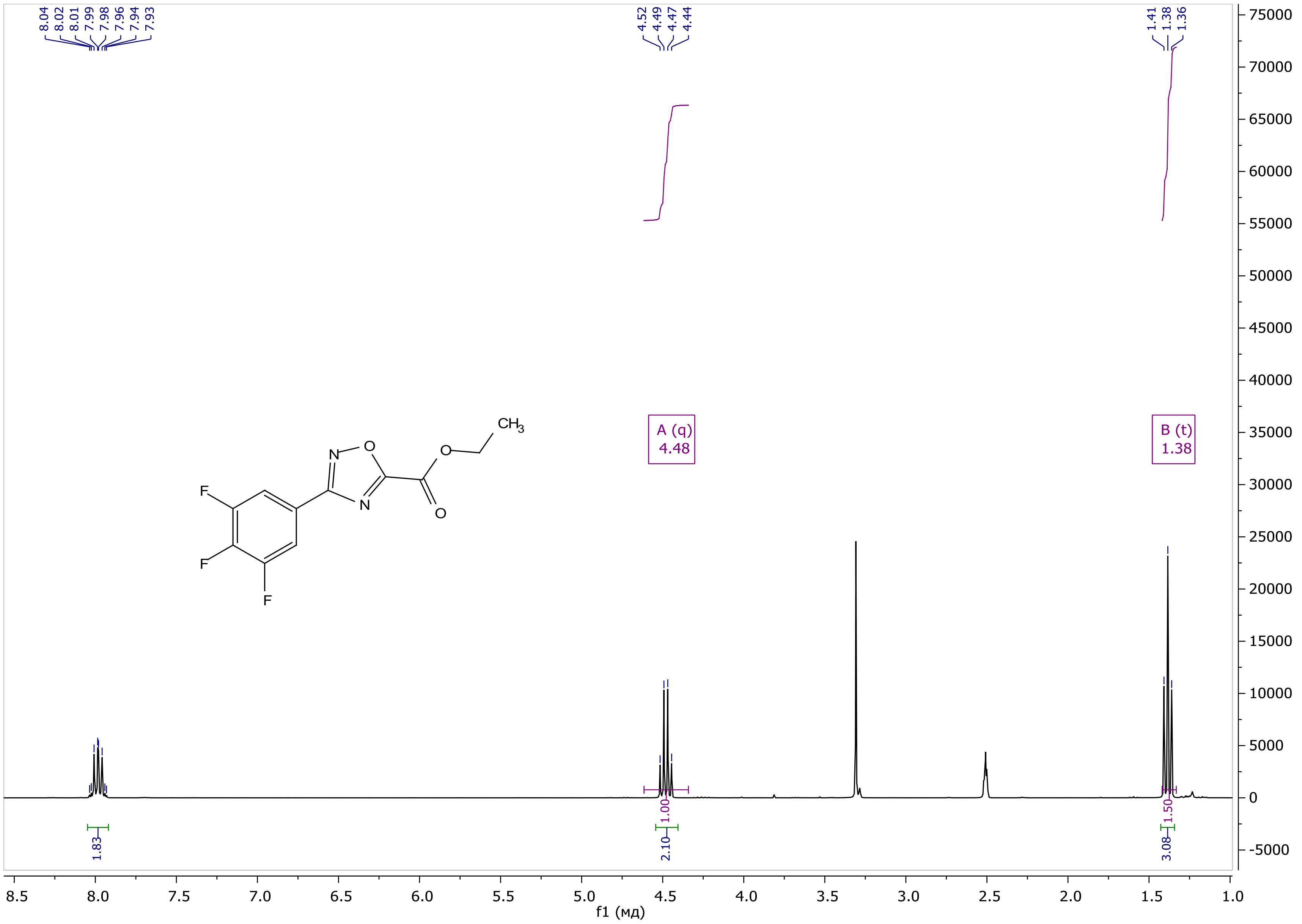

^{13}C NMR spectrum of compound **3c**

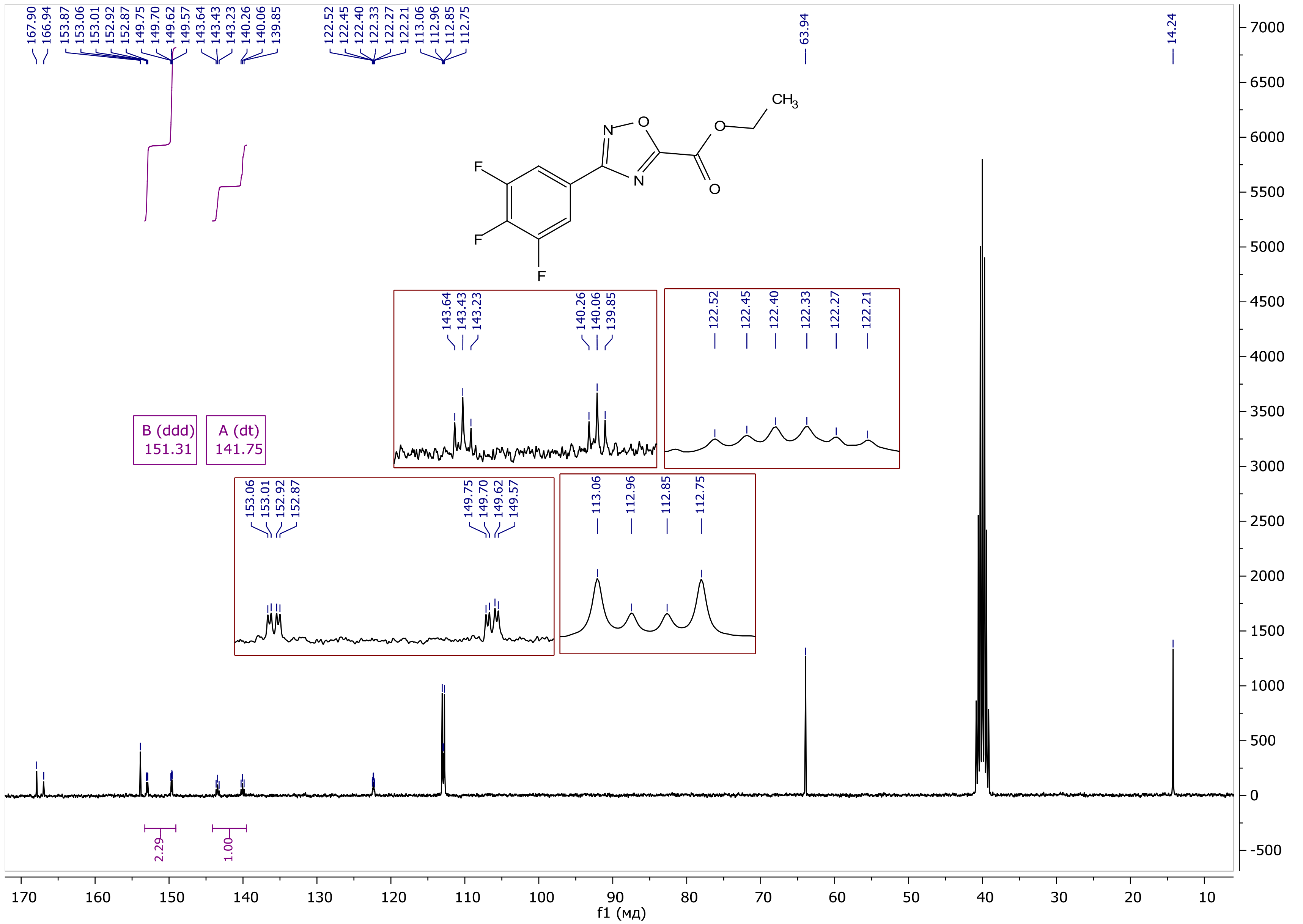

^{1}H NMR spectrum of compound **3d**

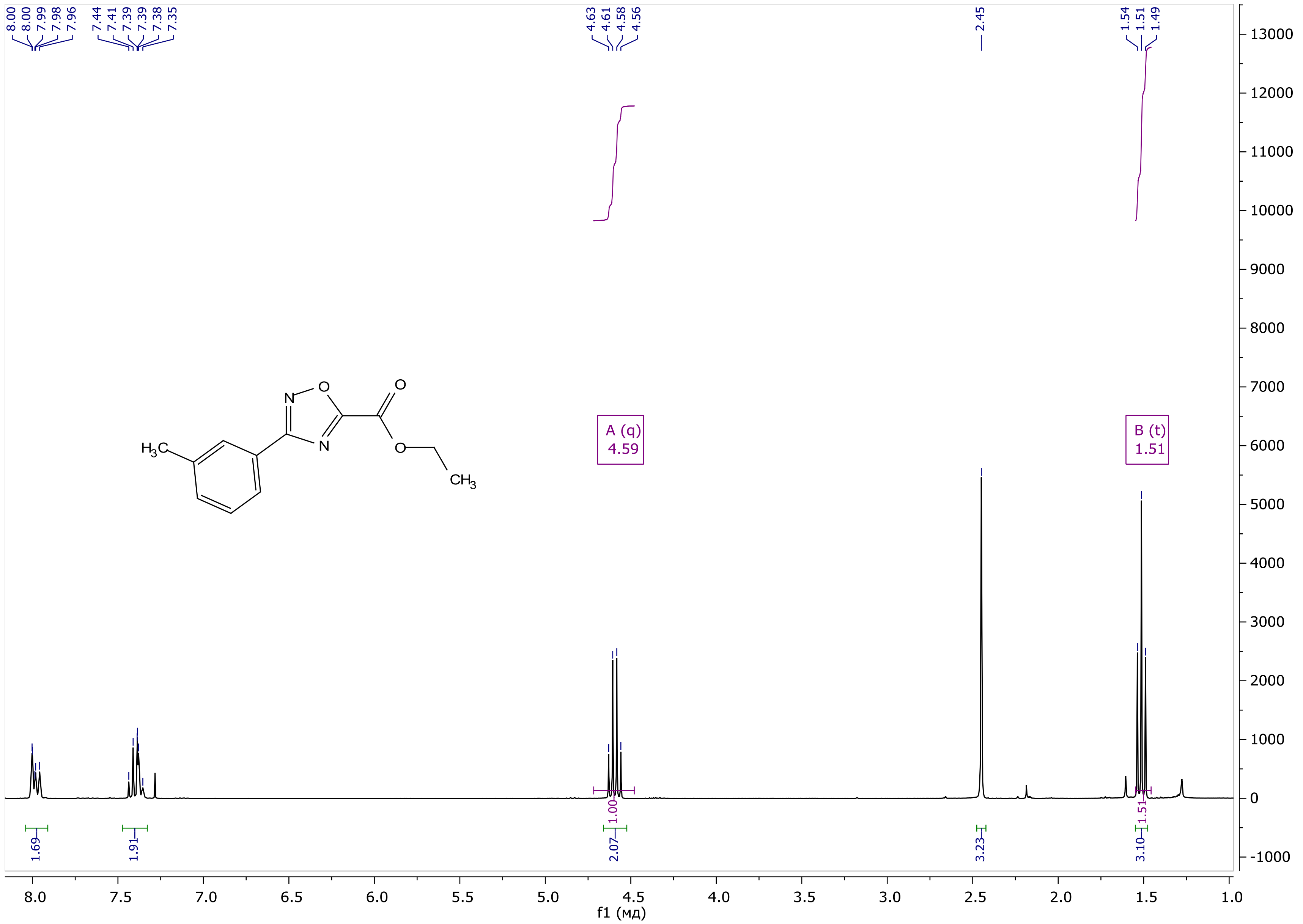

^{13}C NMR spectrum of compound **3d**

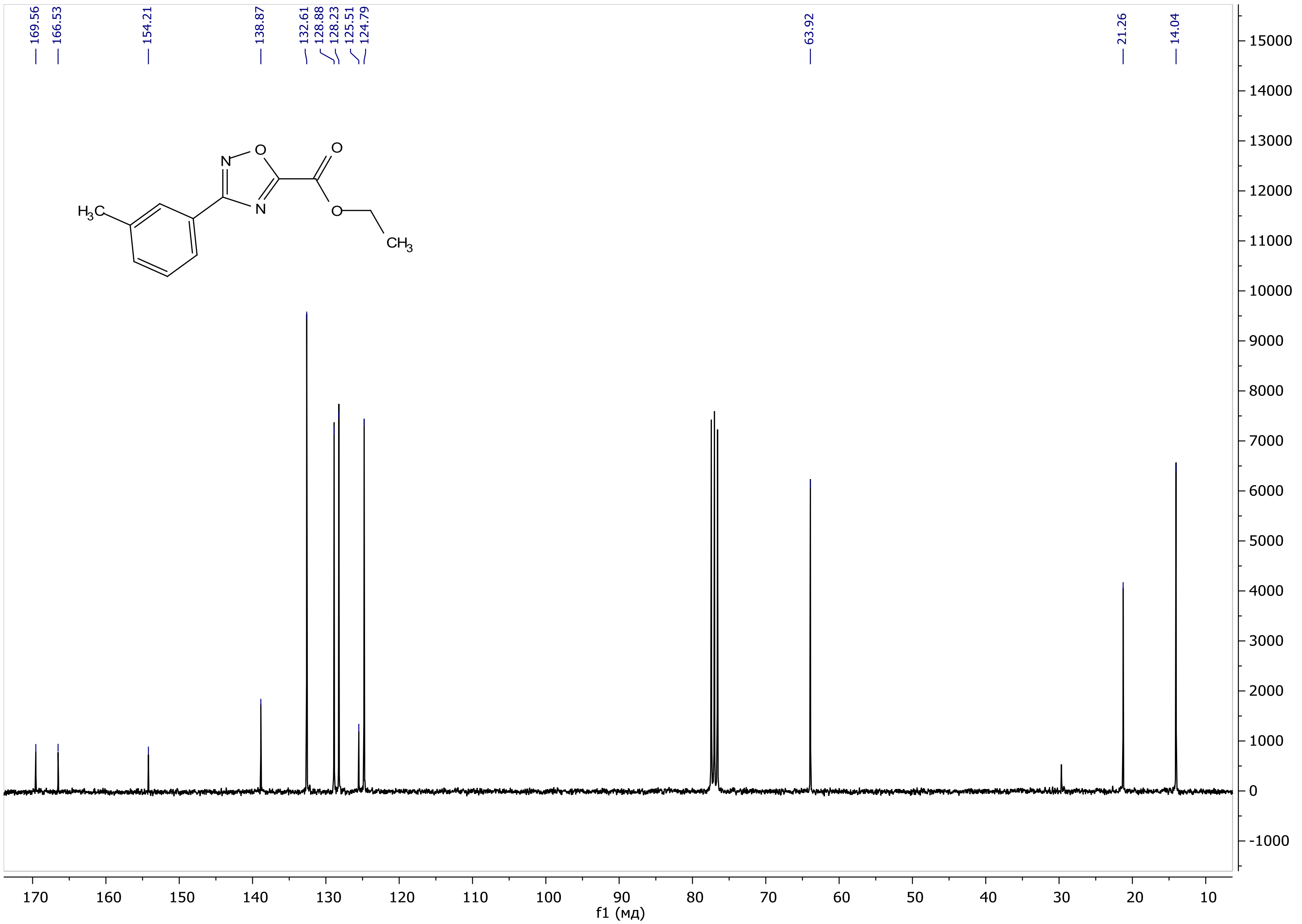

^{1}H NMR spectrum of compound **3e**

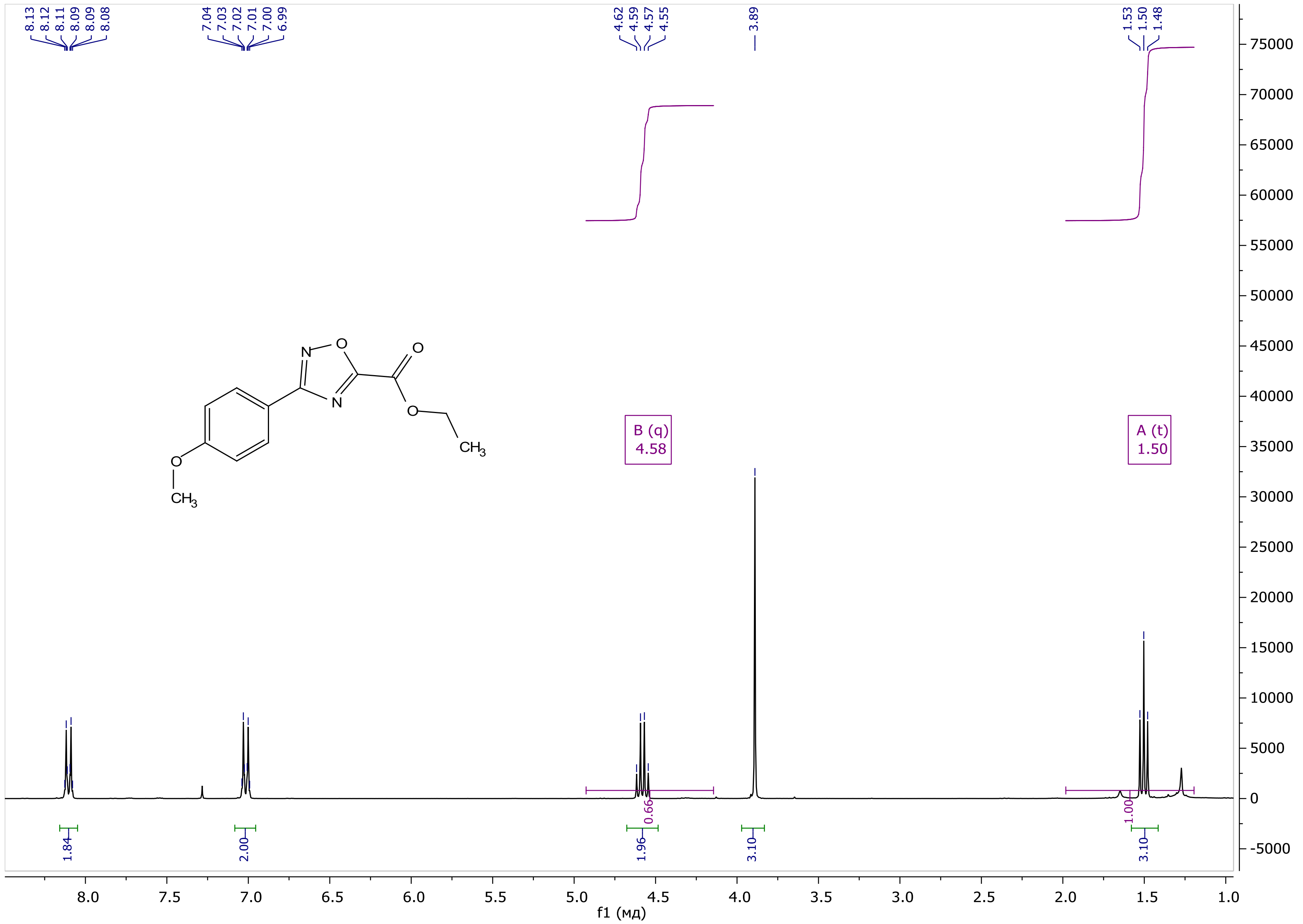

^{13}C NMR spectrum of compound **3e**

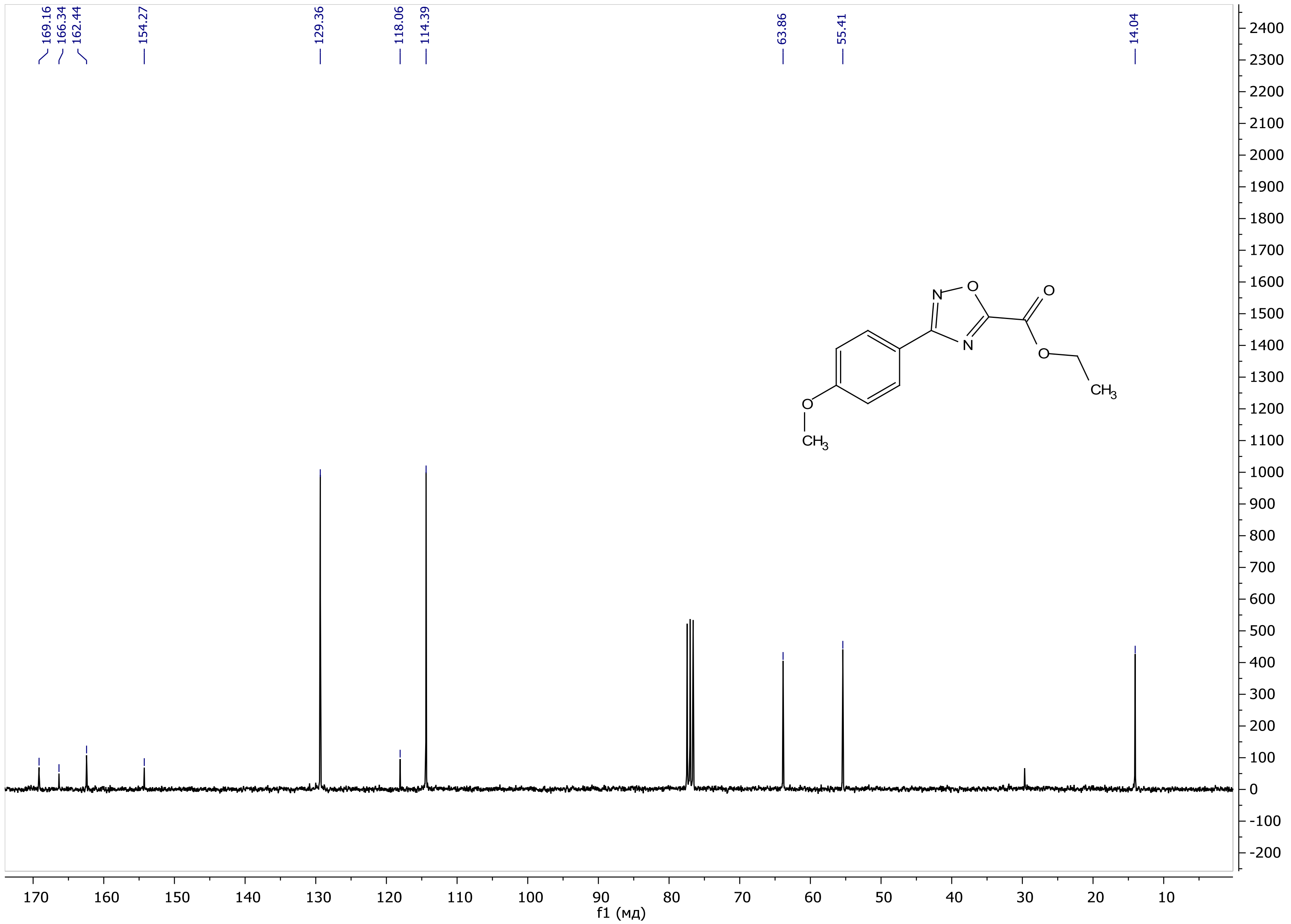

^{1}H NMR spectrum of compound **3f**

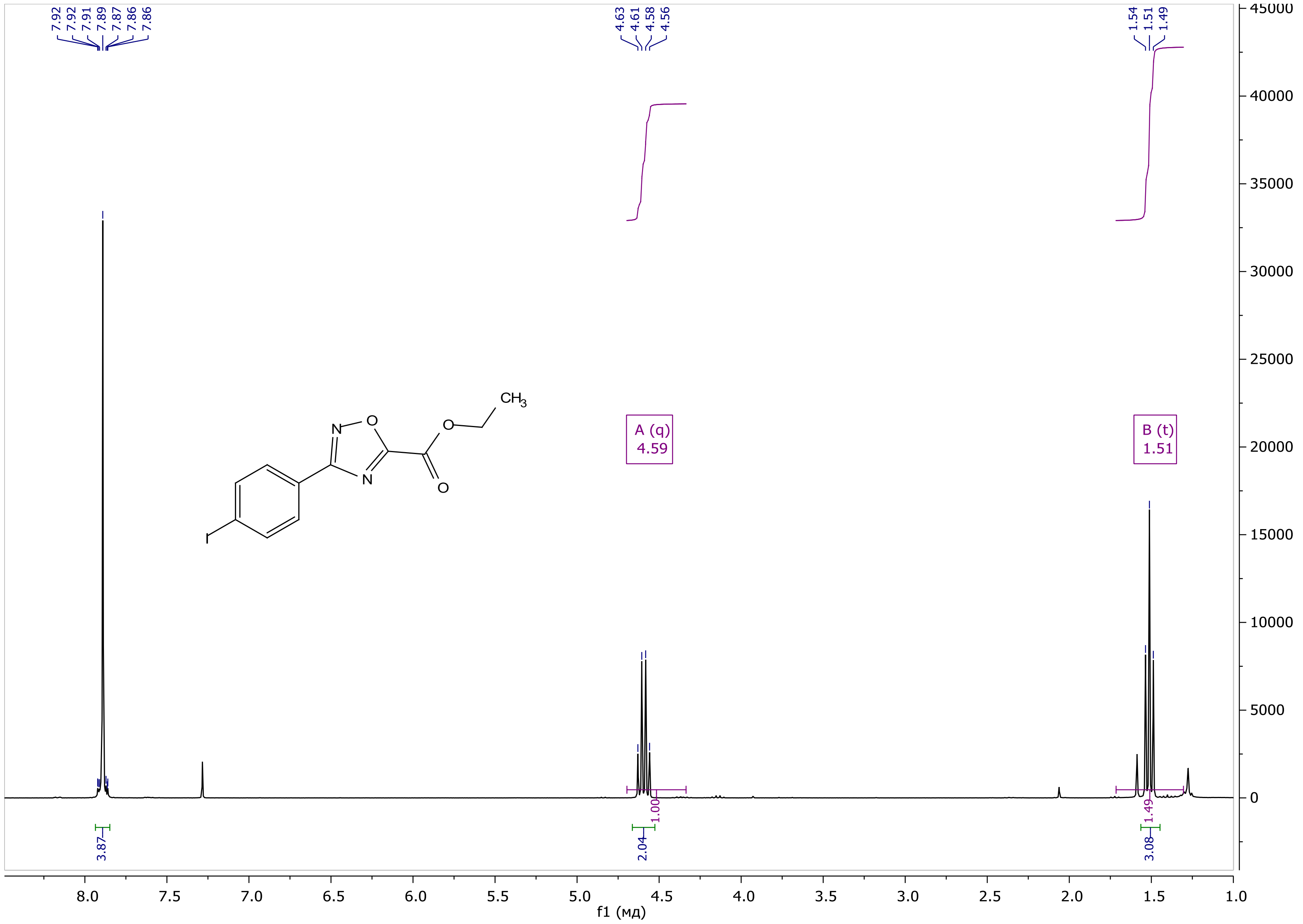

^{13}C NMR spectrum of compound **3f**

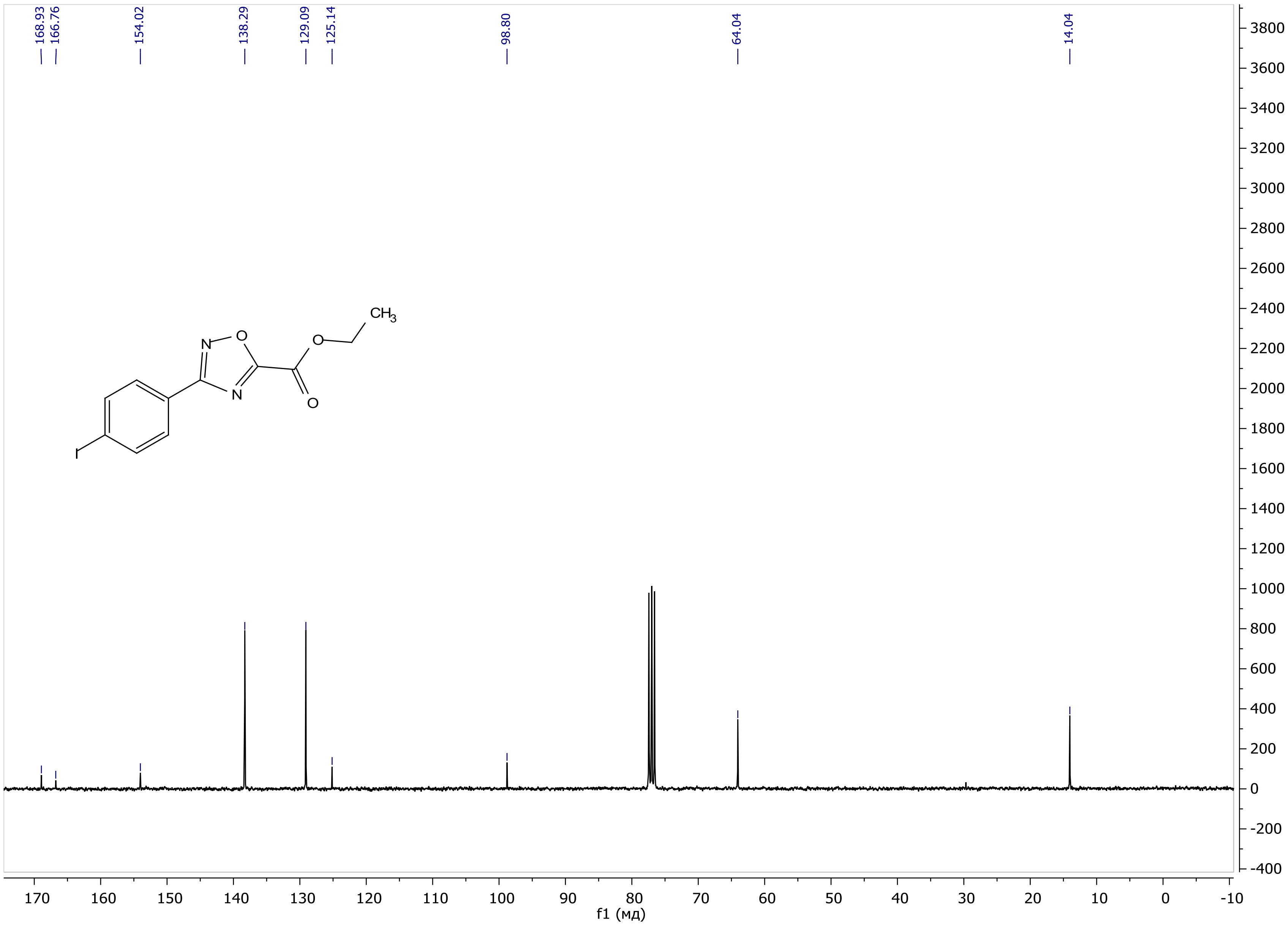

^{1}H, ^{13}C NMR spectra for methyl 2-(3'-aryl-1',2',4'-oxadiazol-5'-yl)acetates and methyl 3-(3-aryl-1,2,4-oxadiazol-5-yl)benzoates synthesized

^{1}H NMR spectrum of compound **8a**

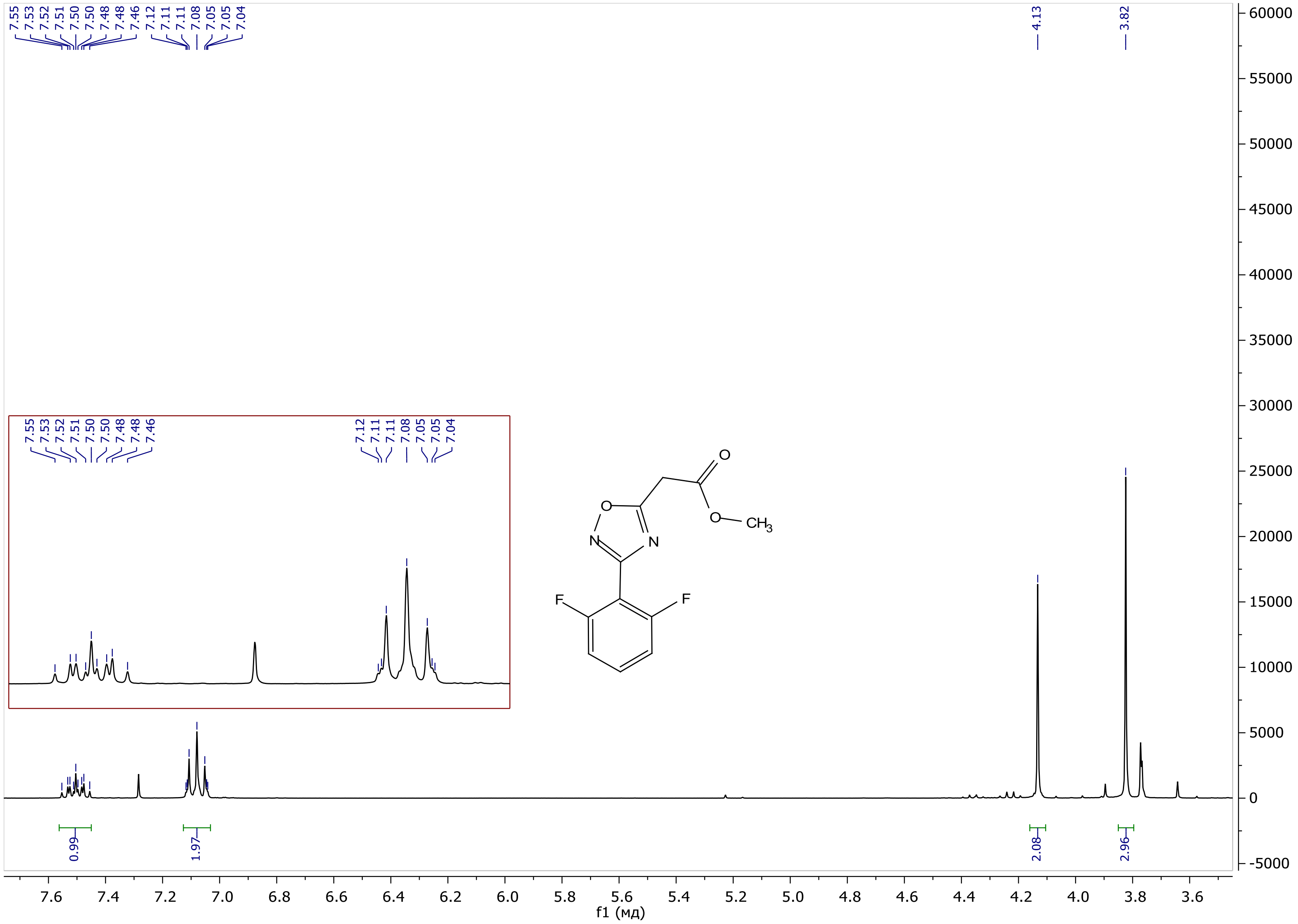

^{13}C NMR spectrum of compound **8a**

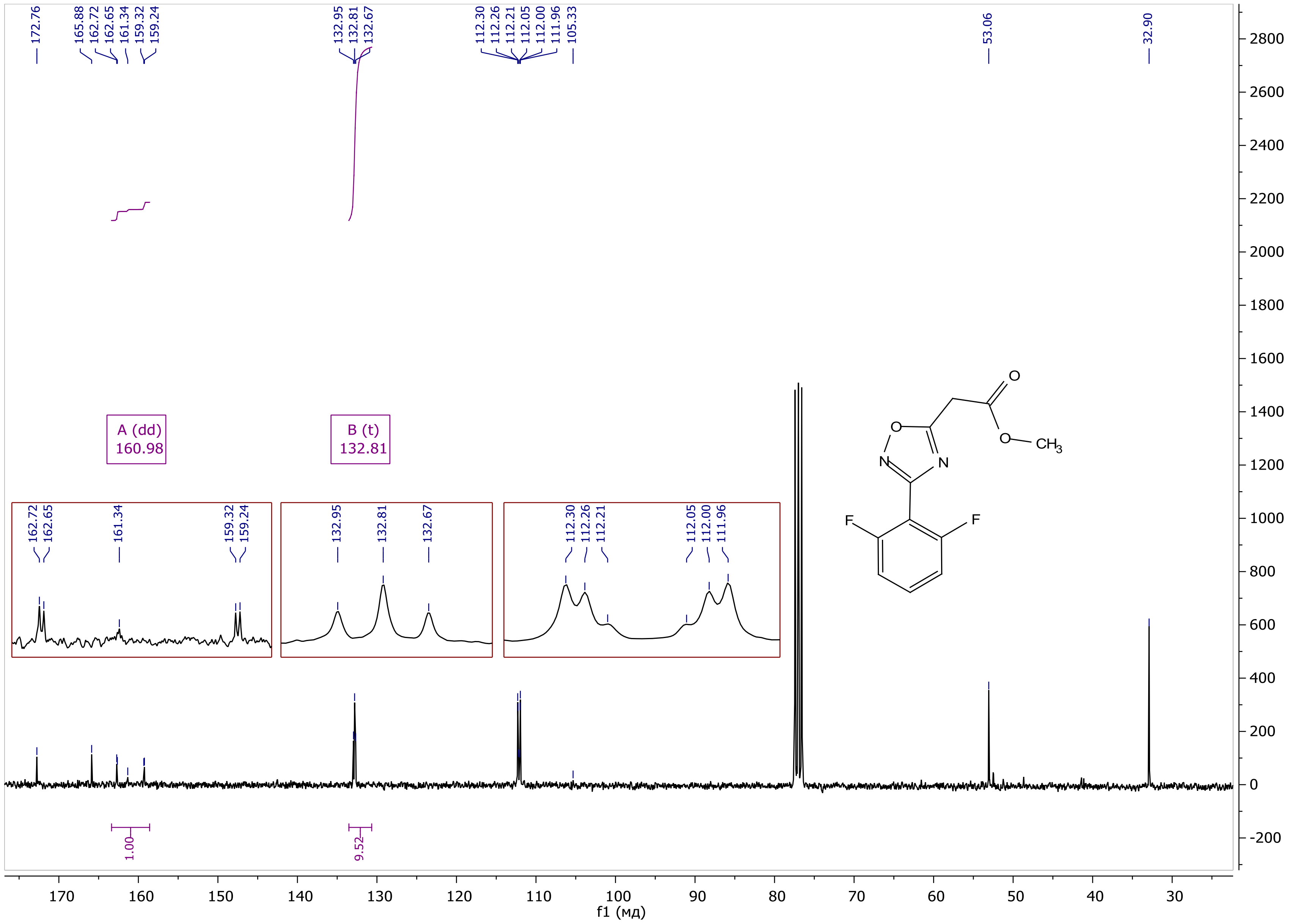

^{1}H NMR spectrum of compound **8b**

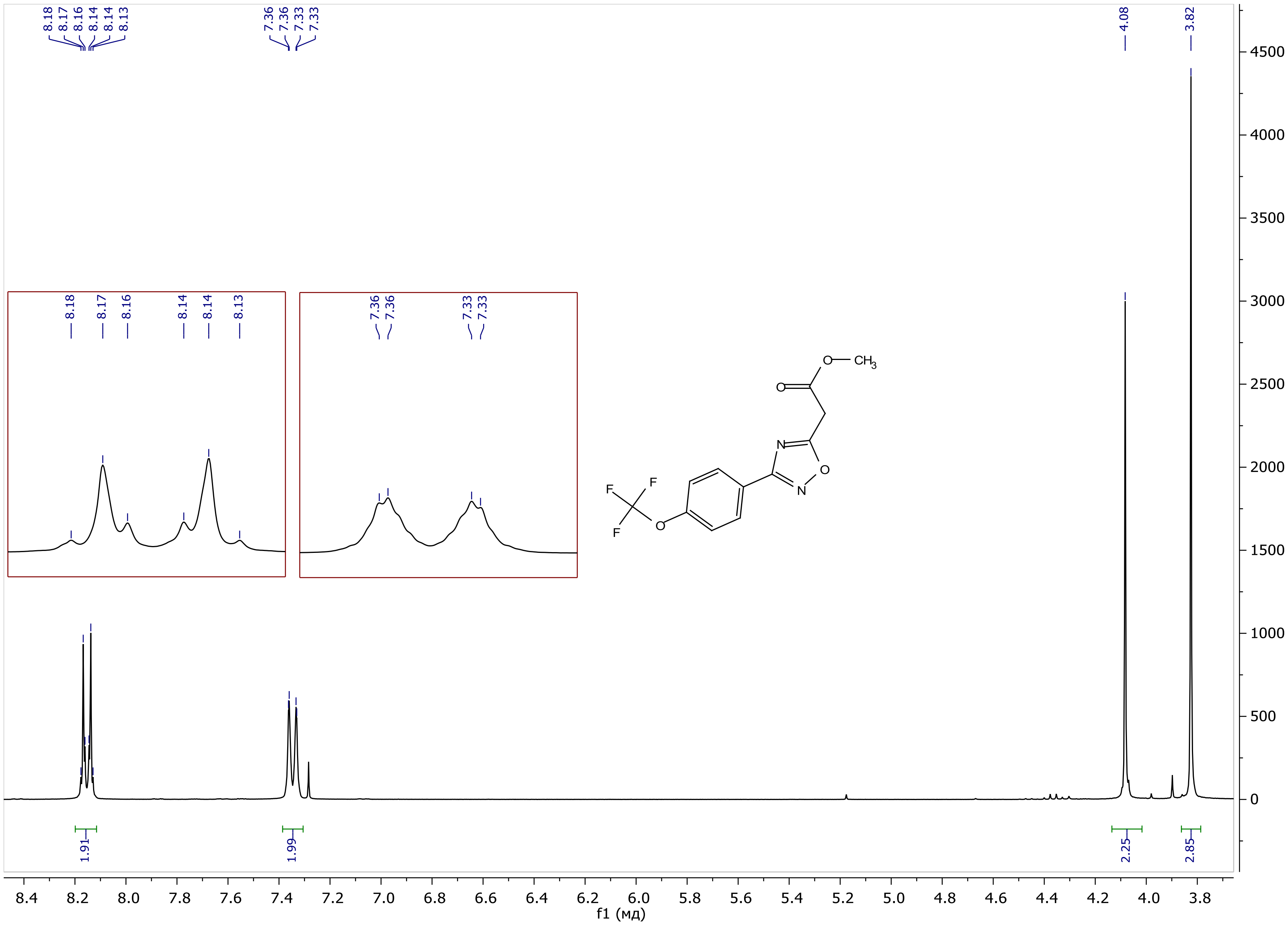

^{13}C NMR spectrum of compound **8b**

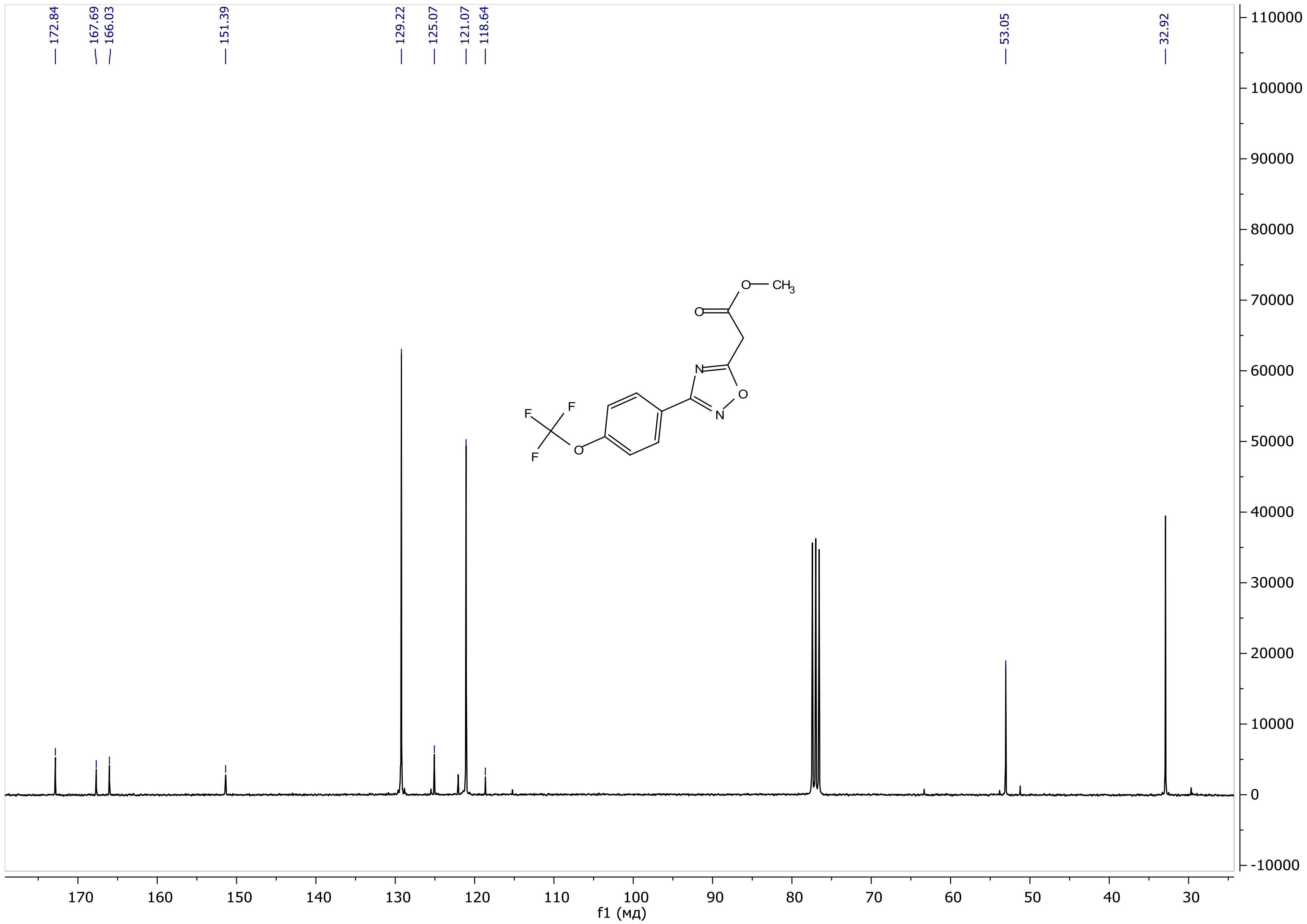

^{1}H NMR spectrum of compound **8c**

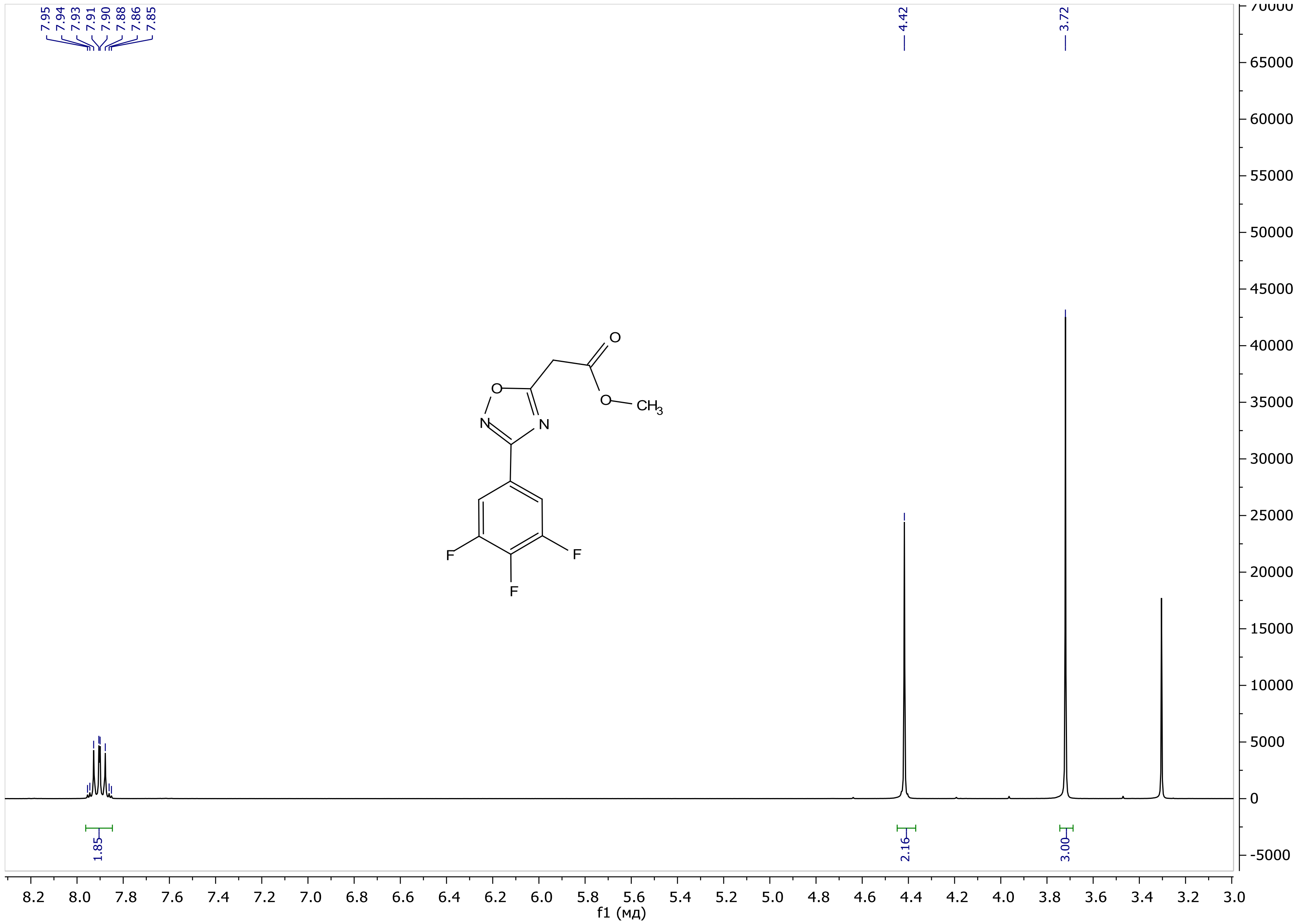

^{13}C NMR spectrum of compound **8c**

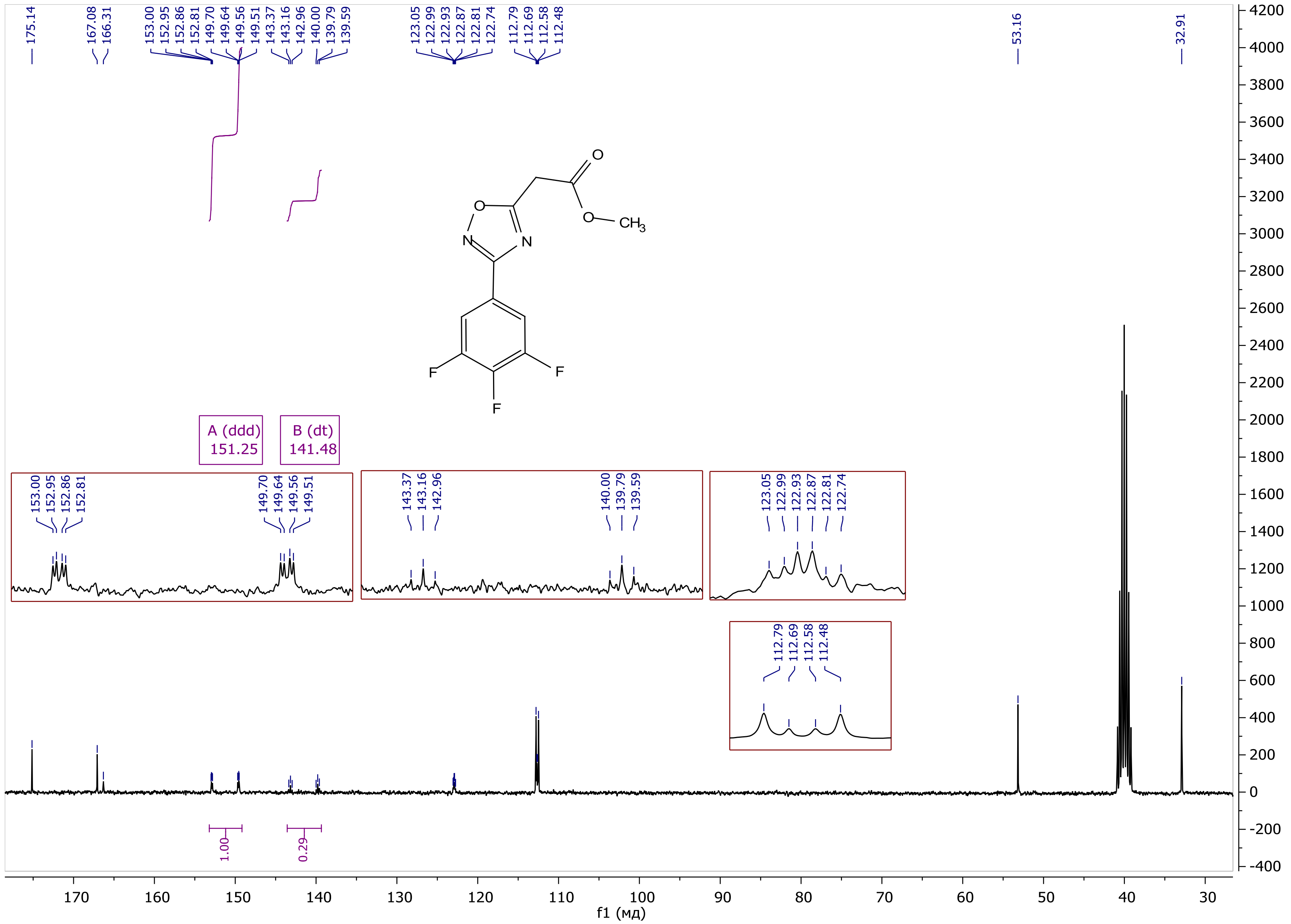

^{1}H NMR spectrum of compound **8d**

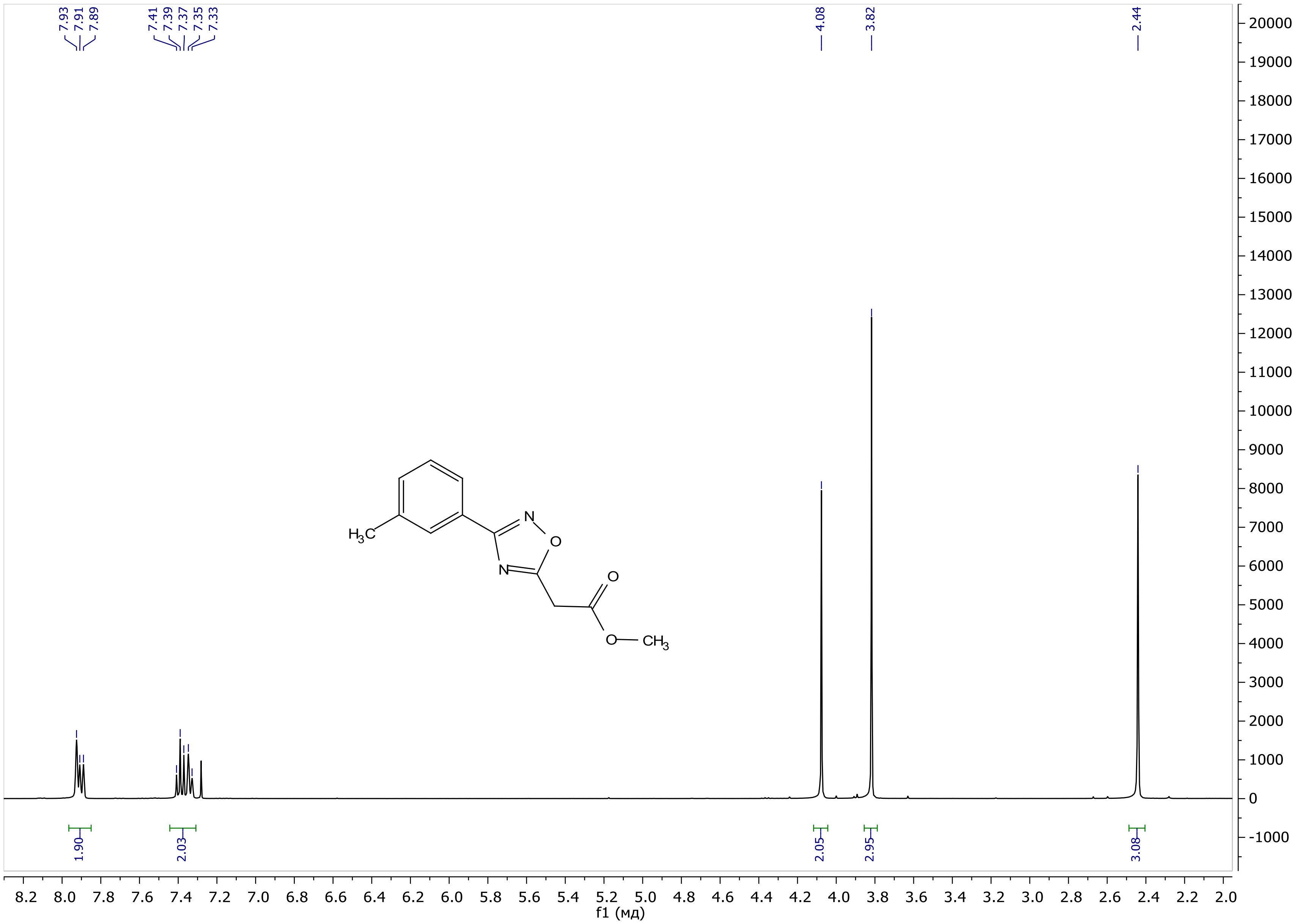

^{13}C NMR spectrum of compound **8d**

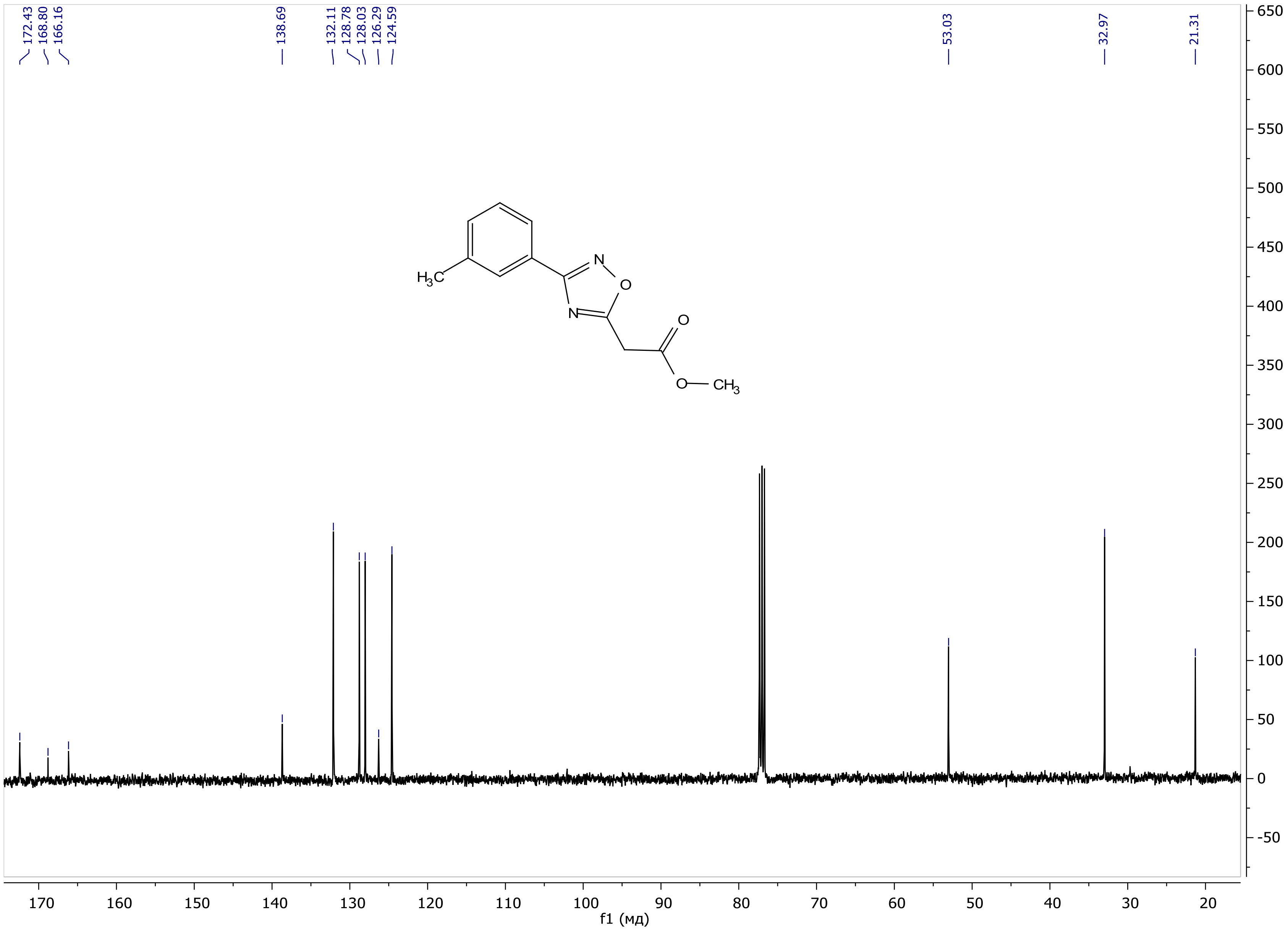

^{1}H NMR spectrum of compound **12a**

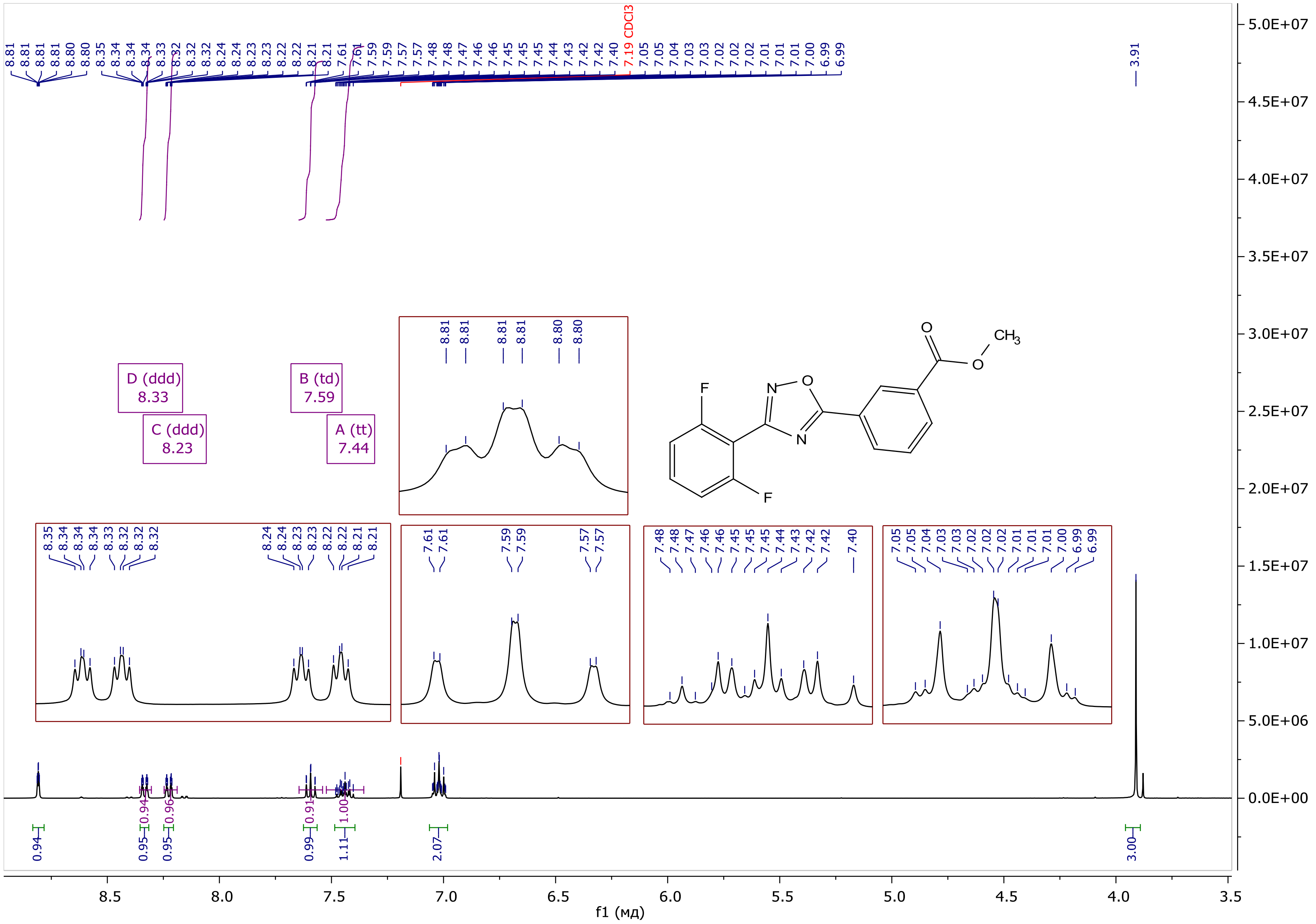

^{13}C NMR spectrum of compound **12a**

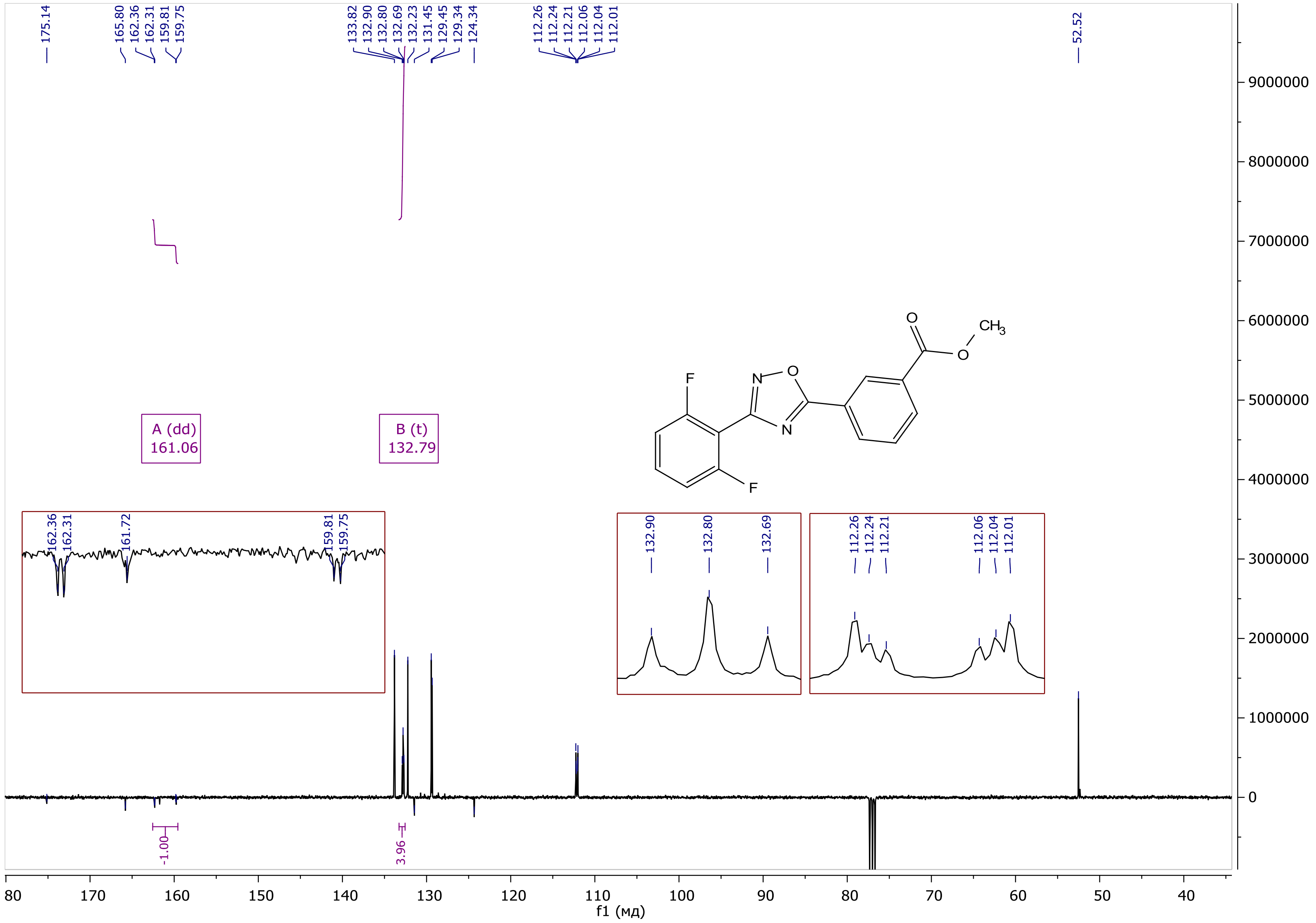

^{1}H NMR spectrum of compound **12b**

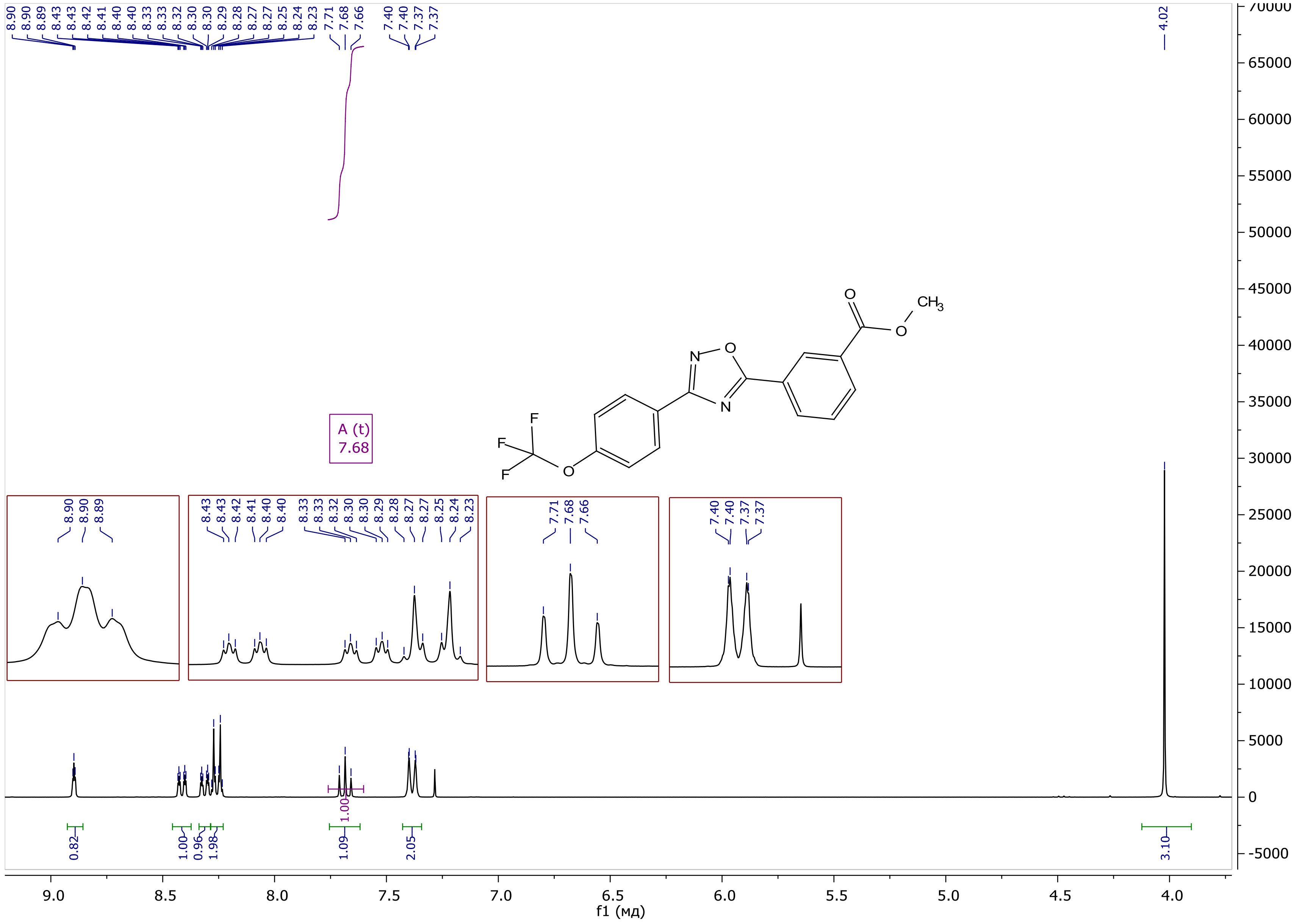

^{13}C NMR spectrum of compound **12b**

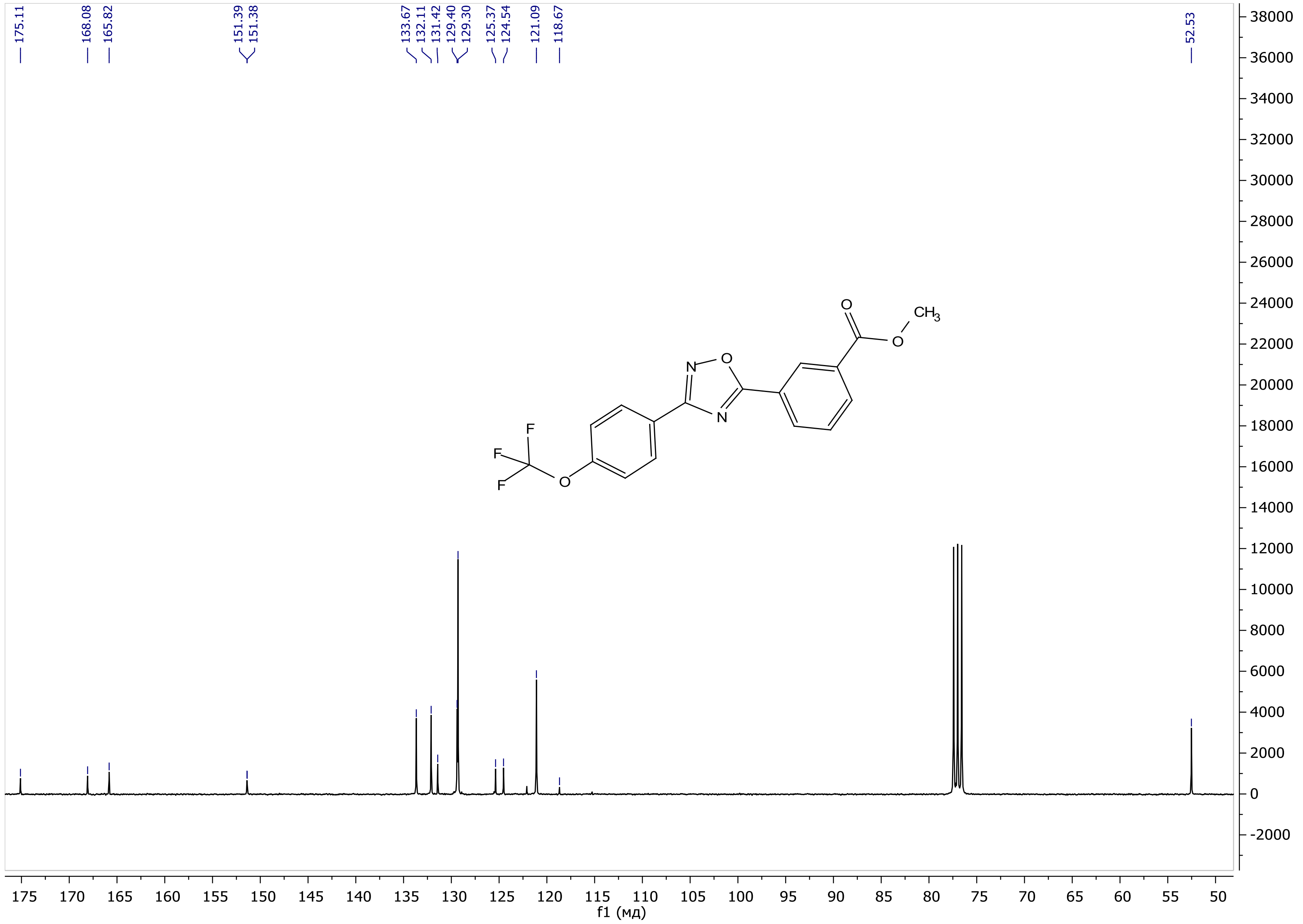

^{1}H NMR spectrum of compound **12d**

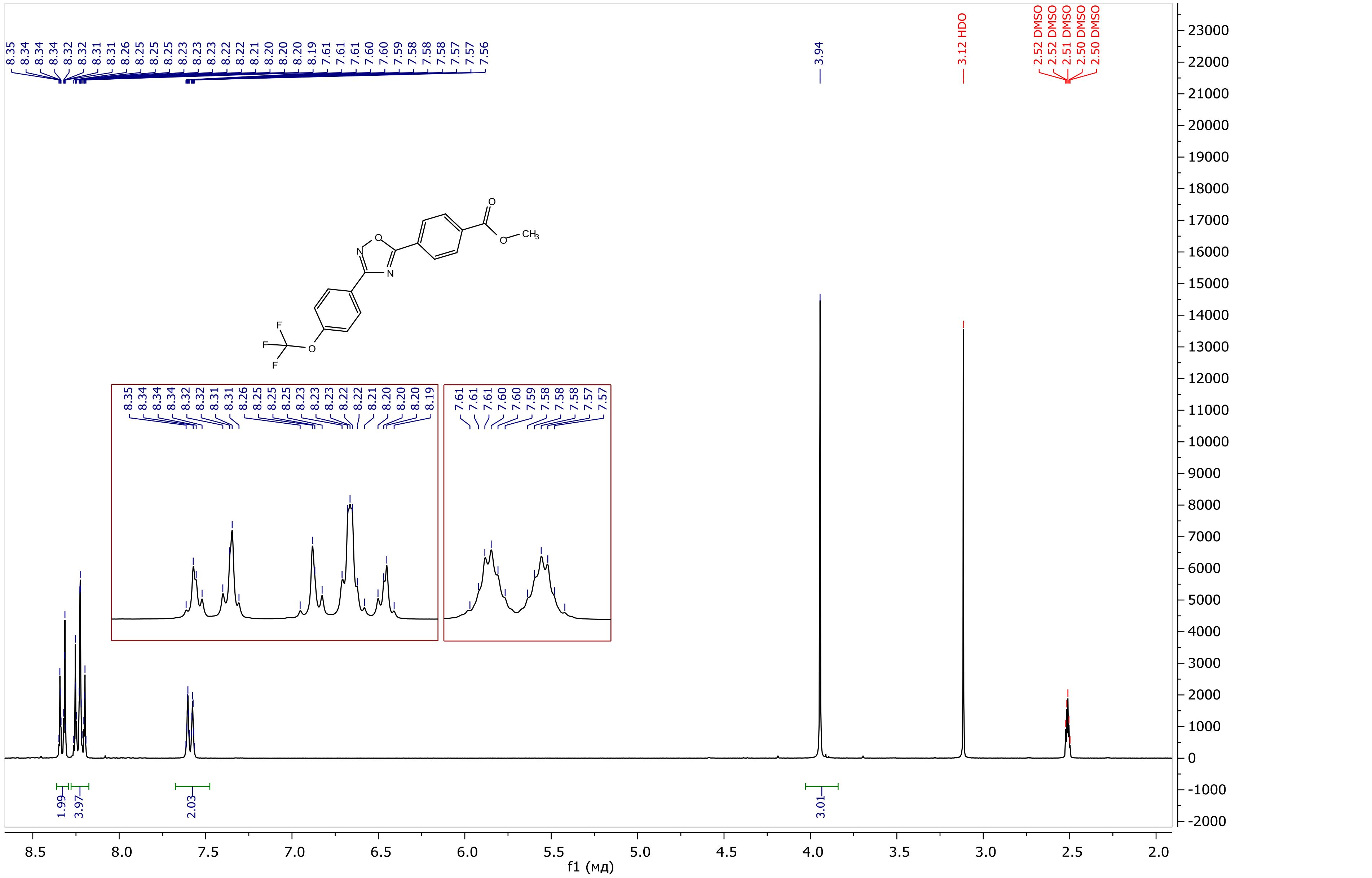

^{13}C NMR spectrum of compound **12d**

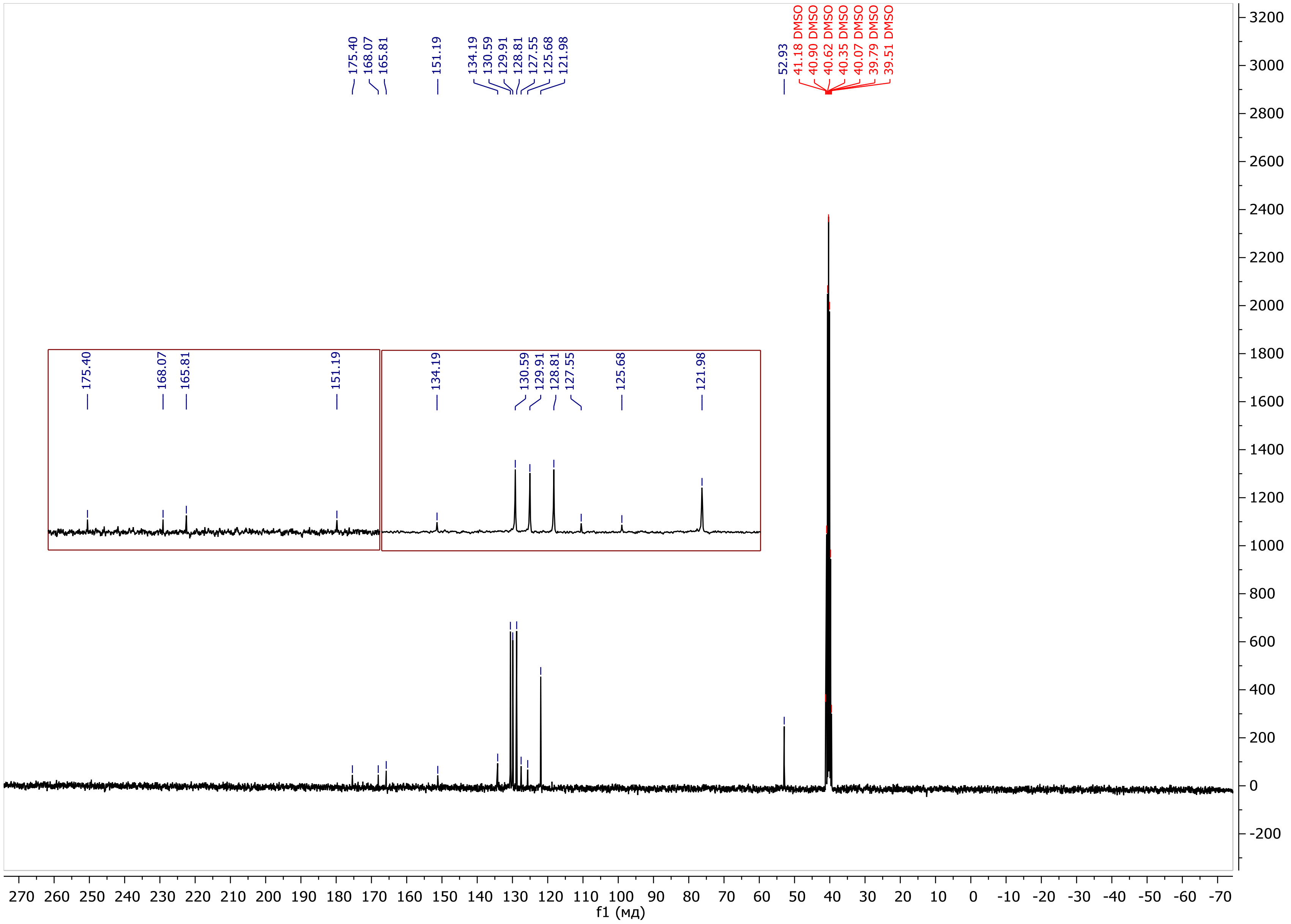

^{1}H NMR spectrum of compound **12e**

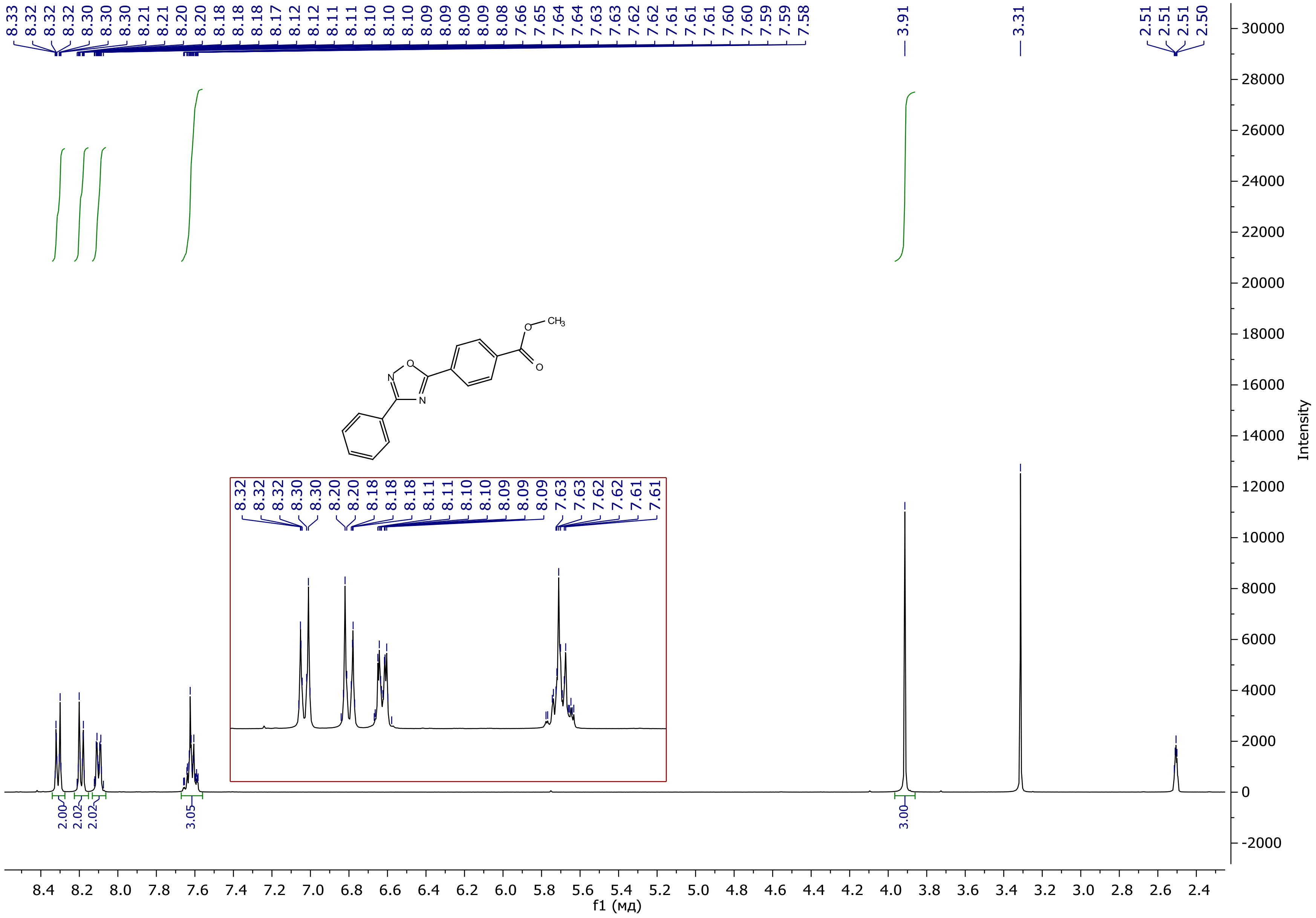

^{13}C NMR spectrum of compound **12e**

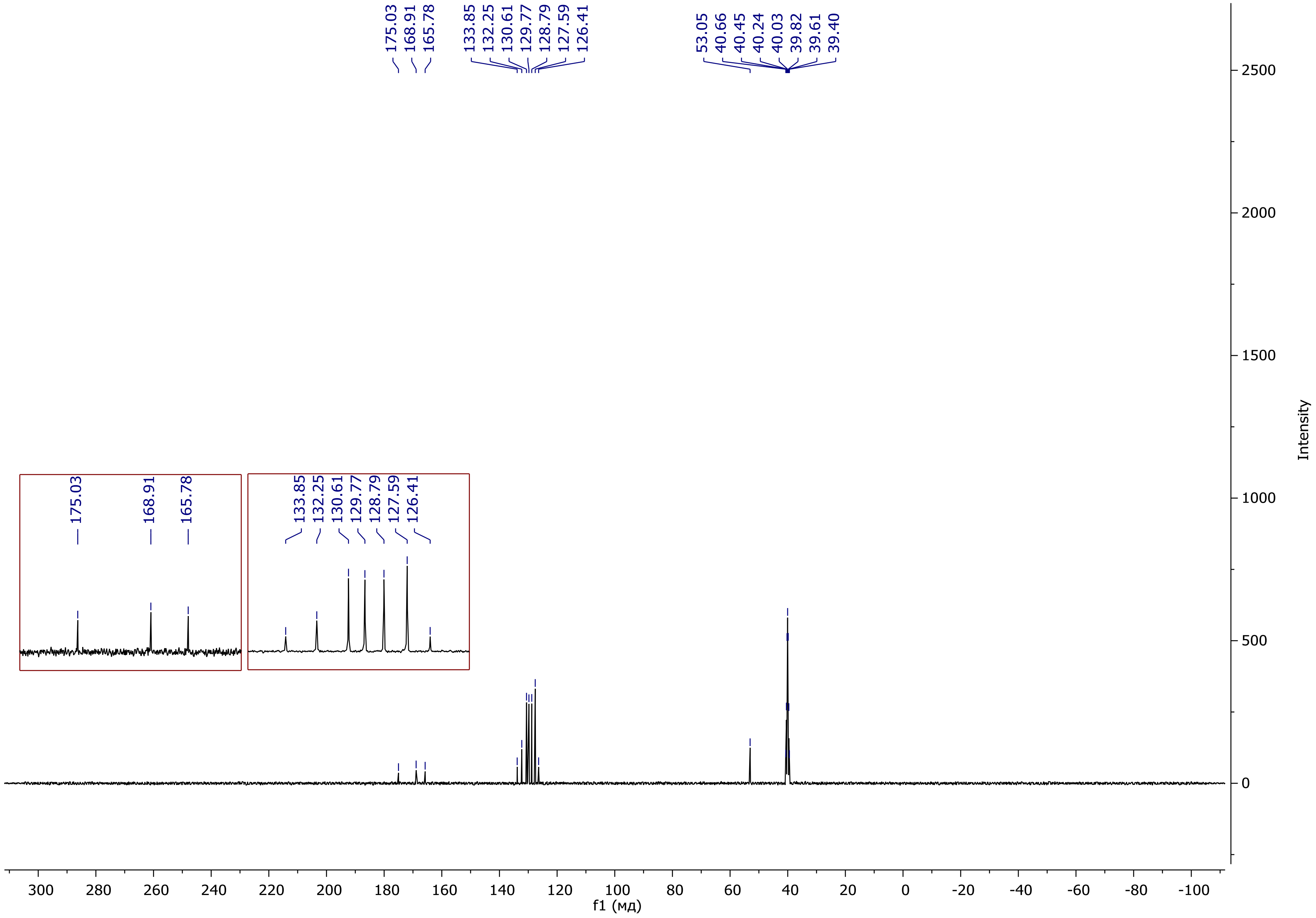

^{1}H, ^{13}C NMR spectra for ethyl 2-(5'-(aryl)-1',2',4'-oxadiazol-3'-yl)acetates synthesized

^{1}H NMR spectrum of compound **22a**

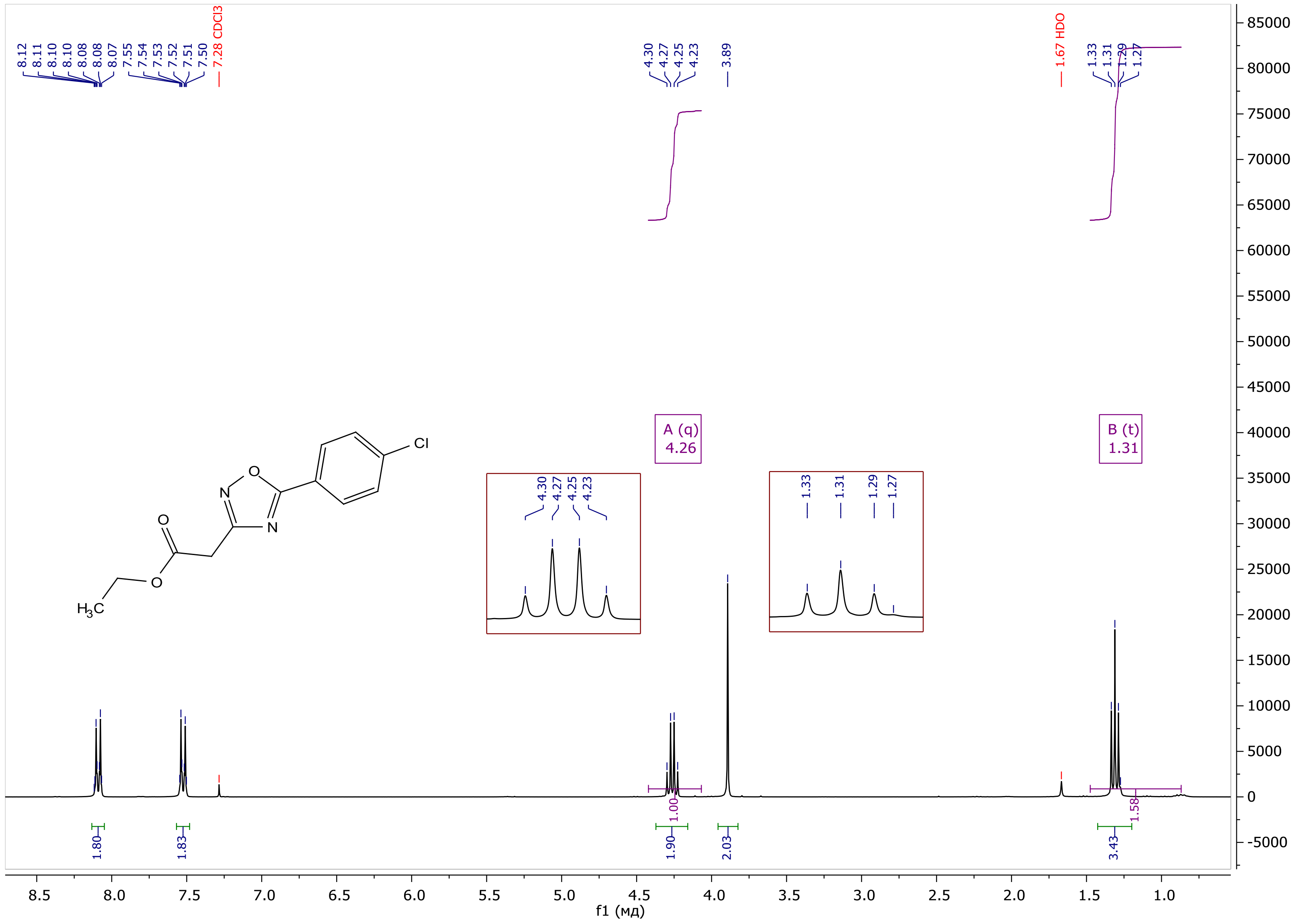

^{13}C NMR spectrum of compound **22a**

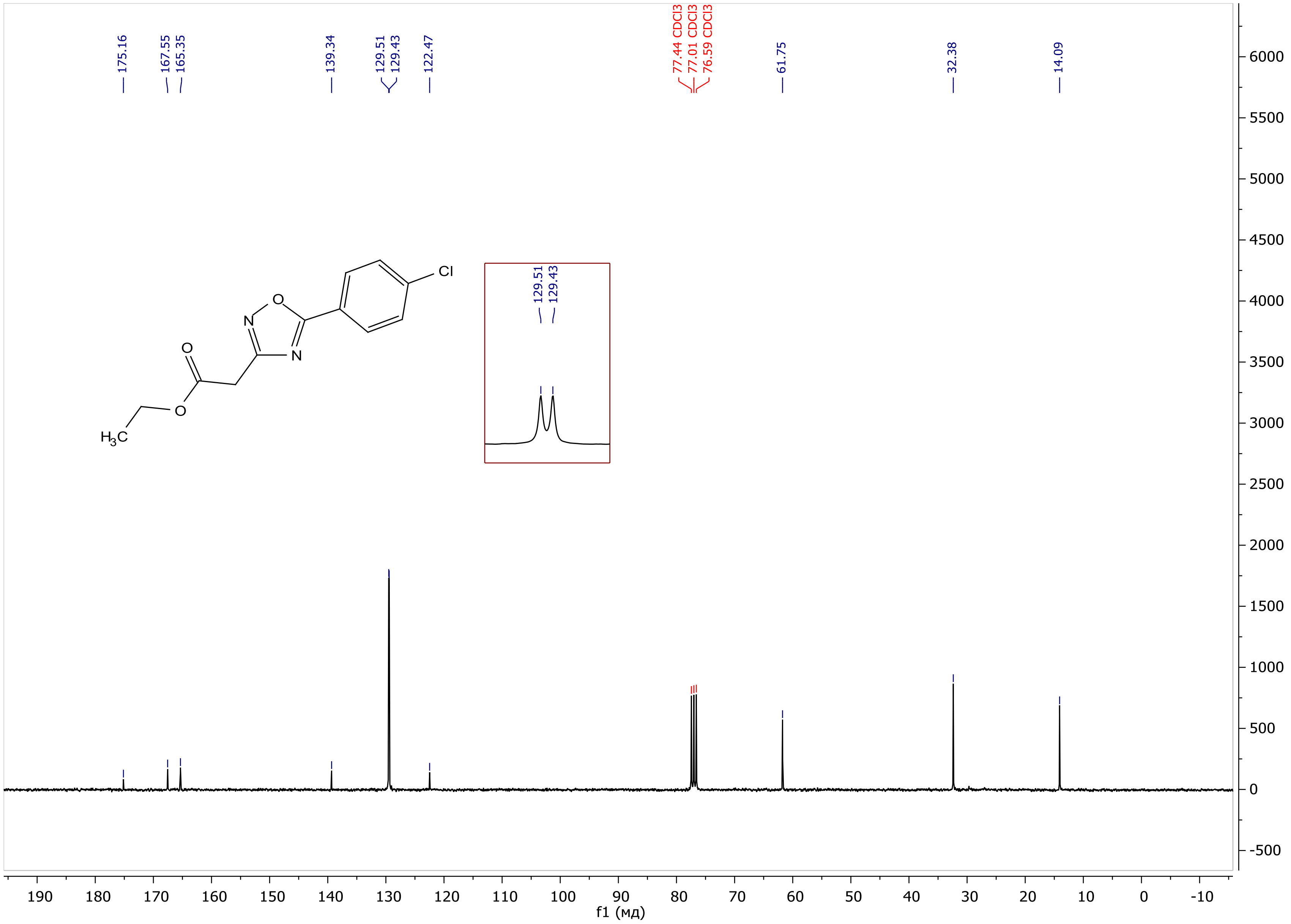

^{1}H NMR spectrum of compound **22b**

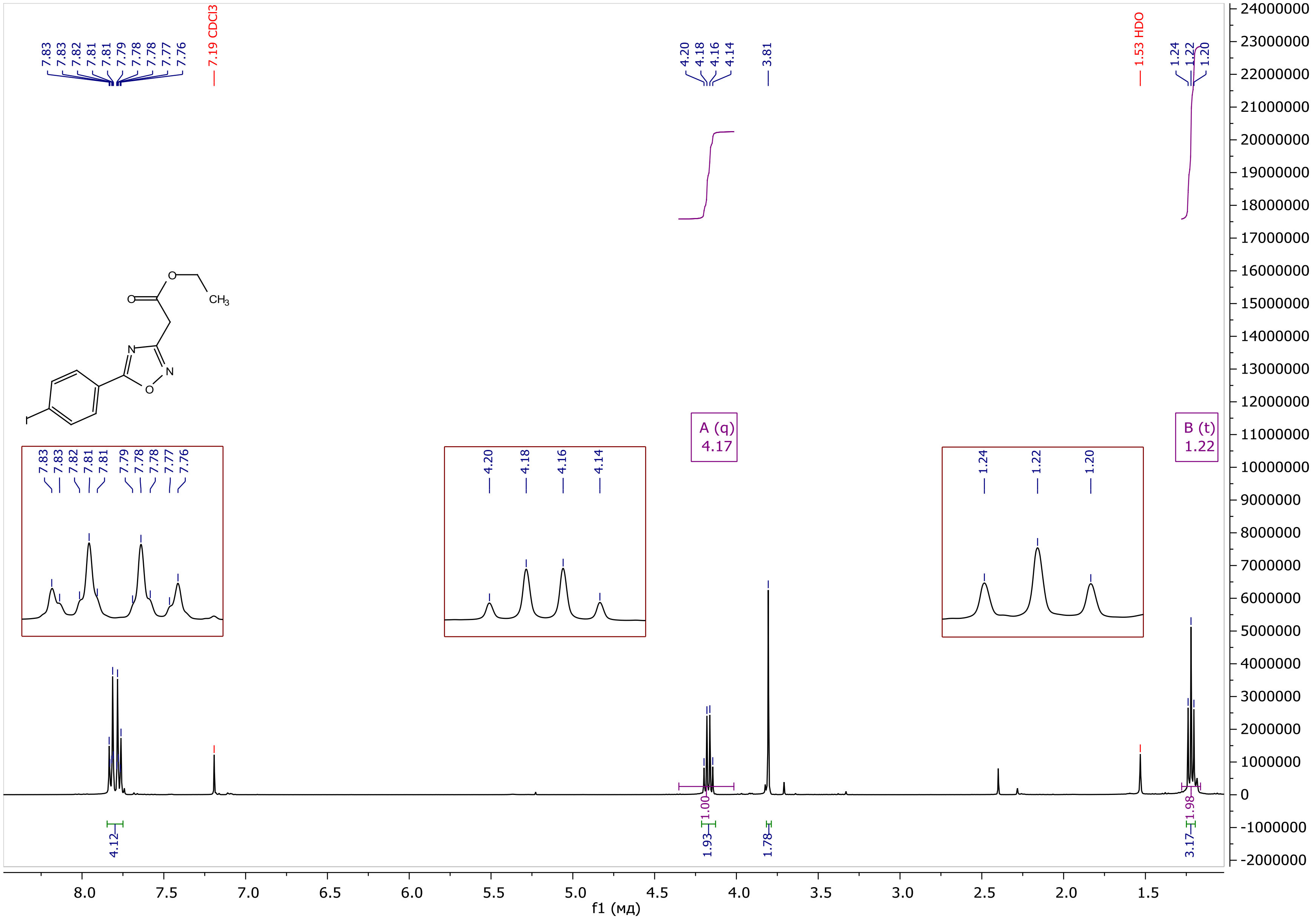

^{13}C NMR spectrum of compound **22b**

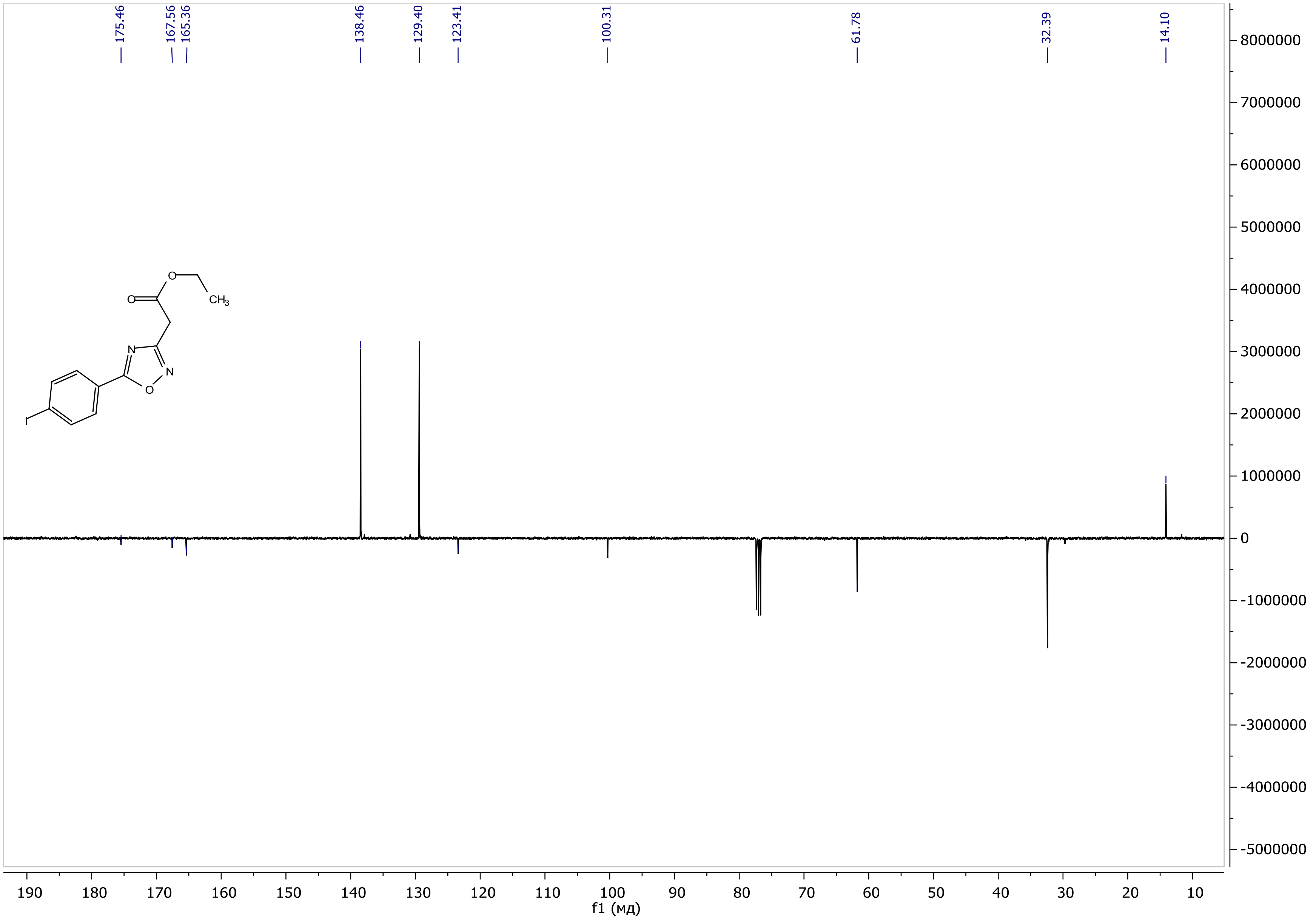

^{1}H NMR spectrum of compound **22c**

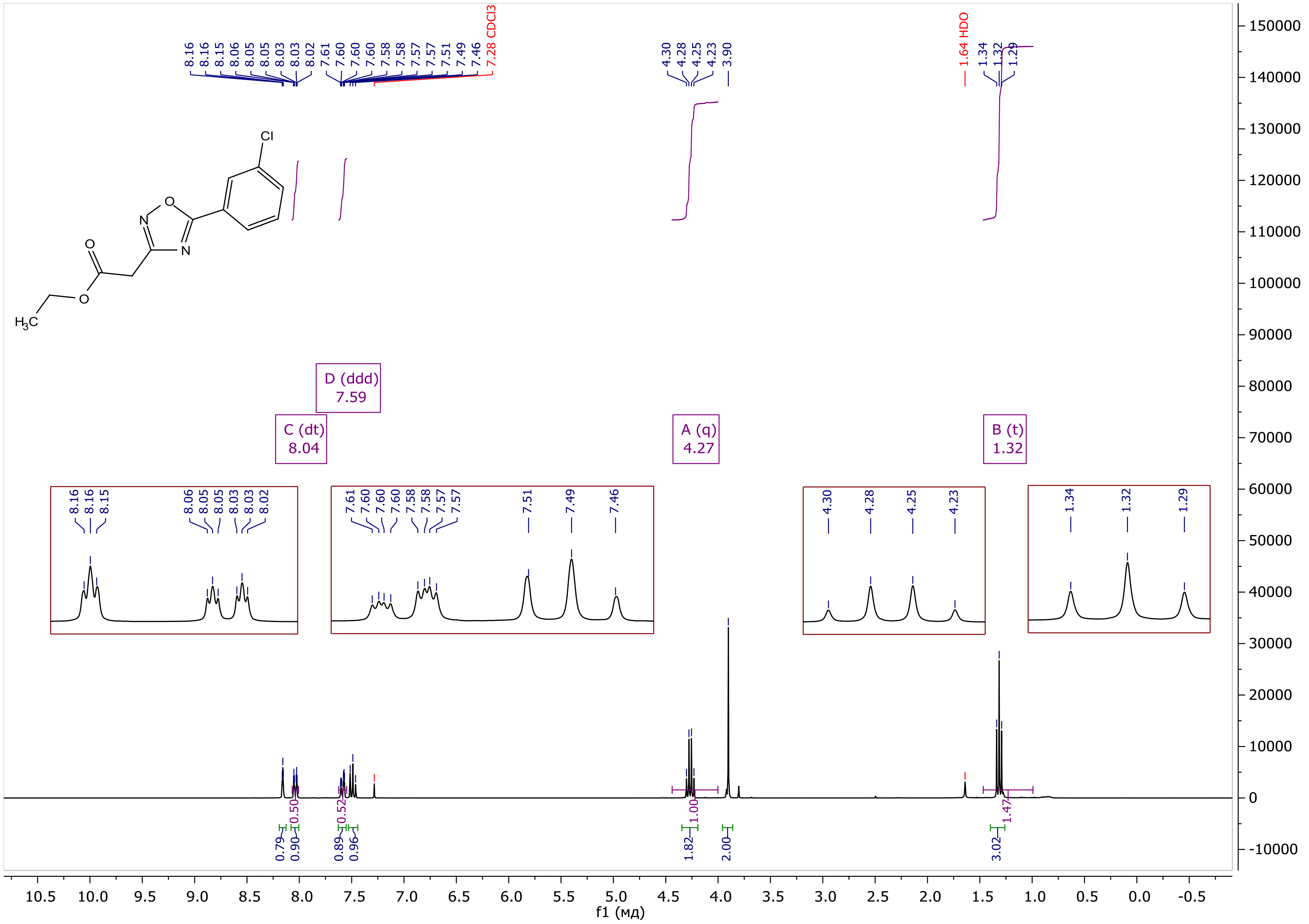

^{13}C NMR spectrum of compound **22c**

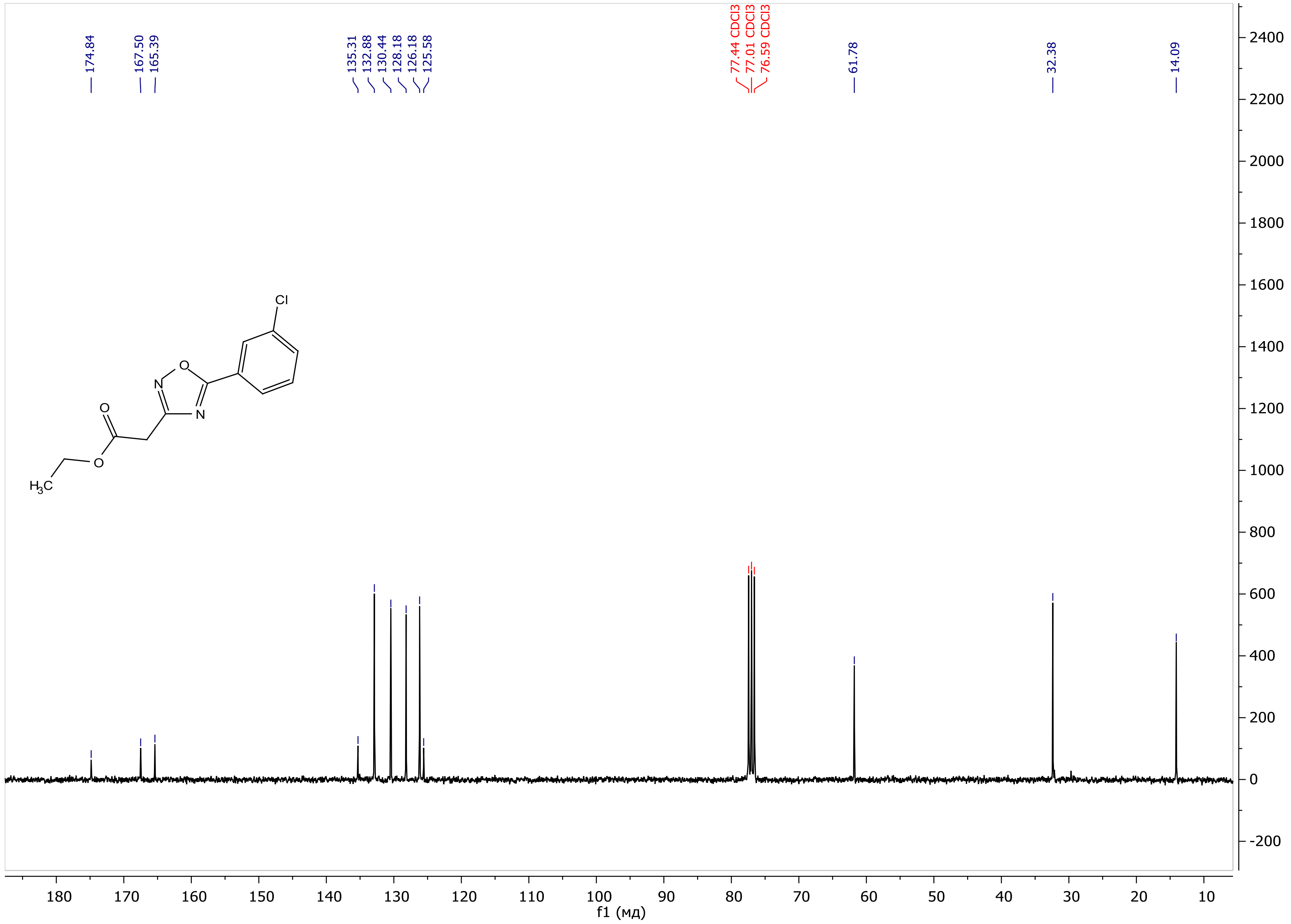

^{1}H NMR spectrum of compound **22d**

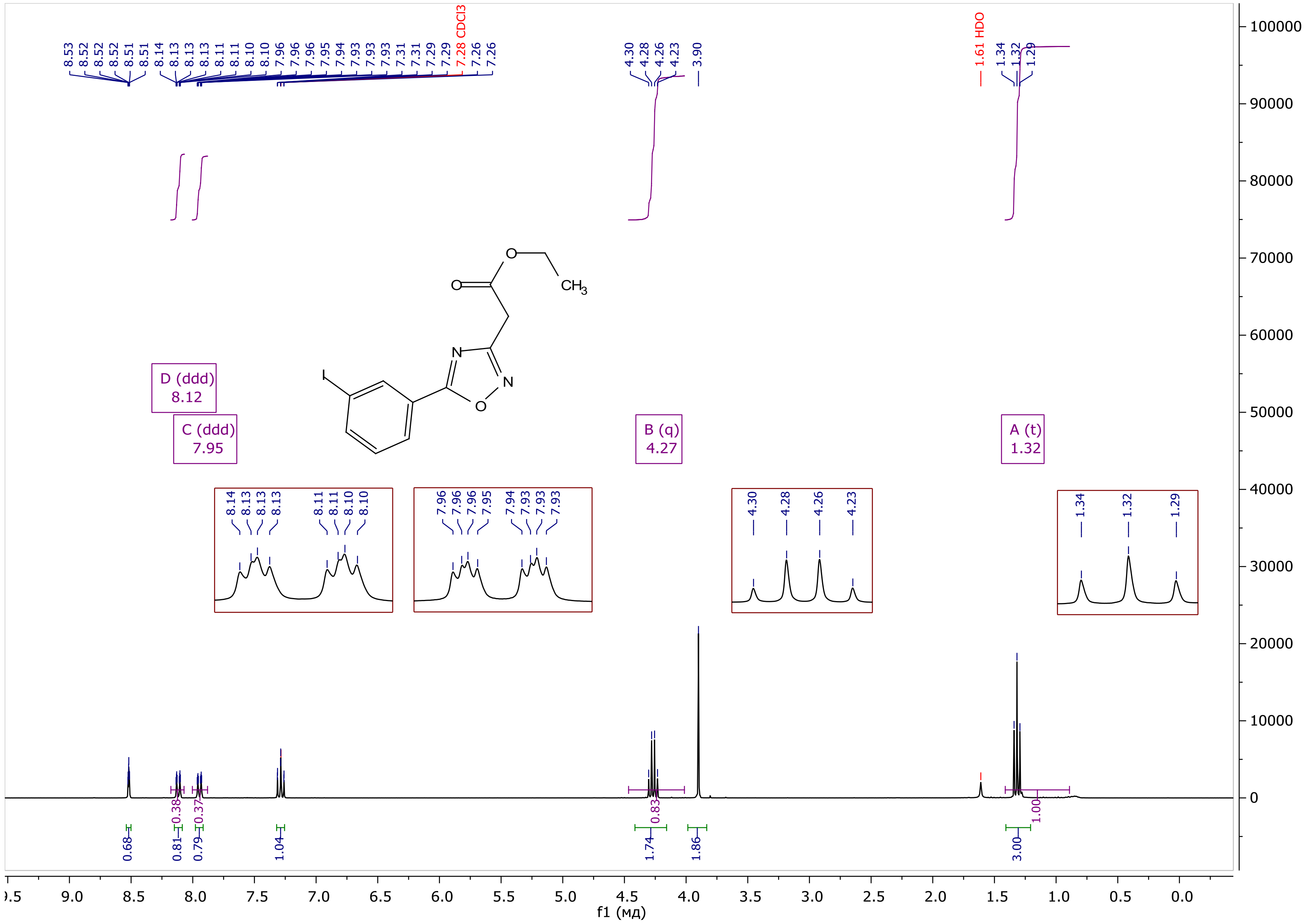

^{13}C NMR spectrum of compound **22d**

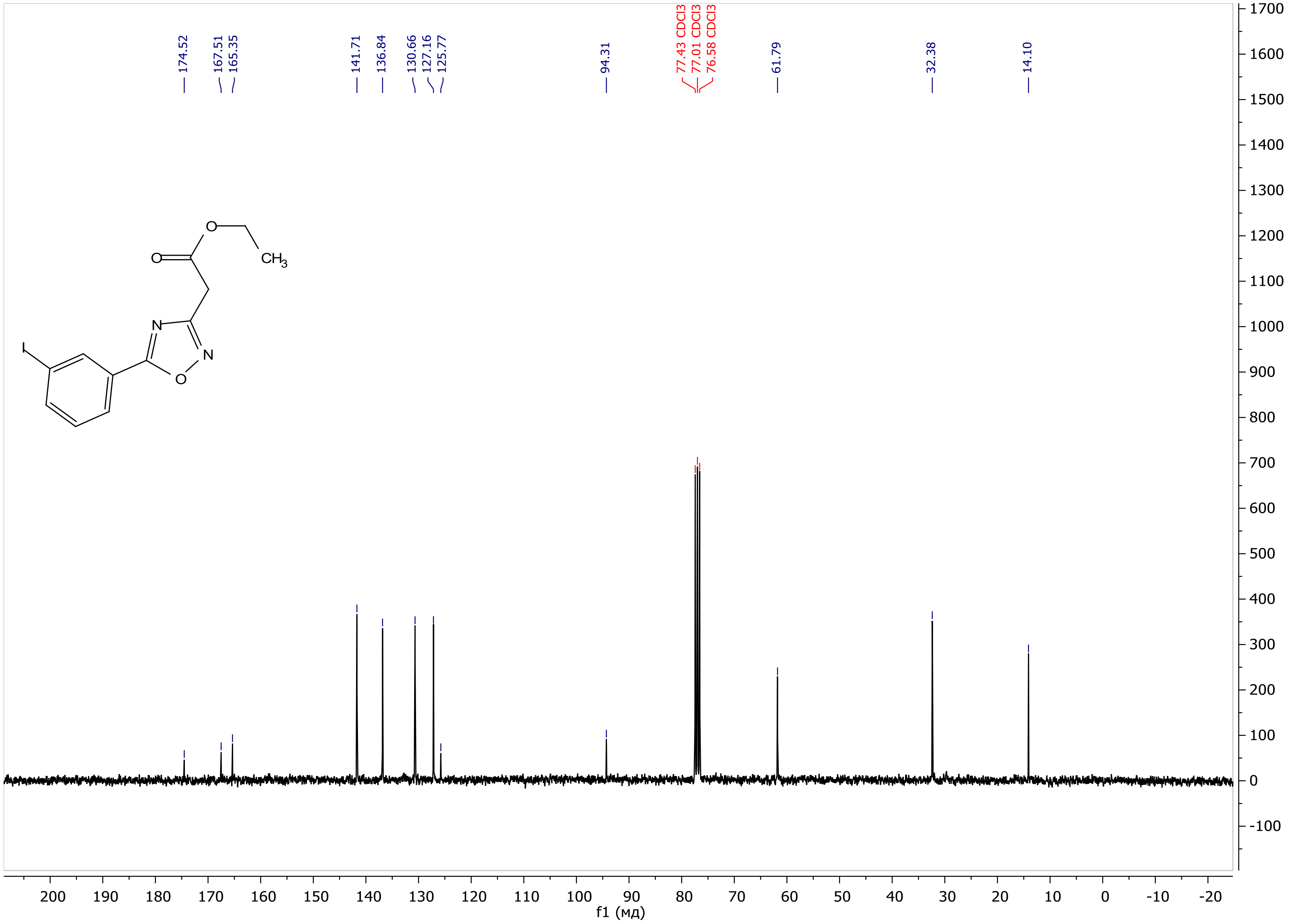

^{1}H, ^{13}C NMR spectra for 3-(3-aryl-1,2,4-oxadiazol-5-yl)benzoic acids synthesized

^{1}H NMR spectrum of compound **13a**

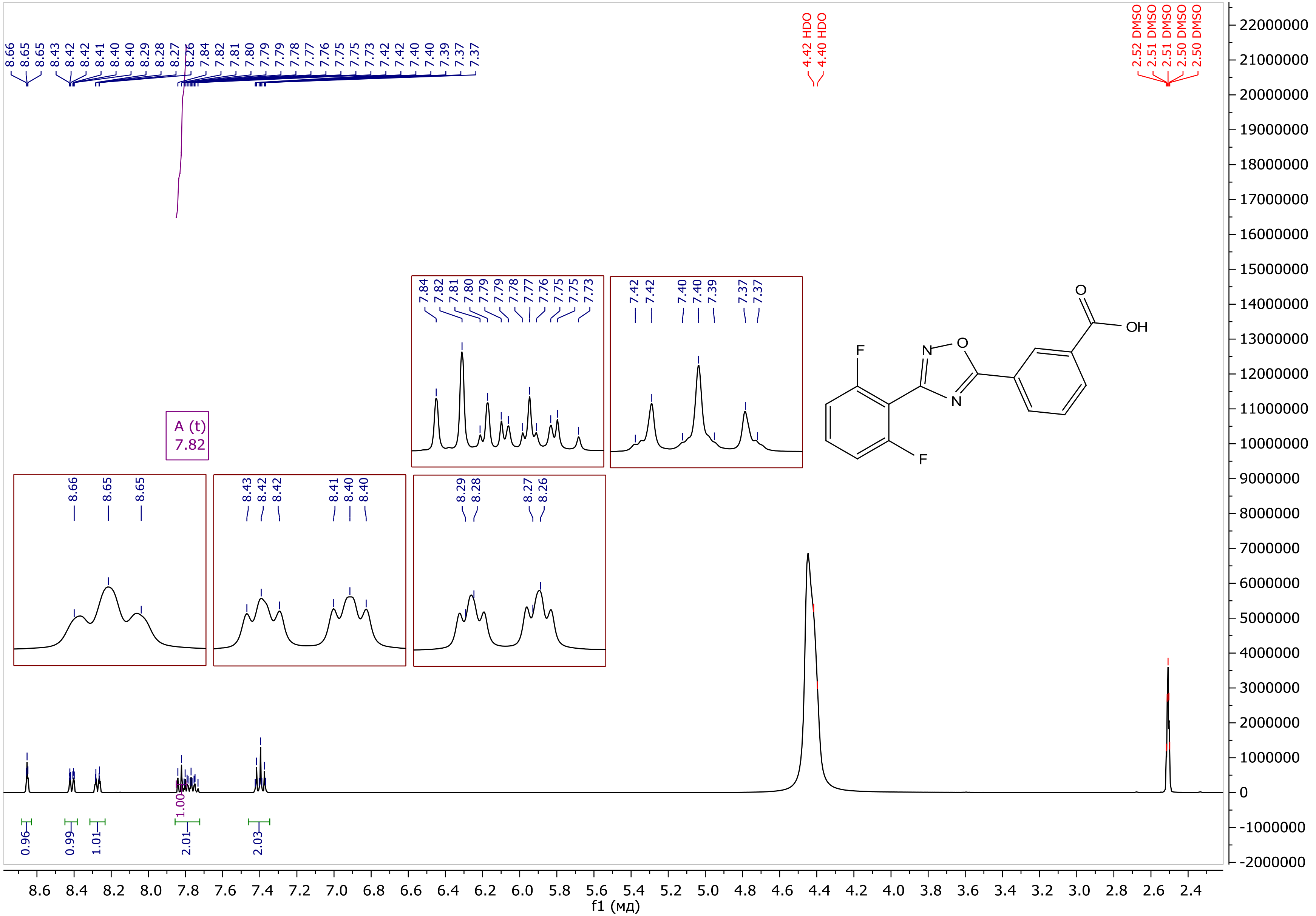

^{13}C NMR spectrum of compound **13a**

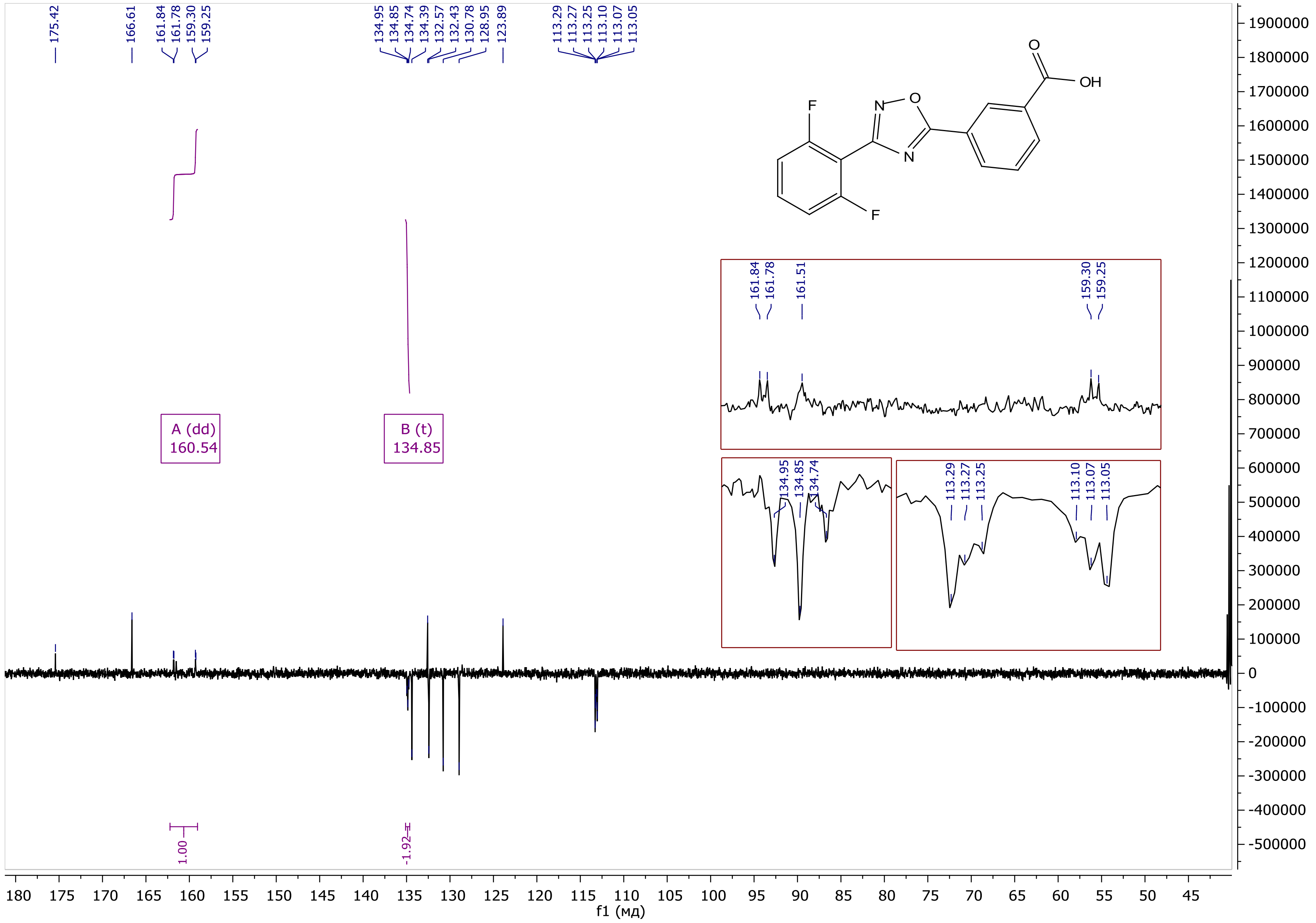

^{1}H NMR spectrum of compound **13b**

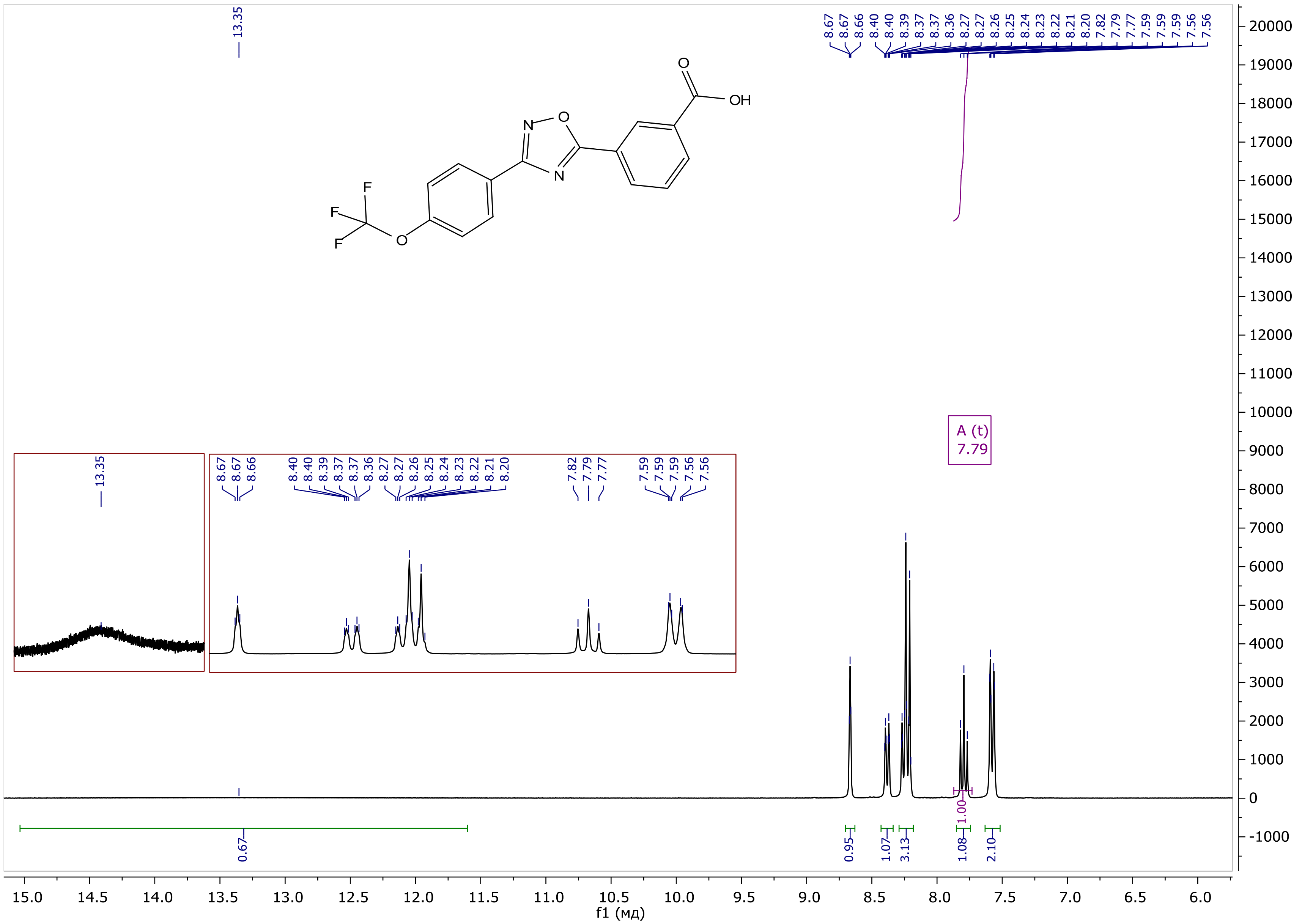

^{13}C NMR spectrum of compound **13b**

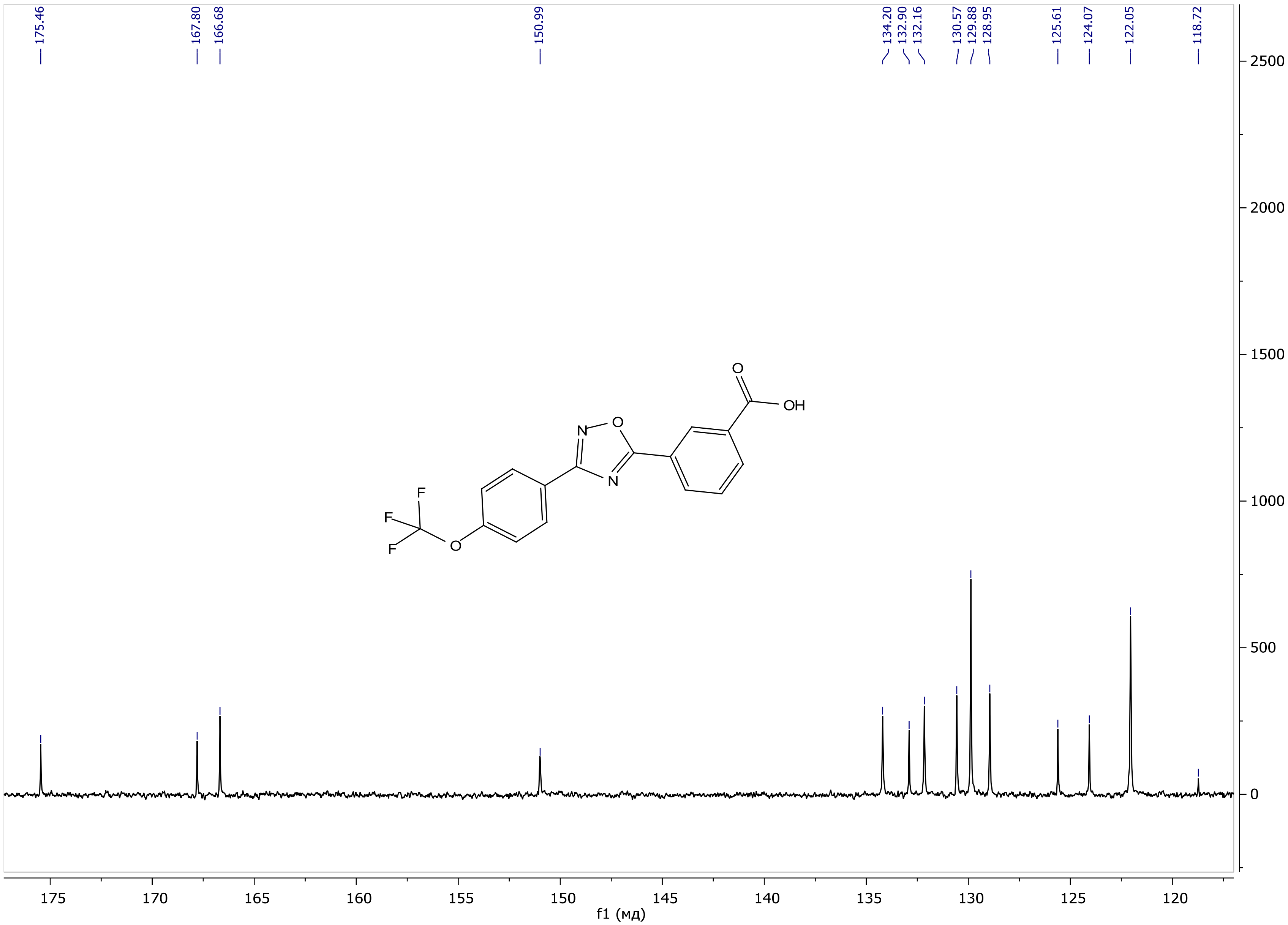

^{1}H NMR spectrum of compound **13c**

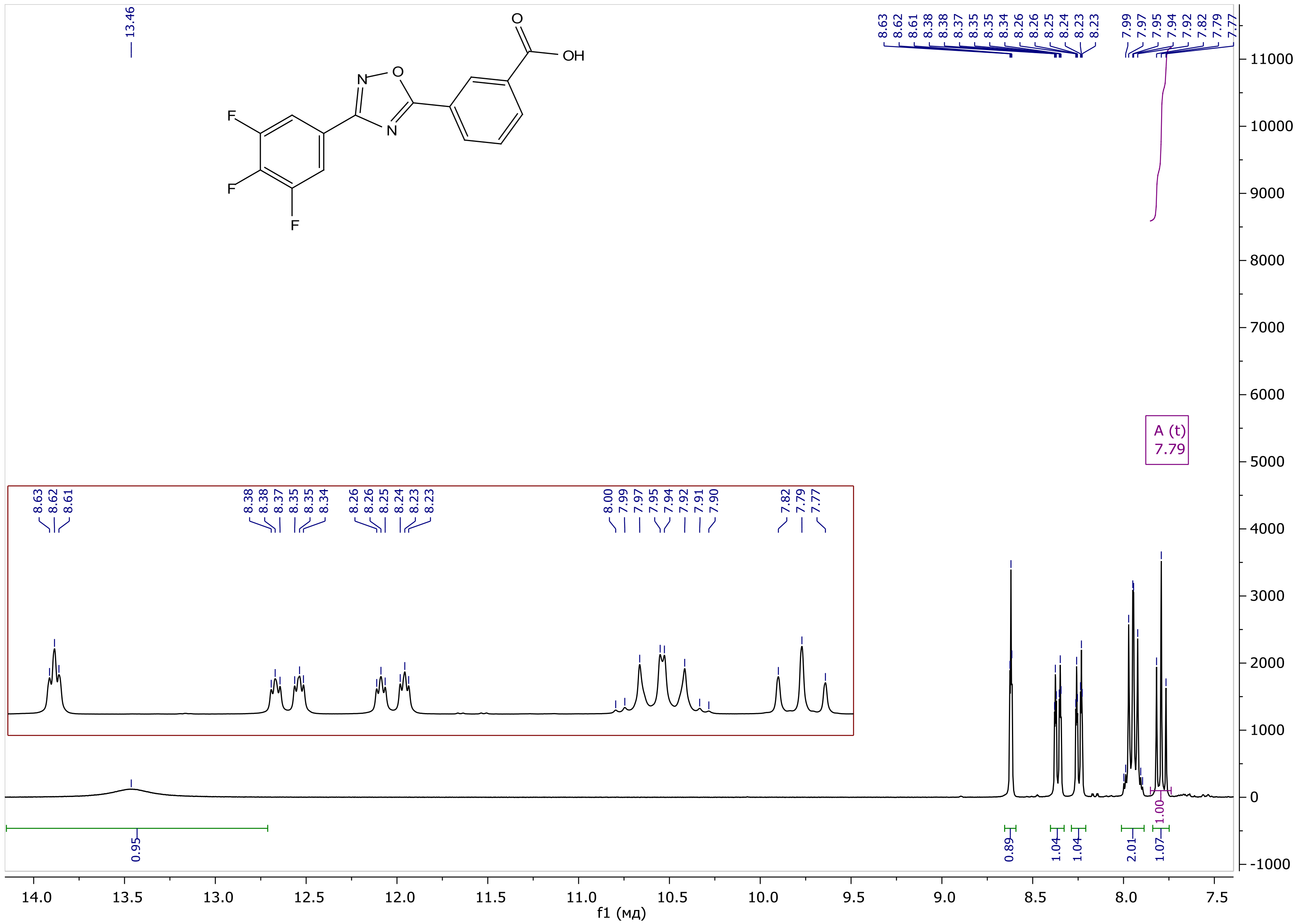

^{13}C NMR spectrum of compound **13c**

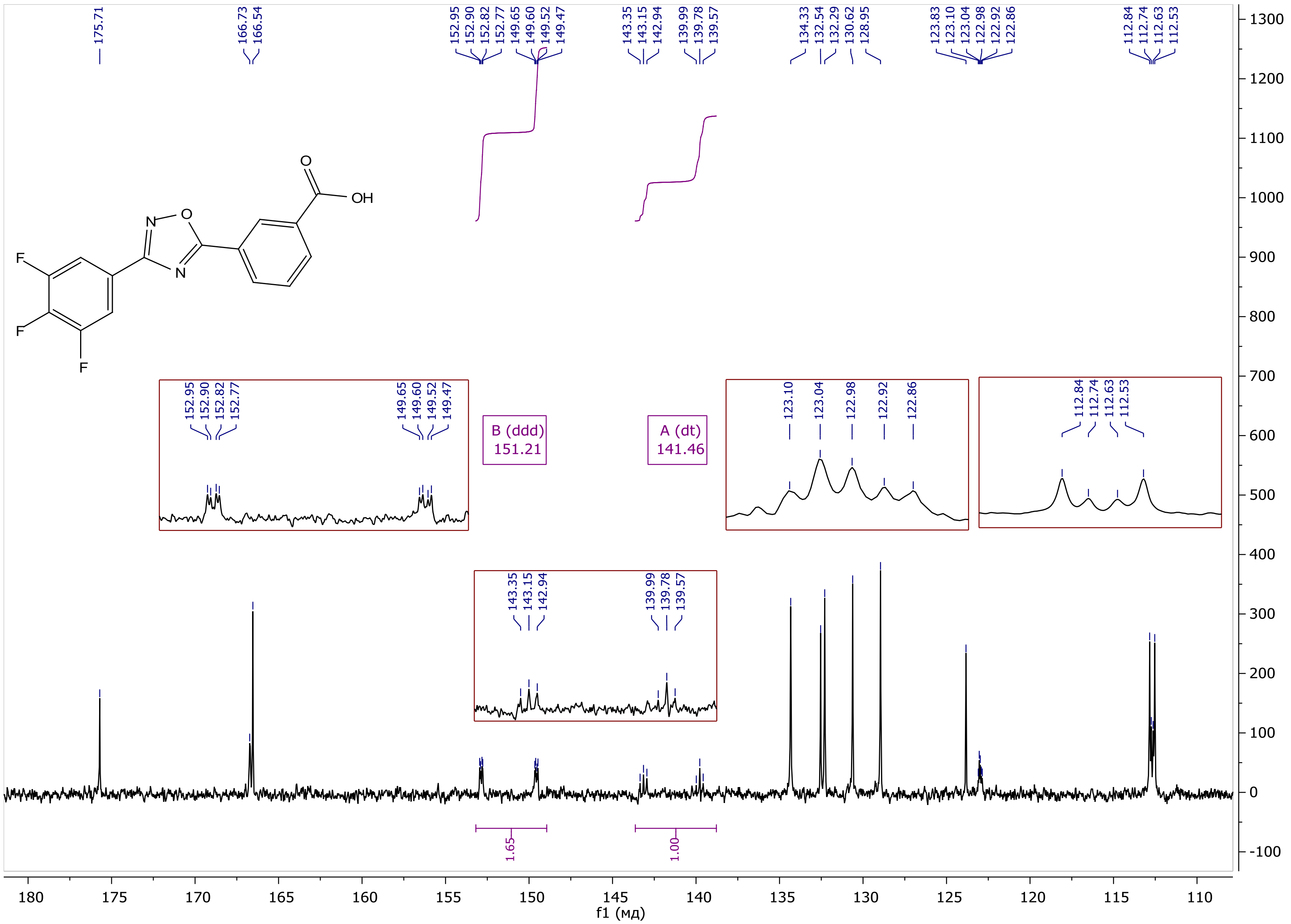

^{1}H, ^{13}C NMR spectra for 3-(3-aryl-1,2,4-oxadiazol-5-yl)propanoic acids synthesized

^{1}H NMR spectrum of compound **15a**

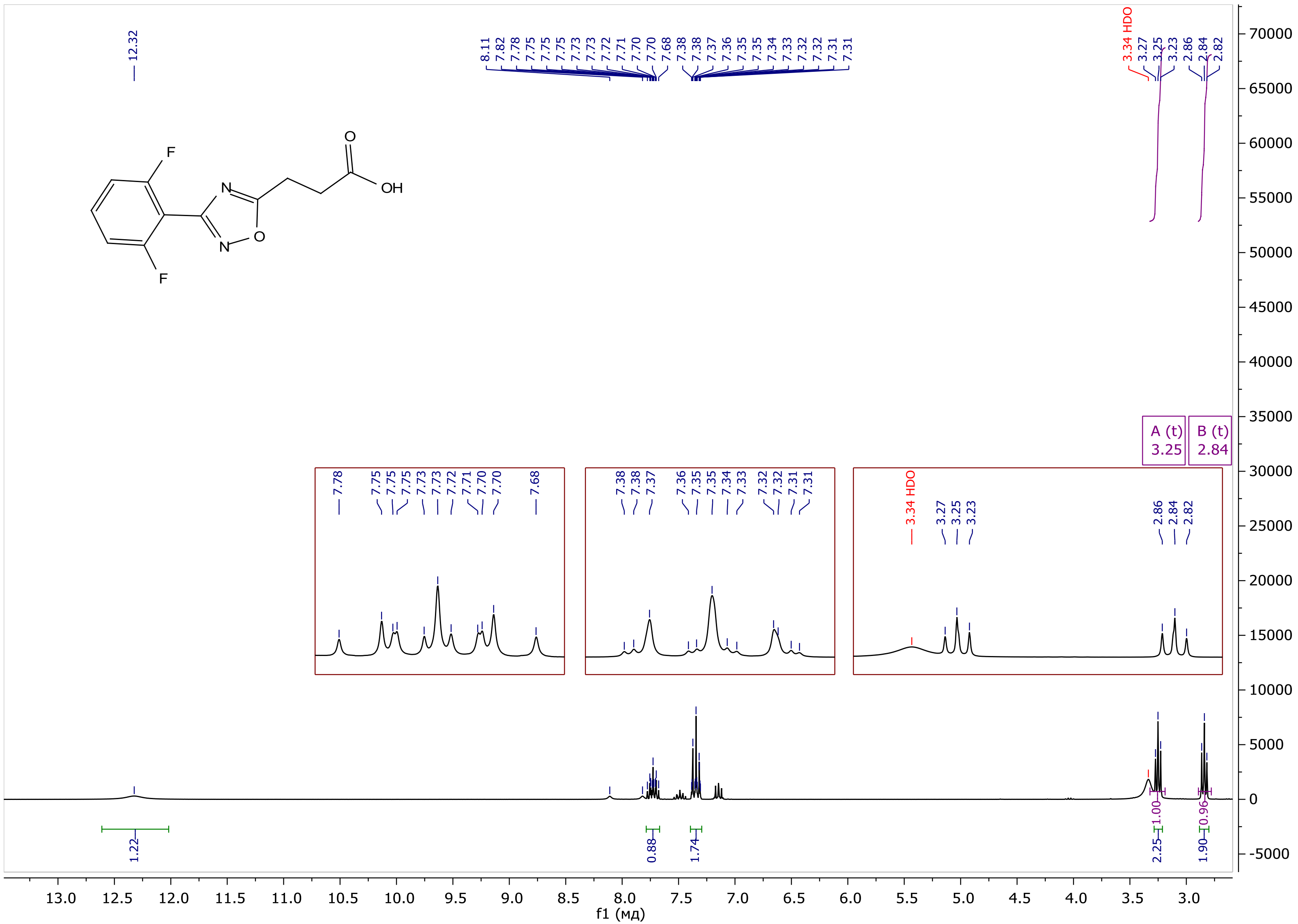

^{13}C NMR spectrum of compound **15a**

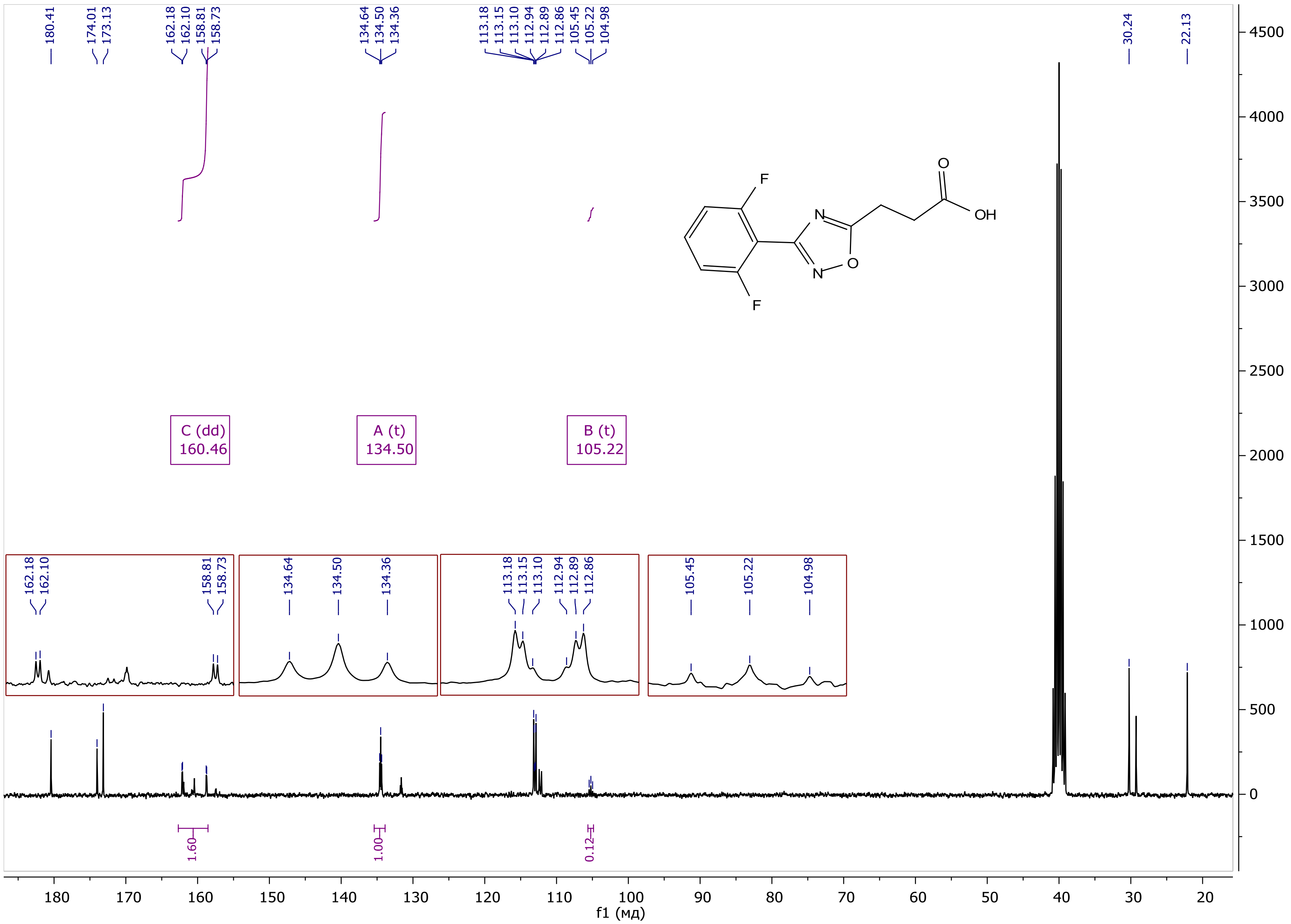

^{1}H NMR spectrum of compound **15b**

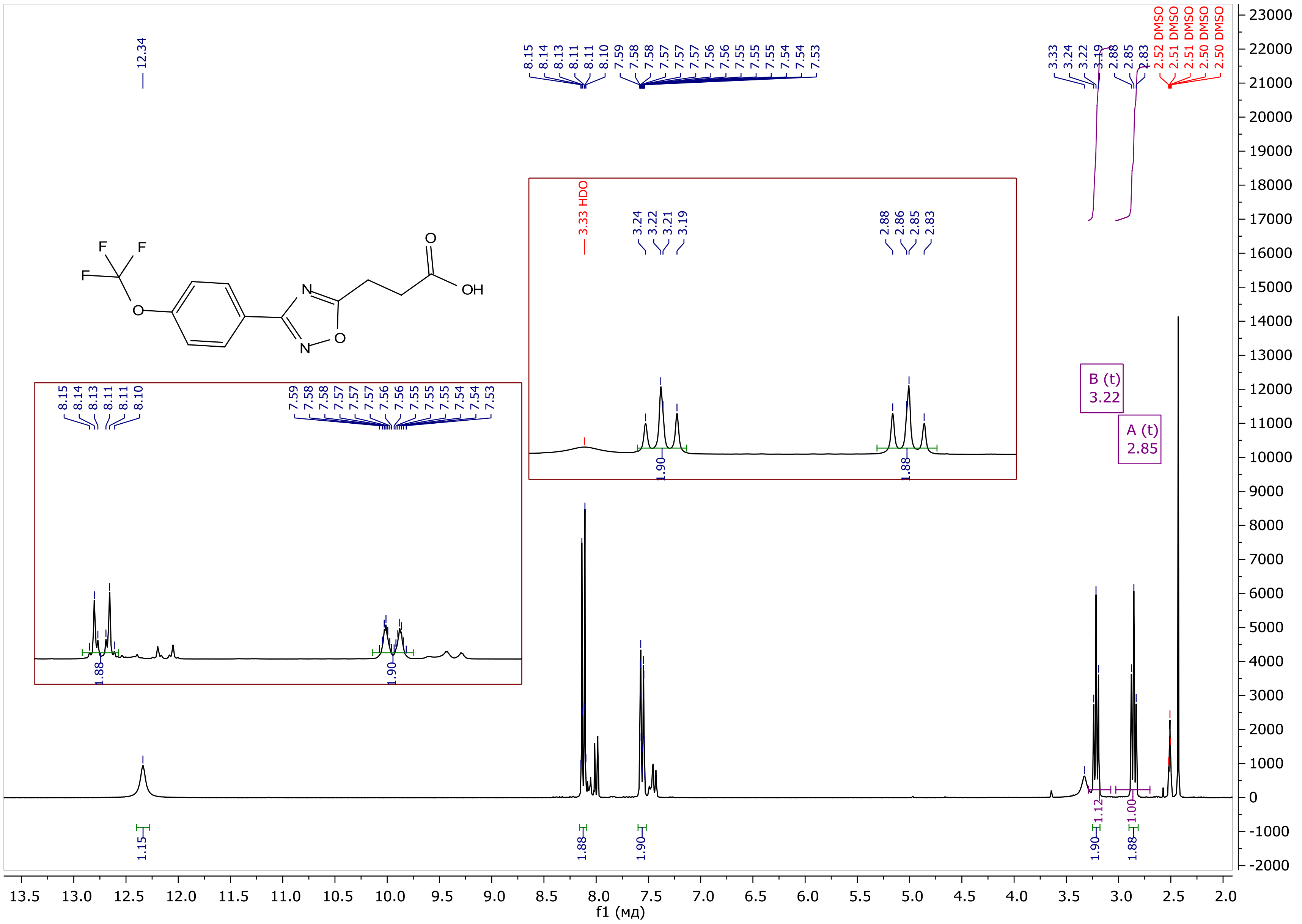

^{13}C NMR spectrum of compound **15b**

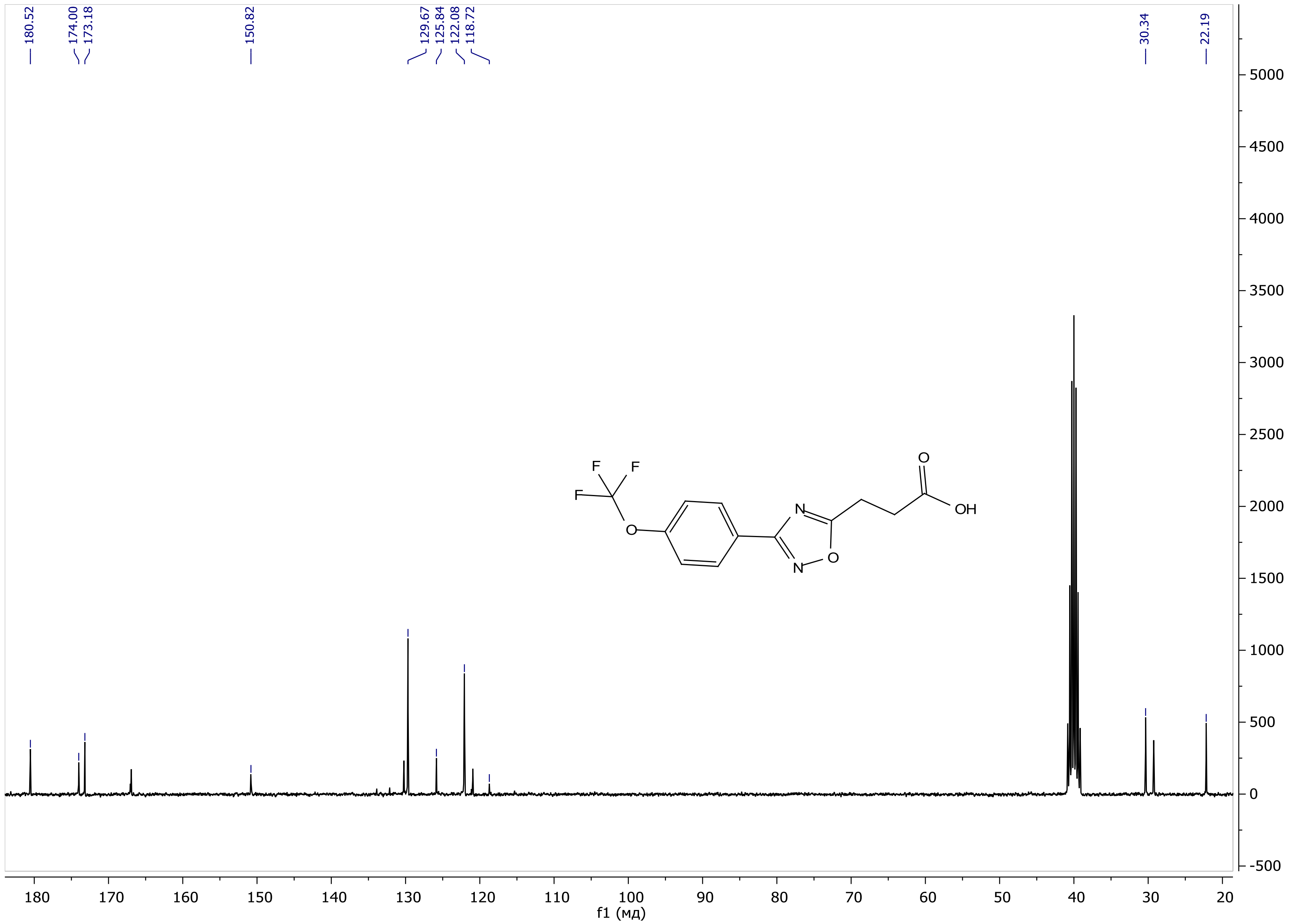

^{1}H NMR spectrum of compound **15c**

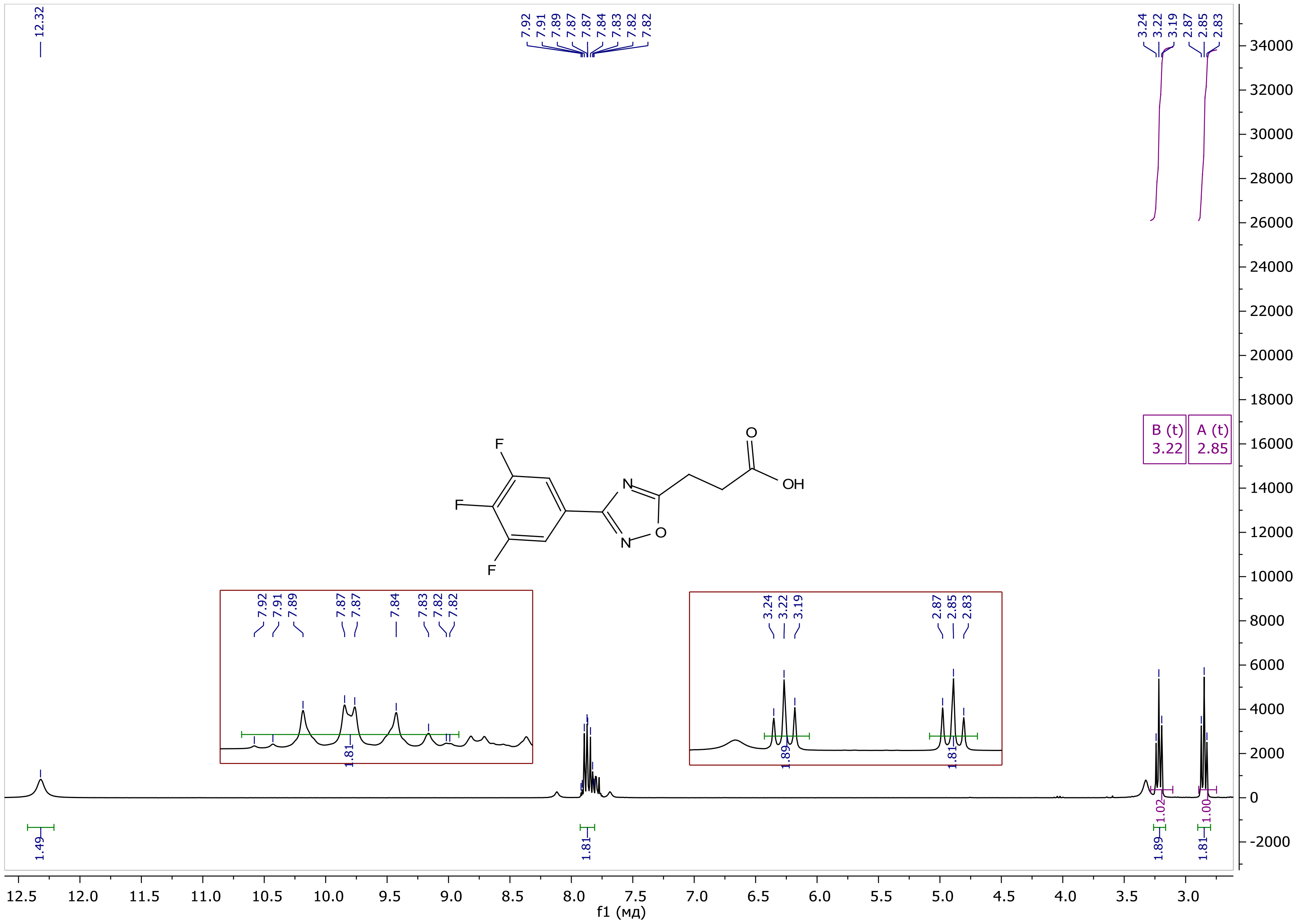

^{13}C NMR spectrum of compound **15c**

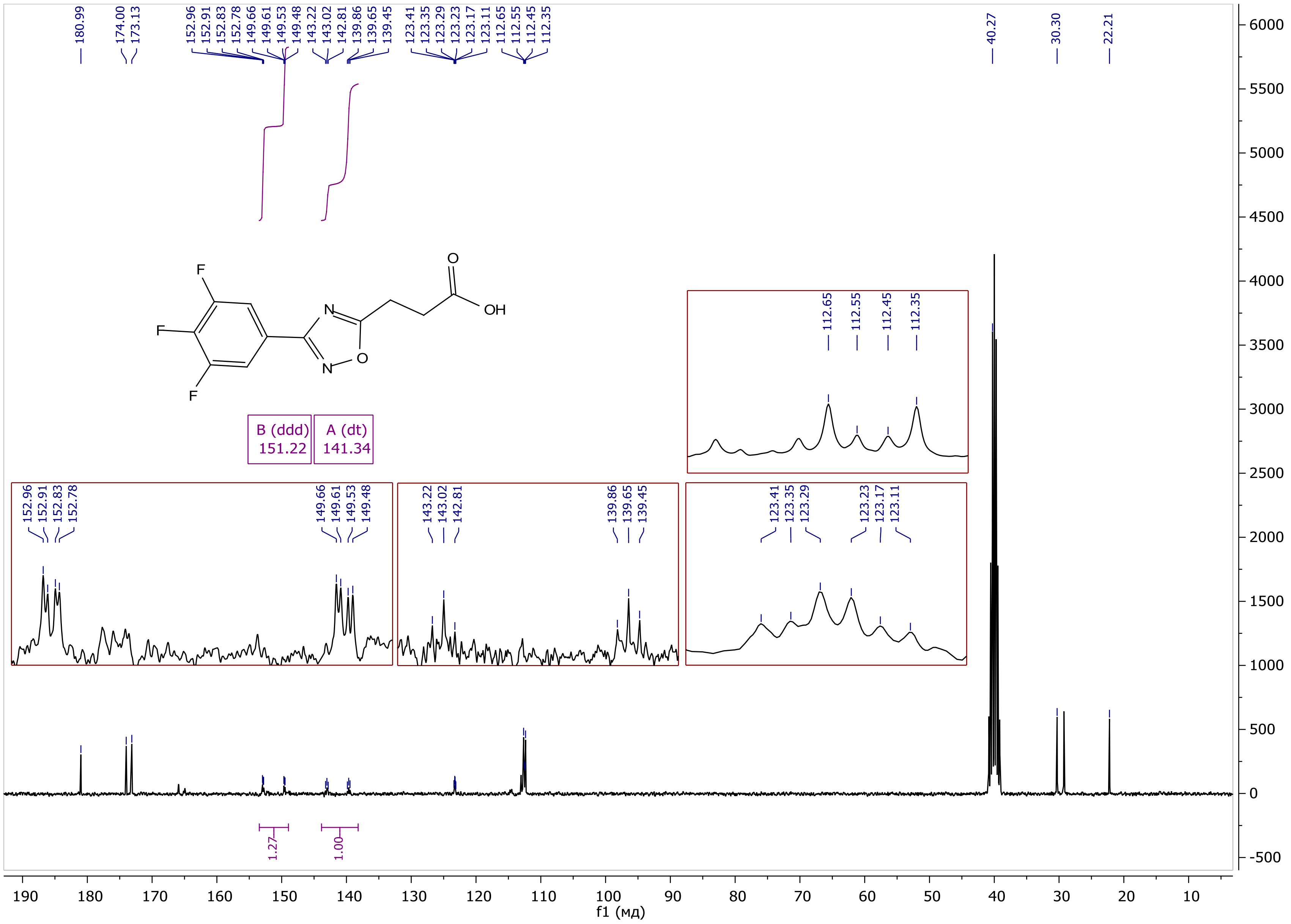

^{1}H NMR spectrum of compound **15d**

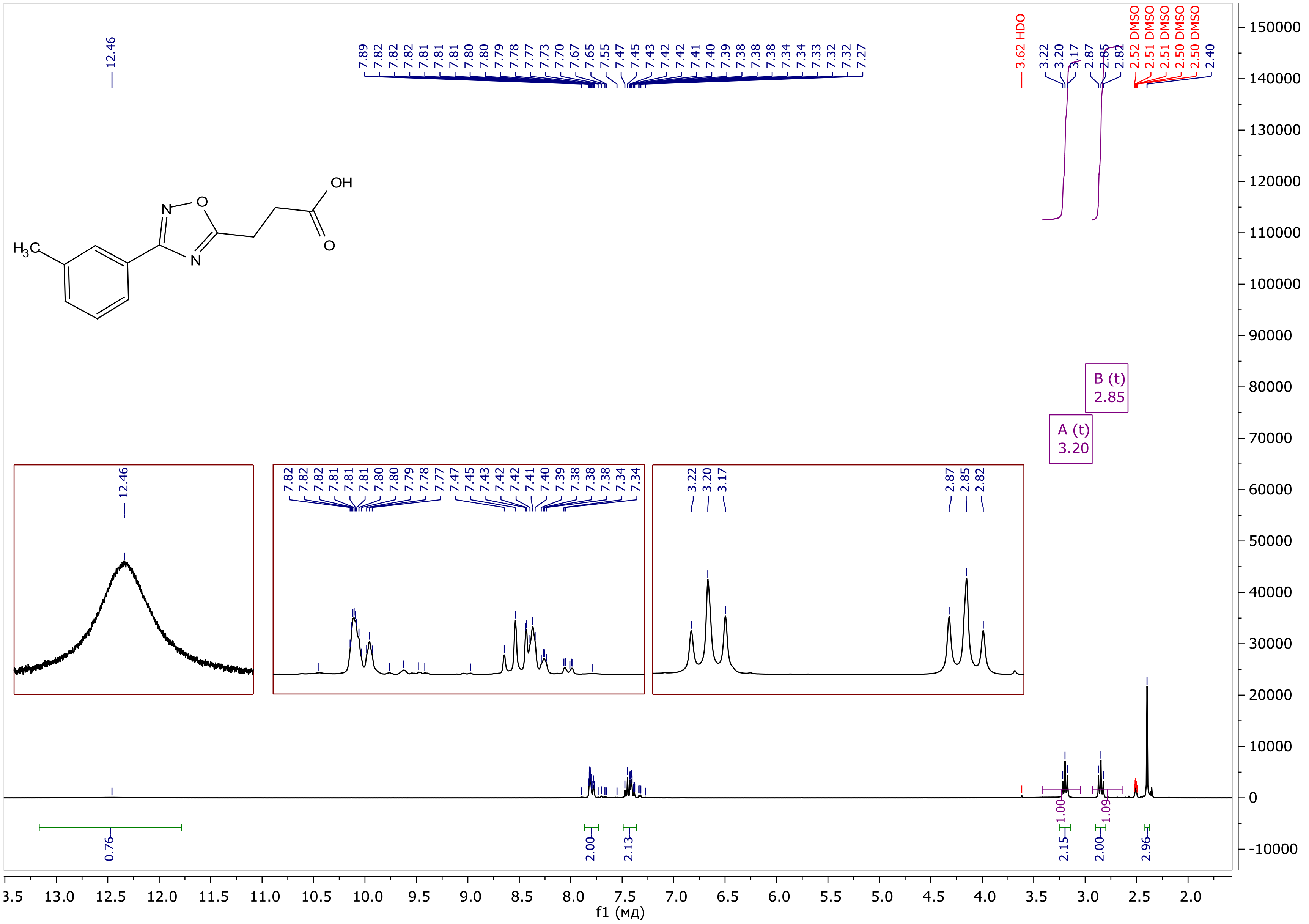

^{13}C NMR spectrum of compound **15d**

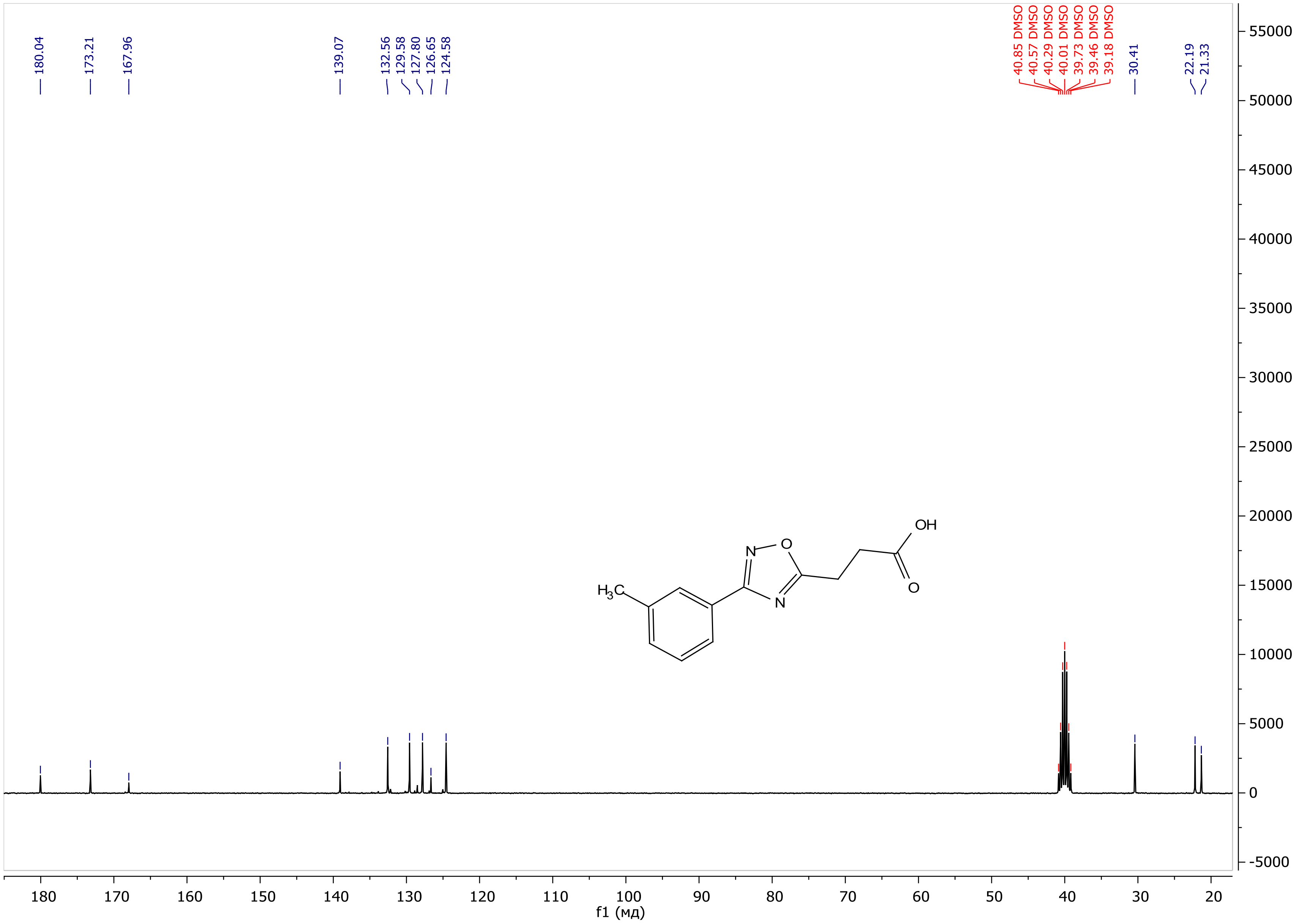

^{1}H, ^{13}C NMR spectra for methyl 3-(3-aryl-1,2,4-oxadiazol-5-yl)propanoates synthesized

^{1}H NMR spectrum of compound **16a**

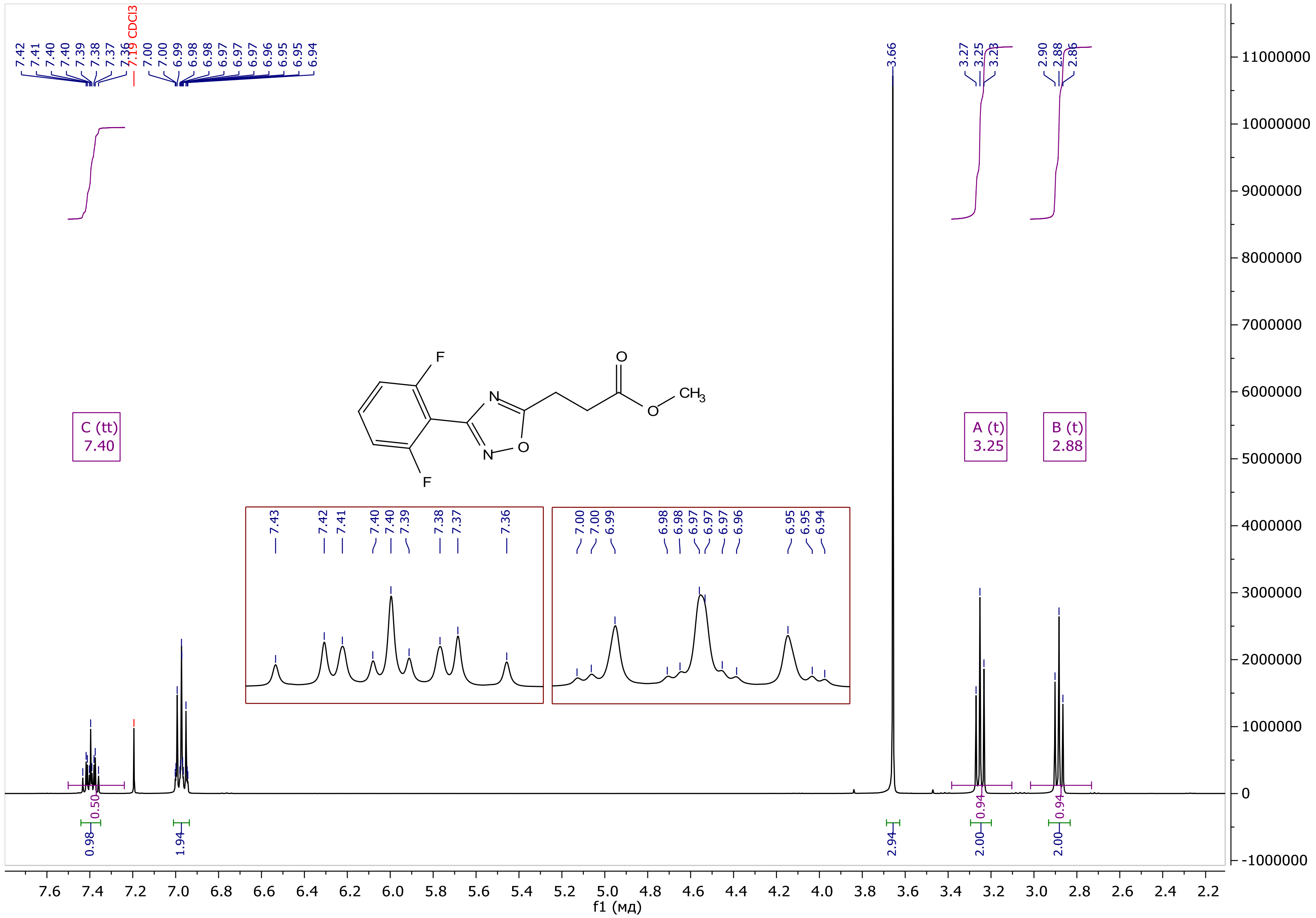

^{13}C NMR spectrum of compound **16a**

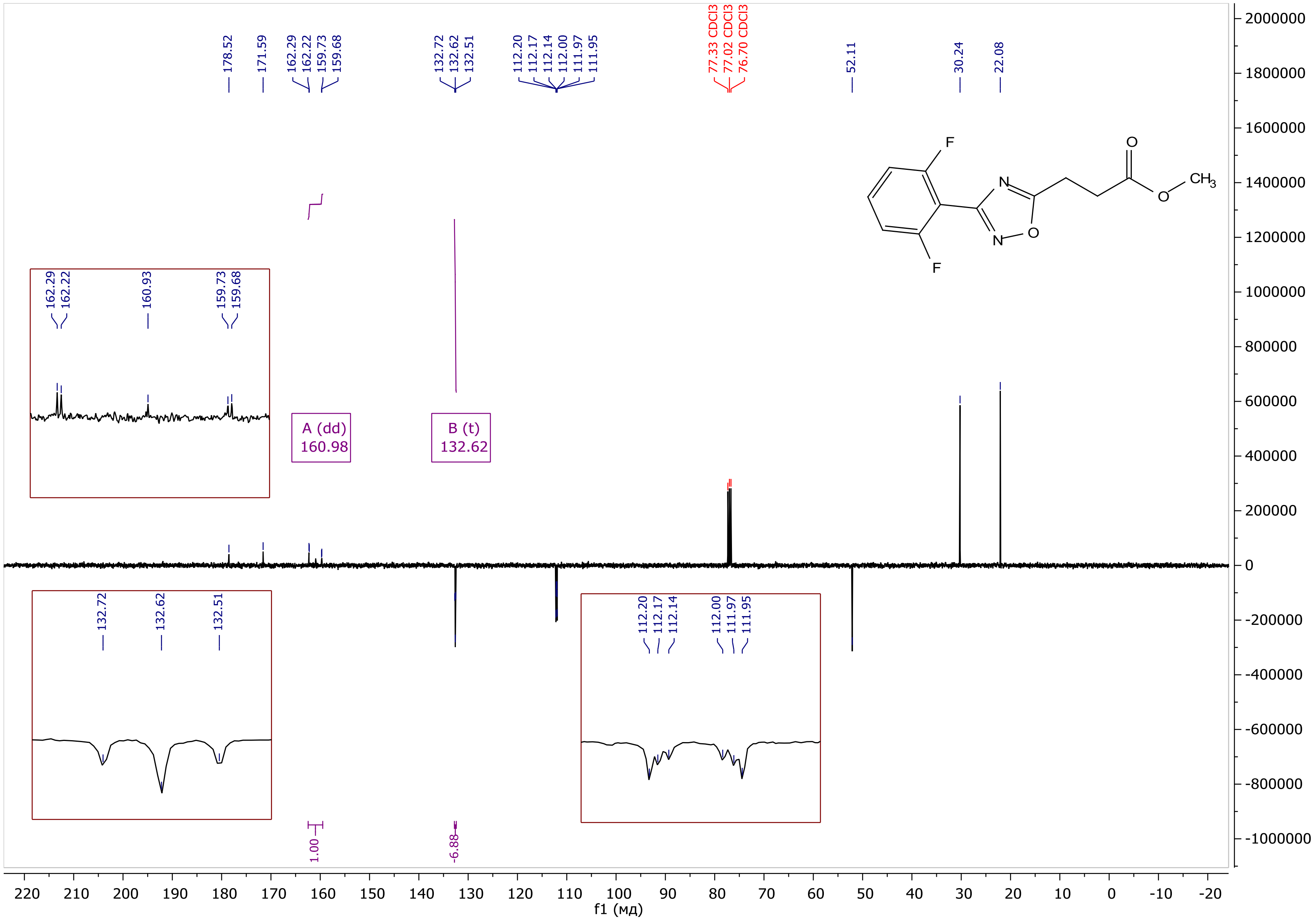

^{1}H NMR spectrum of compound **16b**

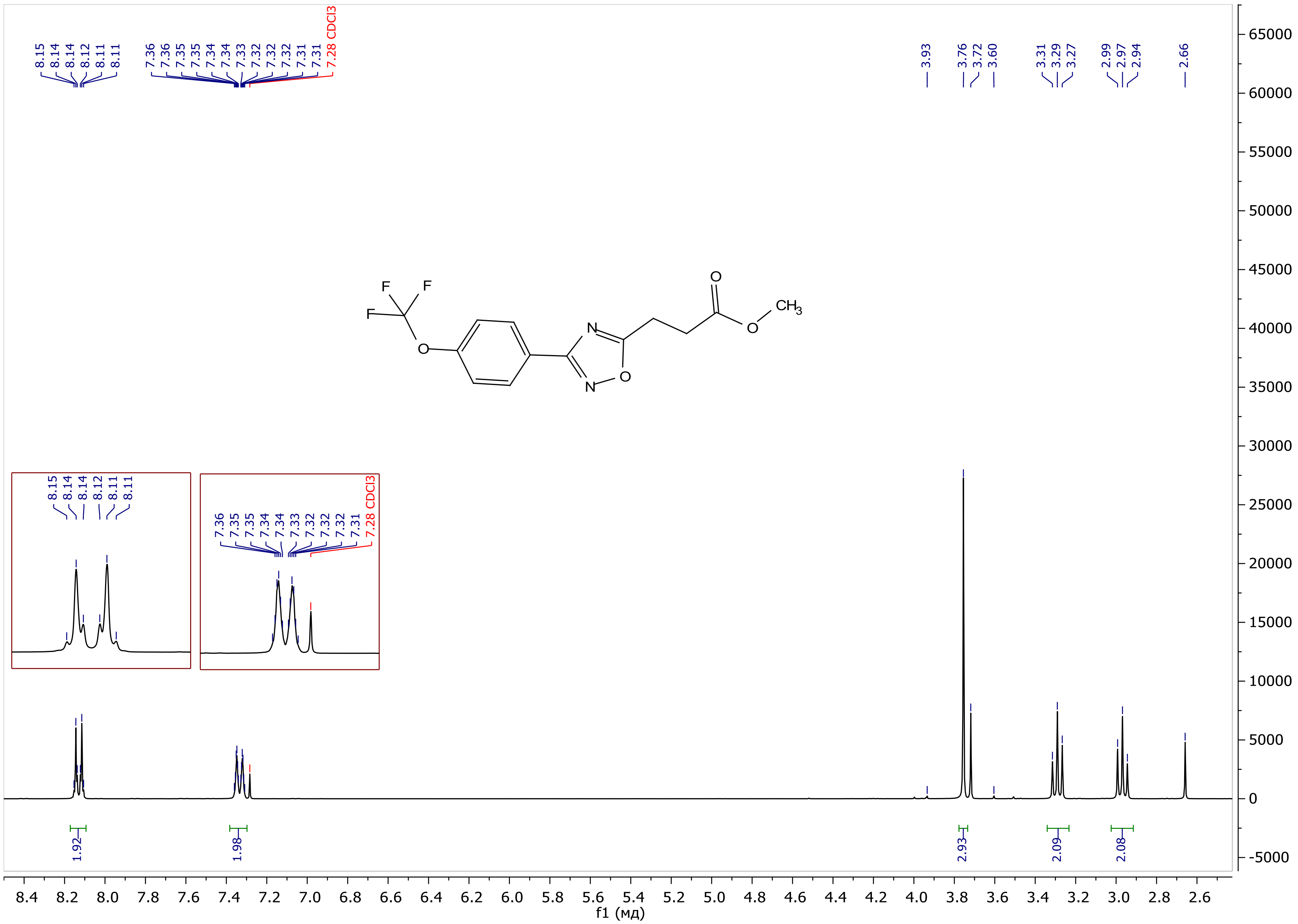

^{13}C NMR spectrum of compound **16b**

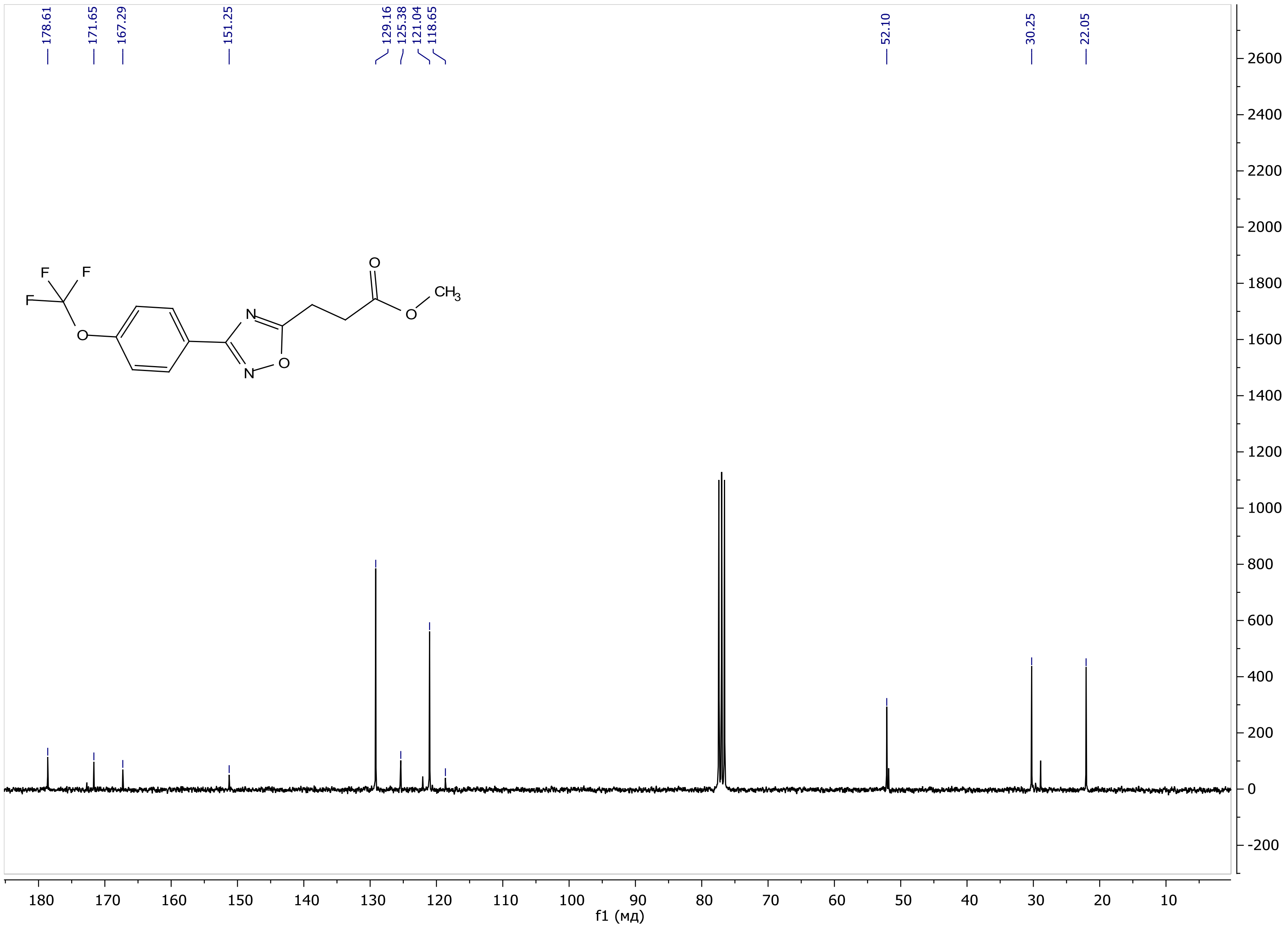

^{1}H NMR spectrum of compound **16c**

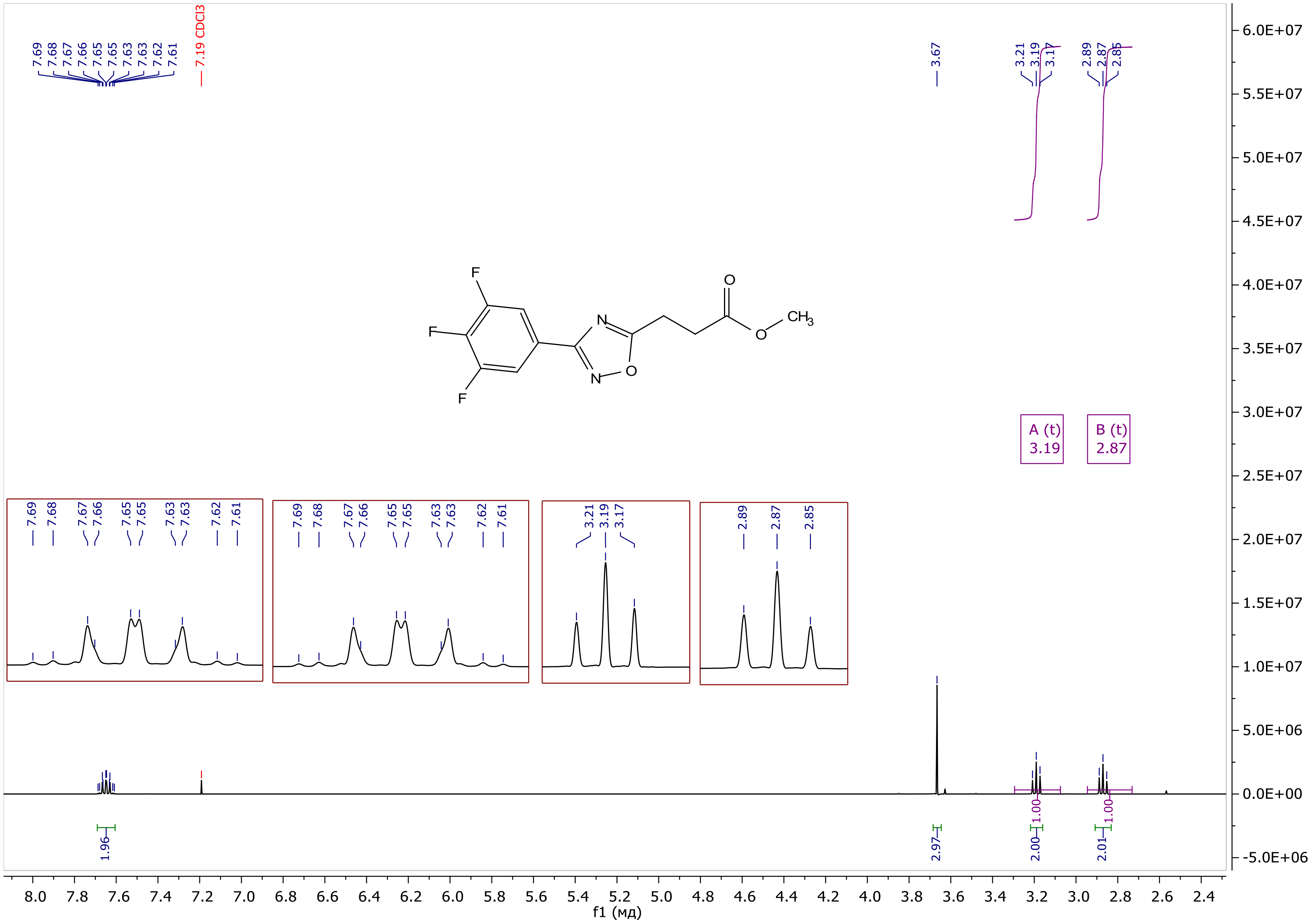

^{13}C NMR spectrum of compound **16c**

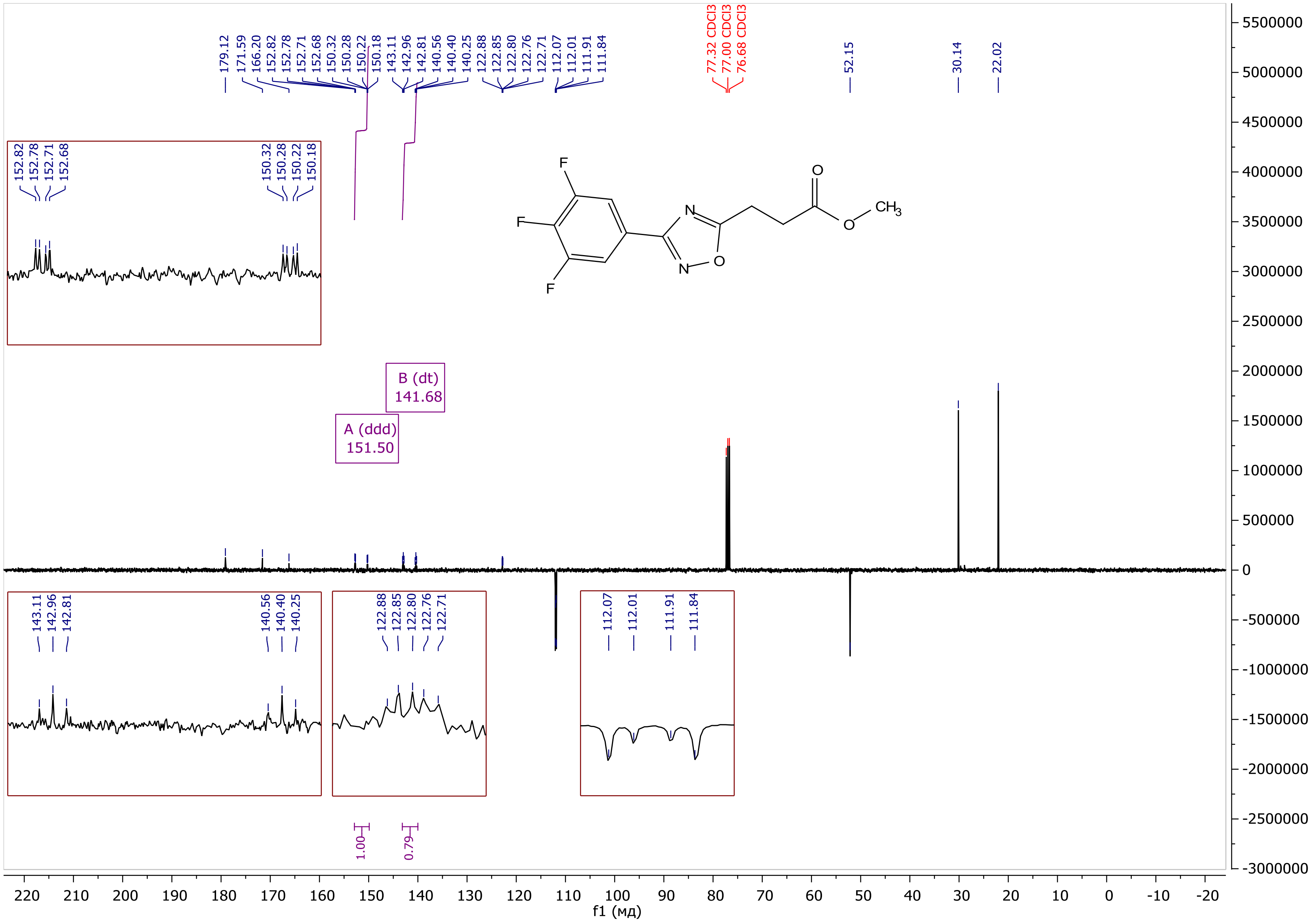

^{1}H, ^{13}C NMR spectra for 3-aryl-*N*-hydroxy-1,2,4-oxadiazole-5-carboxamides synthesized

^{1}H NMR spectrum of compound **4a**

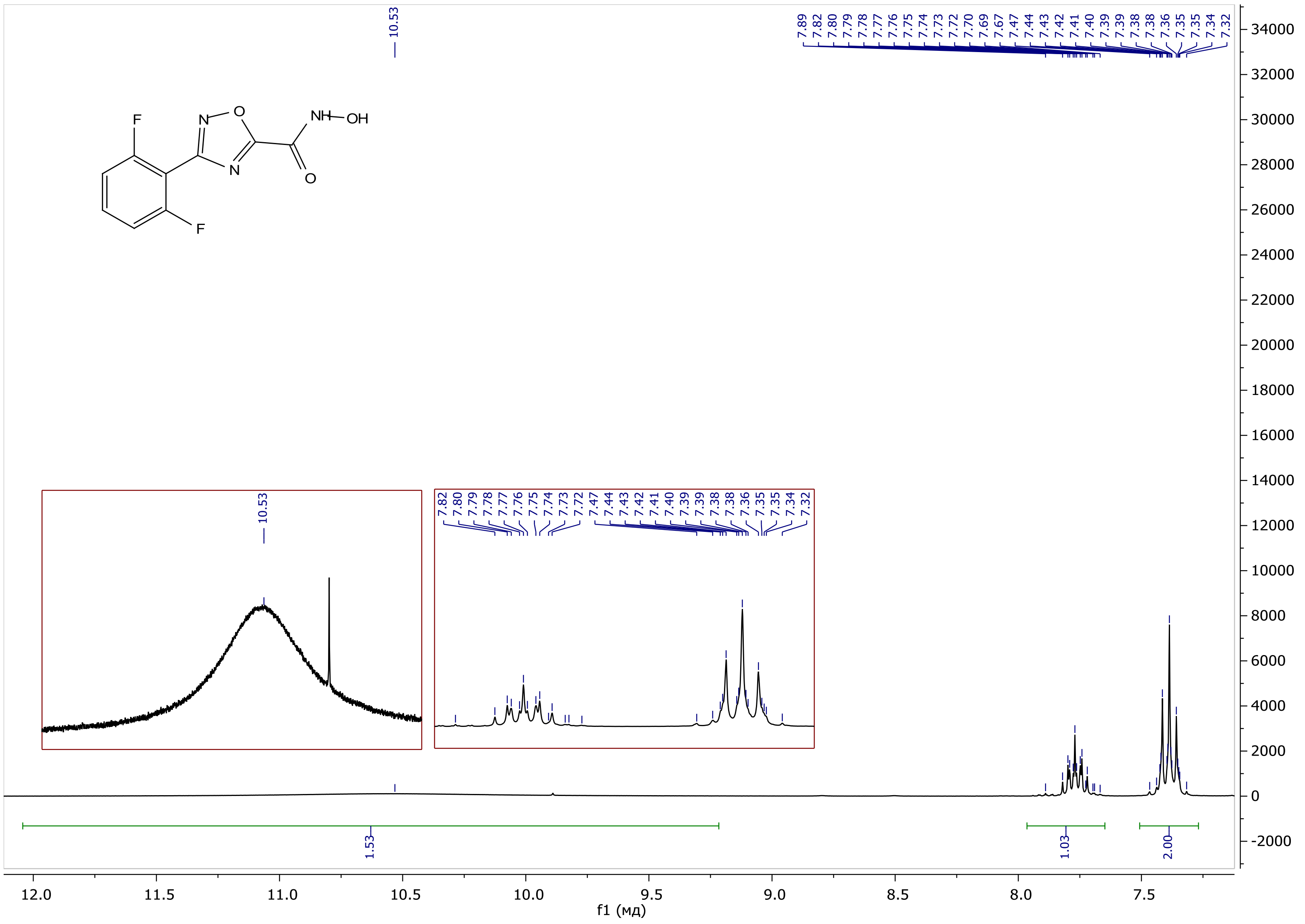

^{13}C NMR spectrum of compound **4a**

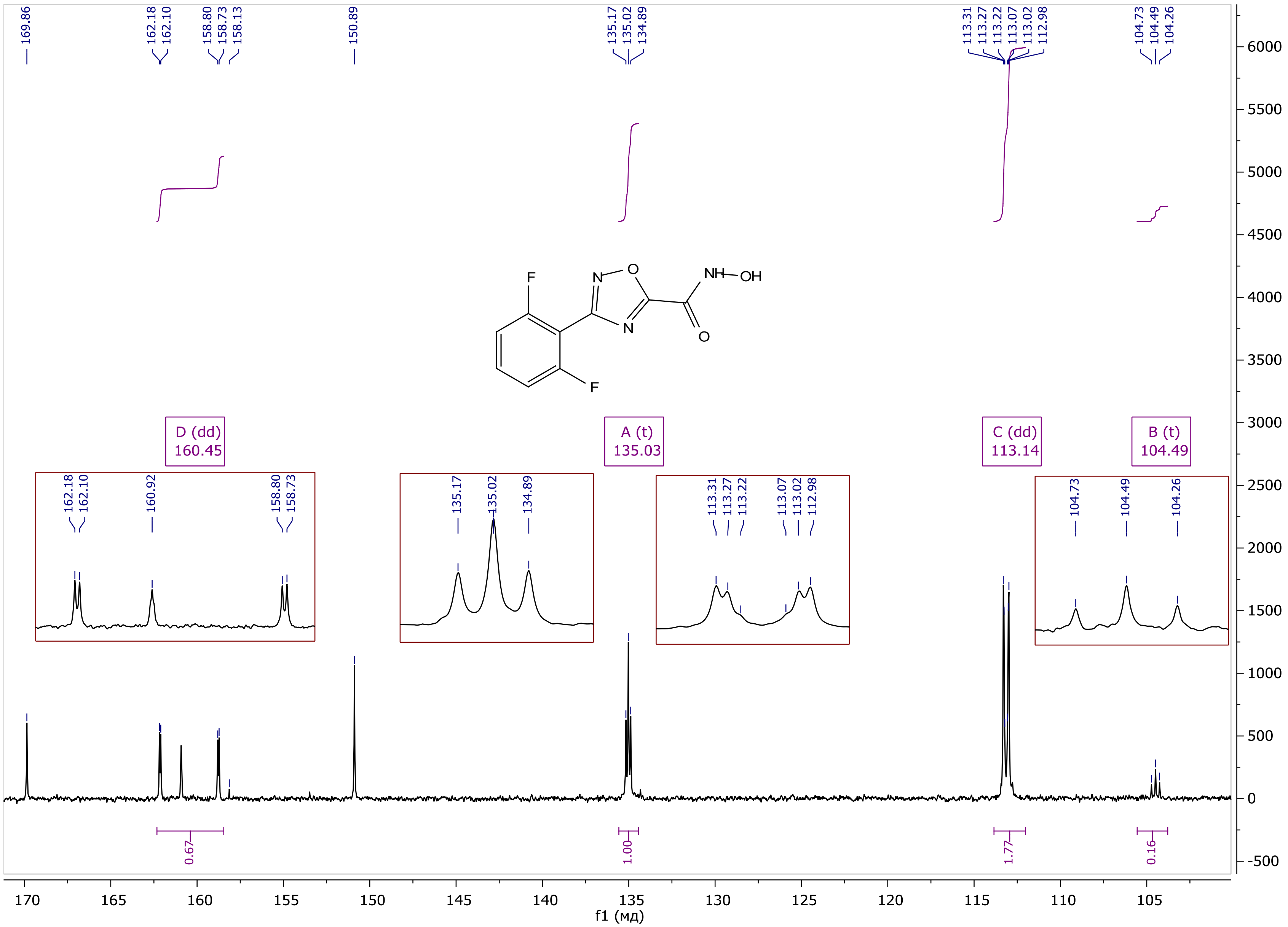

^{1}H NMR spectrum of compound **4b**

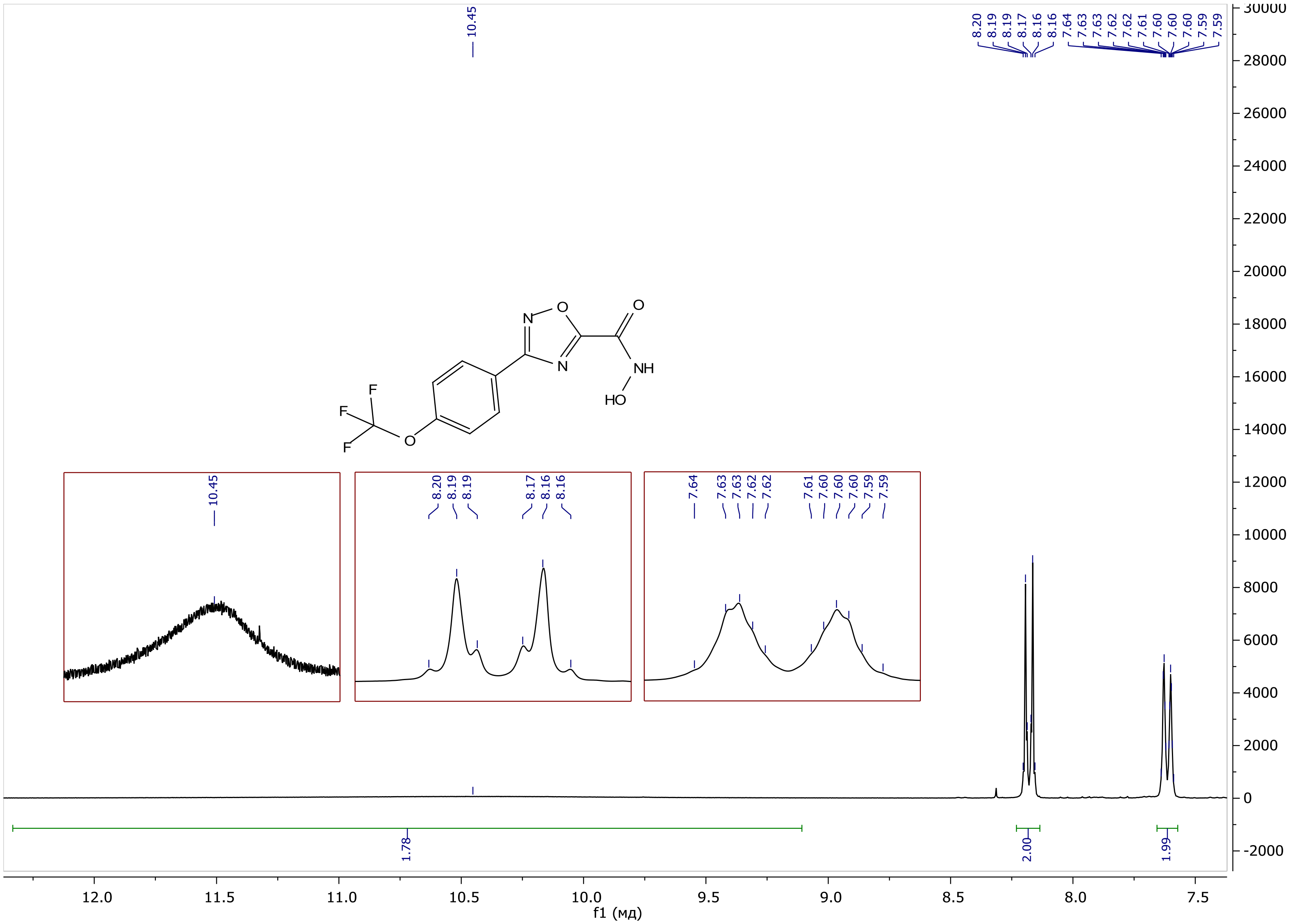

^{13}C NMR spectrum of compound **4b**

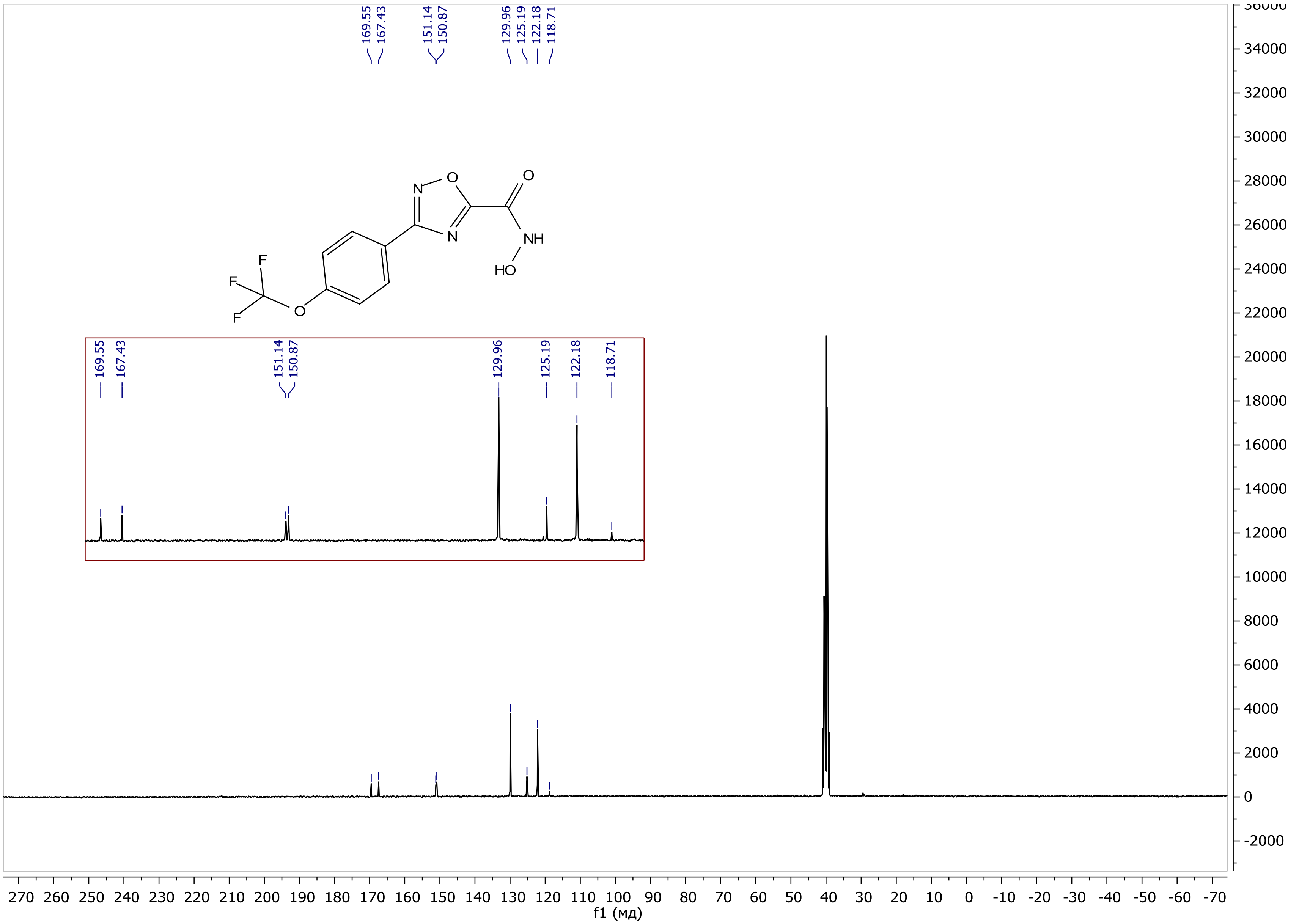

^{1}H NMR spectrum of compound **4c**

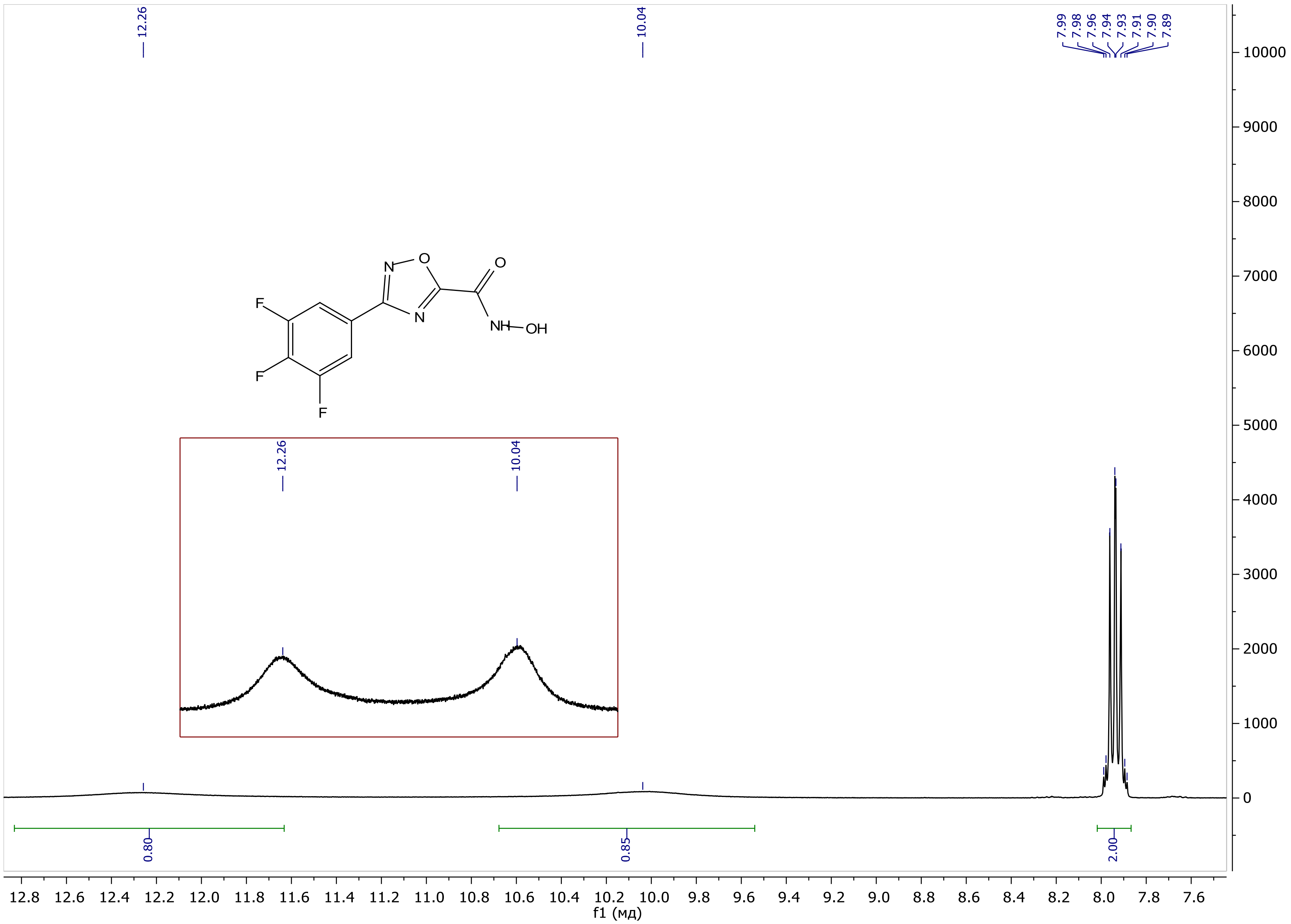

^{13}C NMR spectrum of compound **4c**

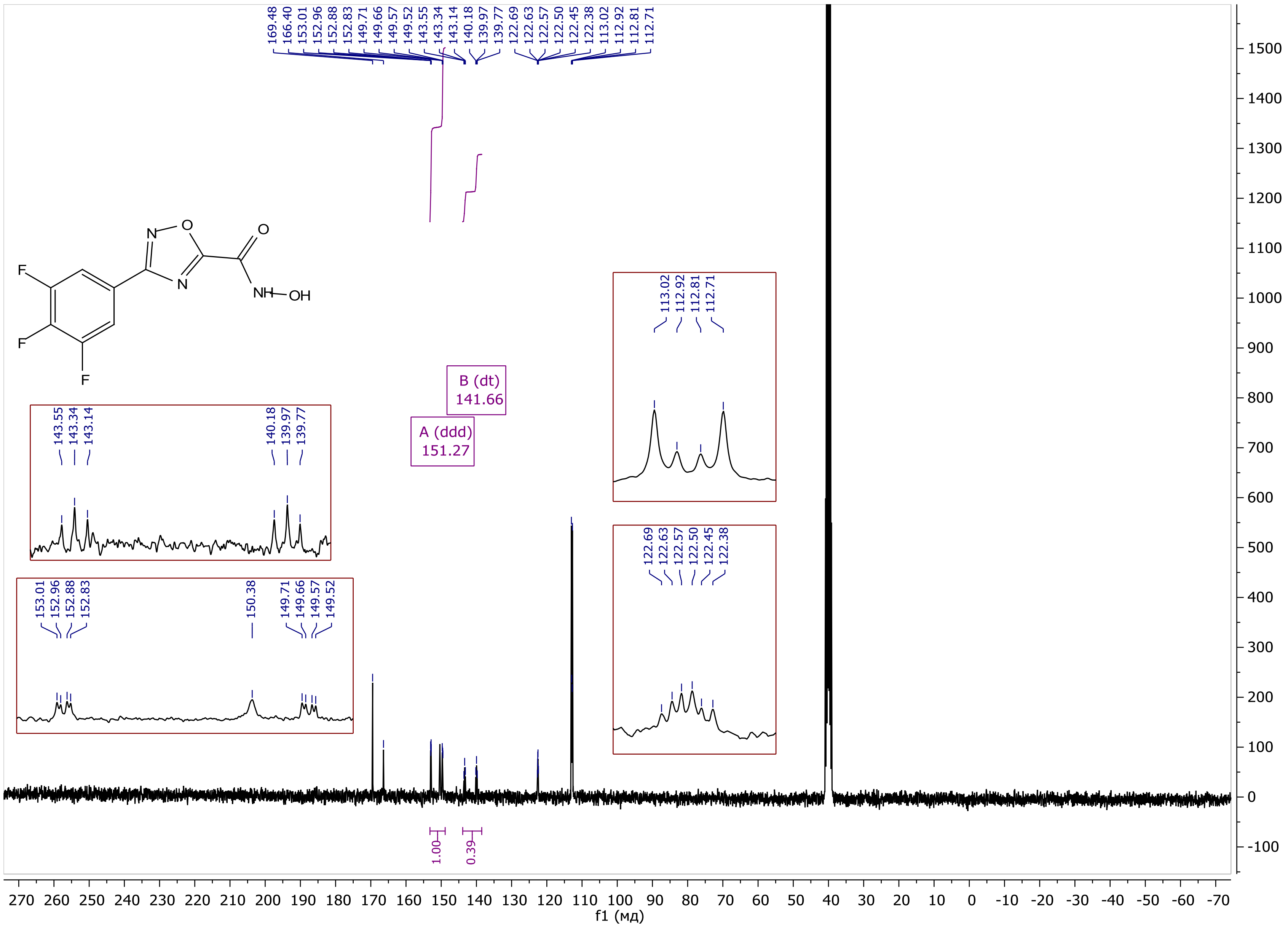

^{1}H NMR spectrum of compound **4d**

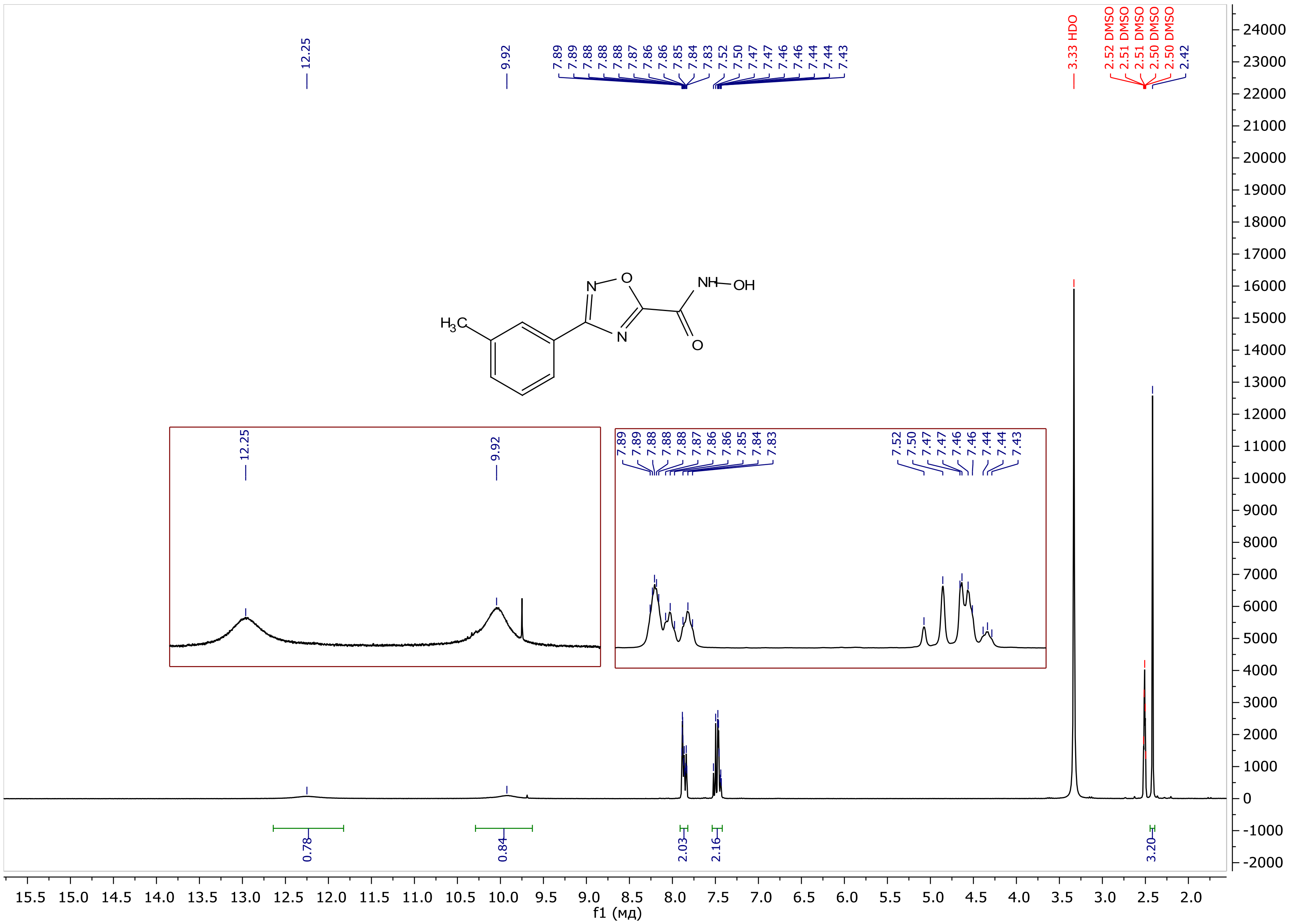

^{13}C NMR spectrum of compound **4d**

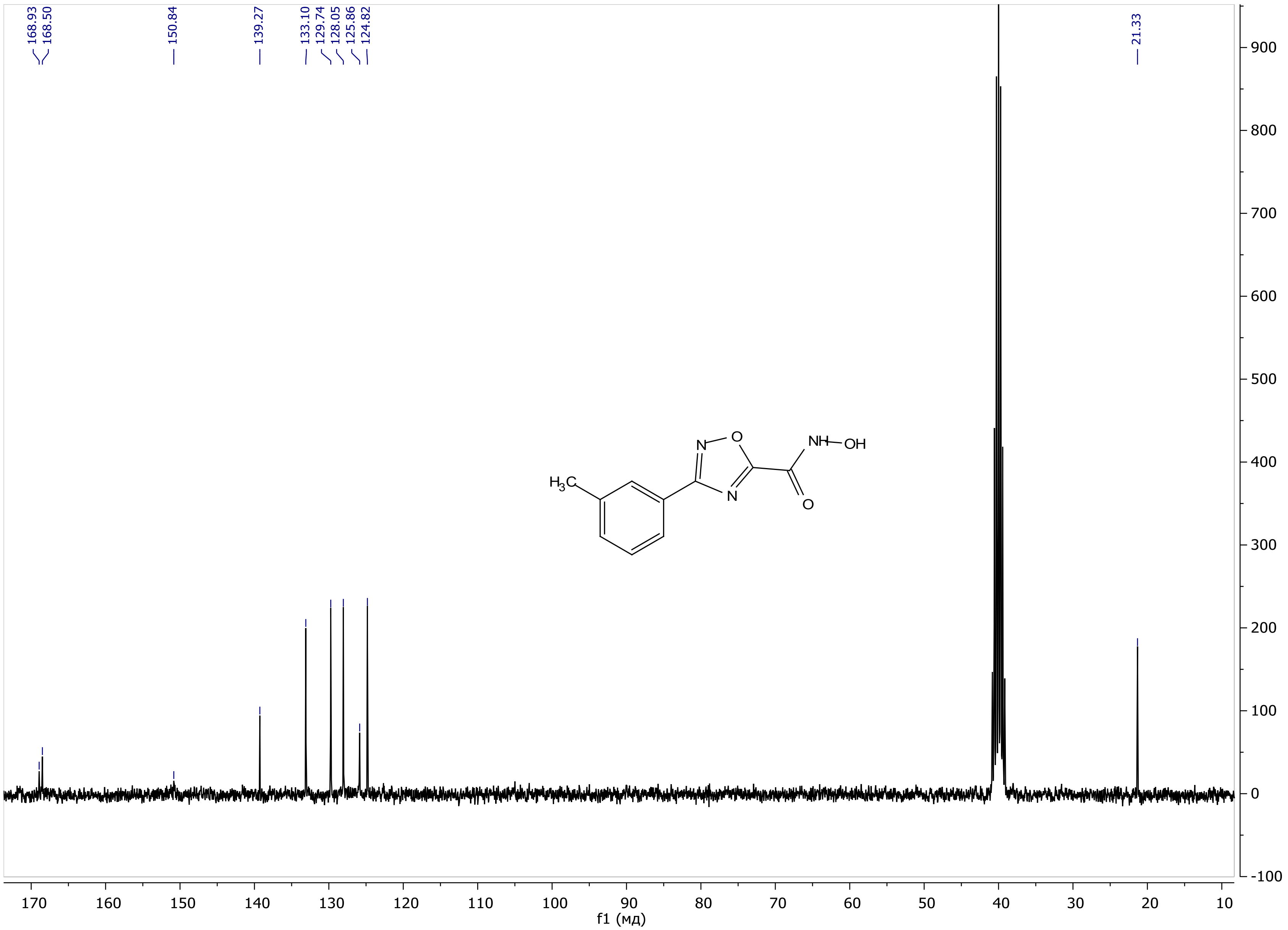

^{1}H NMR spectrum of compound **4e**

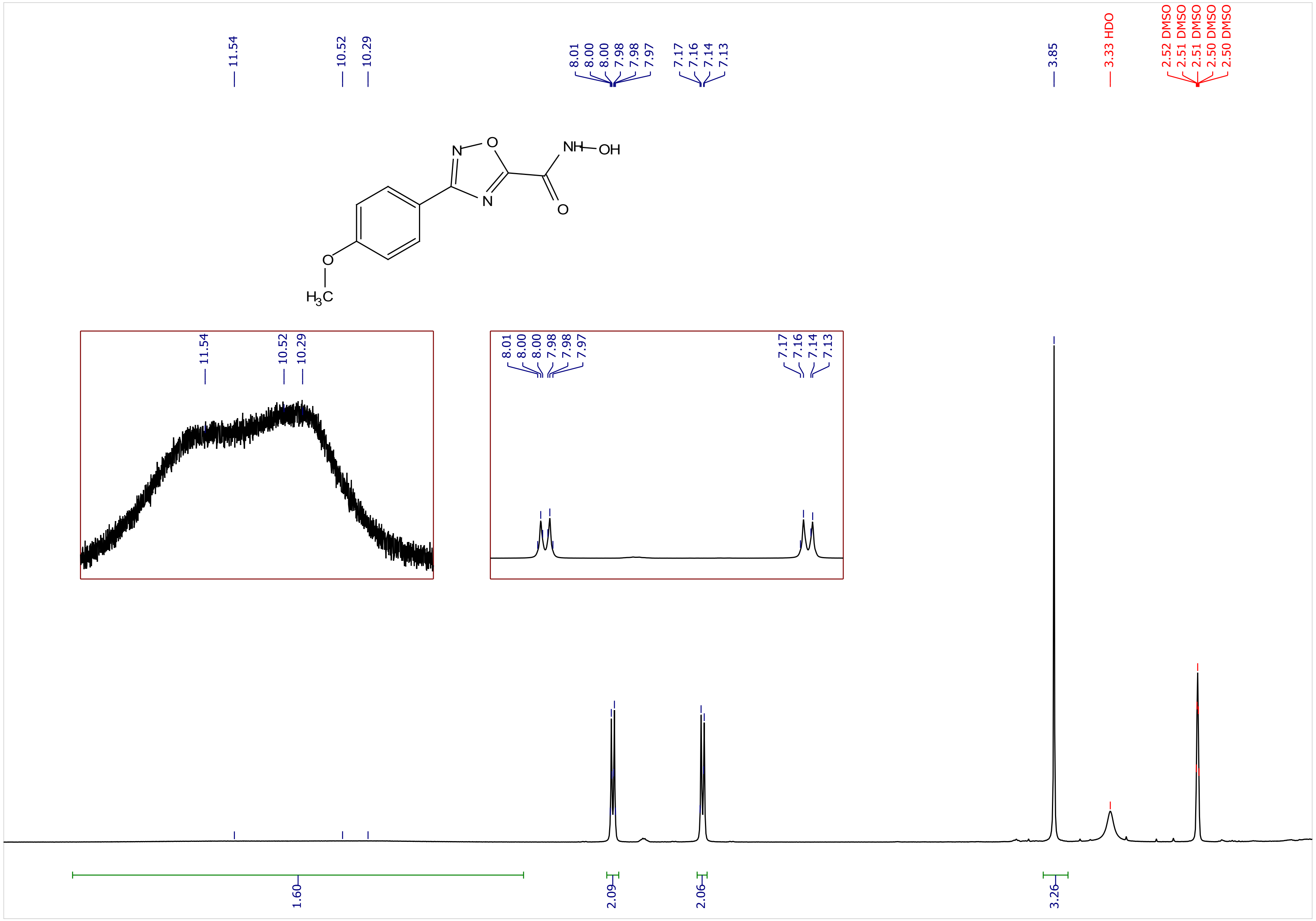

^{13}C NMR spectrum of compound **4e**

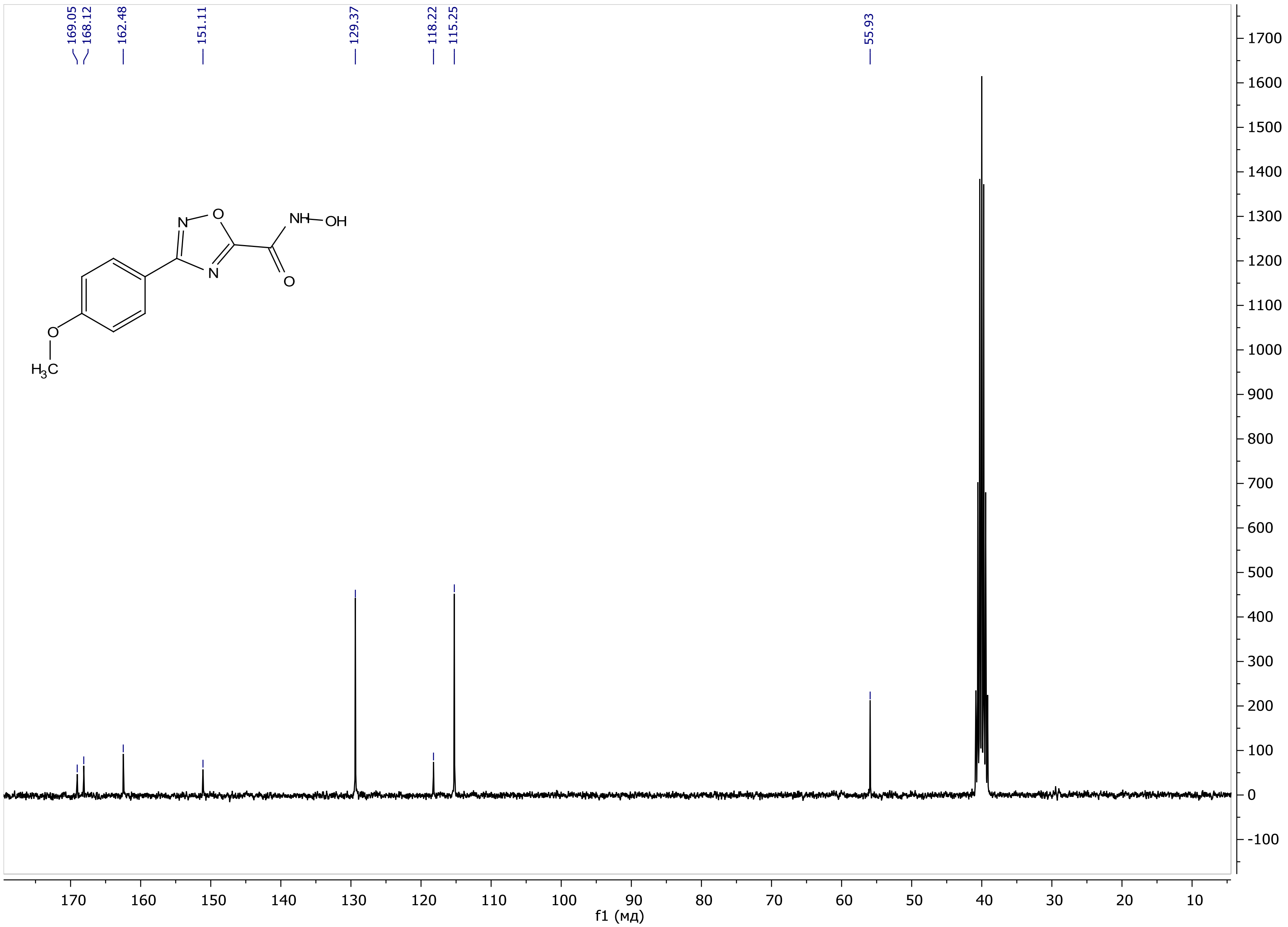

^{1}H NMR spectrum of compound **4f**

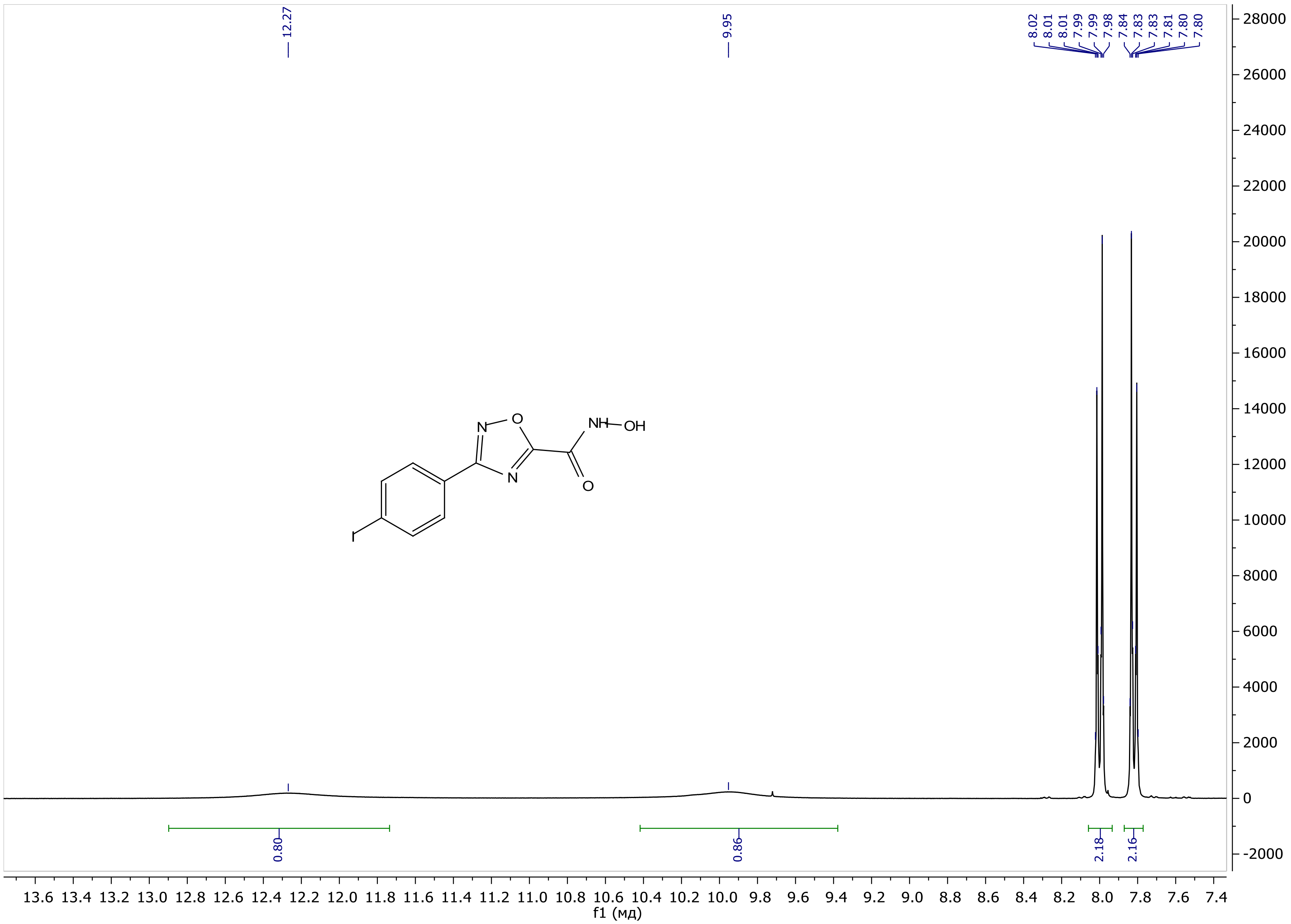

^{13}C NMR spectrum of compound **4f**

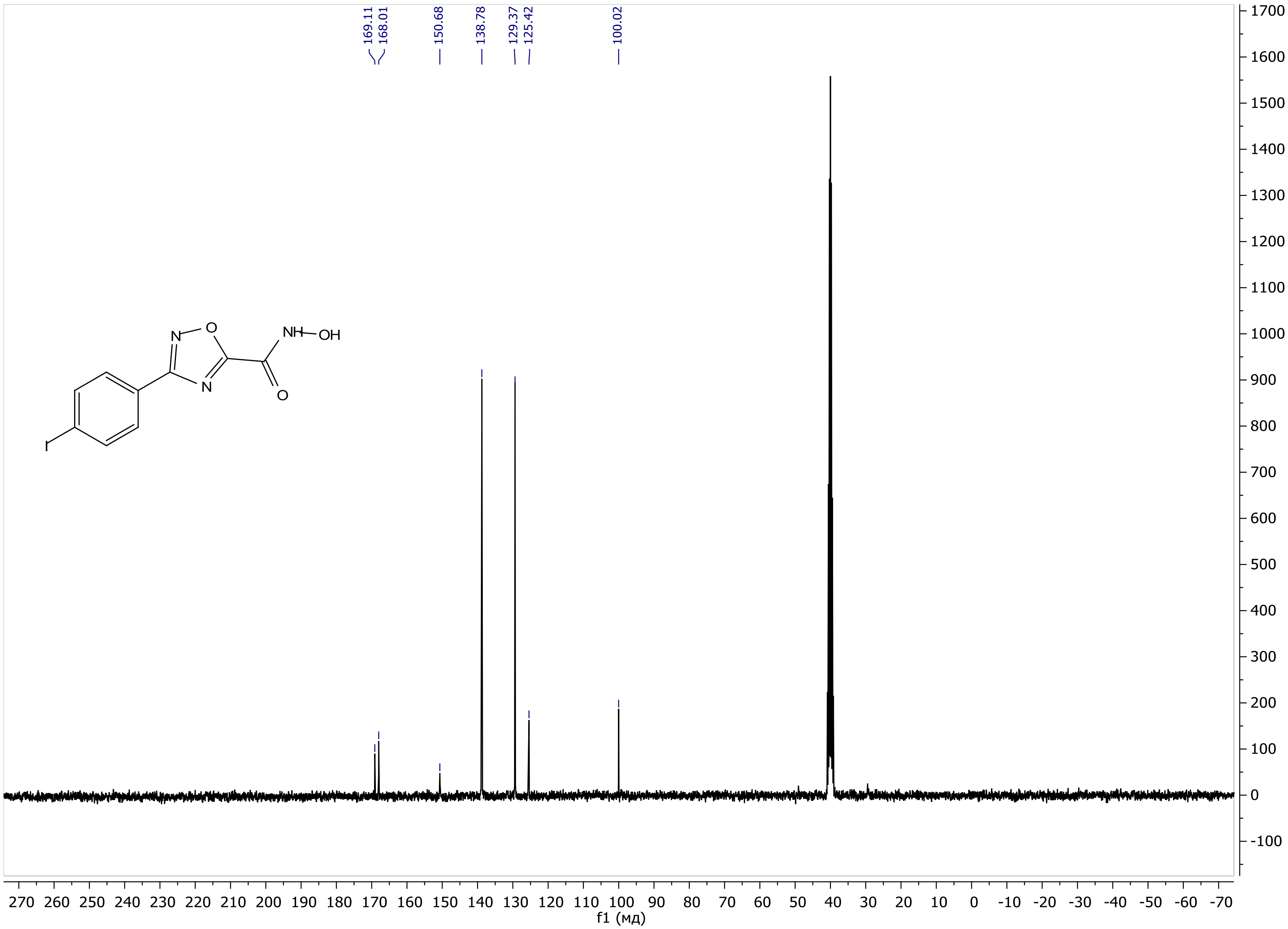

^{1}H, ^{13}C NMR spectra for 2-(3-aryl-1,2,4-oxadiazol-5-yl)-*N*-hydroxyacetamides synthesized

^{1}H NMR spectrum of compound **9a**

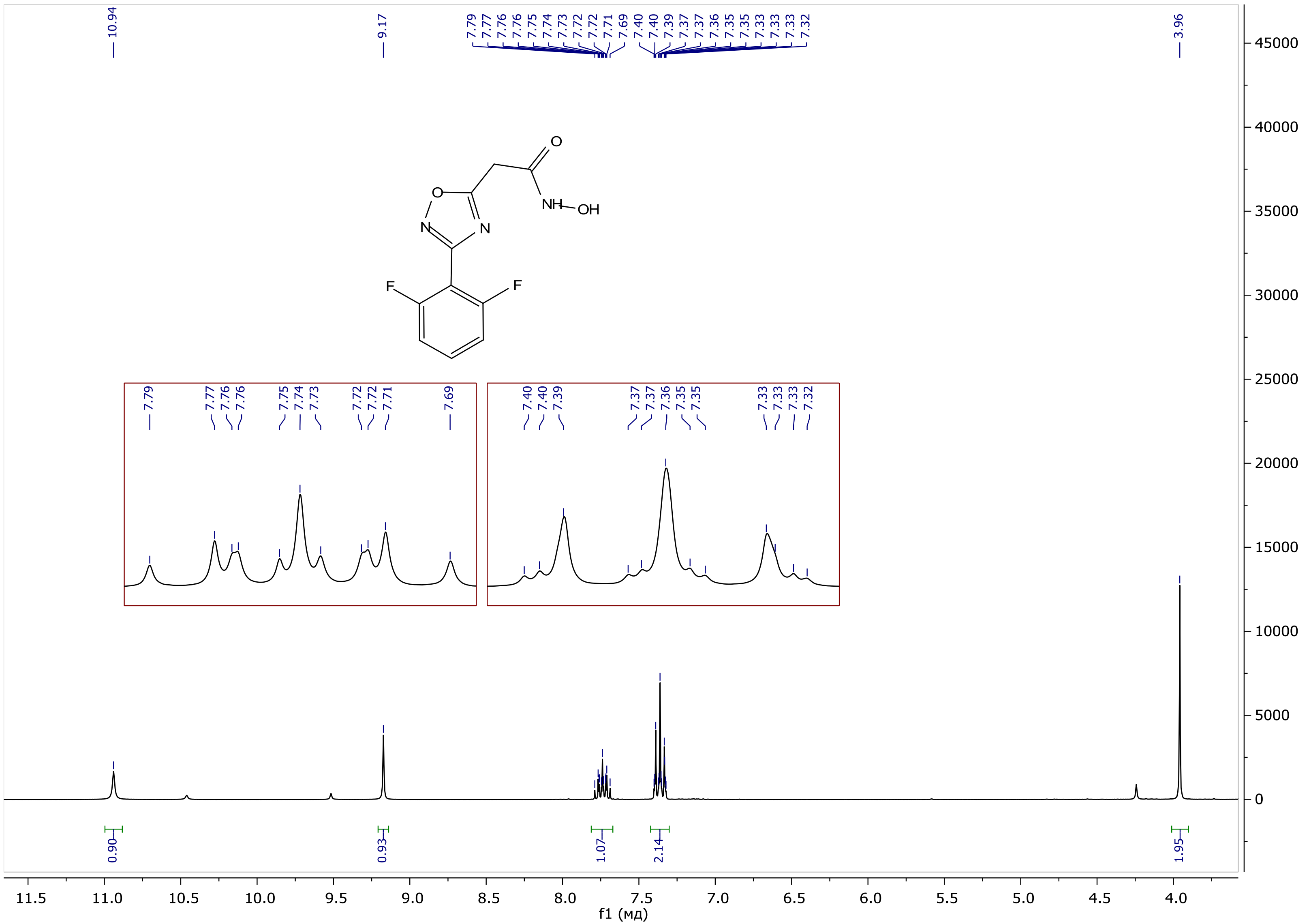

^{13}C NMR spectrum of compound **9a**

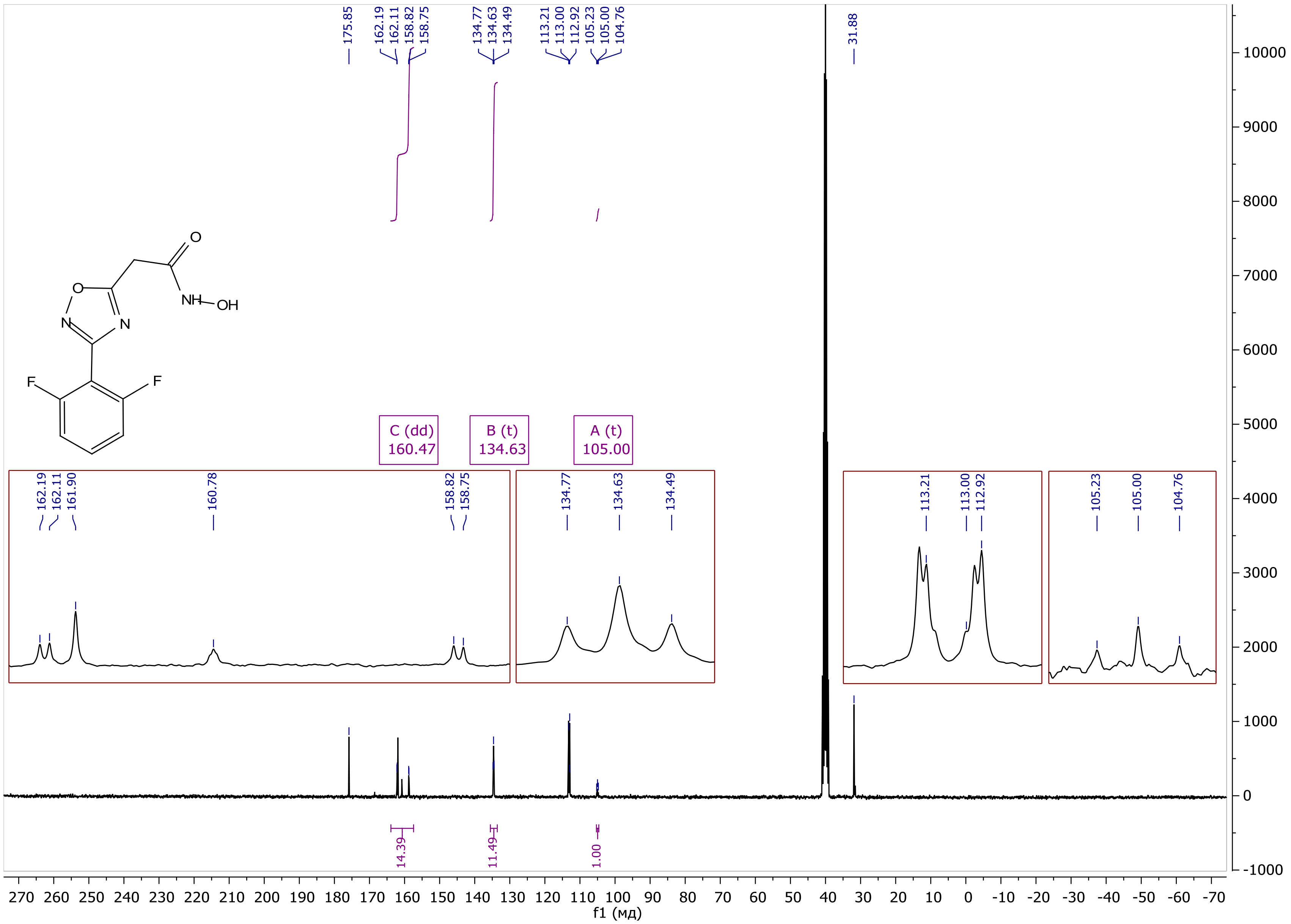

^{1}H NMR spectrum of compound **9b**

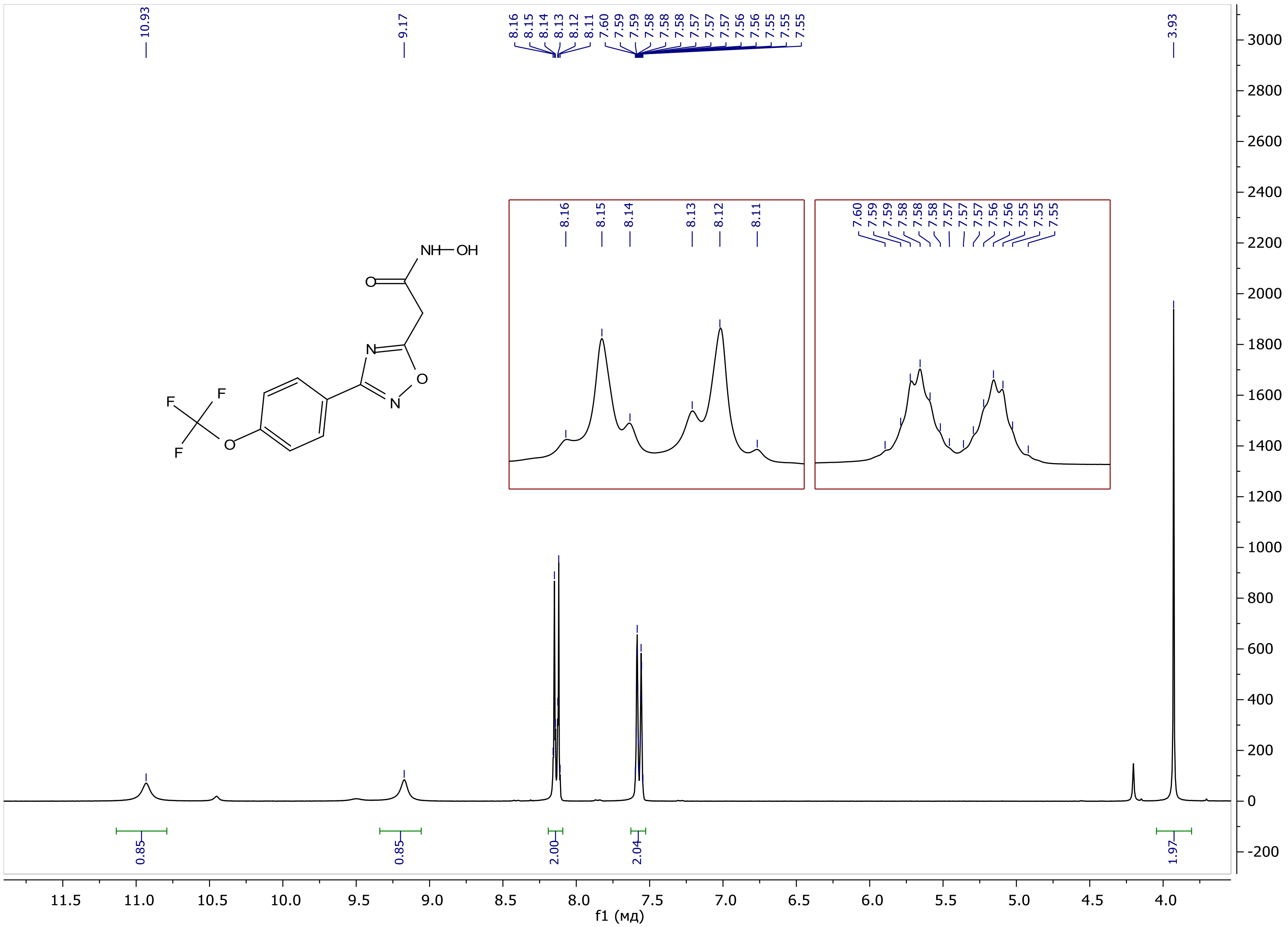

^{13}C NMR spectrum of compound **9b**

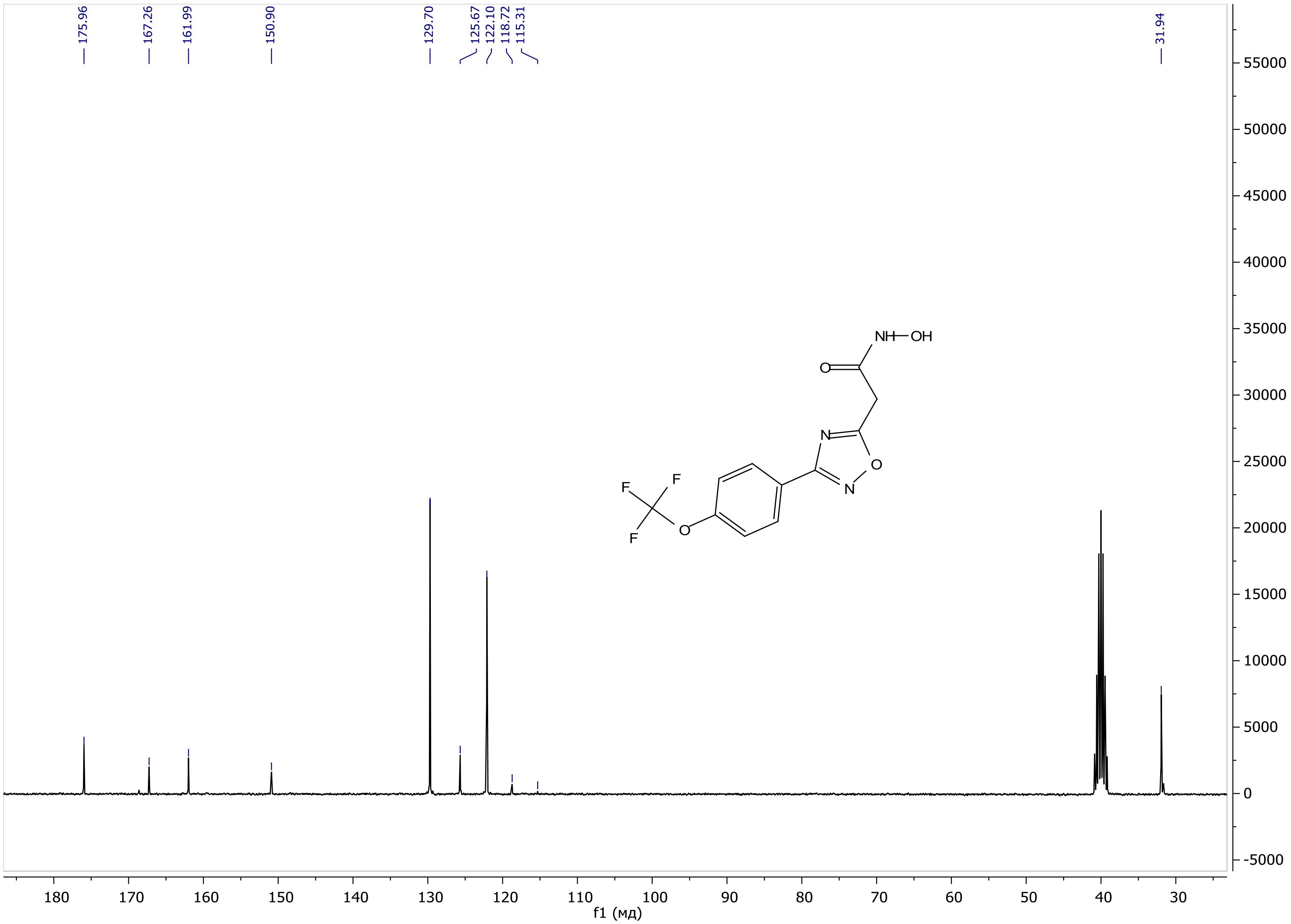

^{1}H NMR spectrum of compound **9c**

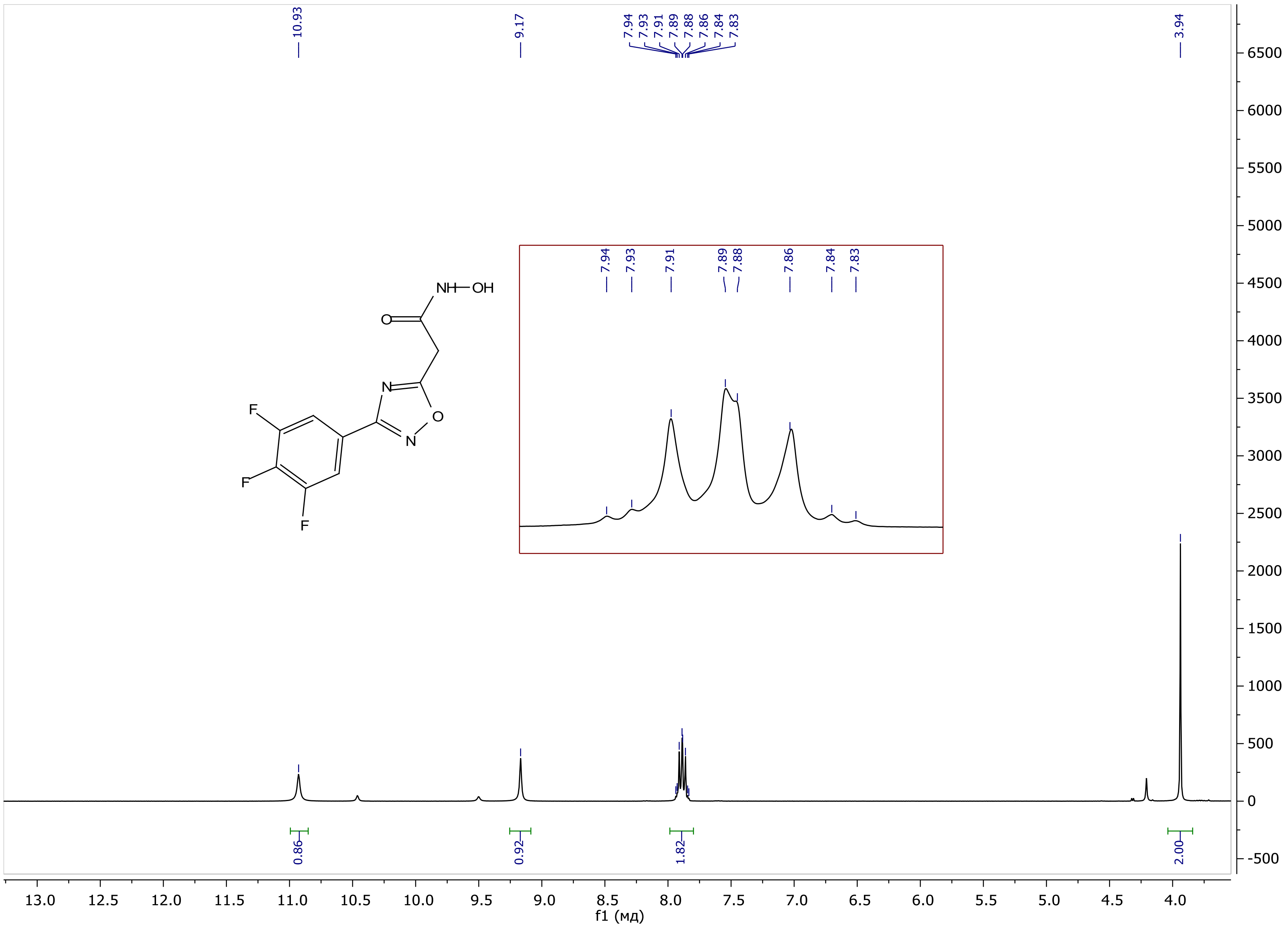

^{13}C NMR spectrum of compound **9c**

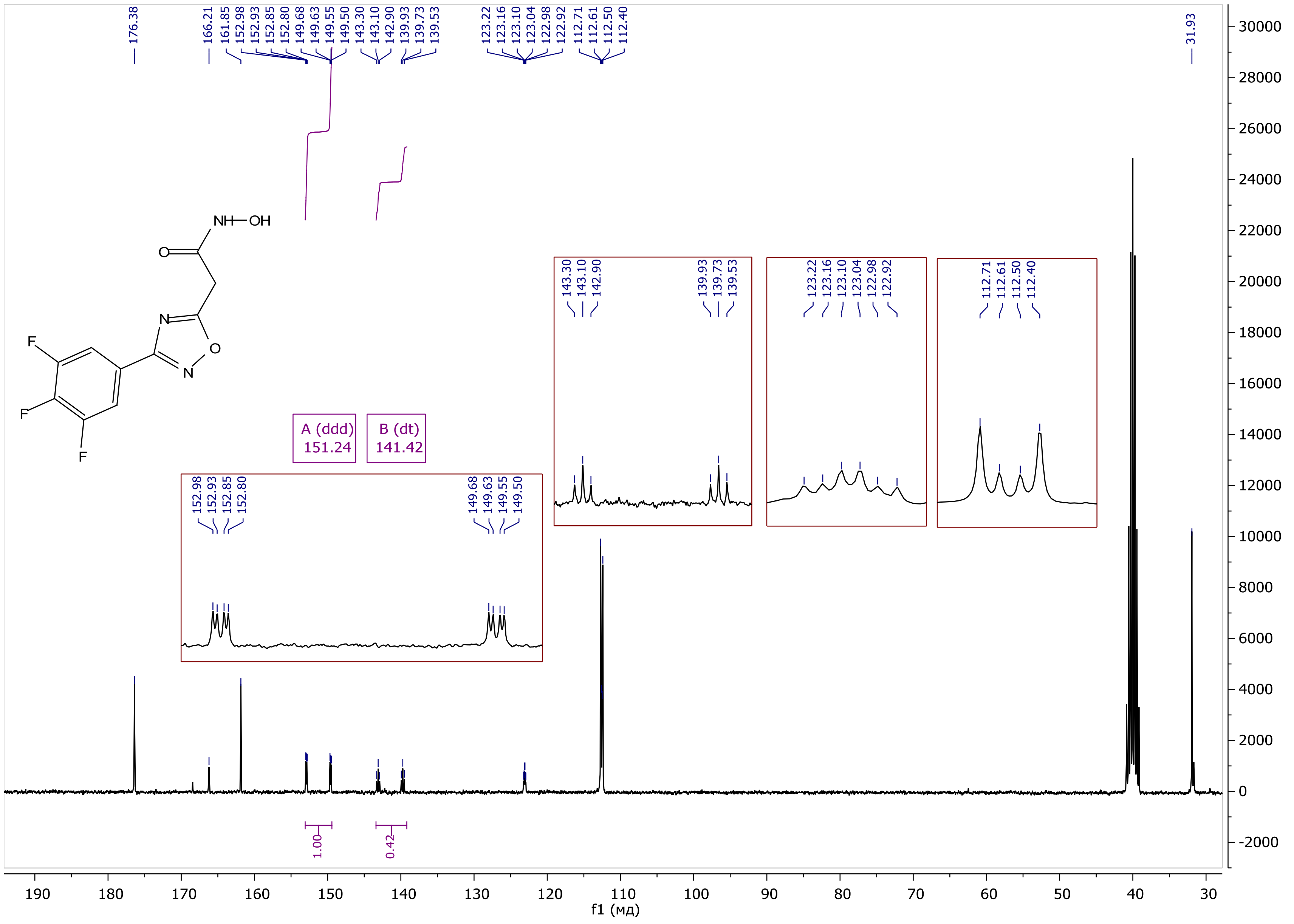

^{1}H NMR spectrum of compound **9d**

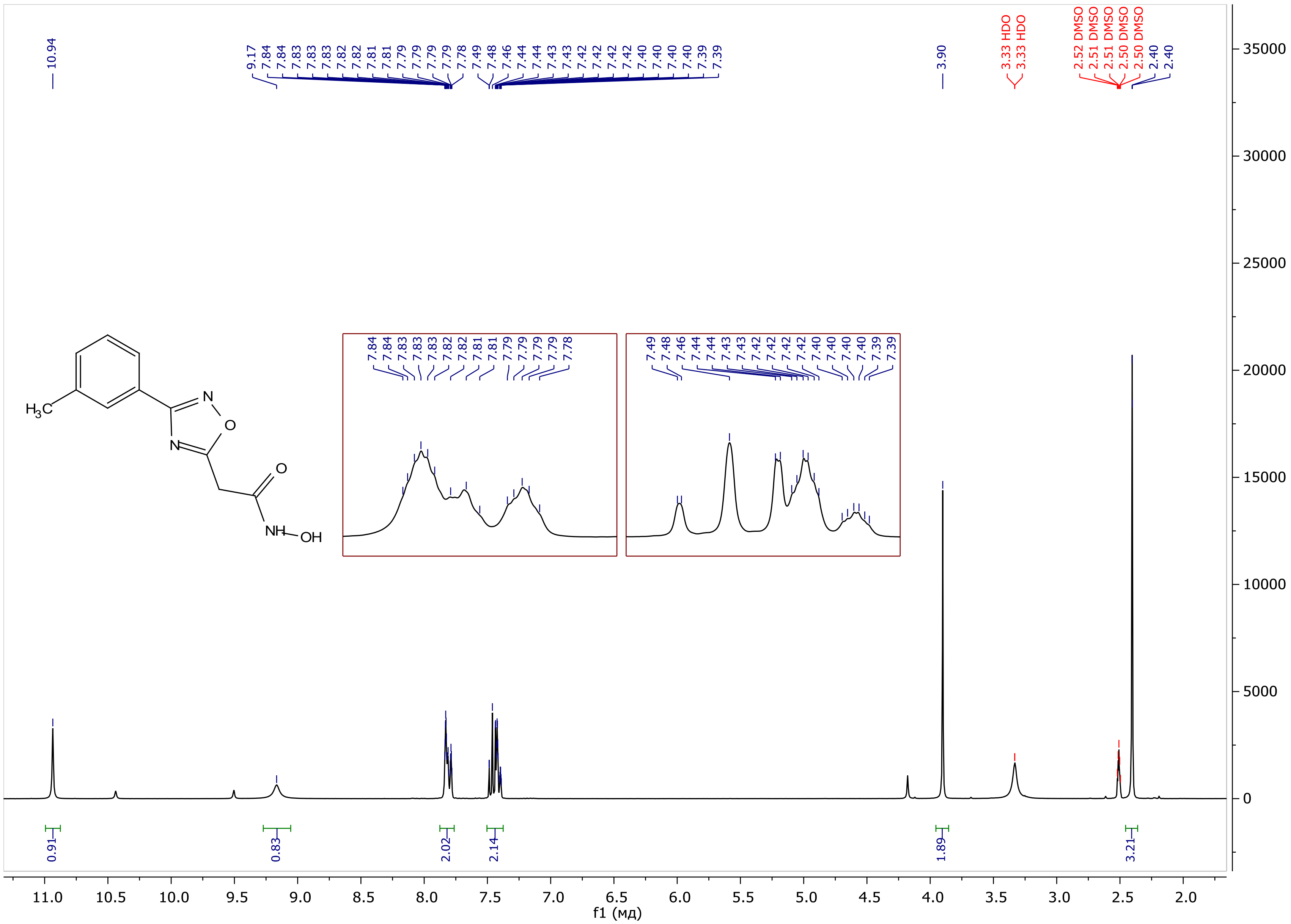

^{13}C NMR spectrum of compound **9d**

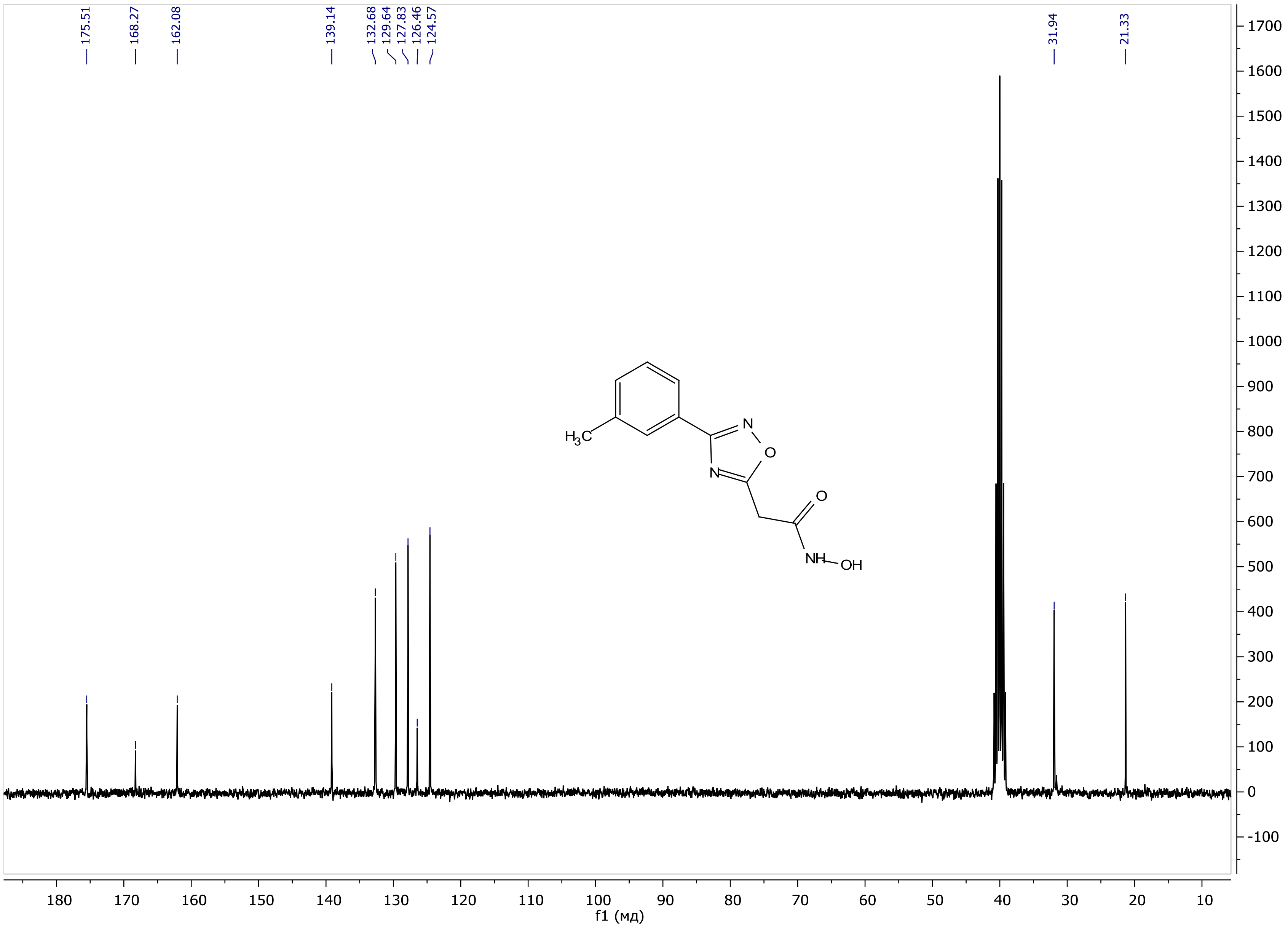

^{1}H, ^{13}C NMR spectrum for 3-(5-(2-fluorophenyl)-1,2,4-oxadiazol-3-yl)-*N*-hydroxybenzamide

^{1}H NMR spectrum of compound **26**

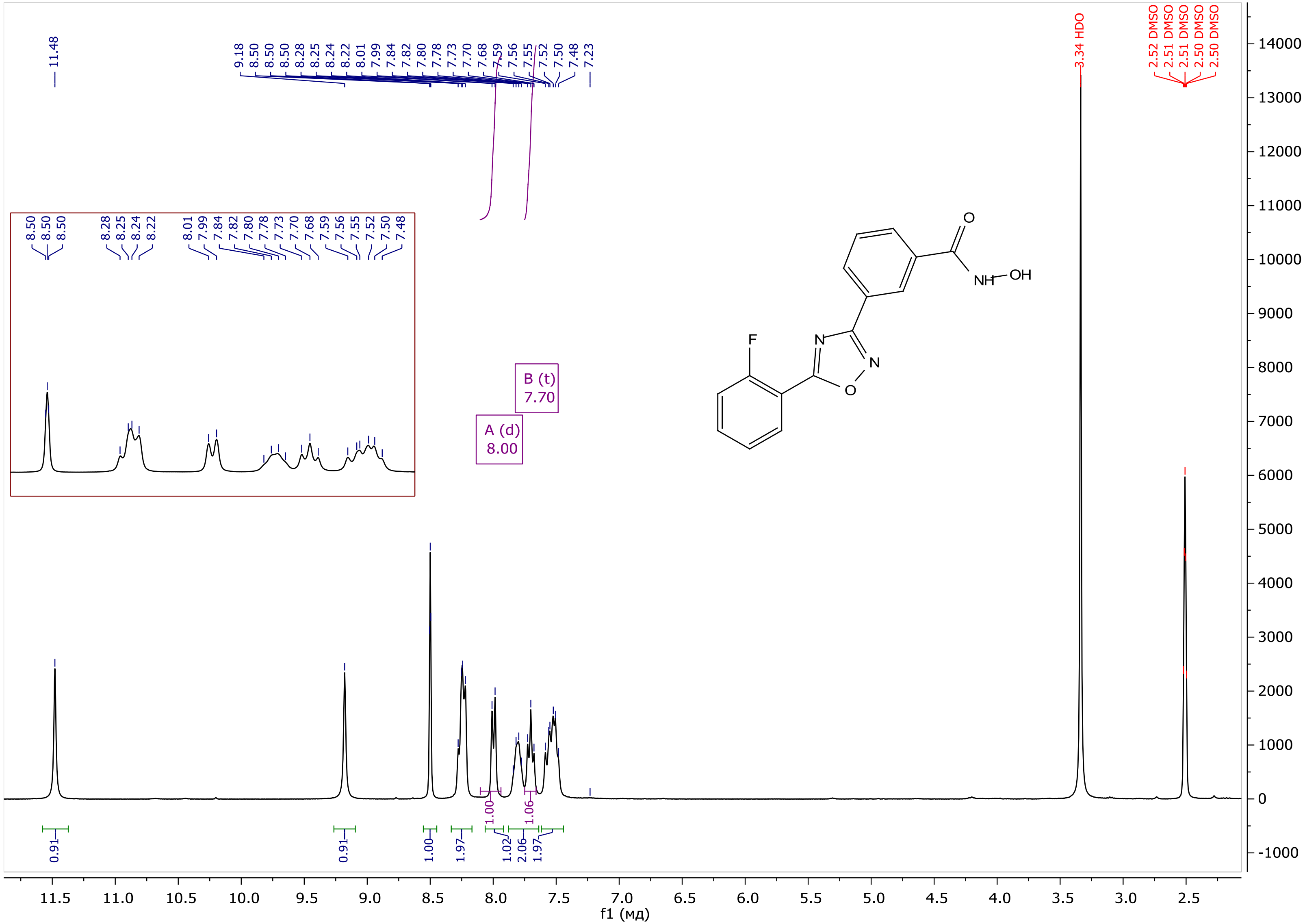

^{13}C NMR spectrum of compound **26**

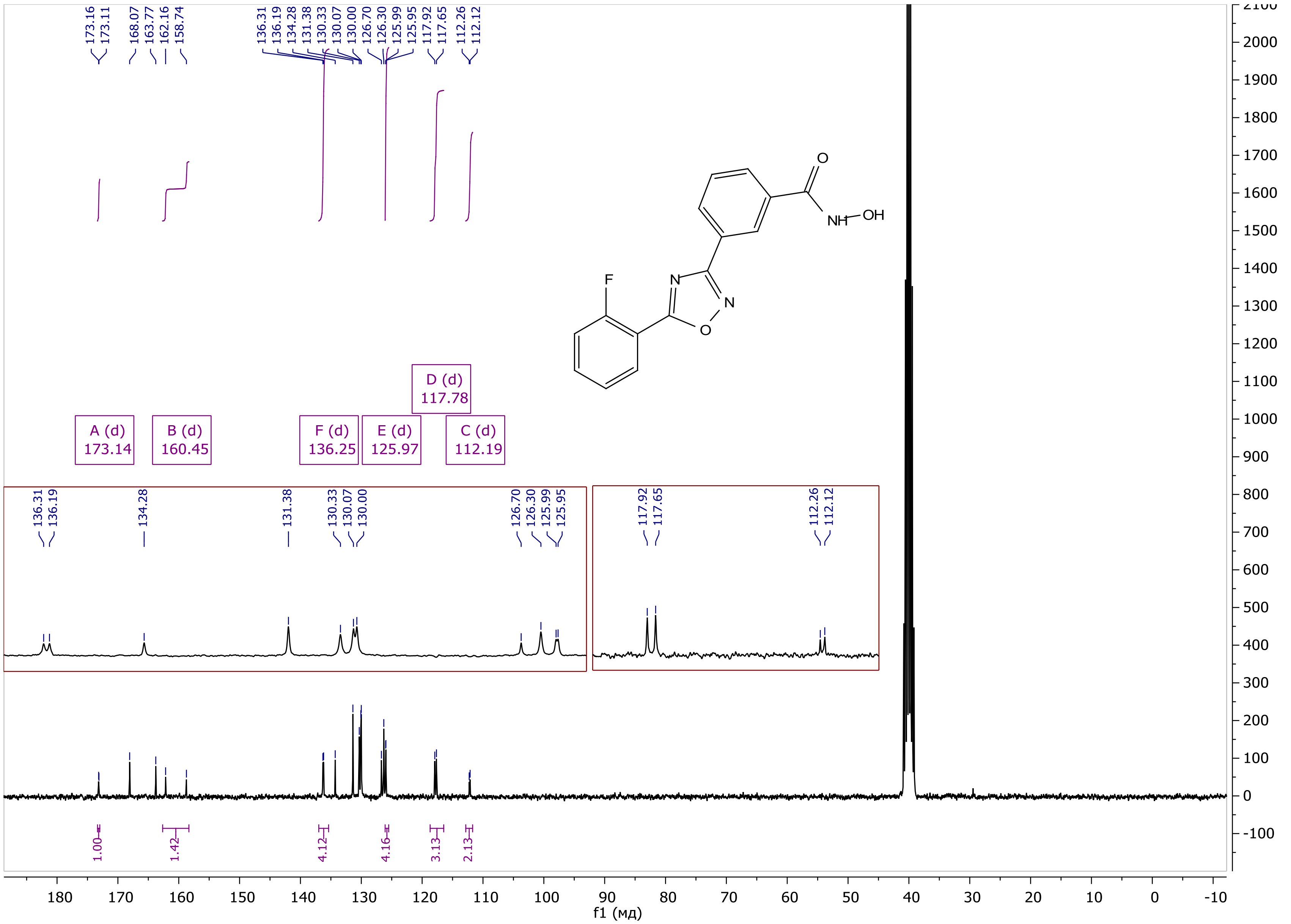

^{1}H, ^{13}C NMR spectra for 3-(3-aryl-1,2,4-oxadiazol-5-yl)-*N*-hydroxybenzamides synthesized

^{1}H NMR spectrum of compound **14a**

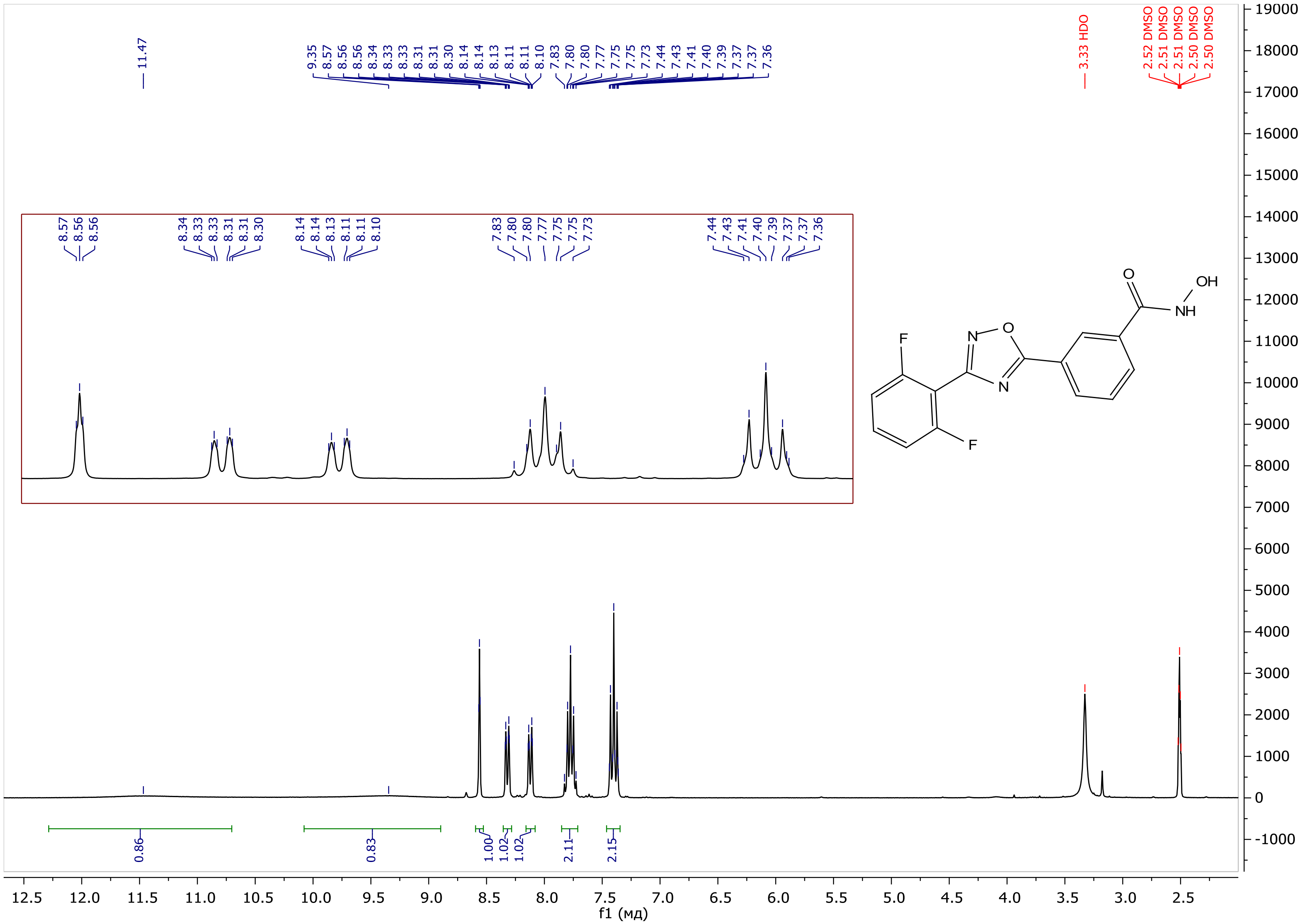

^{13}C NMR spectrum of compound **14a**

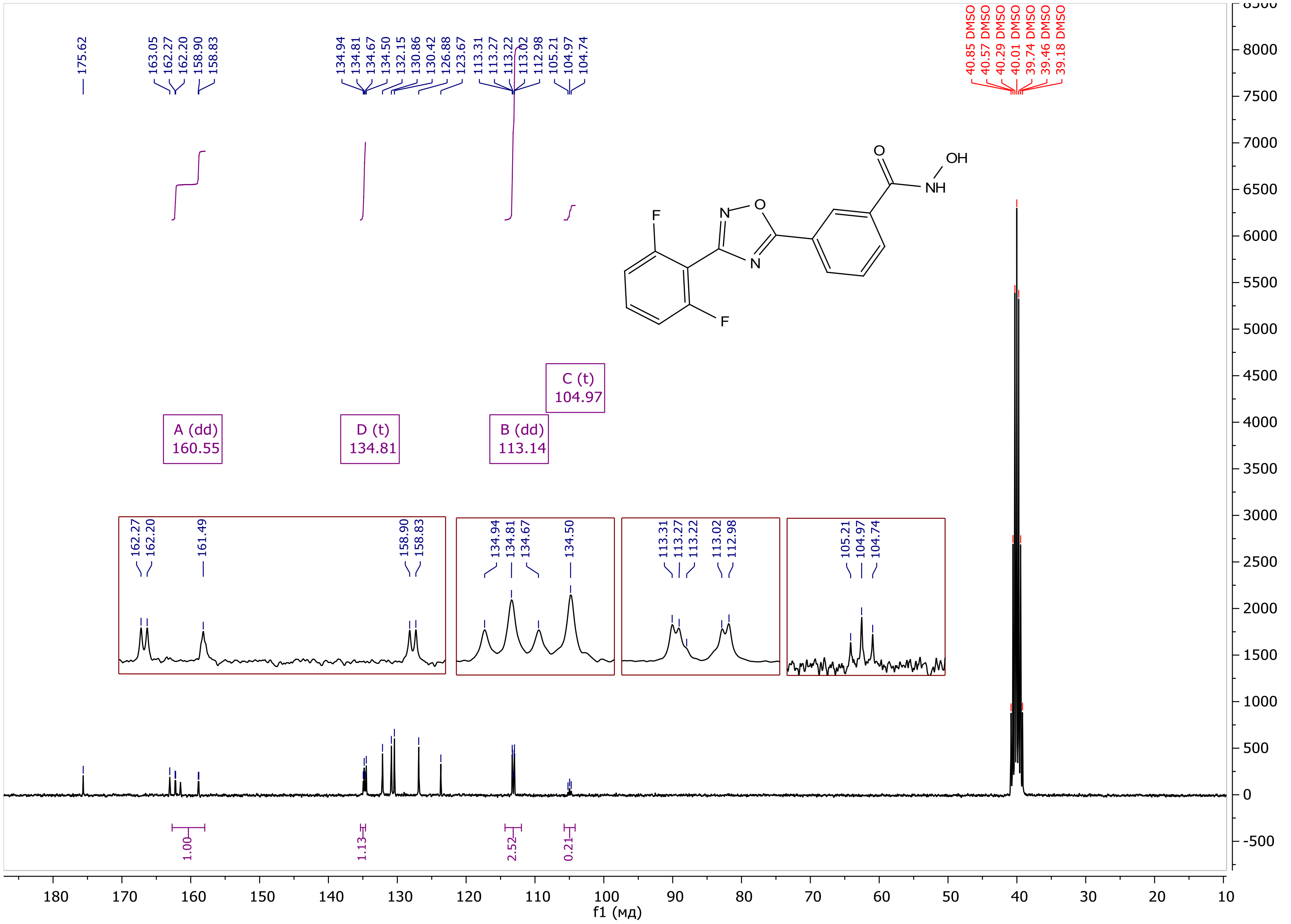

^{1}H NMR spectrum of compound **14b**

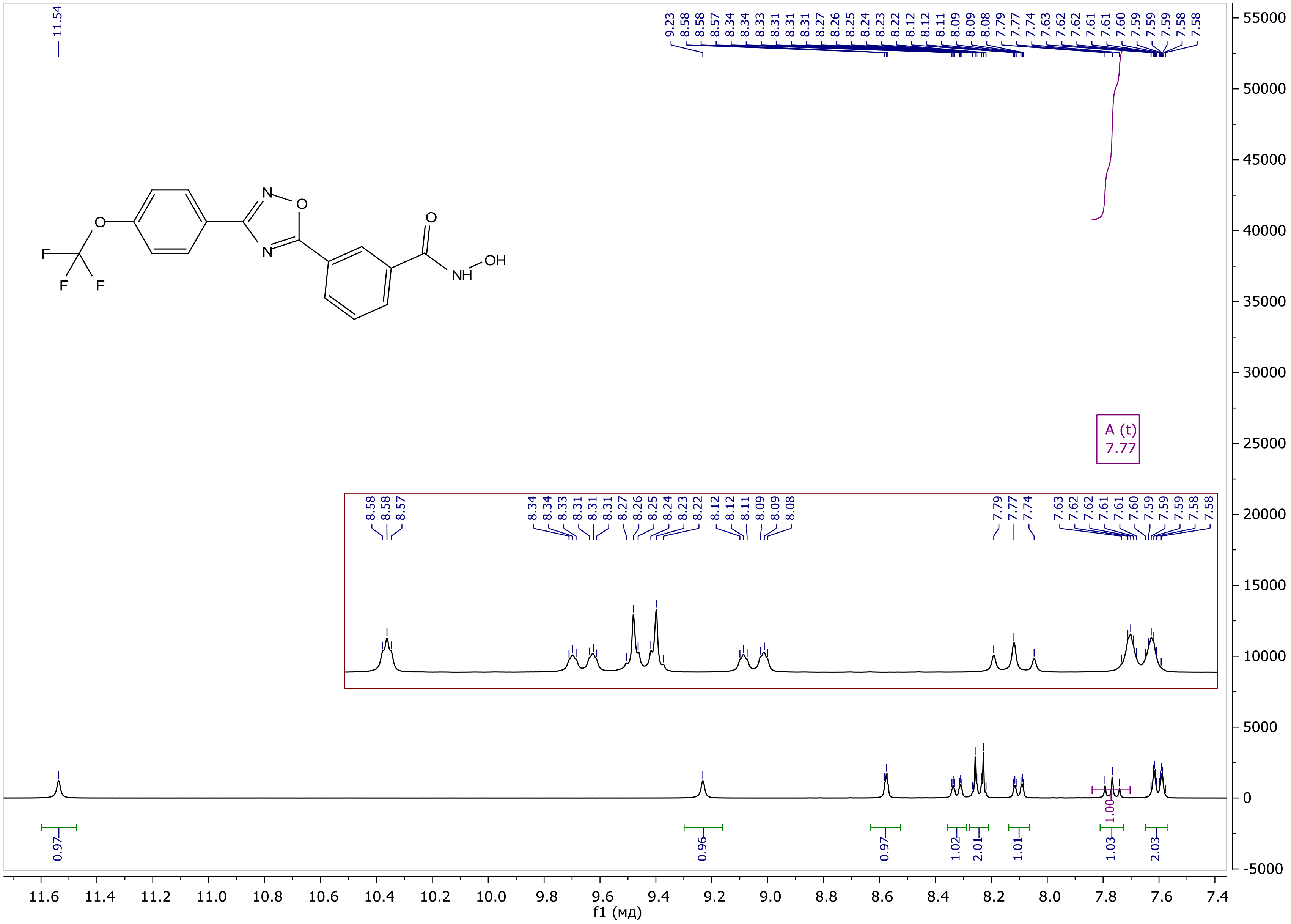

^{13}C NMR spectrum of compound **14b**

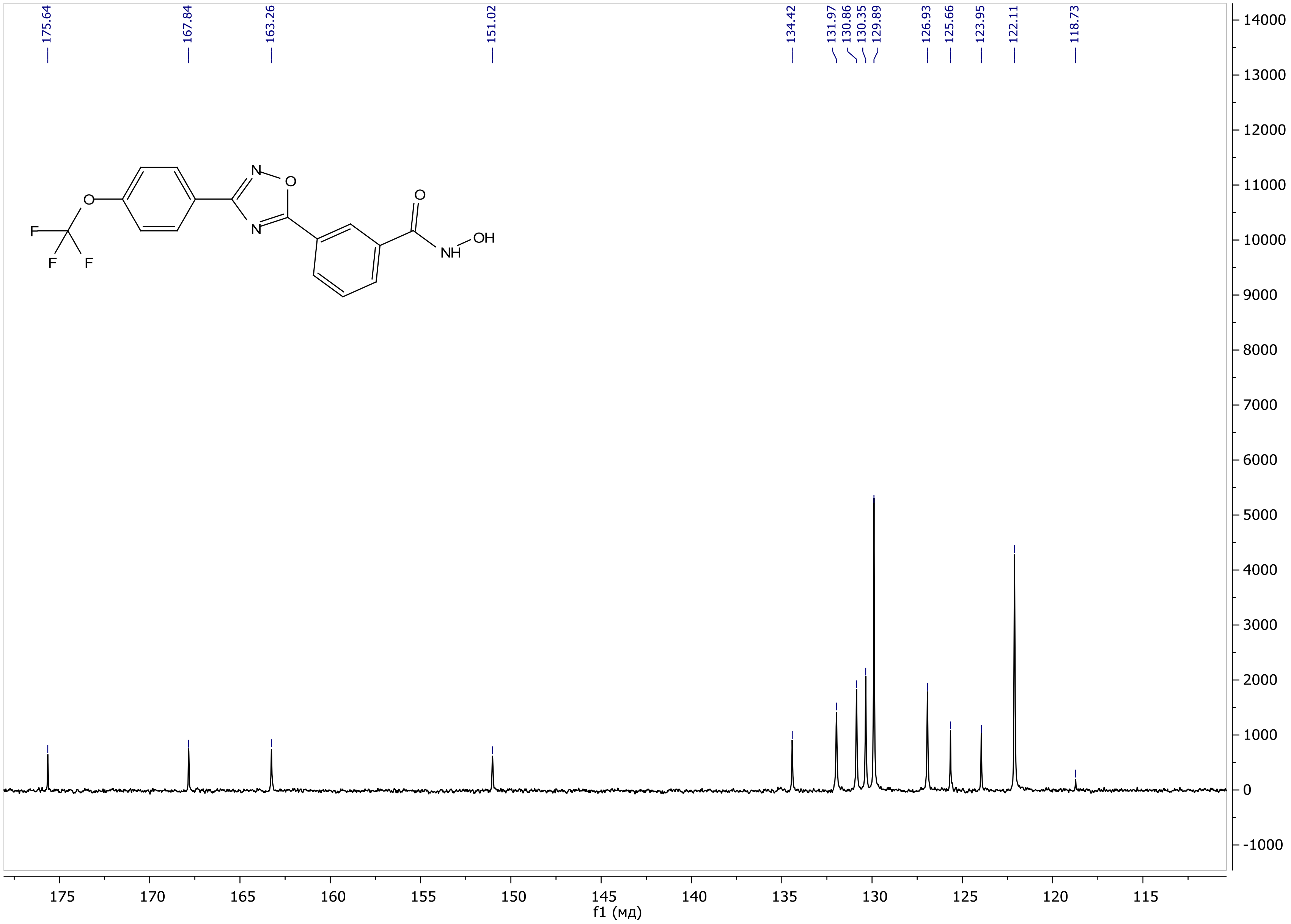

^{1}H NMR spectrum of compound **14c**

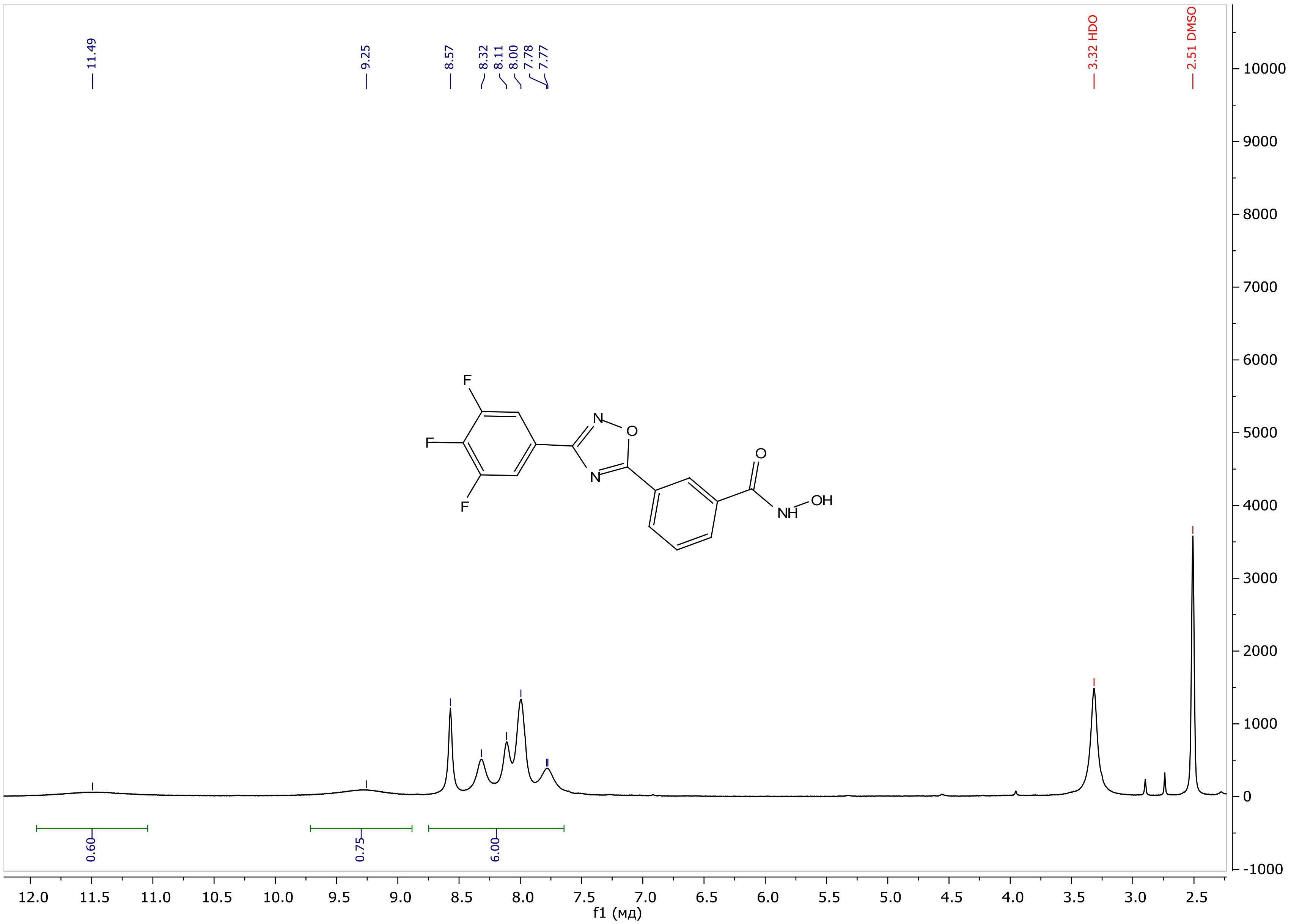

^{13}C NMR spectrum of compound **14c**

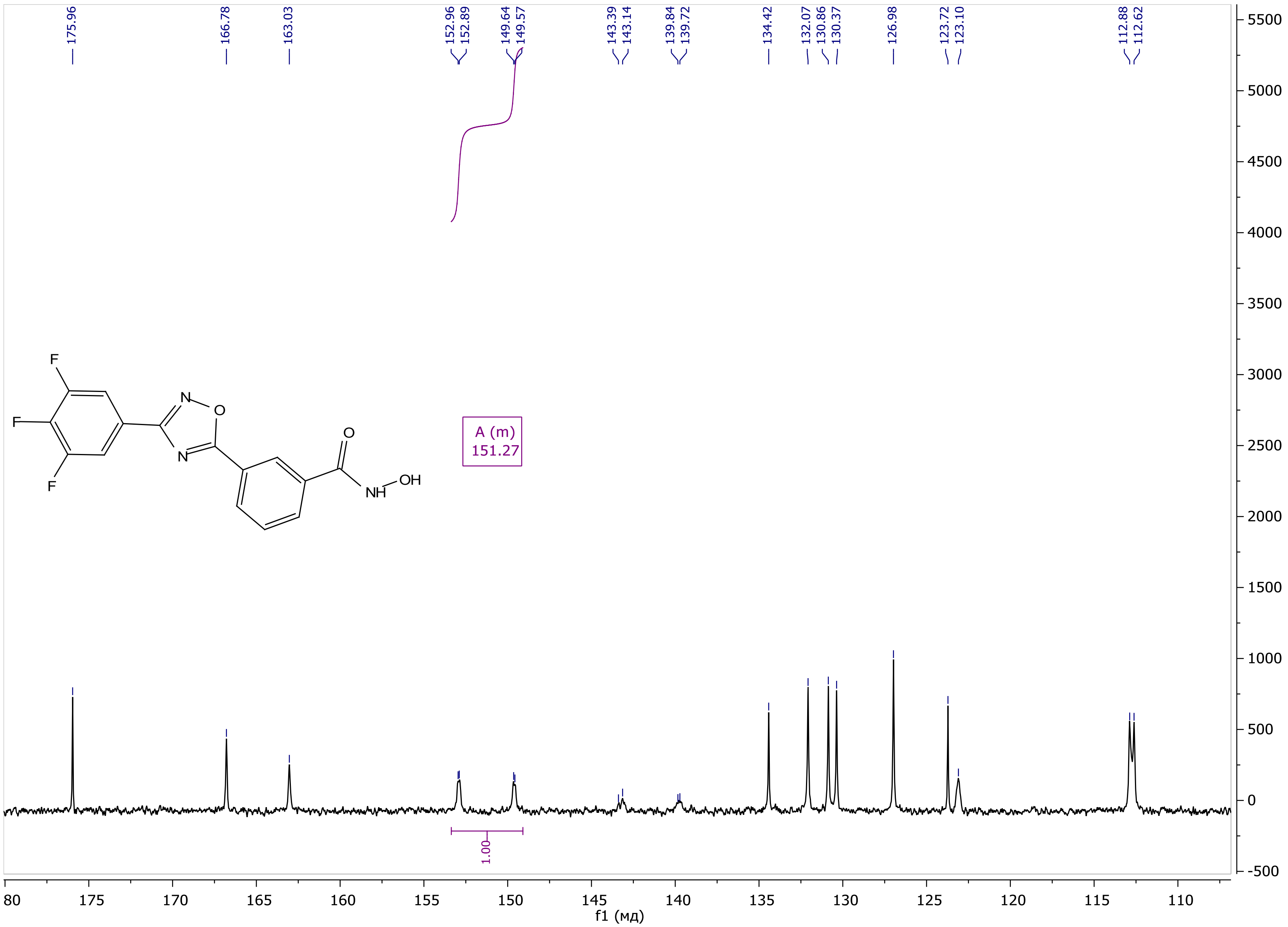

^{1}H NMR spectrum of compound **14d**

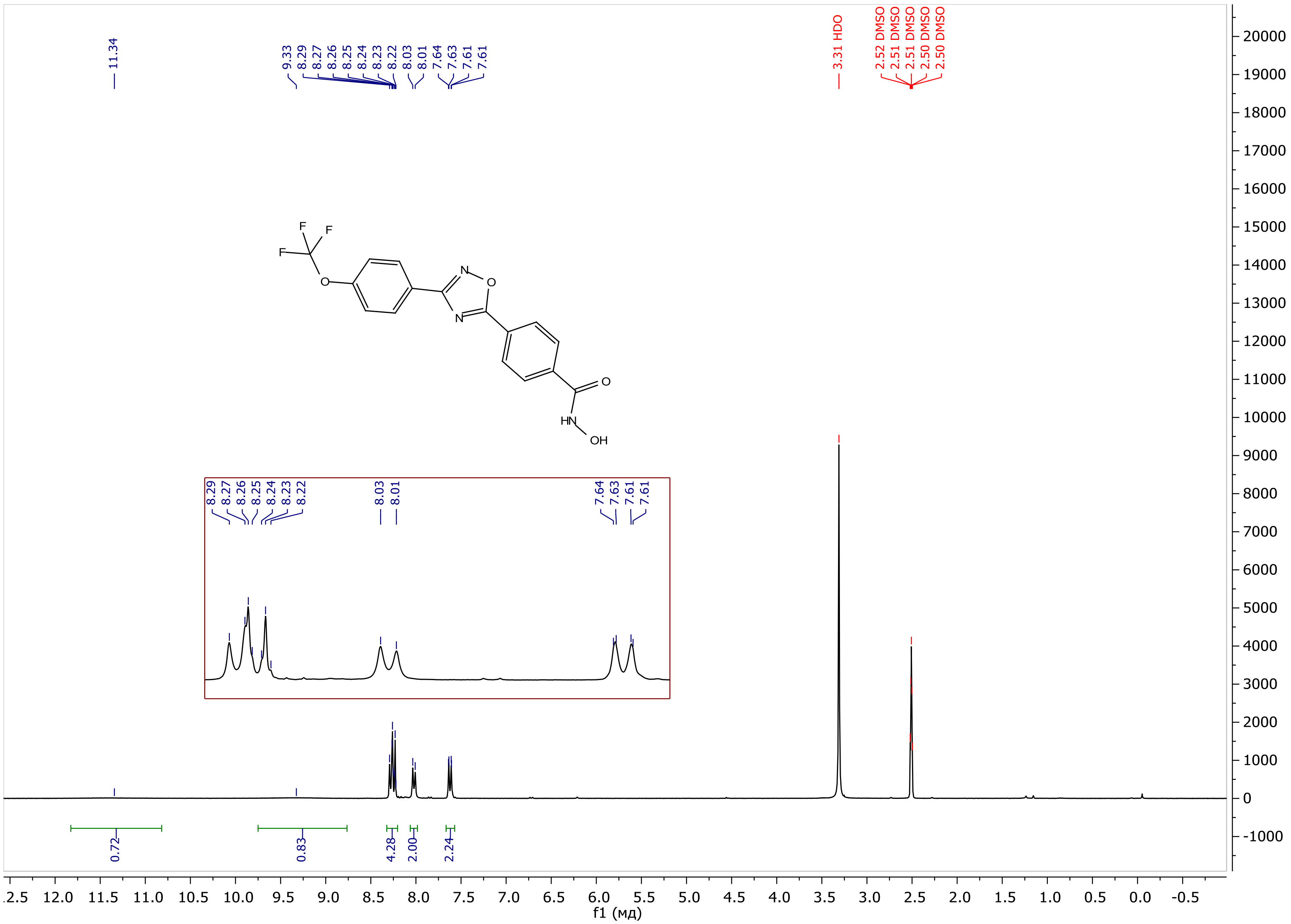

^{13}C NMR spectrum of compound **14d**

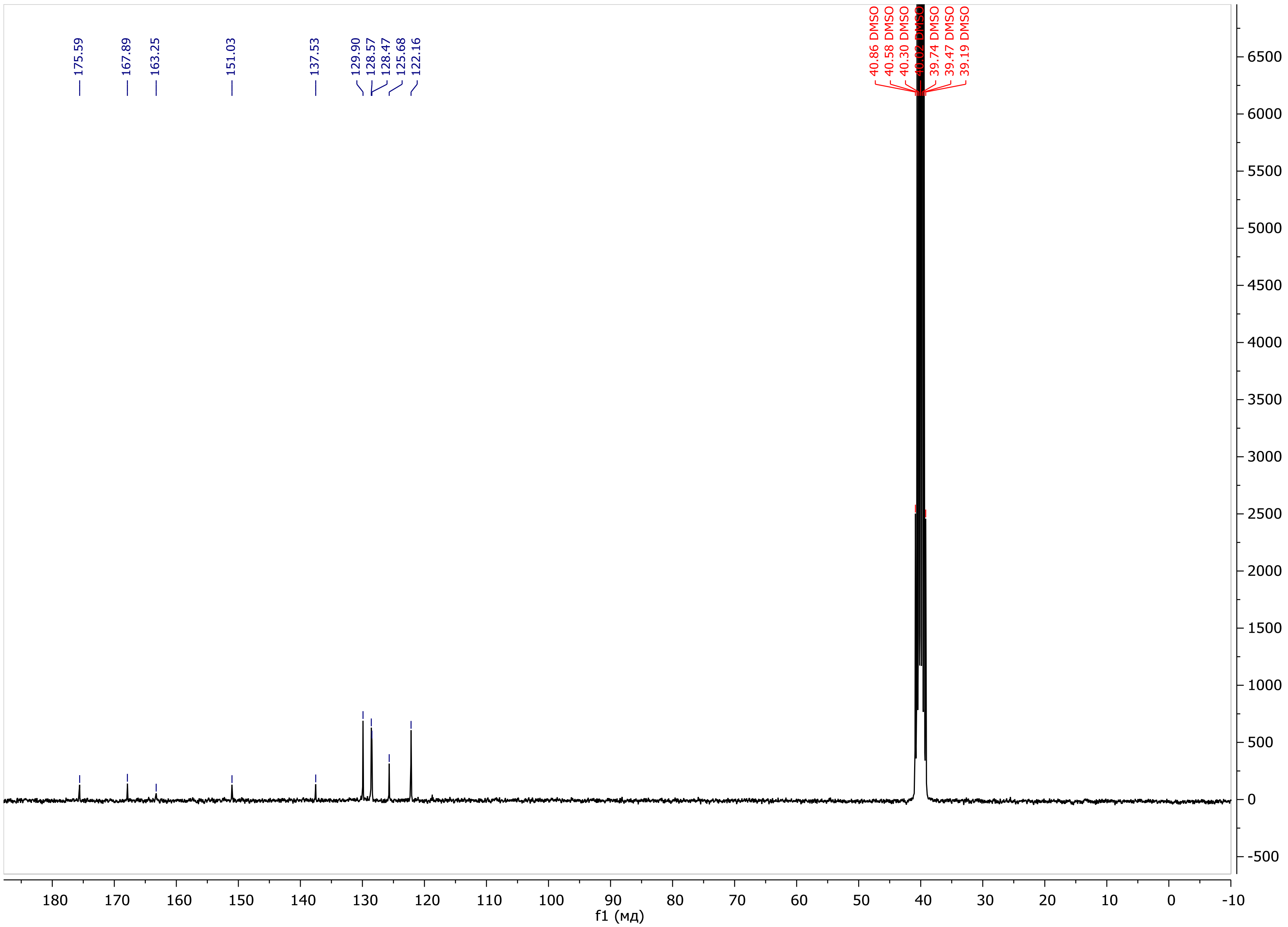

^{1}H NMR spectrum of compound **14e**

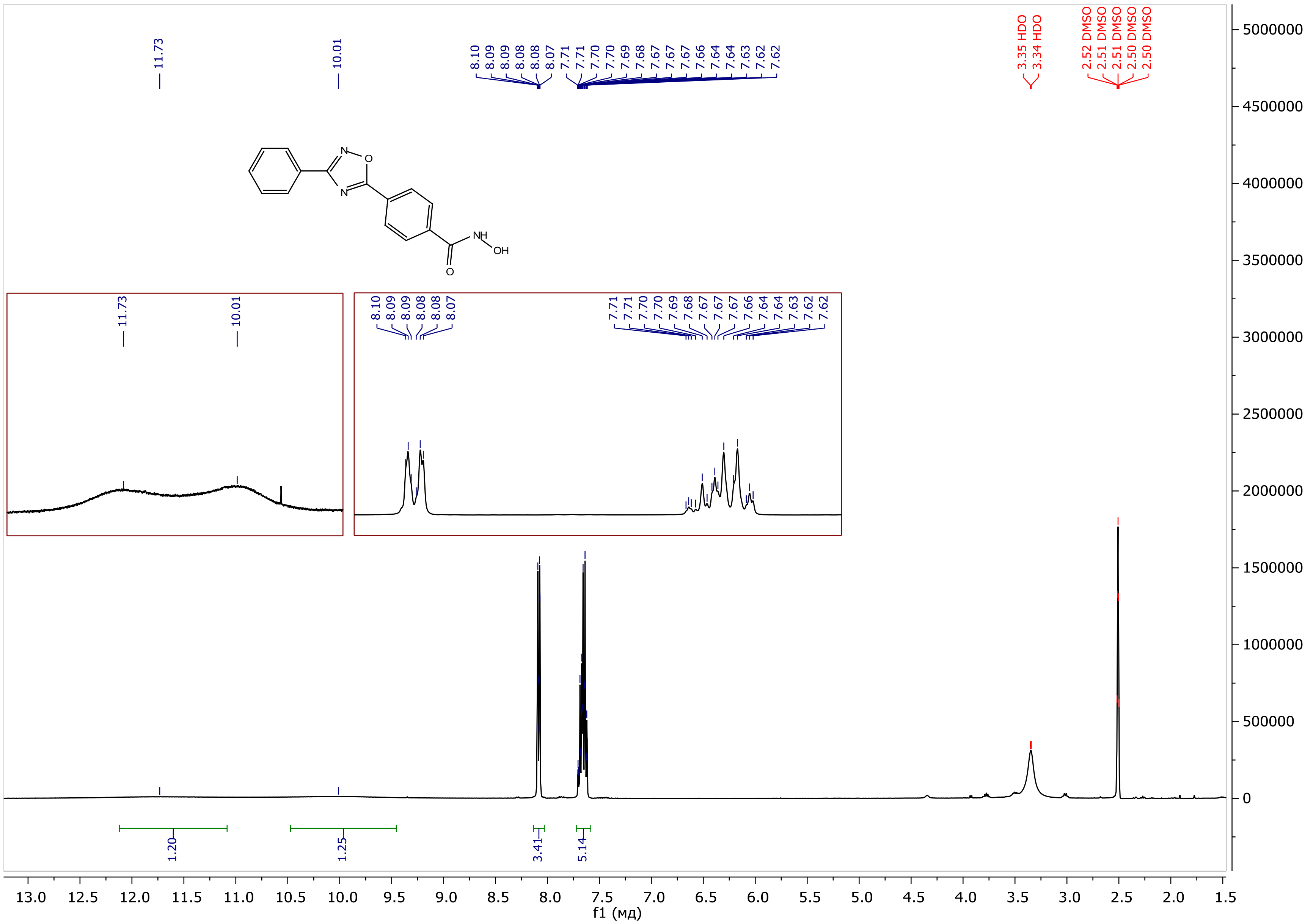

^{13}C NMR spectrum of compound **14e**

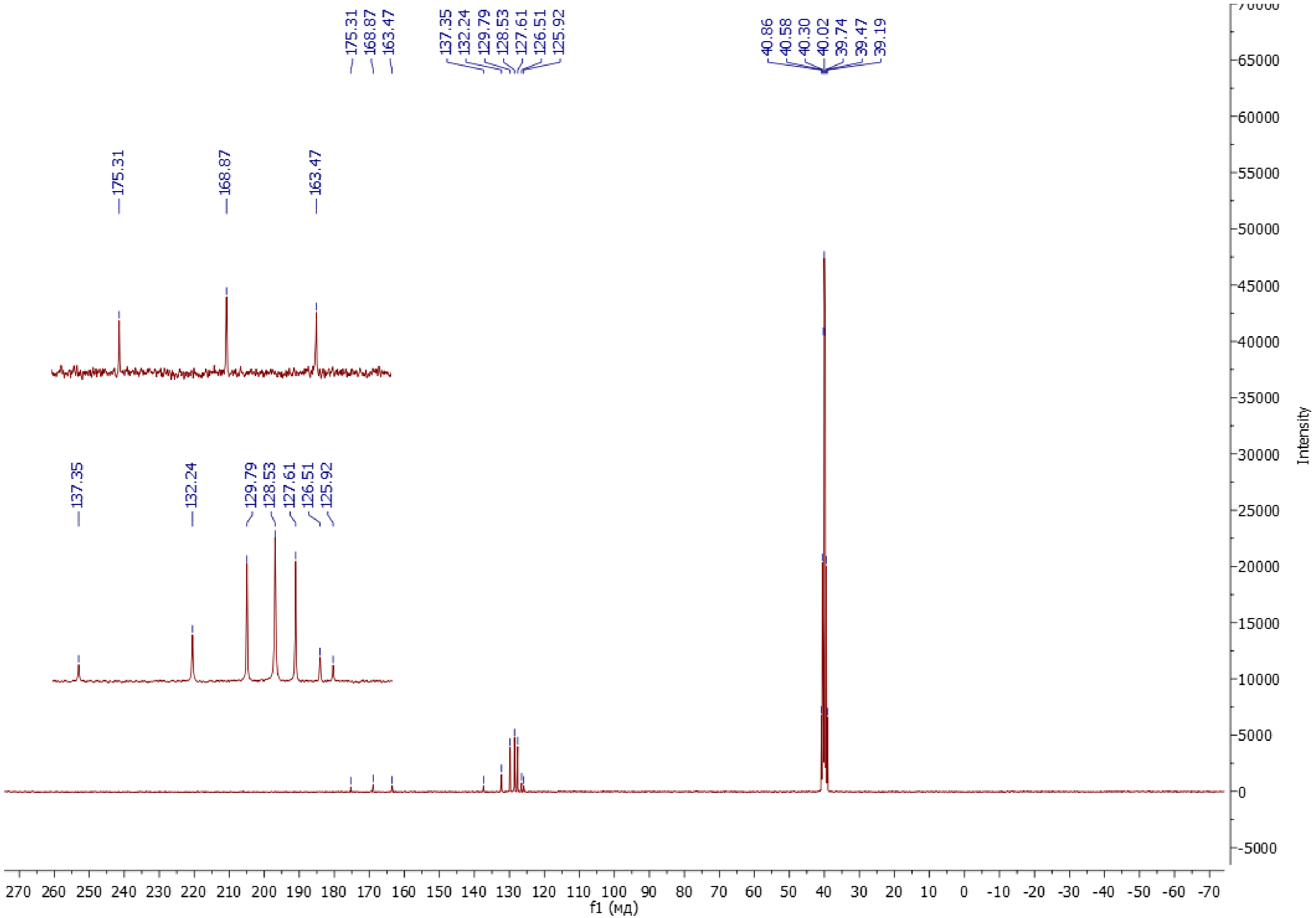

^{1}H, ^{13}C NMR spectra for 2-(5-aryl-1,2,4-oxadiazol-3-yl)-*N*-hydroxyacetamides synthesized

^{1}H NMR spectrum of compound **23a**

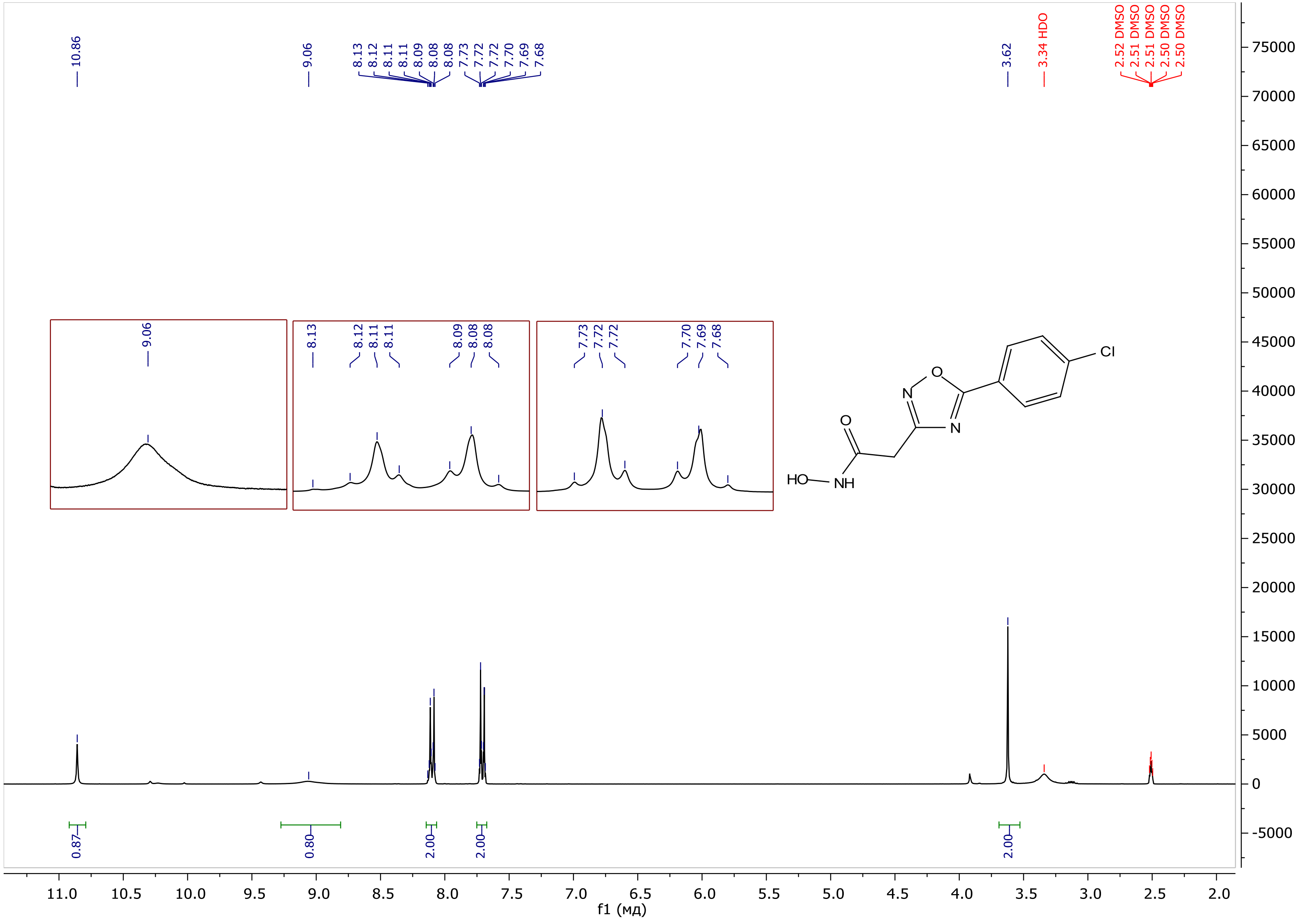

^{13}C NMR spectrum of compound **23a**

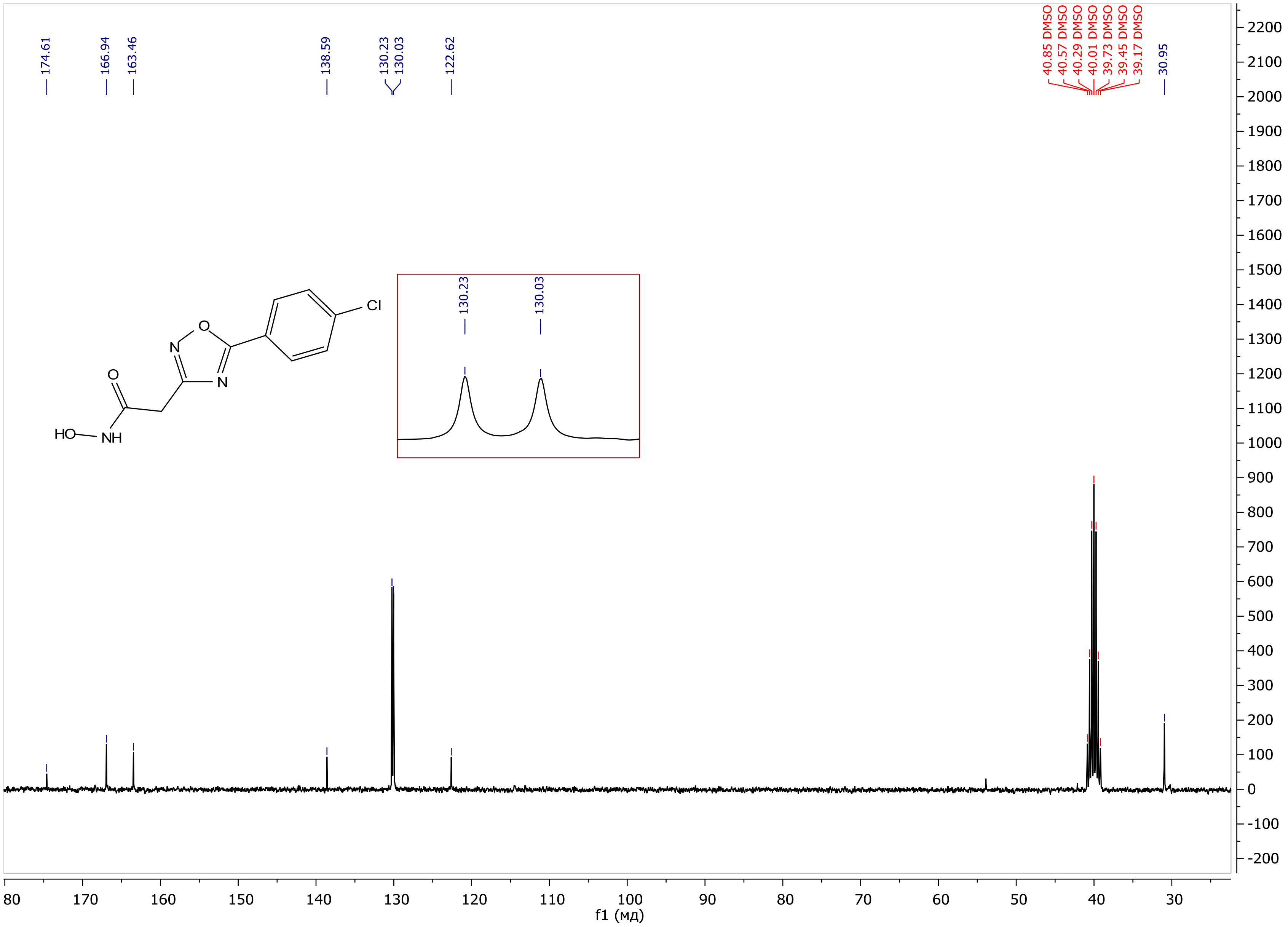

^{1}H NMR spectrum of compound **23b**

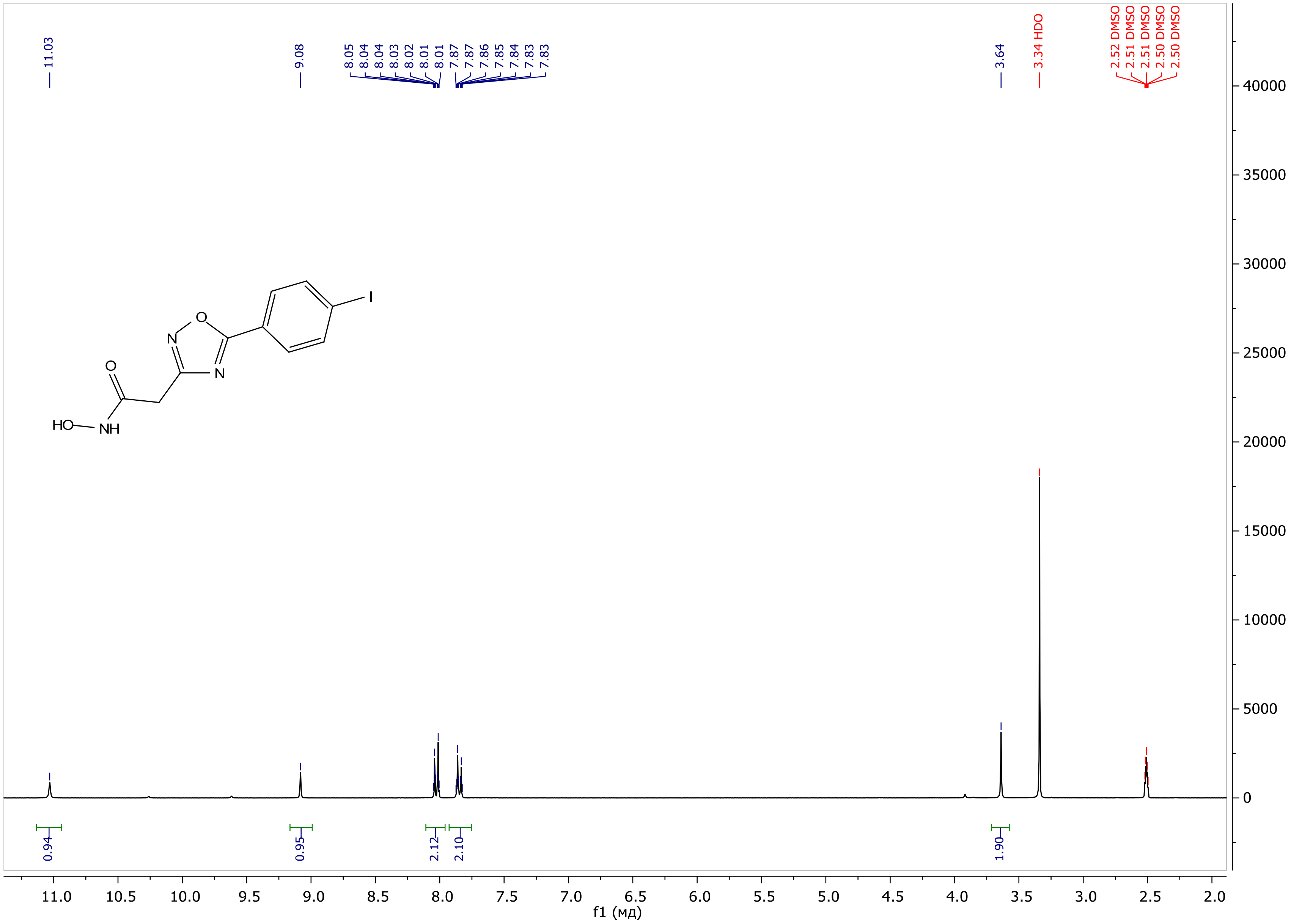

^{13}C NMR spectrum of compound **23b**

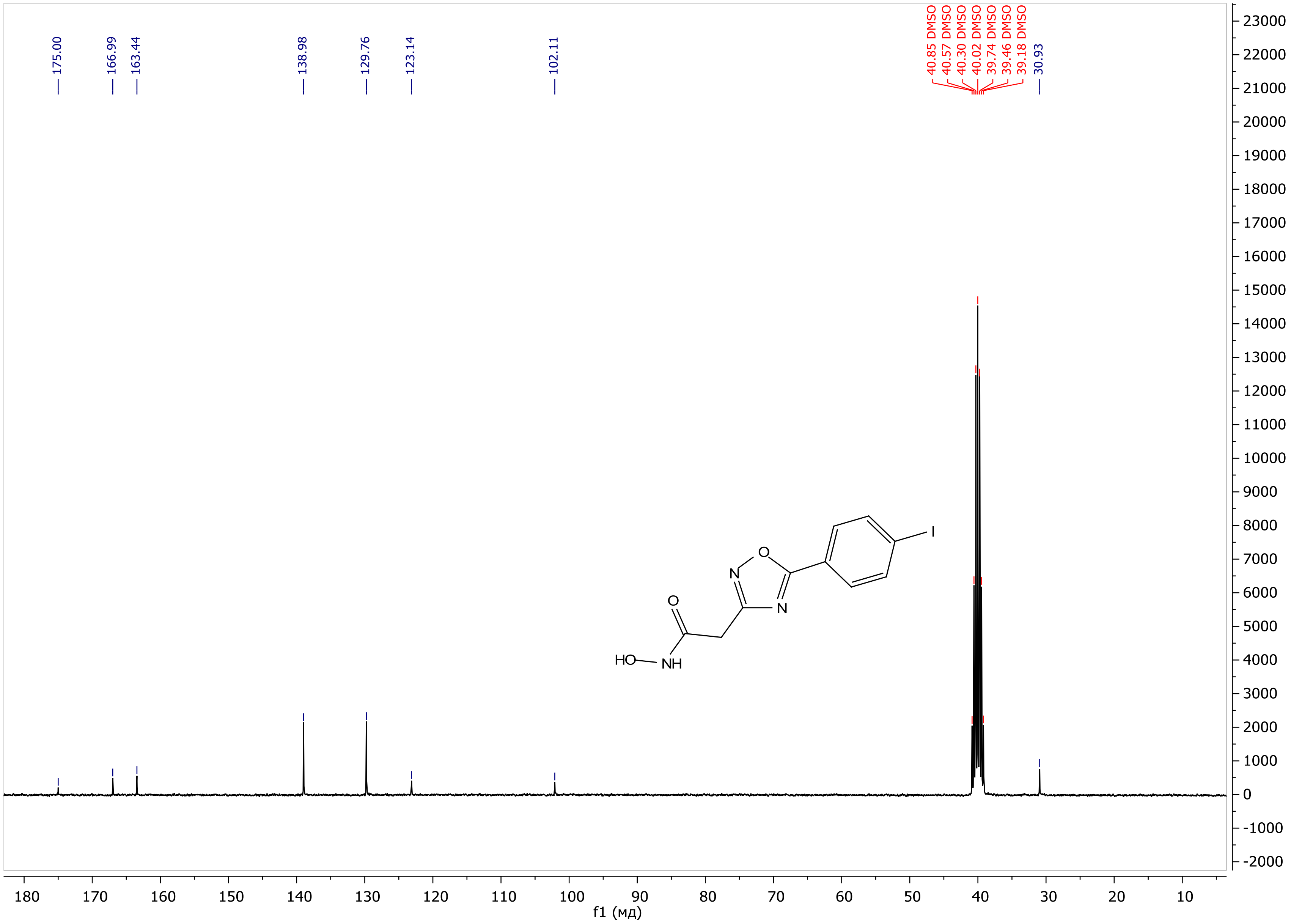

^{1}H NMR spectrum of compound **23c**

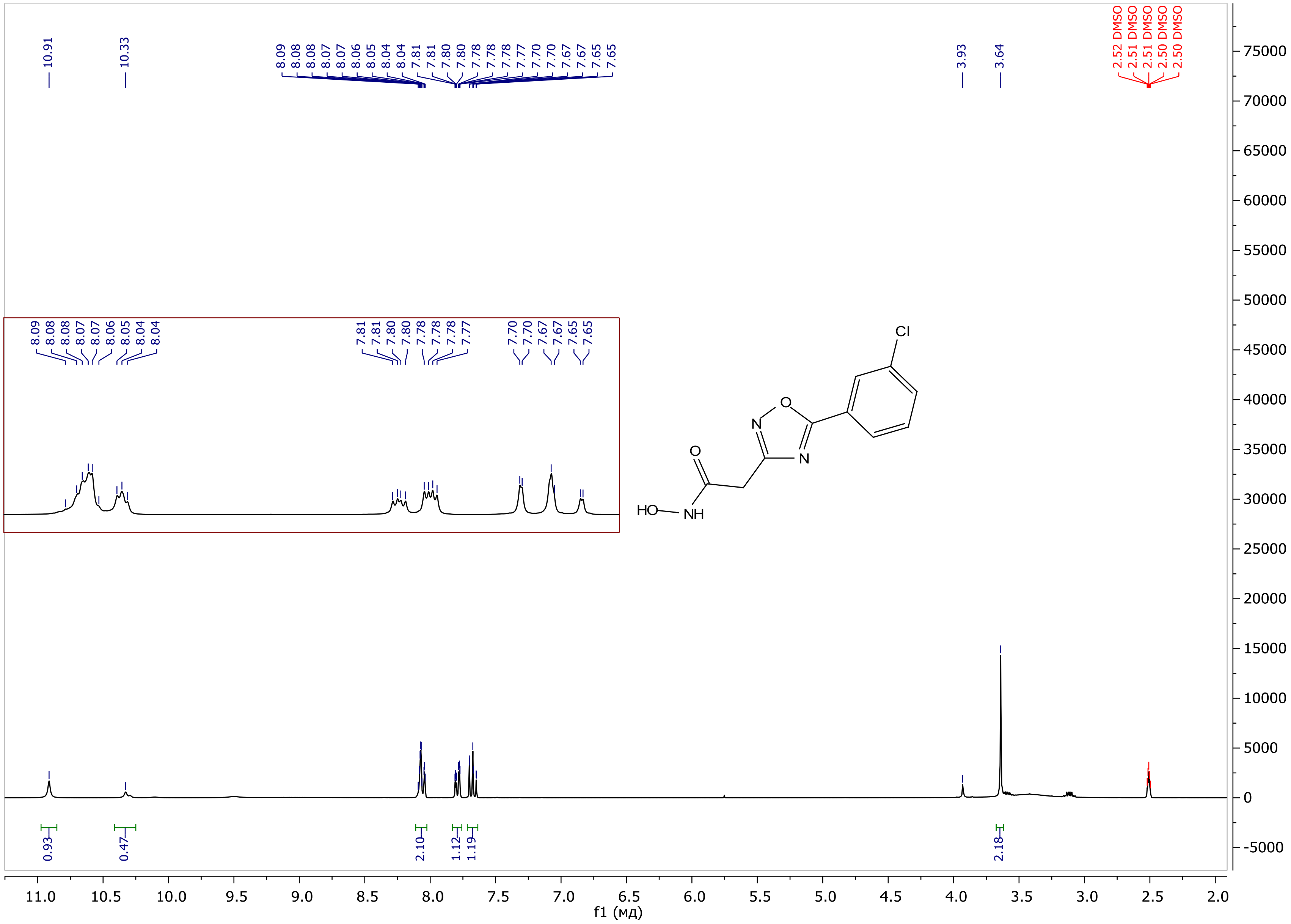

^{13}C NMR spectrum of compound **23c**

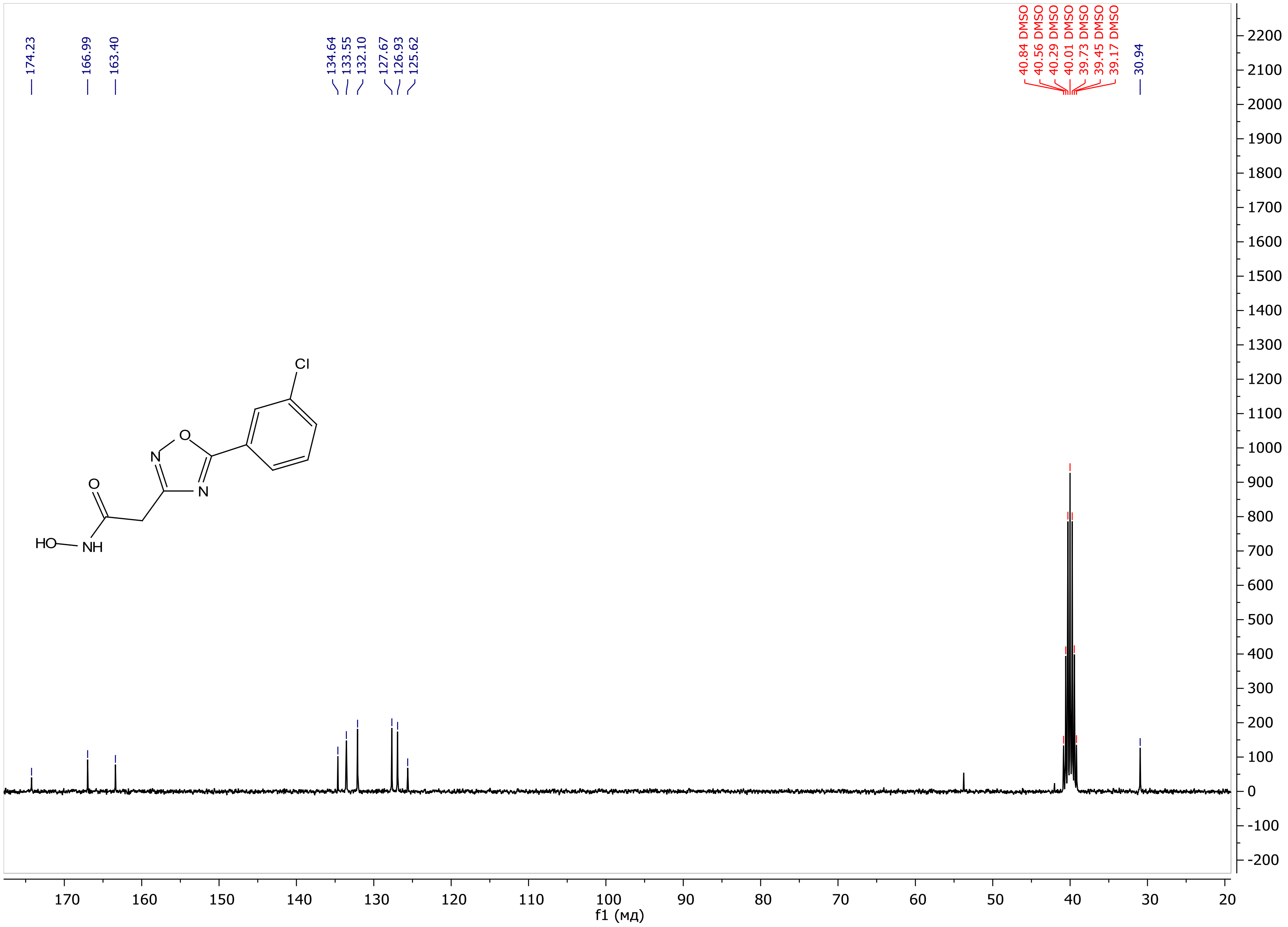

^{1}H NMR spectrum of compound **23d**

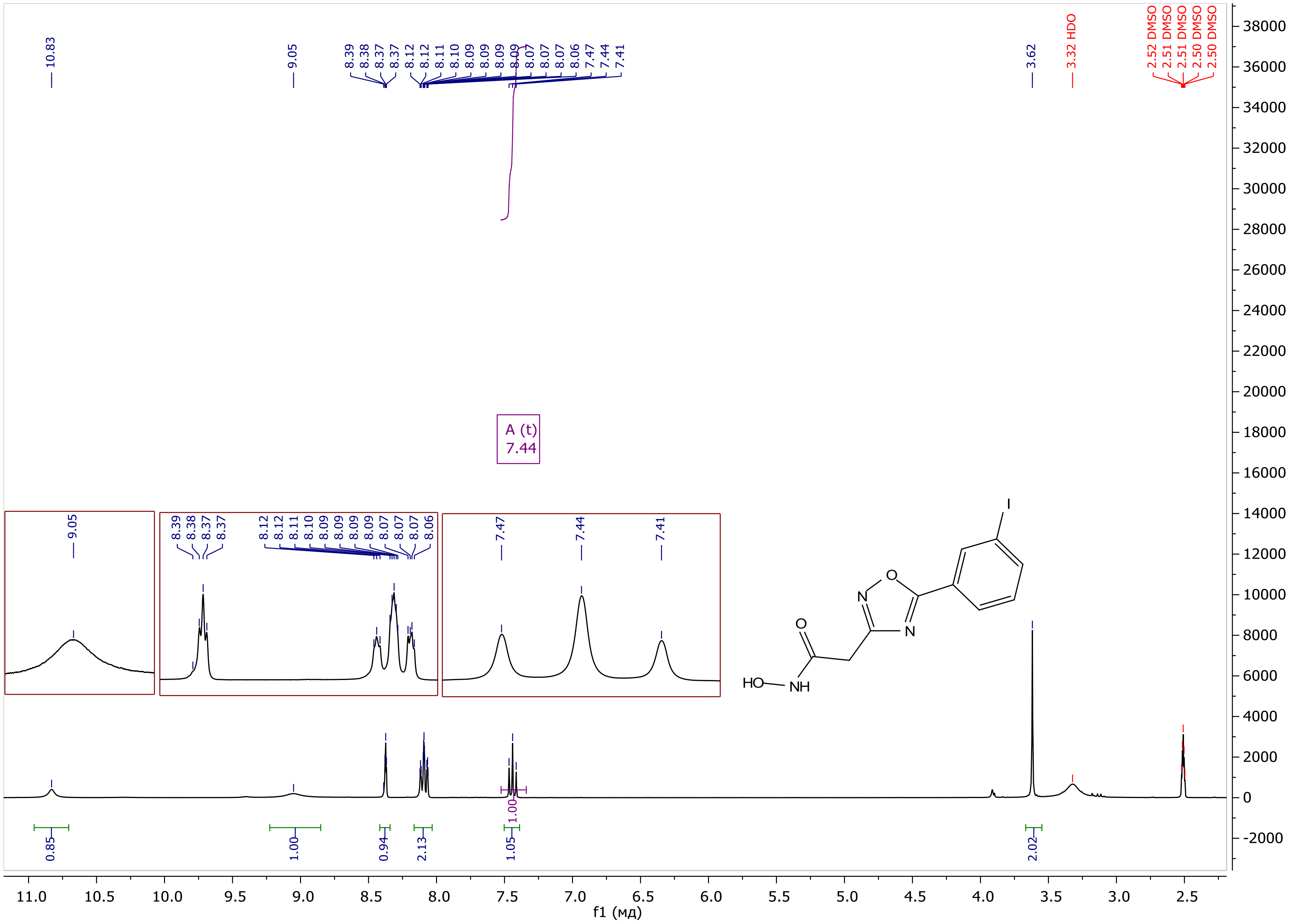

^{13}C NMR spectrum of compound **23d**

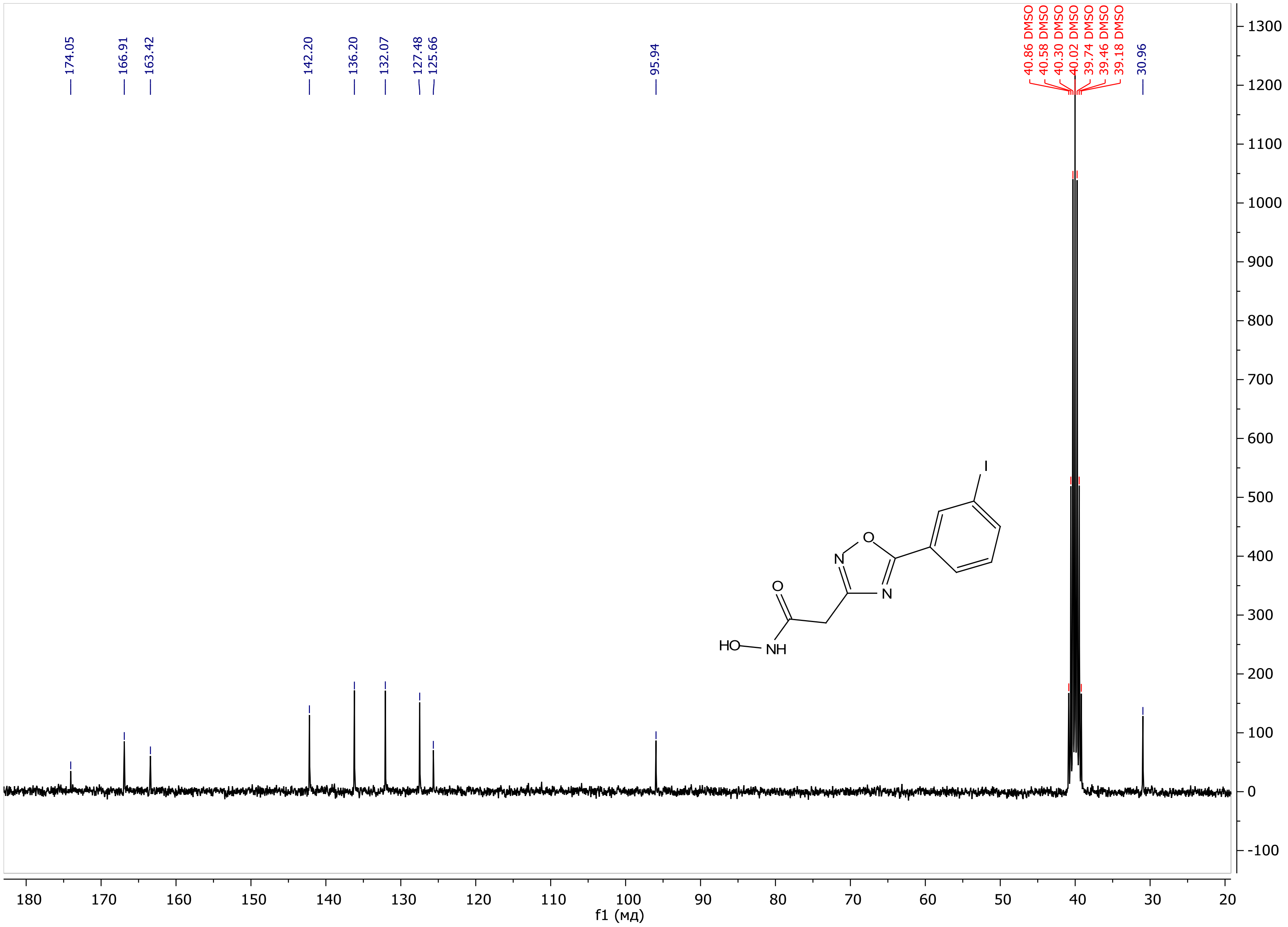

^{1}H, ^{13}C NMR spectra for *N*-hydroxy-3-aryl-1,2,4-oxadiazol-5-yl)propanamides synthesized

^{1}H NMR spectrum of compound **17a**

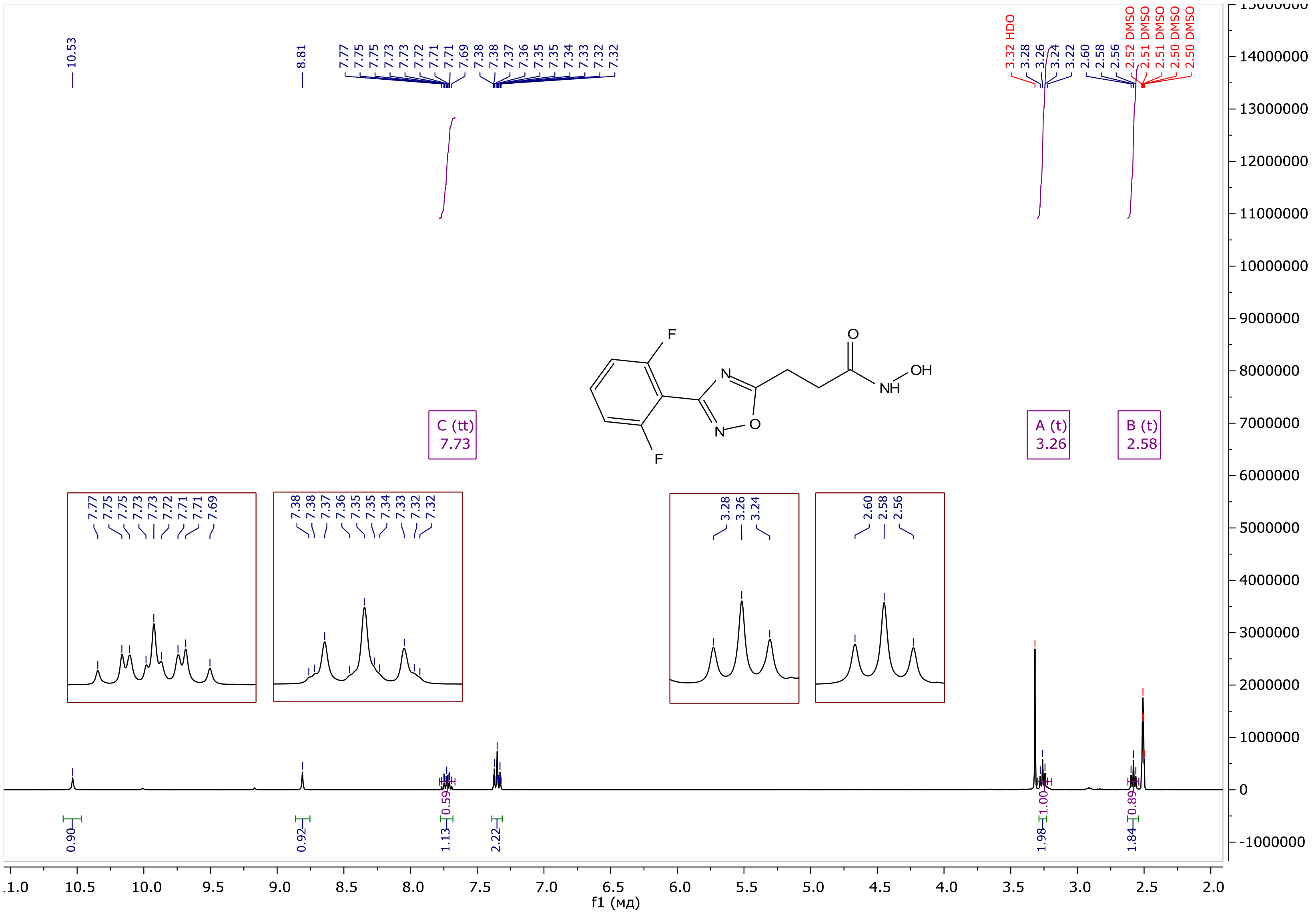

^{13}C NMR spectrum of compound **17a**

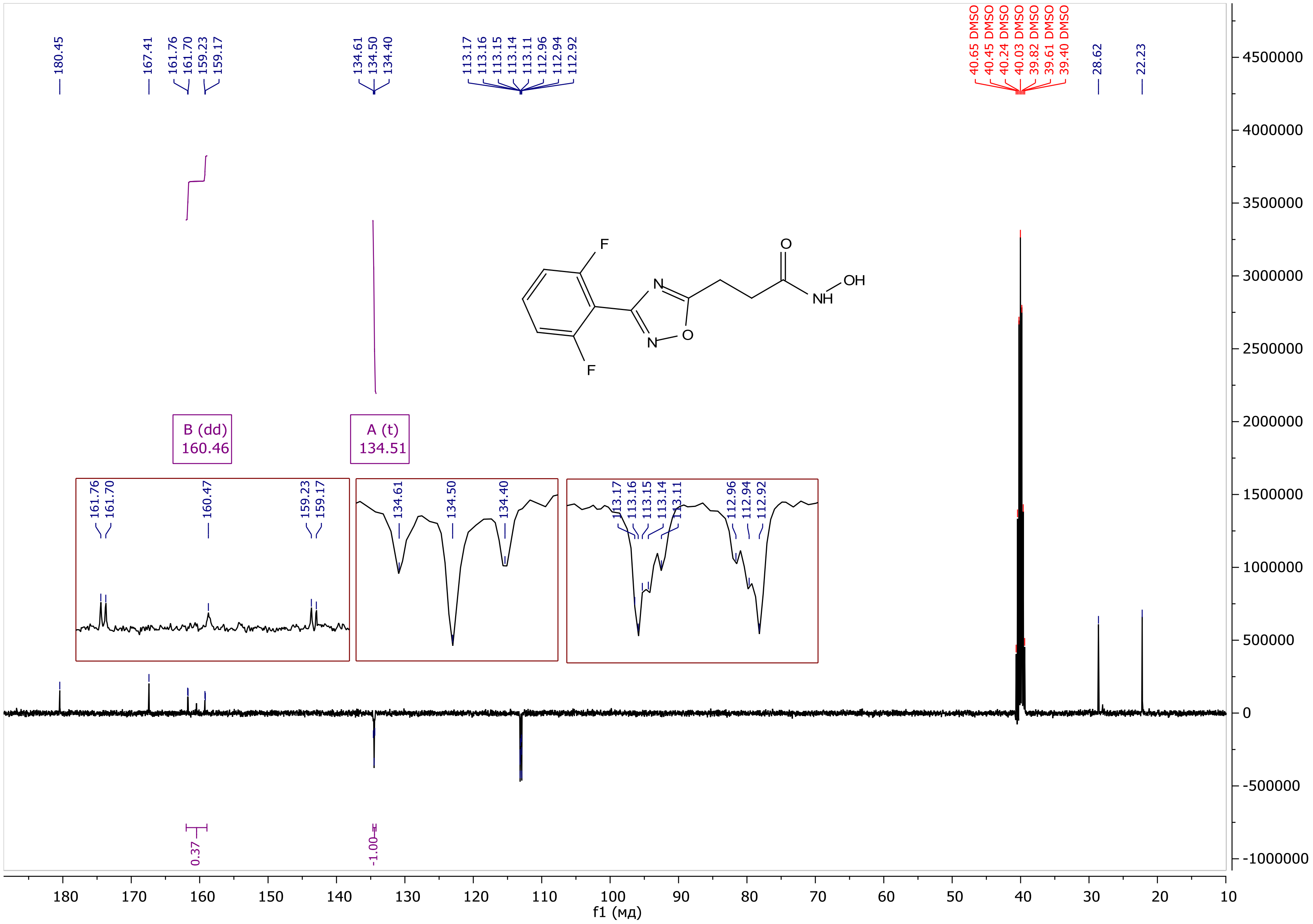

^{1}H NMR spectrum of compound **17b**

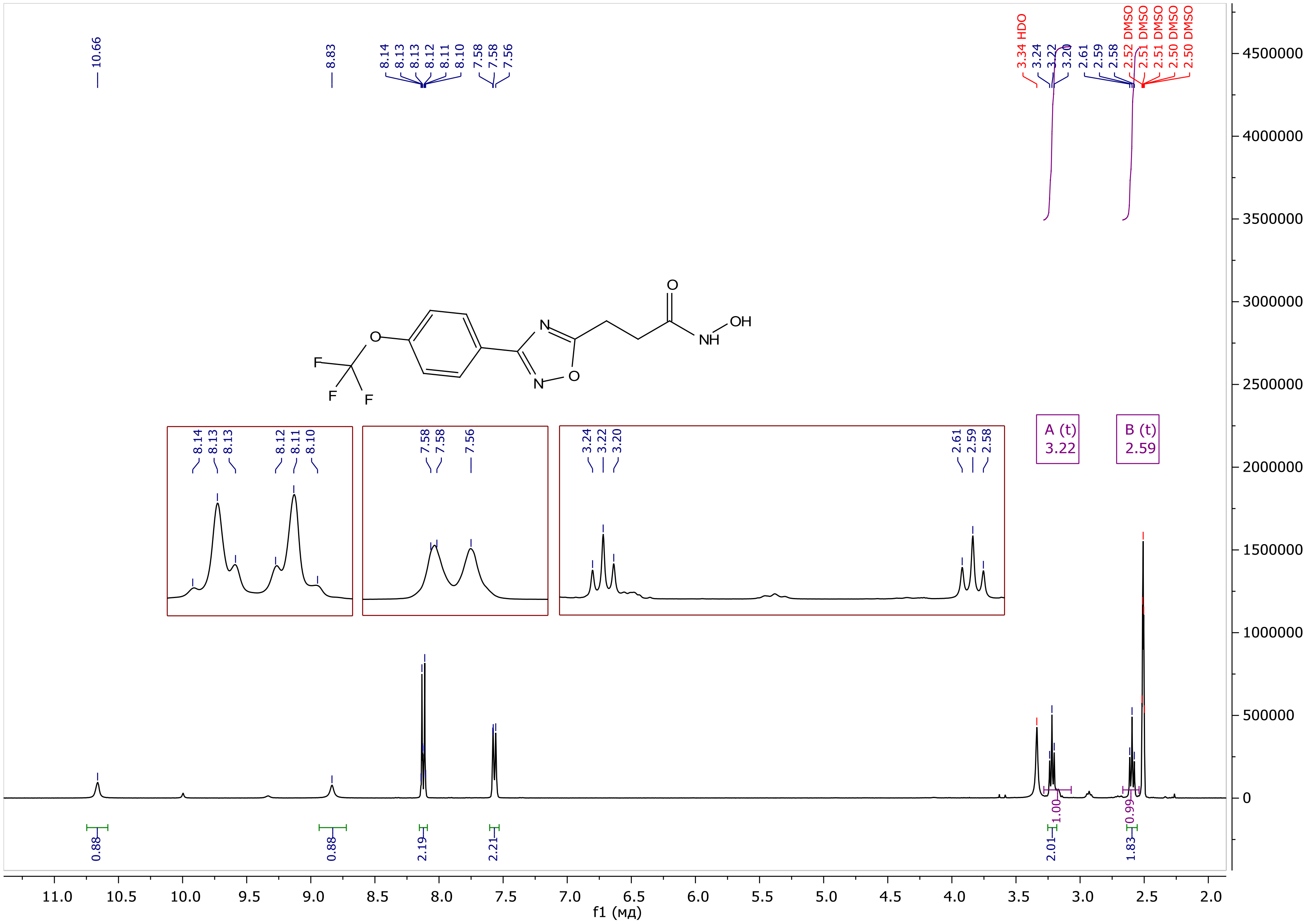

^{13}C NMR spectrum of compound **17b**

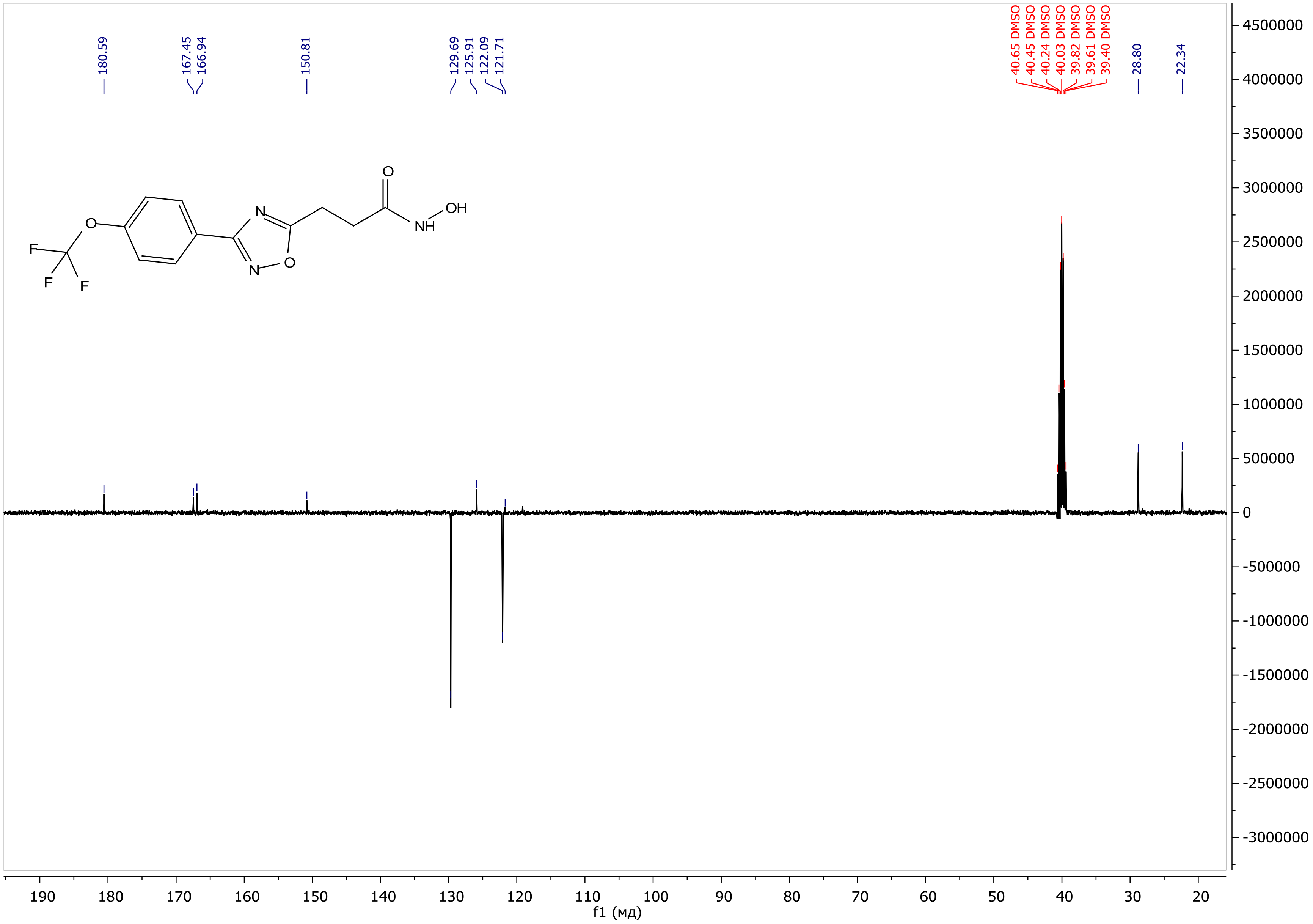

^{1}H NMR spectrum of compound **17c**

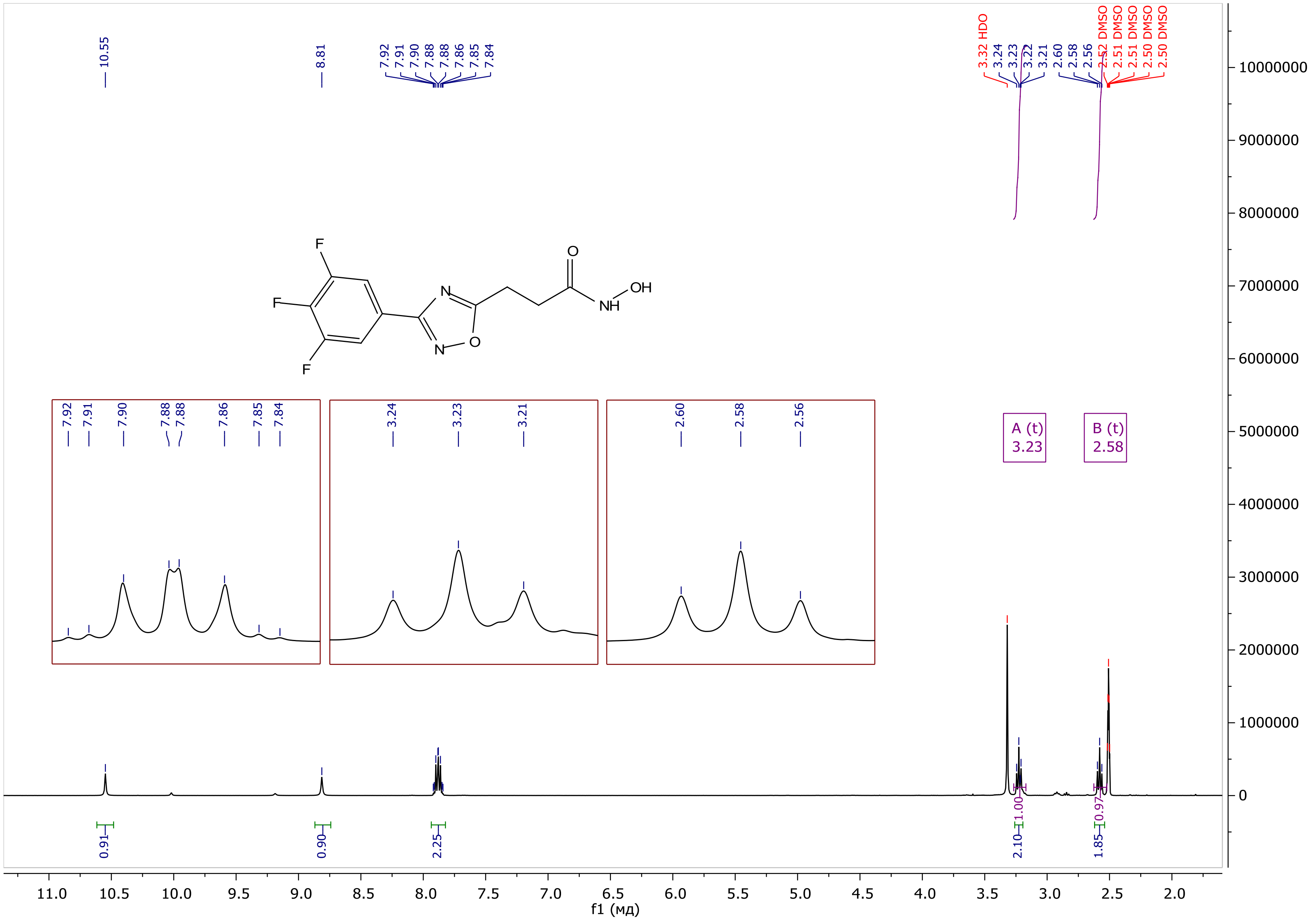

^{13}C NMR spectrum of compound **17c**

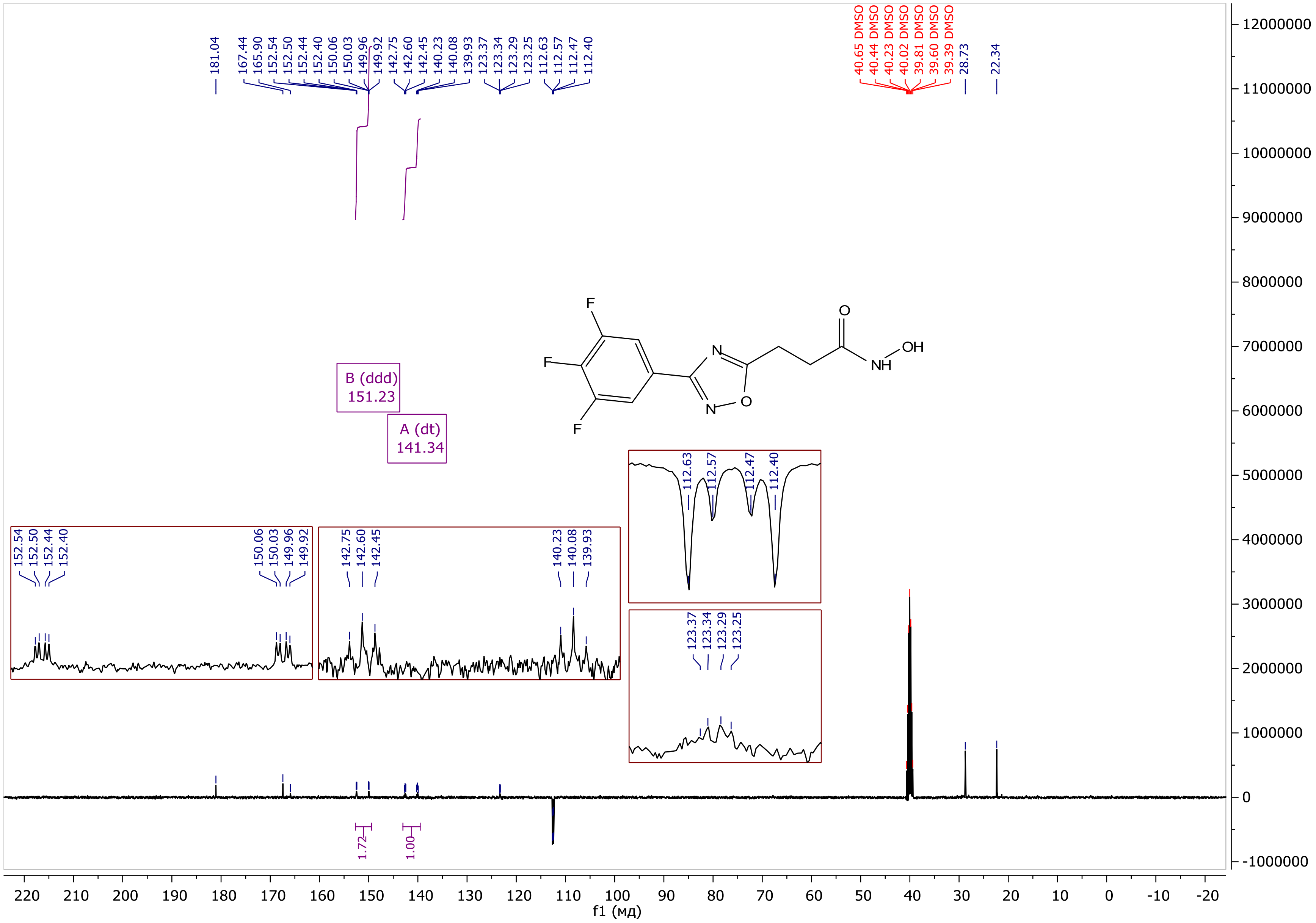

^{1}H NMR spectrum of compound **17d**

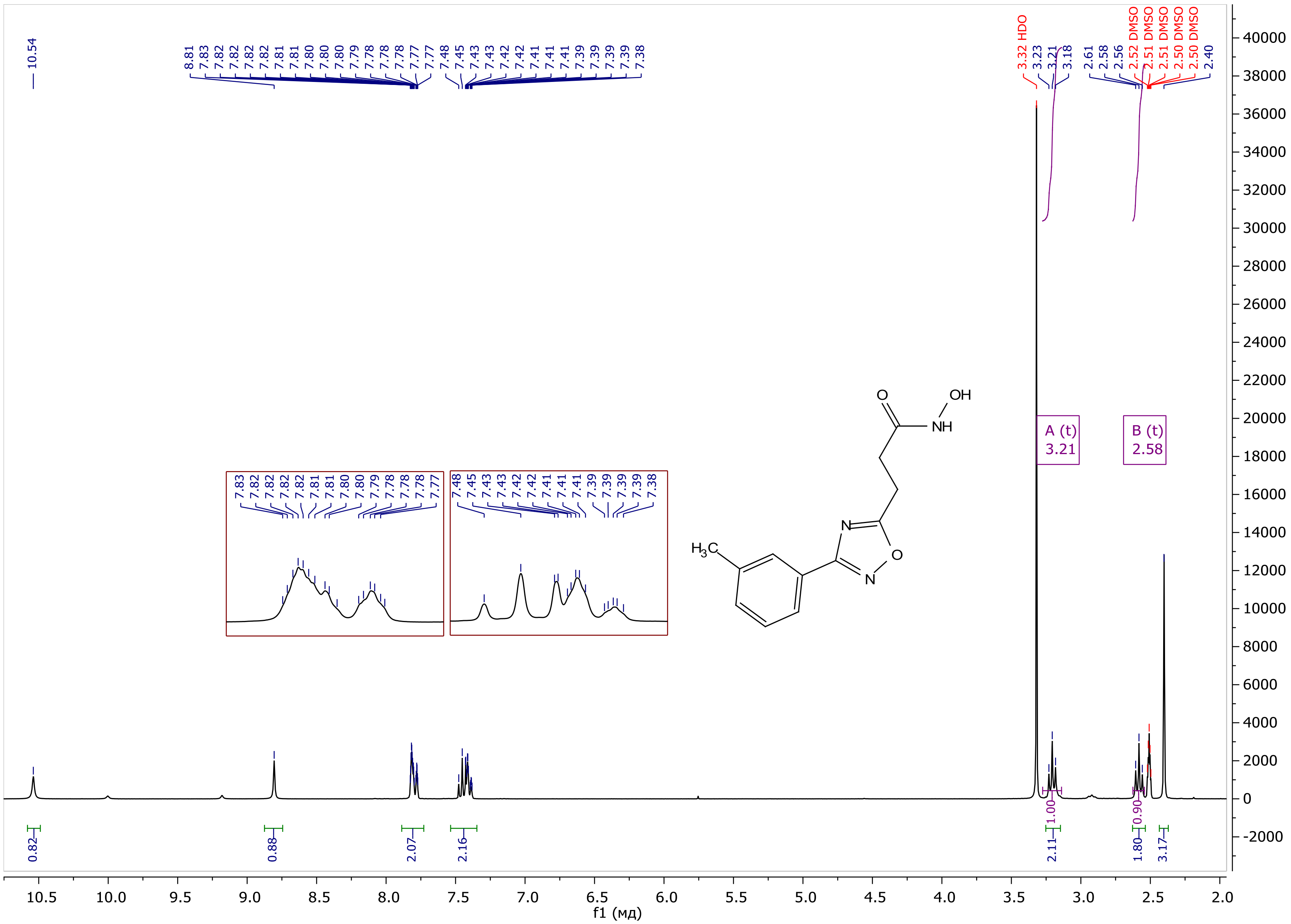

^{13}C NMR spectrum of compound **17d**

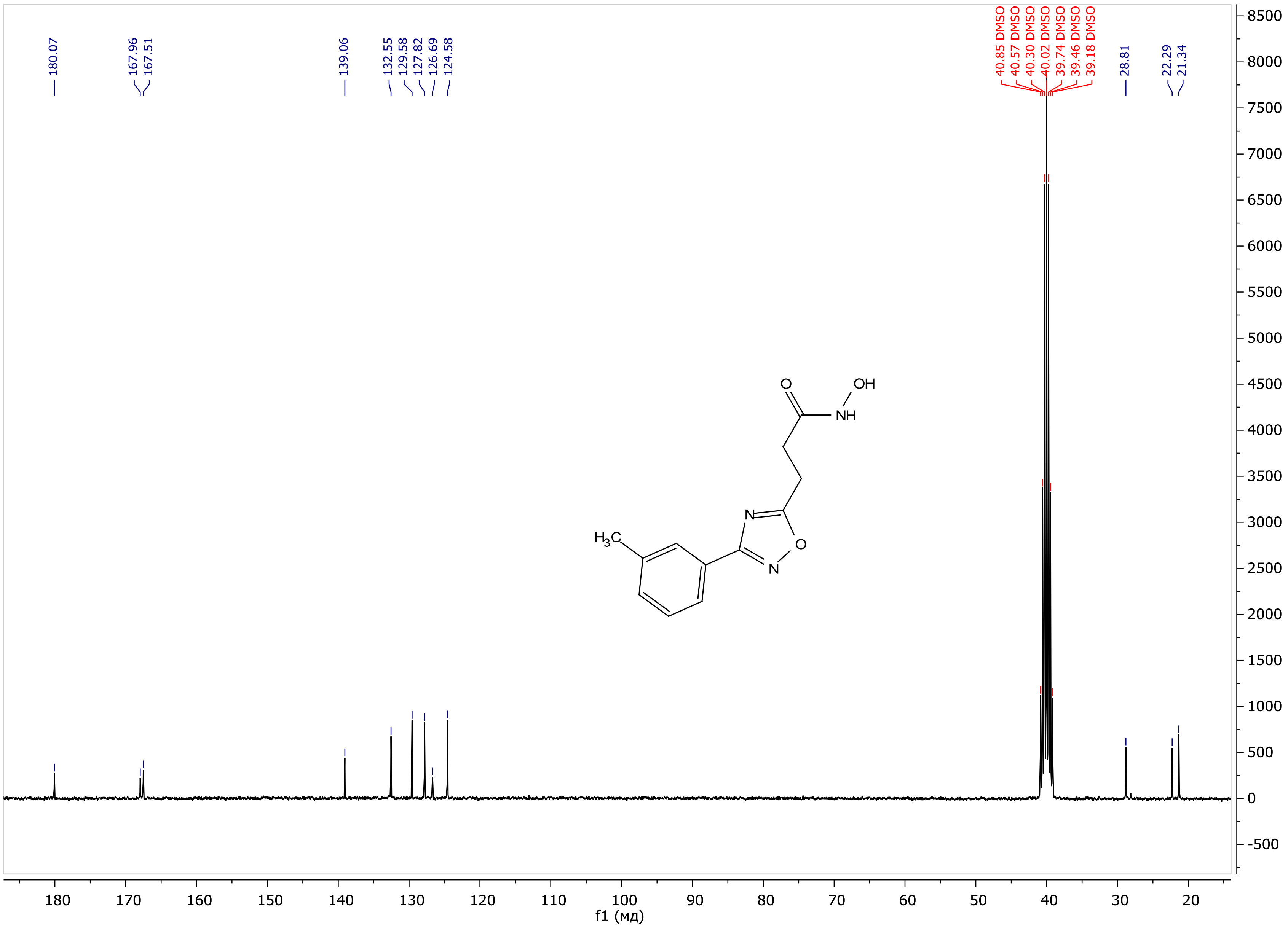

^{1}H, ^{13}C NMR spectra for ethyl 5-aryl-1,3,4-oxadiazole-2-carboxylates synthesized

^{1}H NMR spectrum of compound **29f**

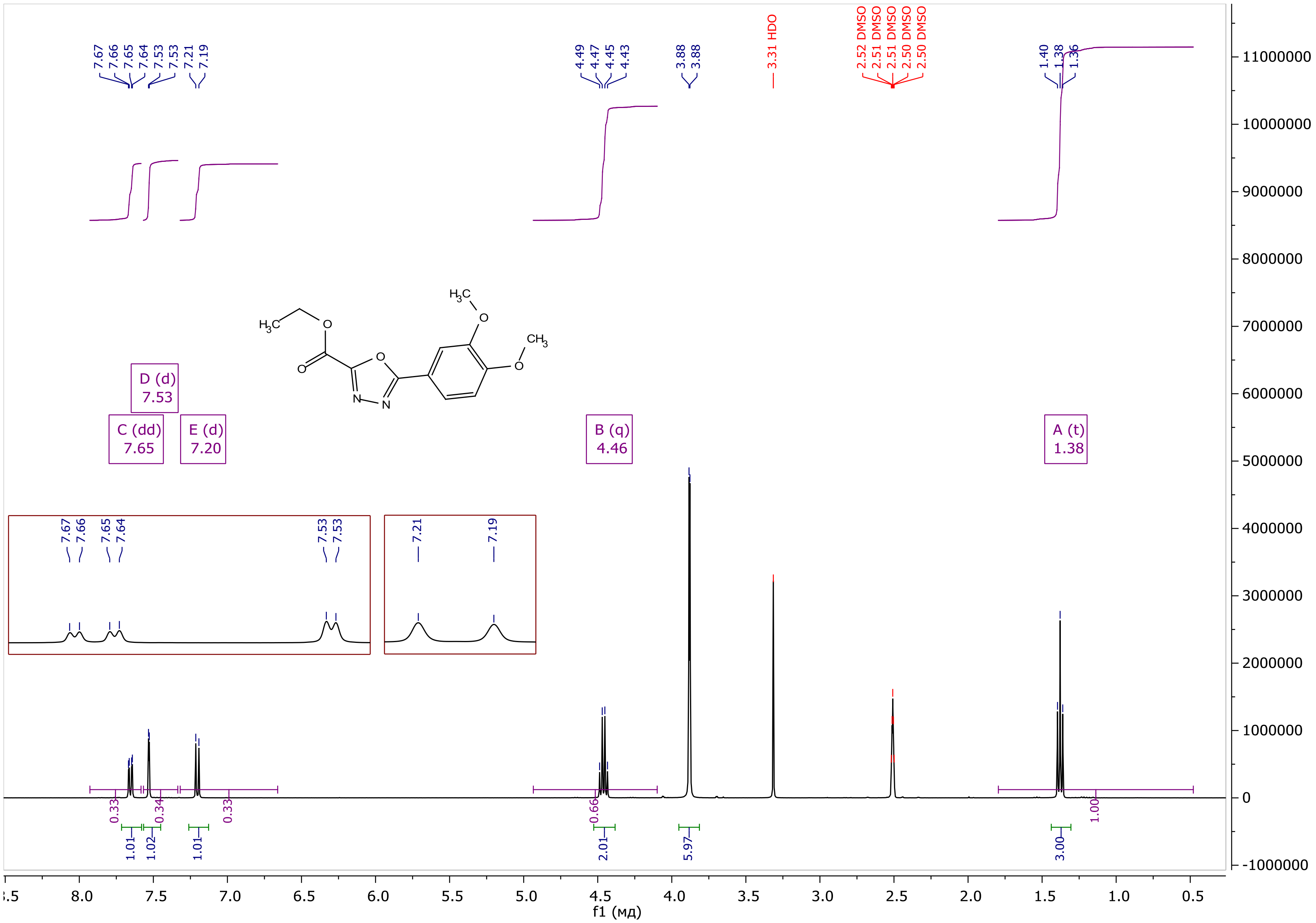

^{13}C NMR spectrum of compound **29f**

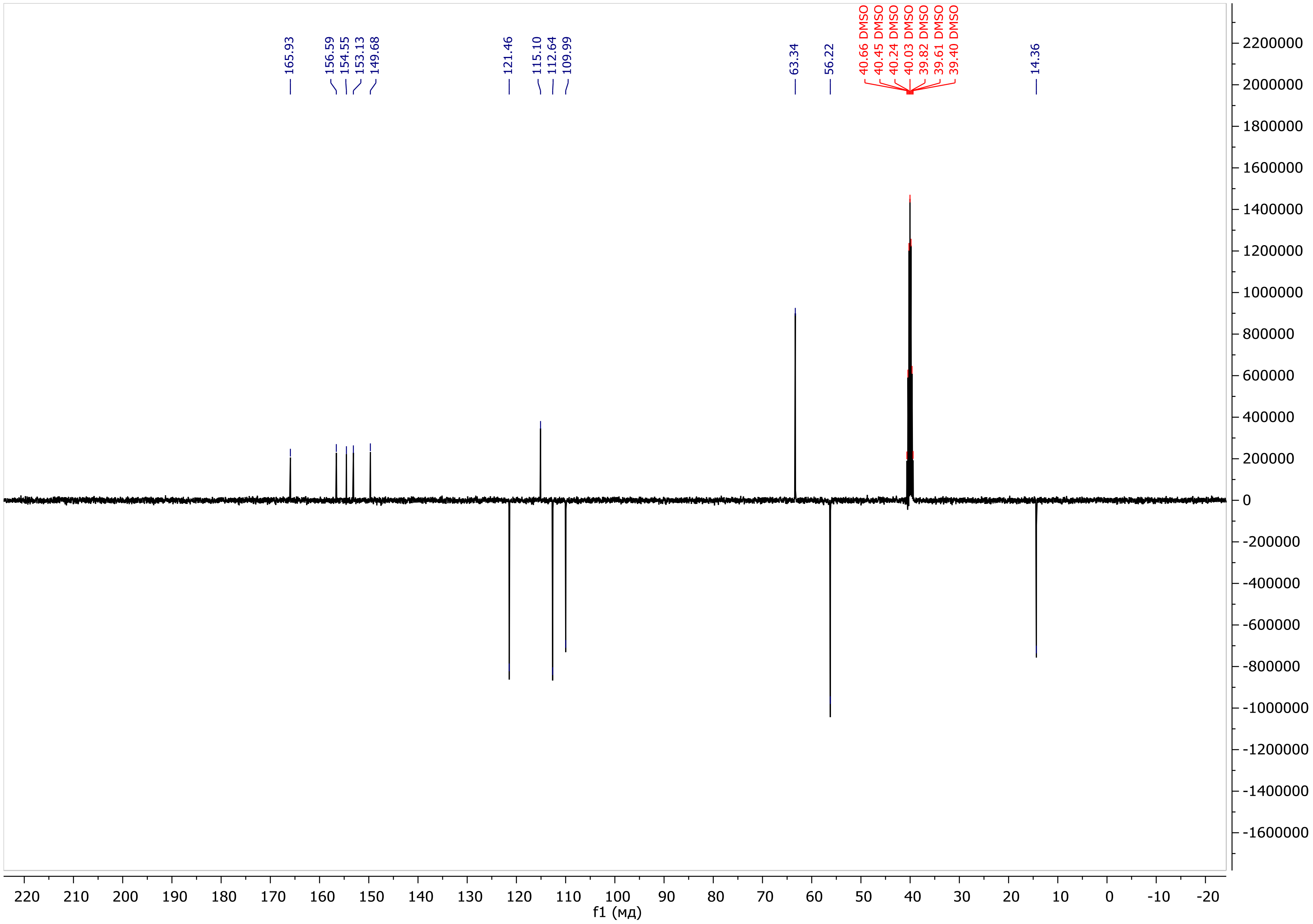

^{1}H NMR spectrum of compound **29e**

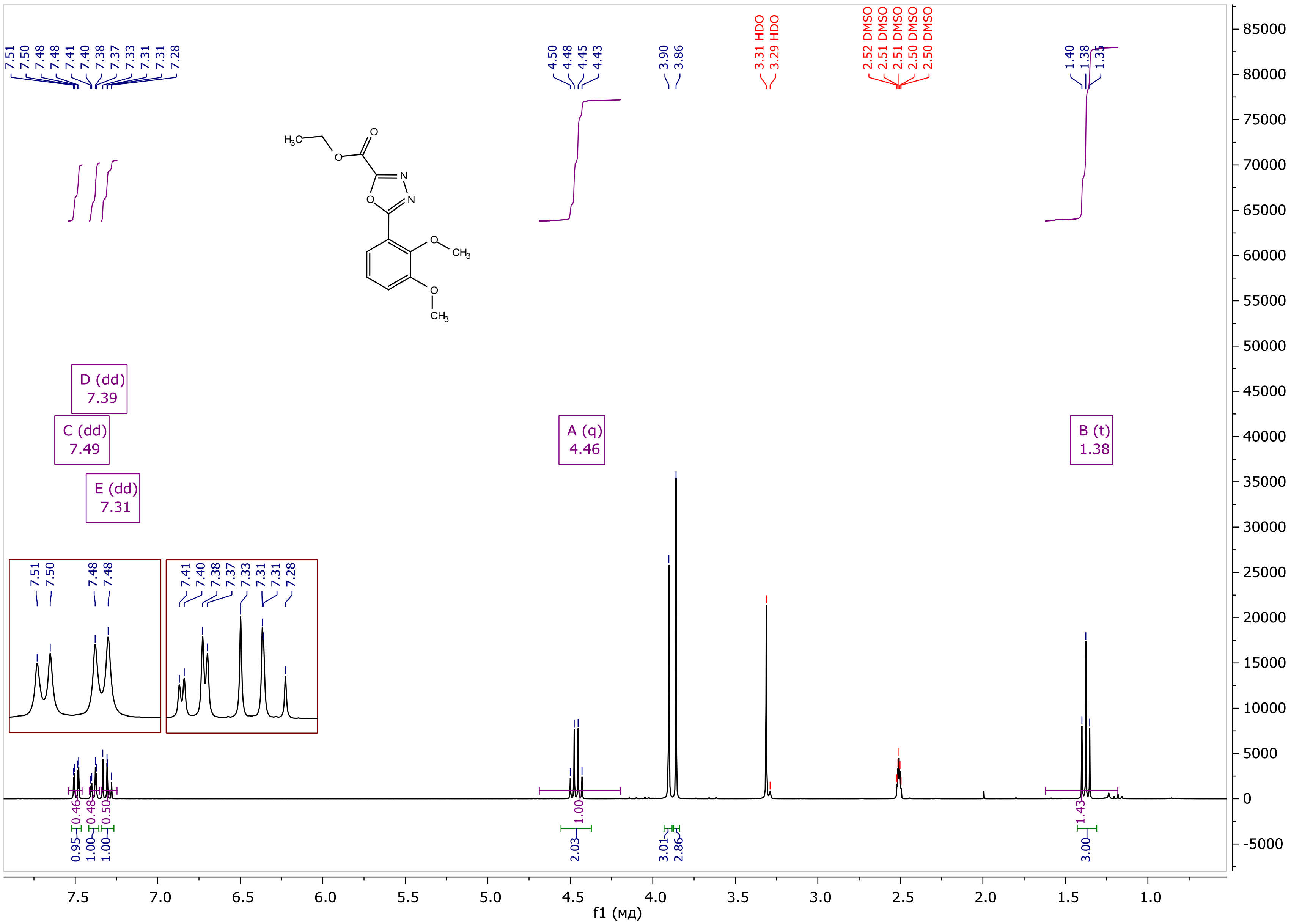

^{13}C NMR spectrum of compound **29e**

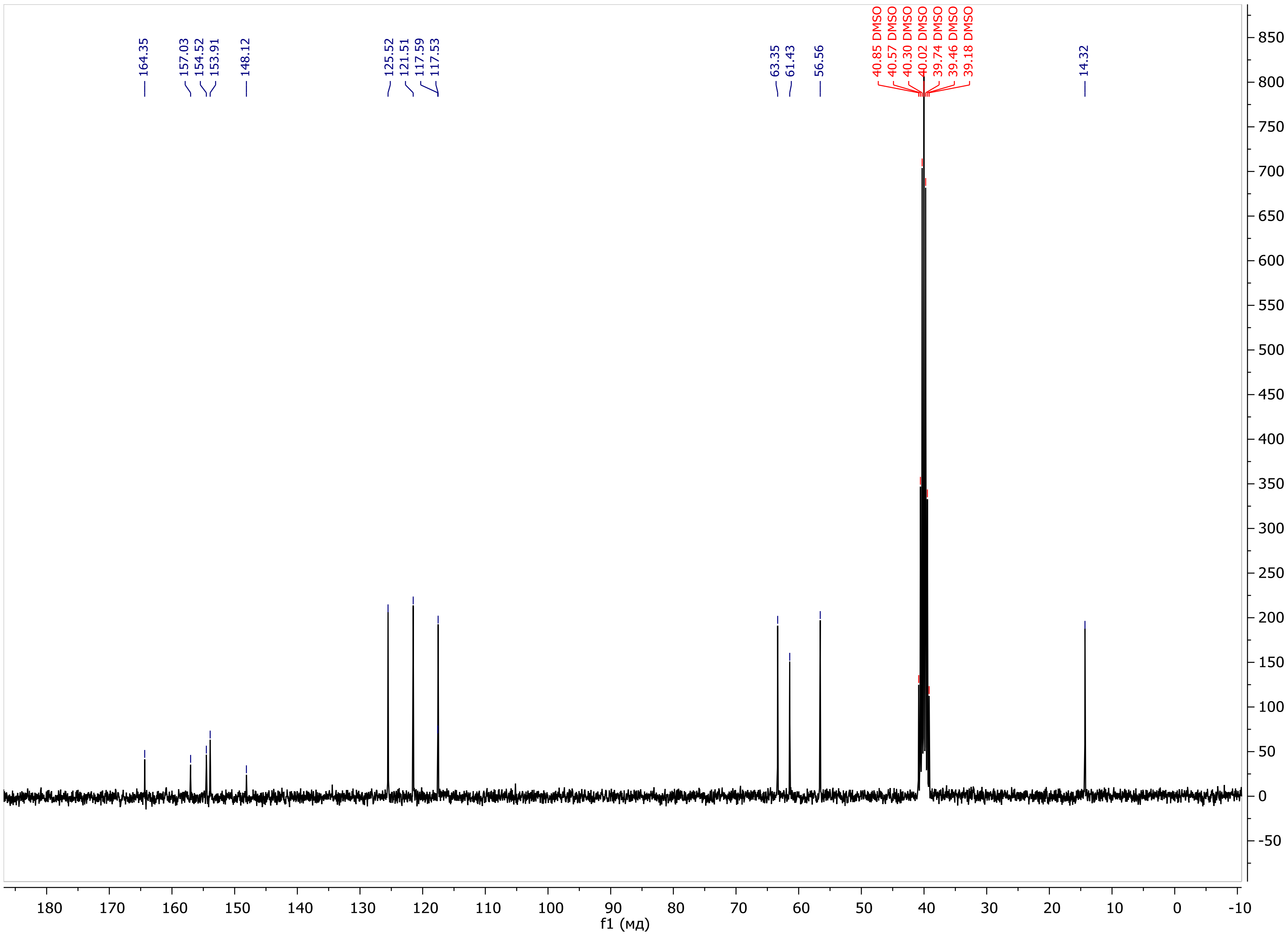

^{1}H NMR spectrum of compound **35**

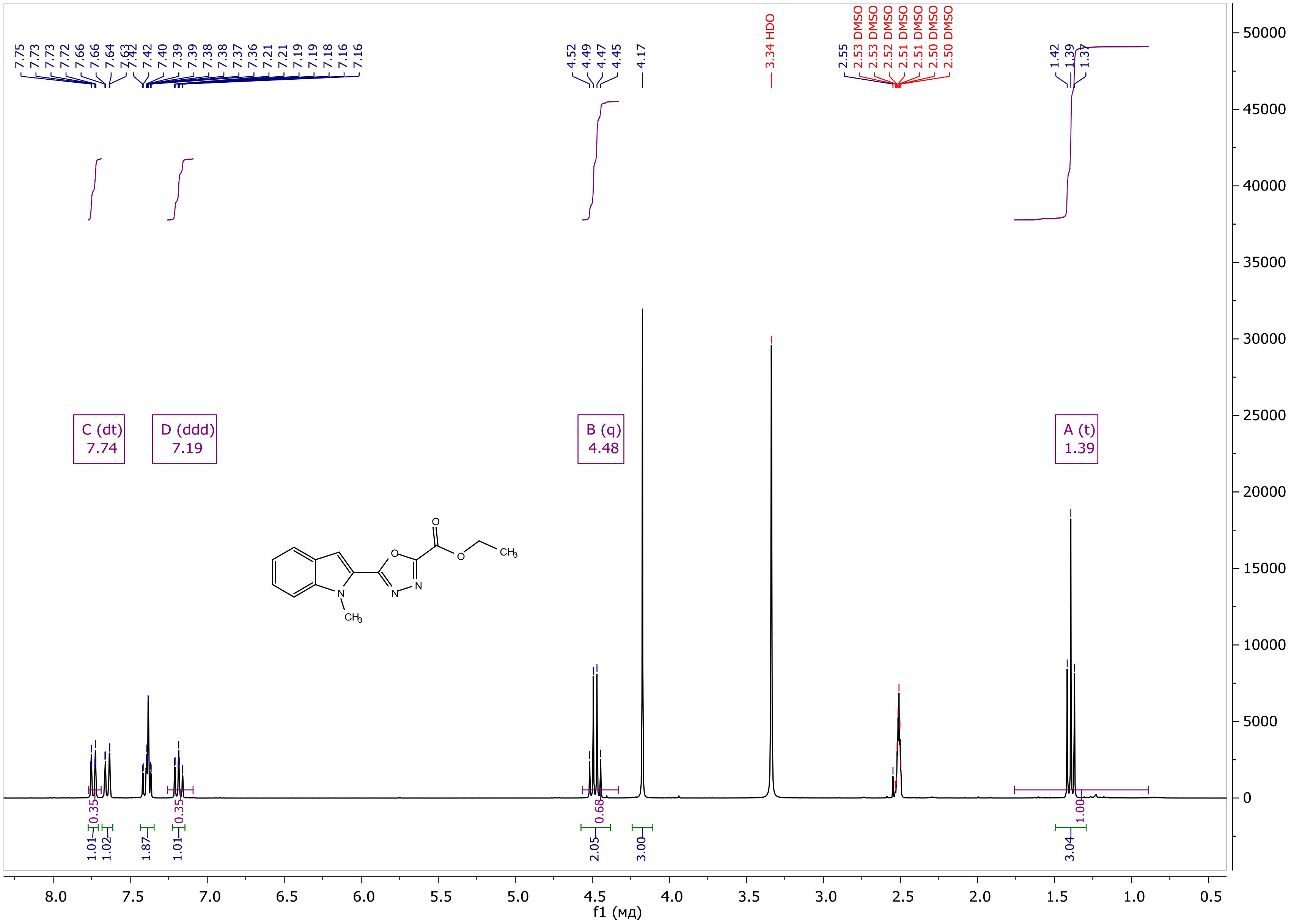

^{13}C NMR spectrum of compound **35**

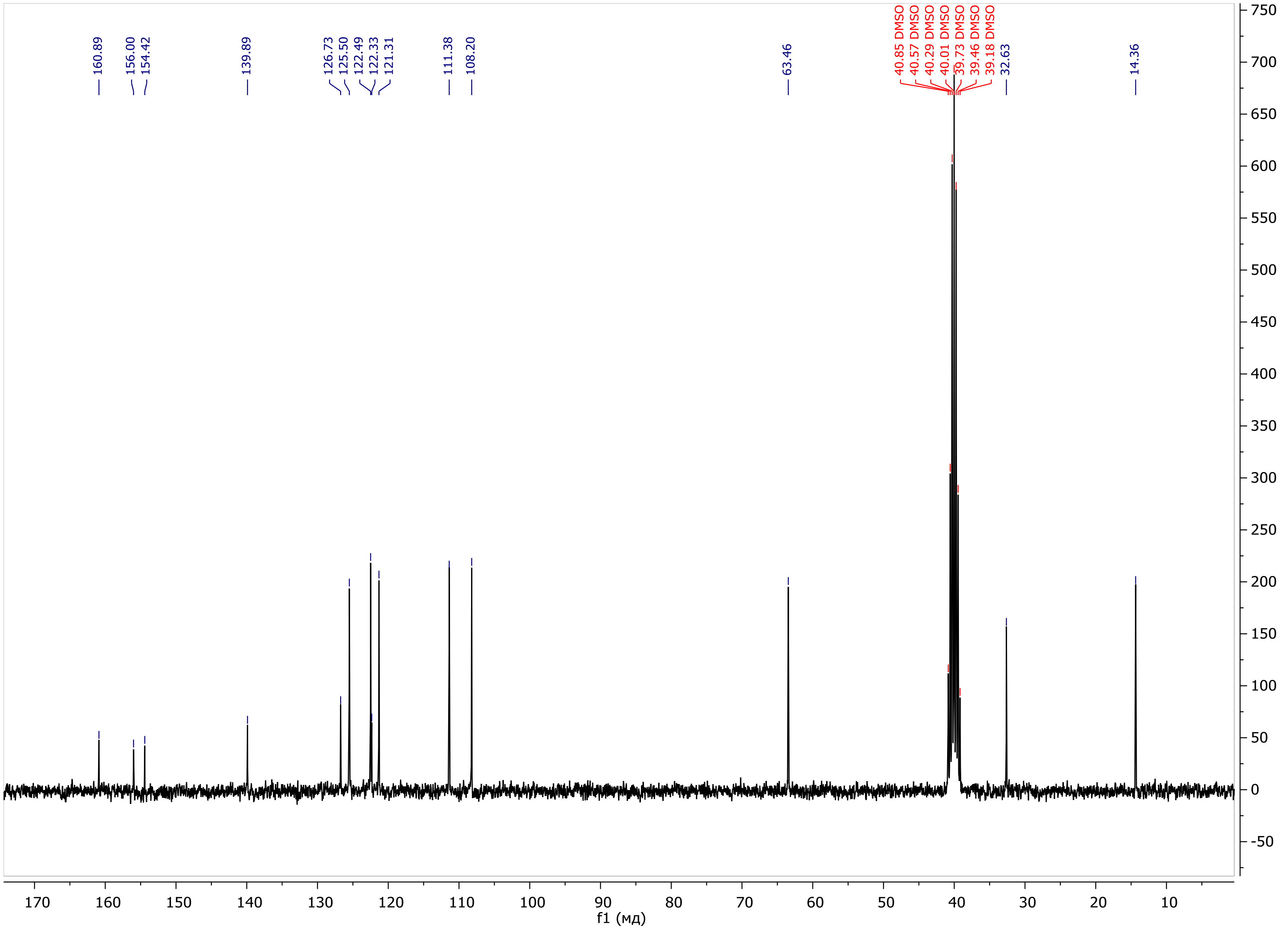

^{1}H NMR spectrum of compound **29c**

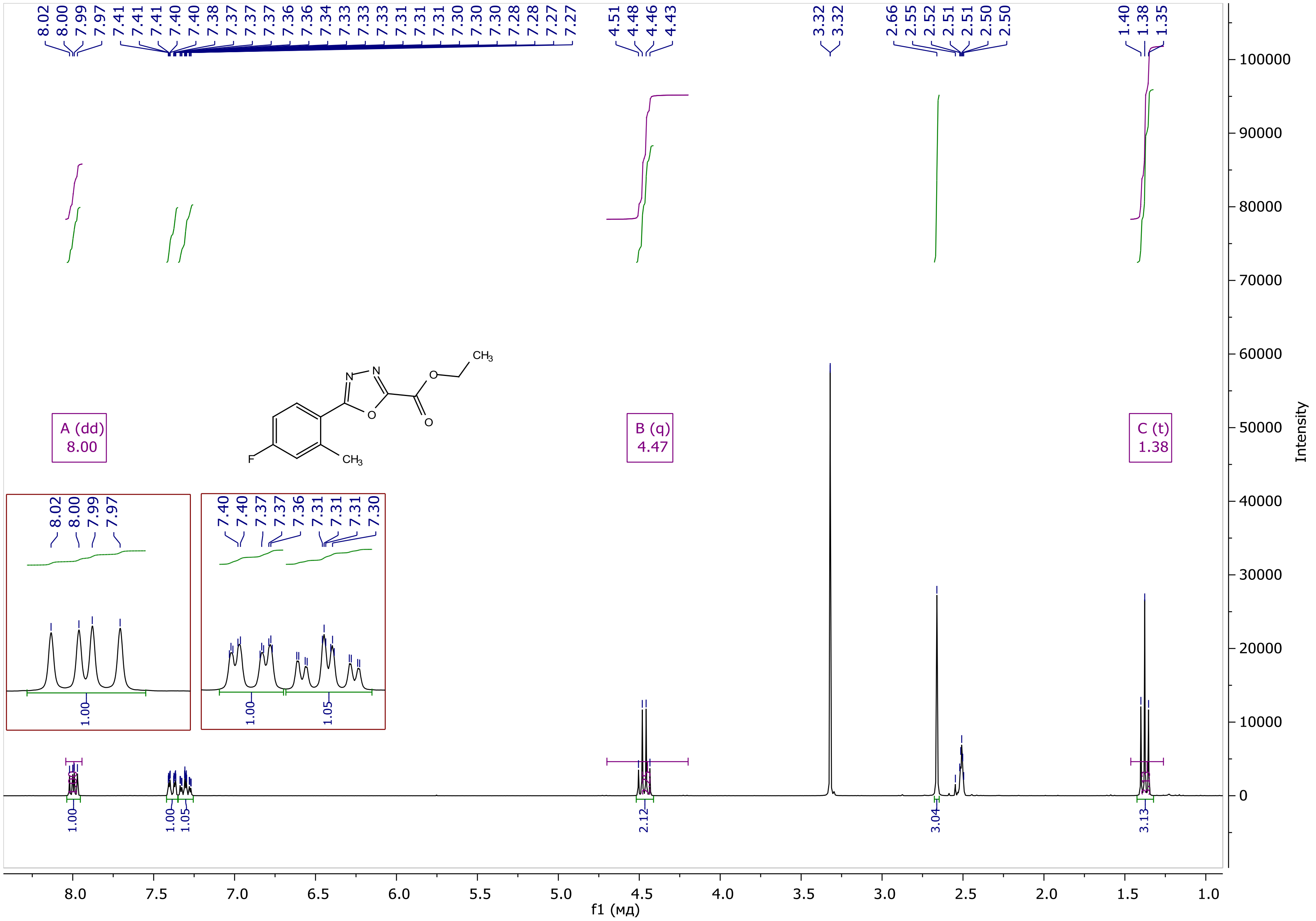

^{13}C NMR spectrum of compound **29c**

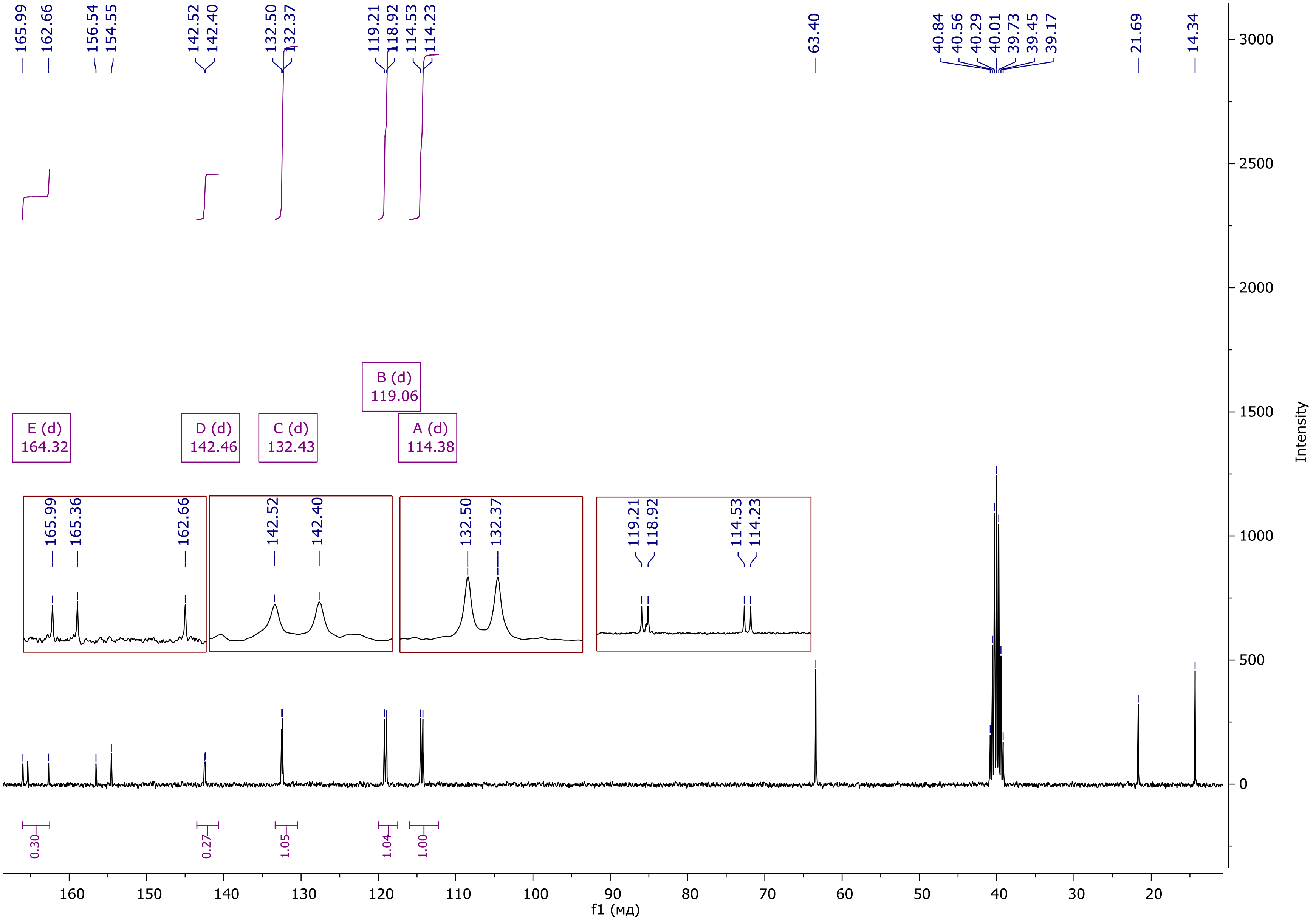

^{1}H NMR spectrum of compound **29b**

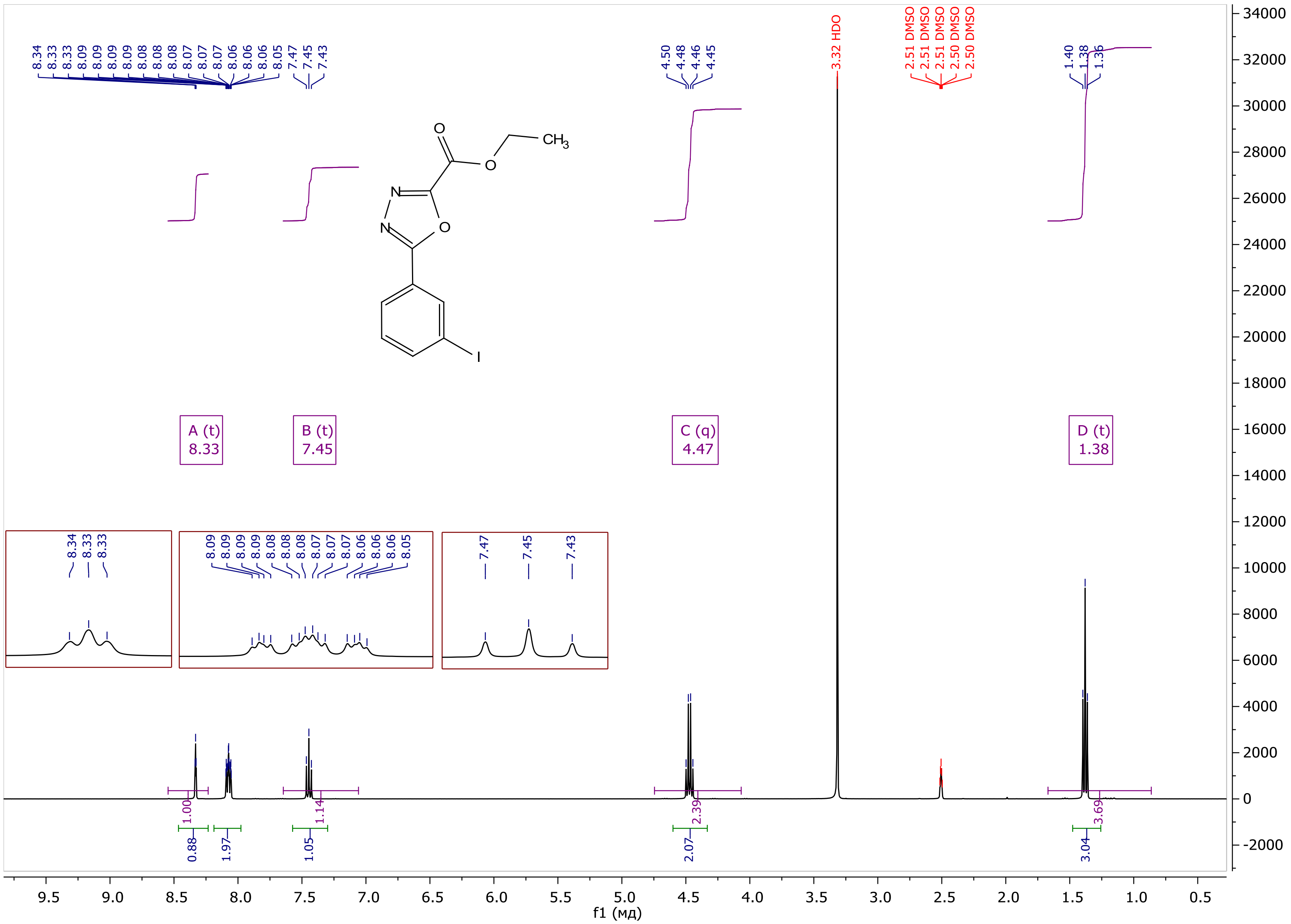

^{13}C NMR spectrum of compound **29b**

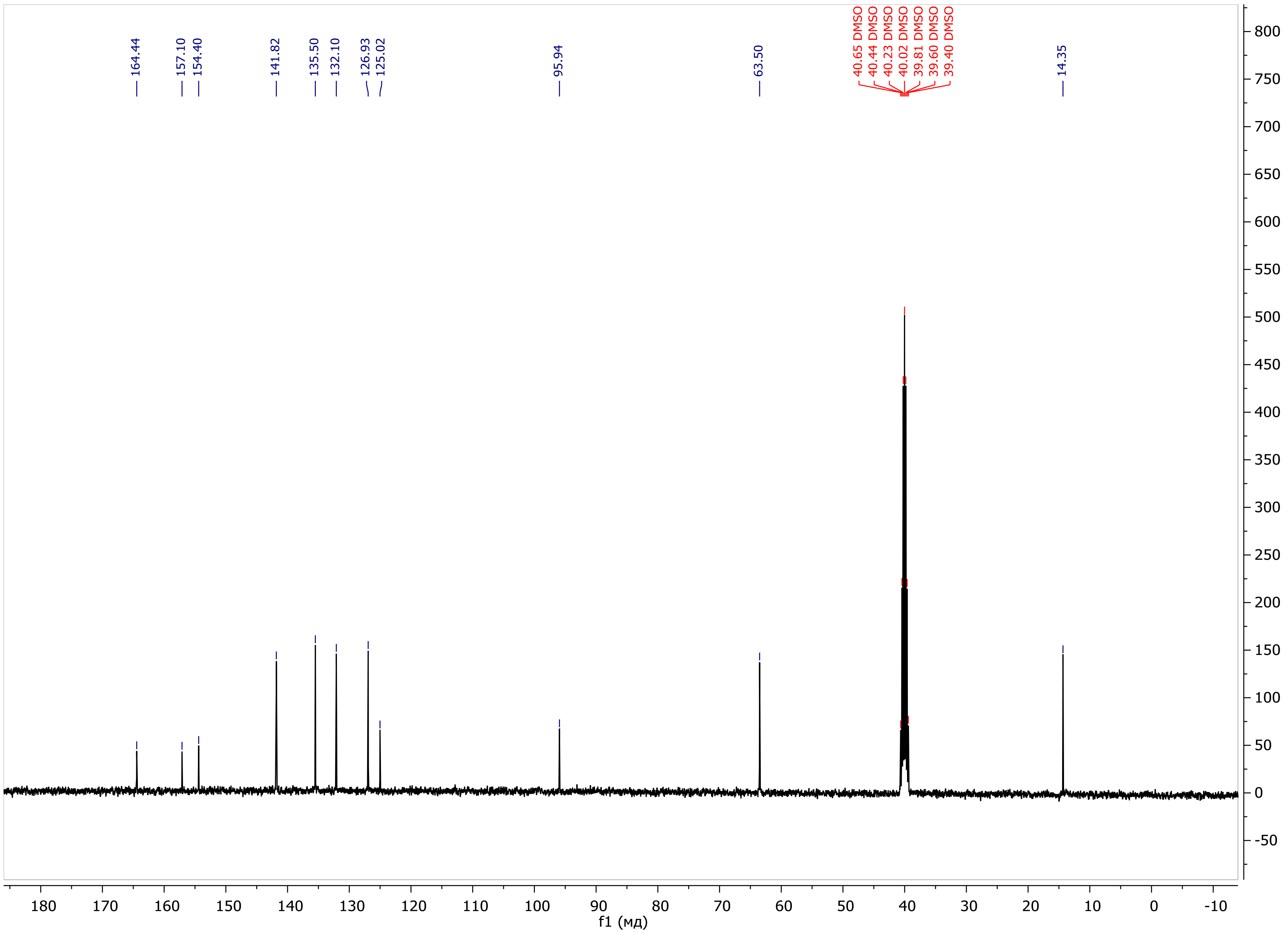

^{1}H, ^{13}C NMR spectra for *N*-Hydroxy-5-(3-iodophenyl)-1,3,4-oxadiazole-2-carboxamides synthesized

^{1}H NMR spectrum of compound **30f**

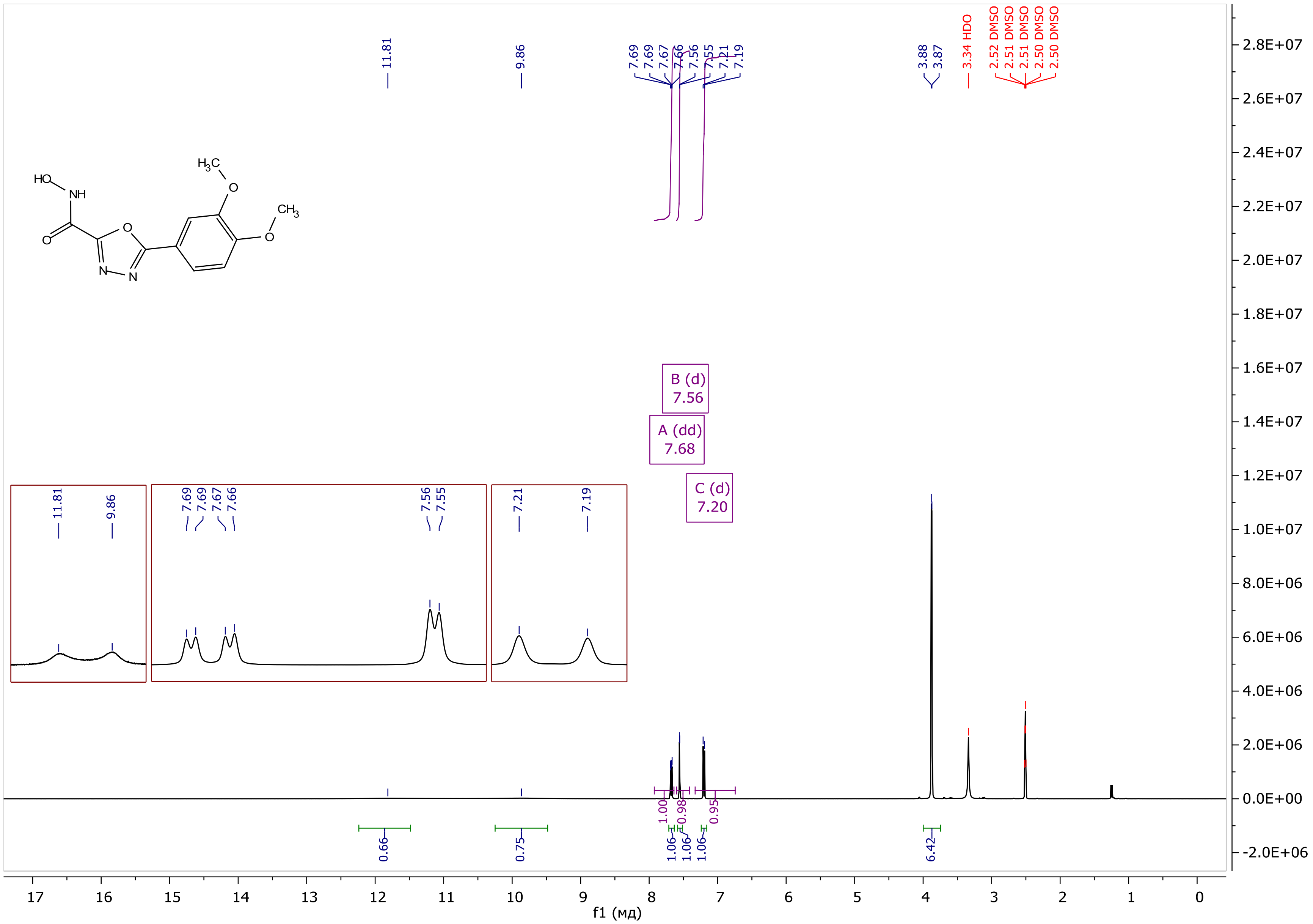

^{13}C NMR spectrum of compound **30f**

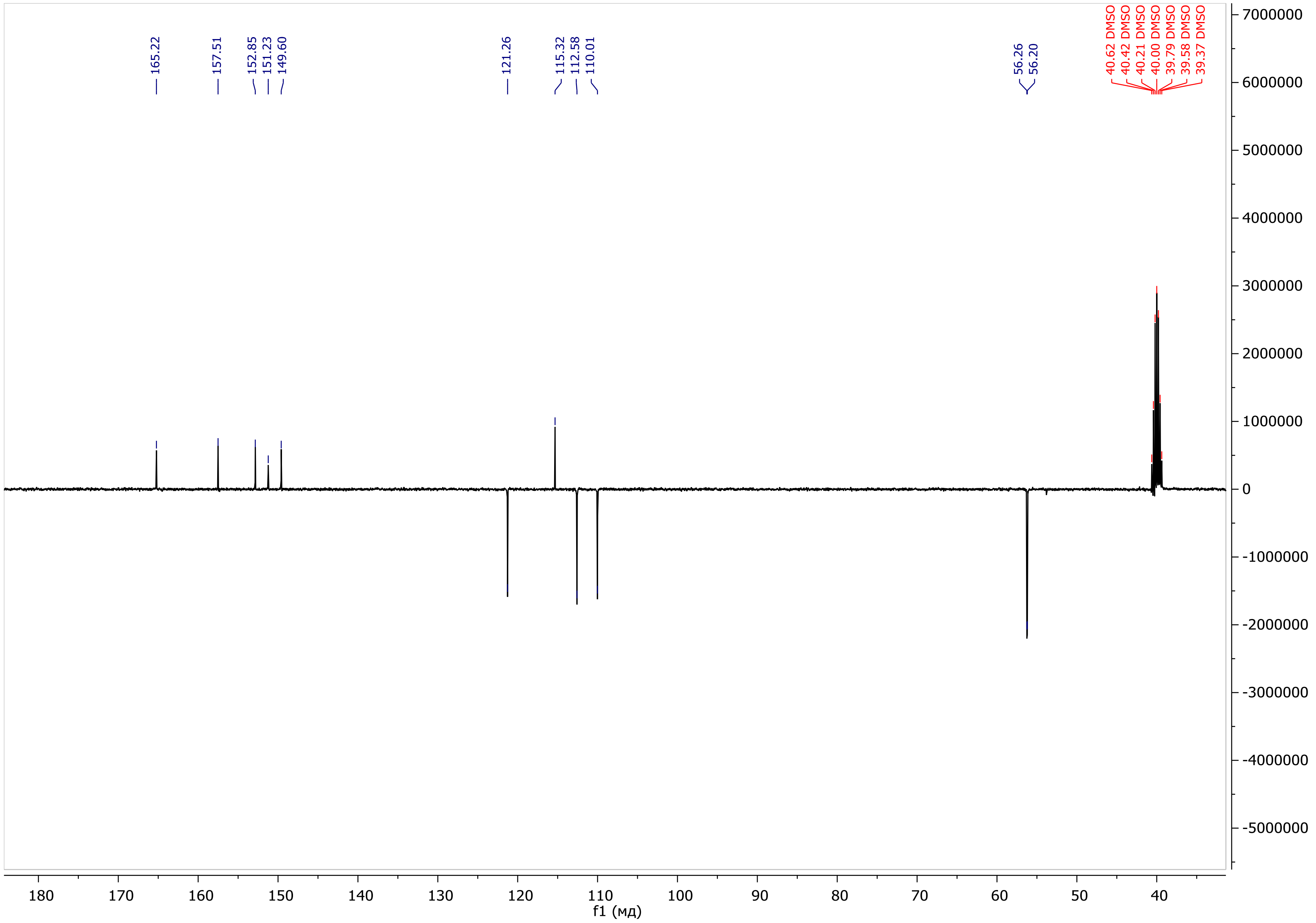

^{1}H NMR spectrum of compound **30d**

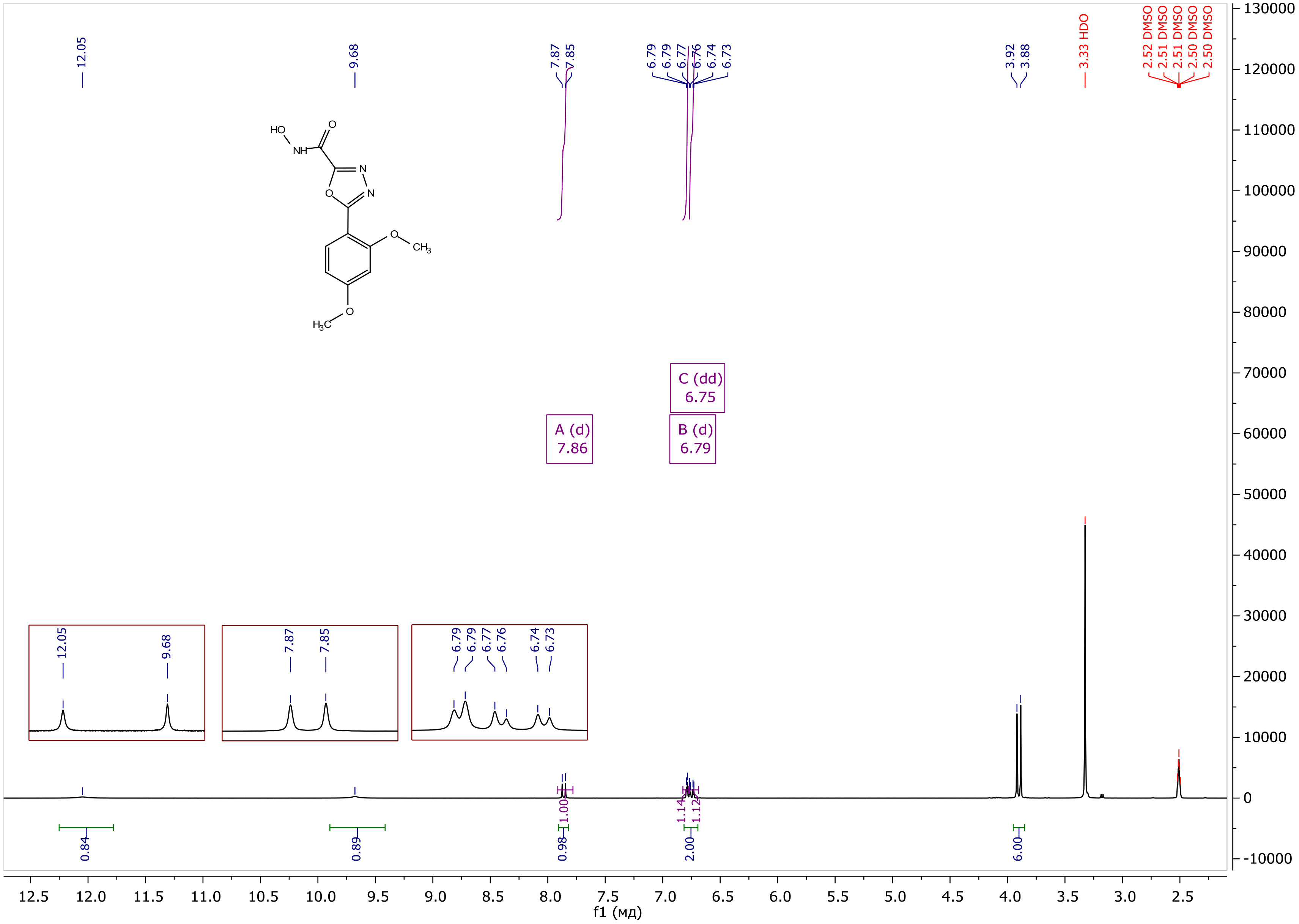

^{13}C NMR spectrum of compound **30d**

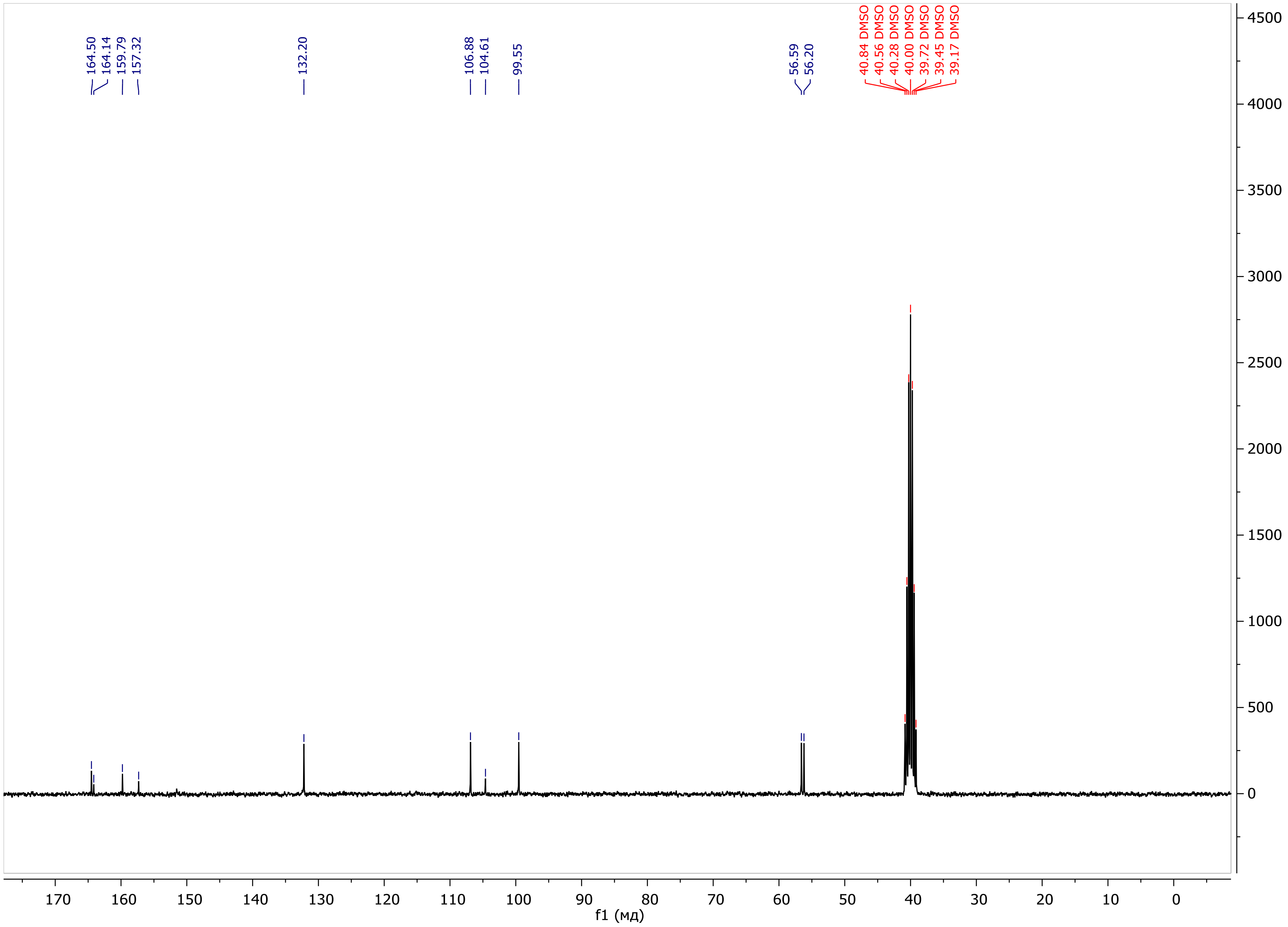

^{1}H NMR spectrum of compound **30e**

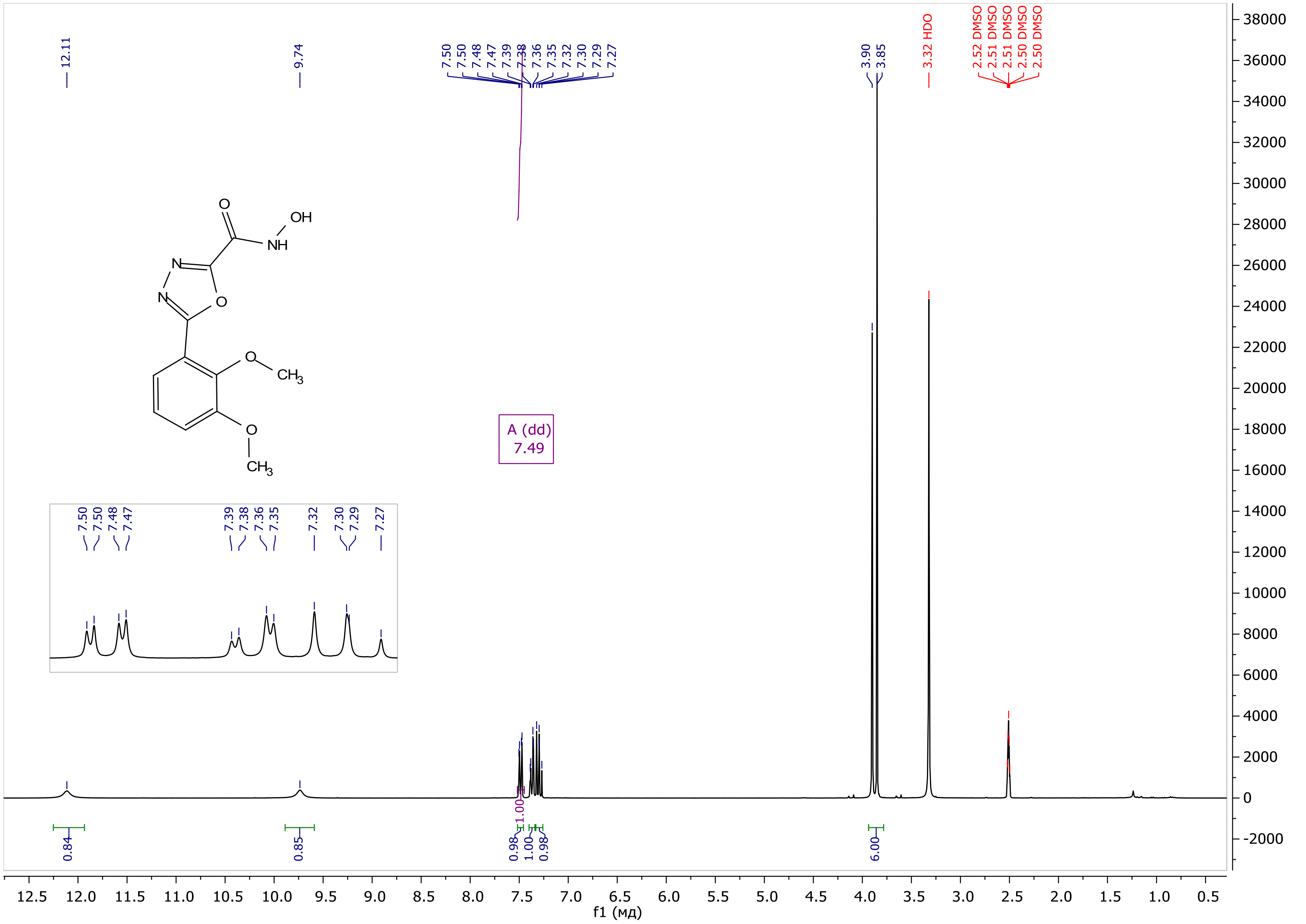

^{13}C NMR spectrum of compound **30e**

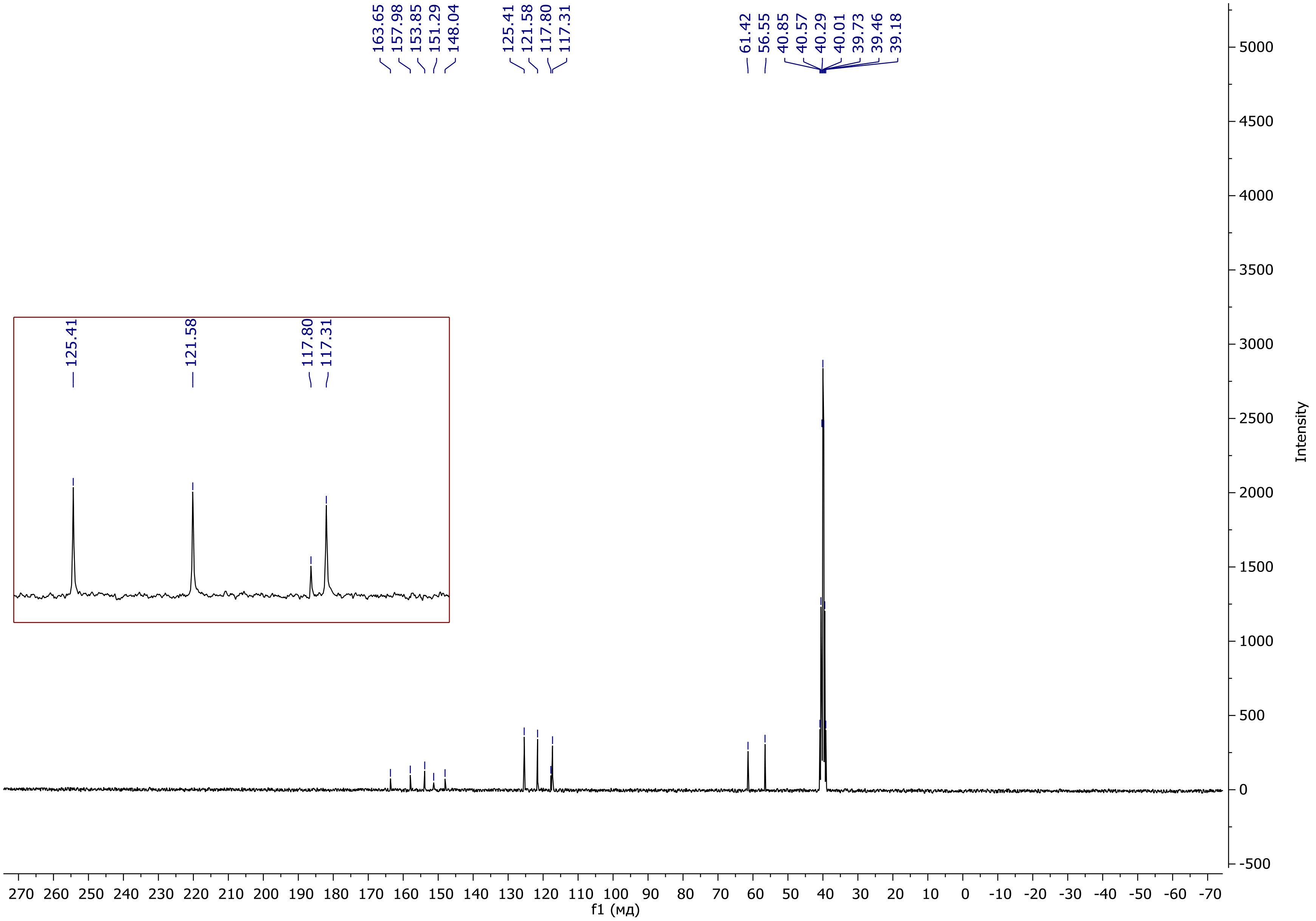

^{1}H NMR spectrum of compound **36**

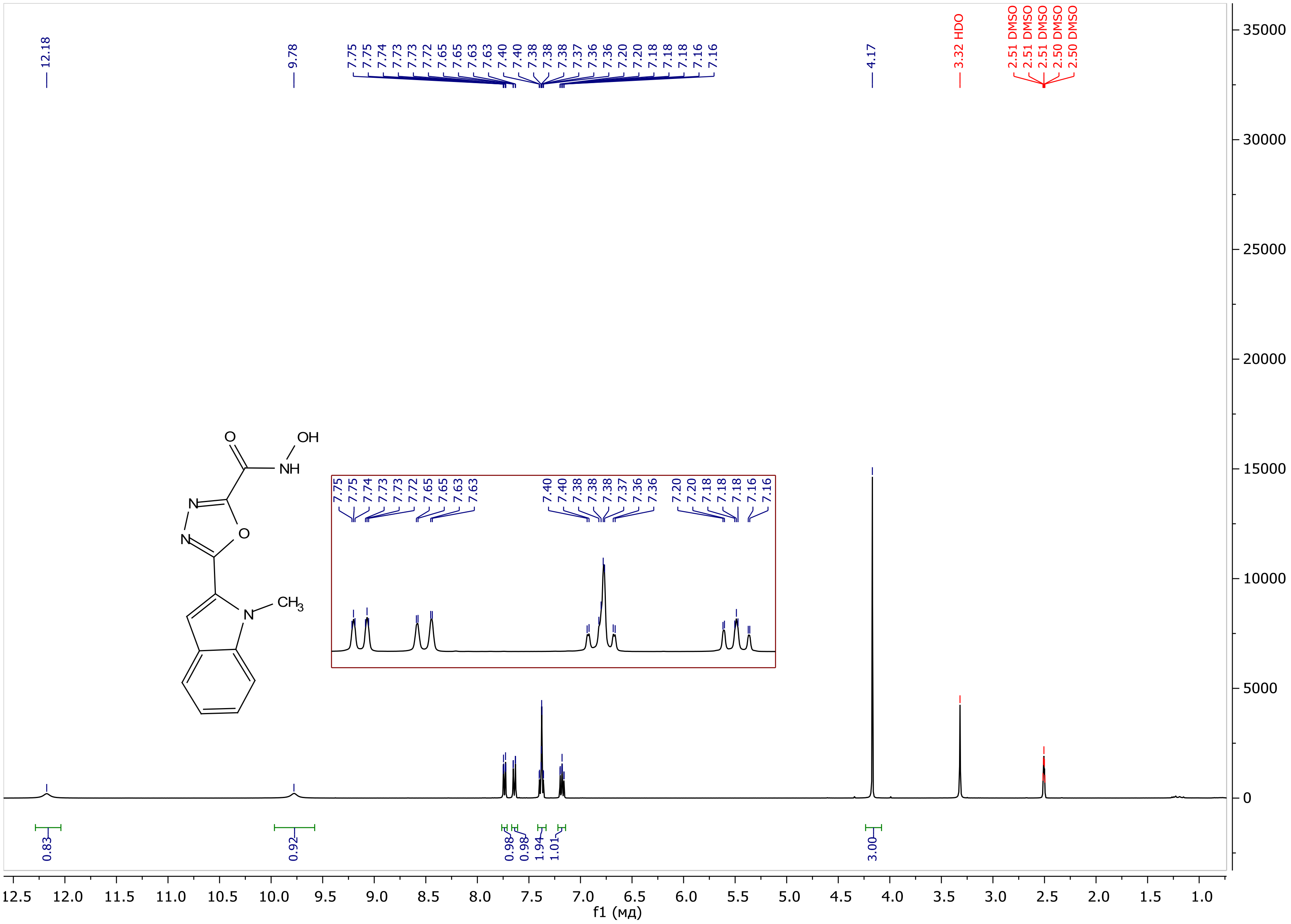

^{13}C NMR spectrum of compound **36**

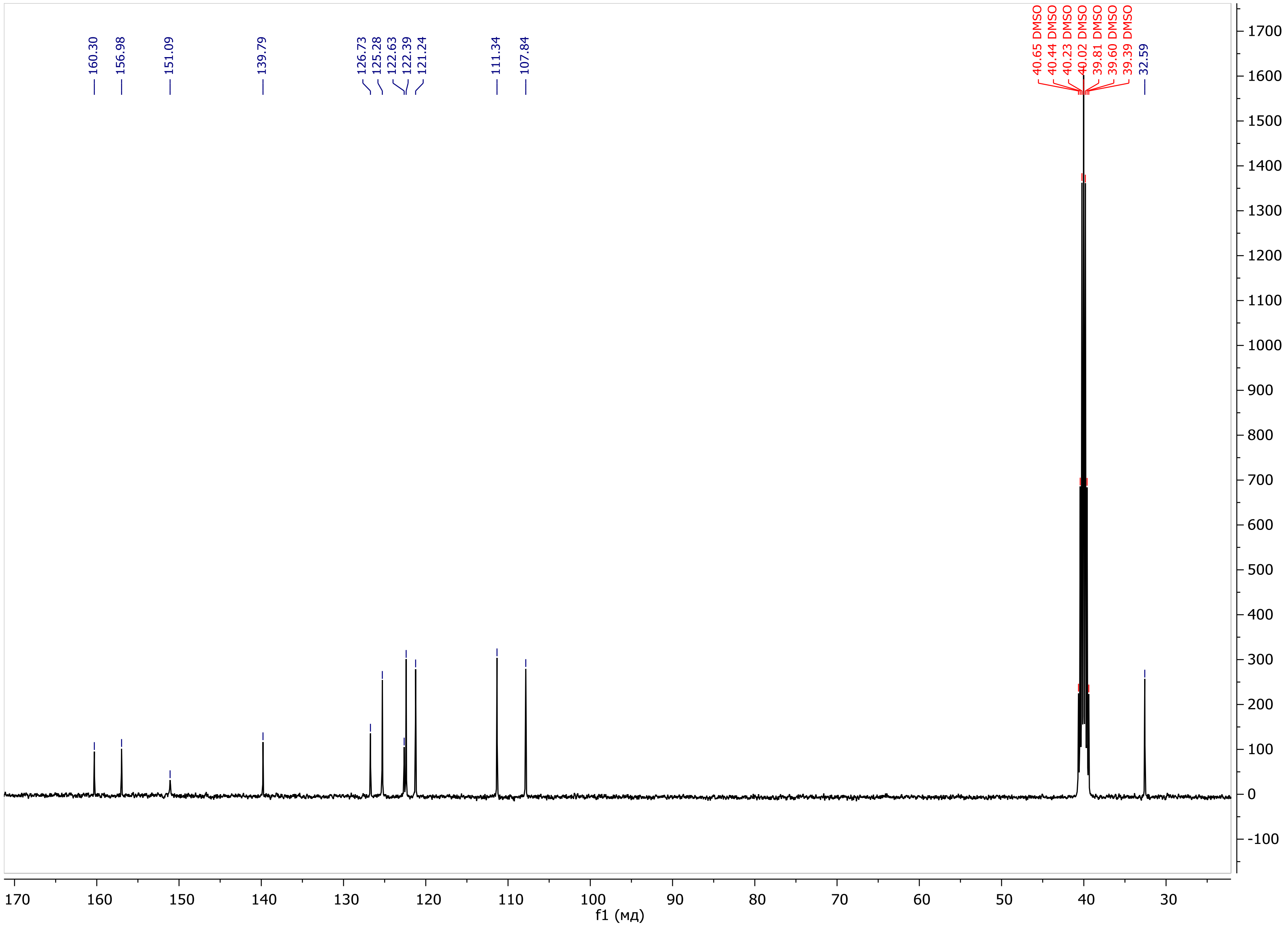

^{1}H NMR spectrum of compound **30a**

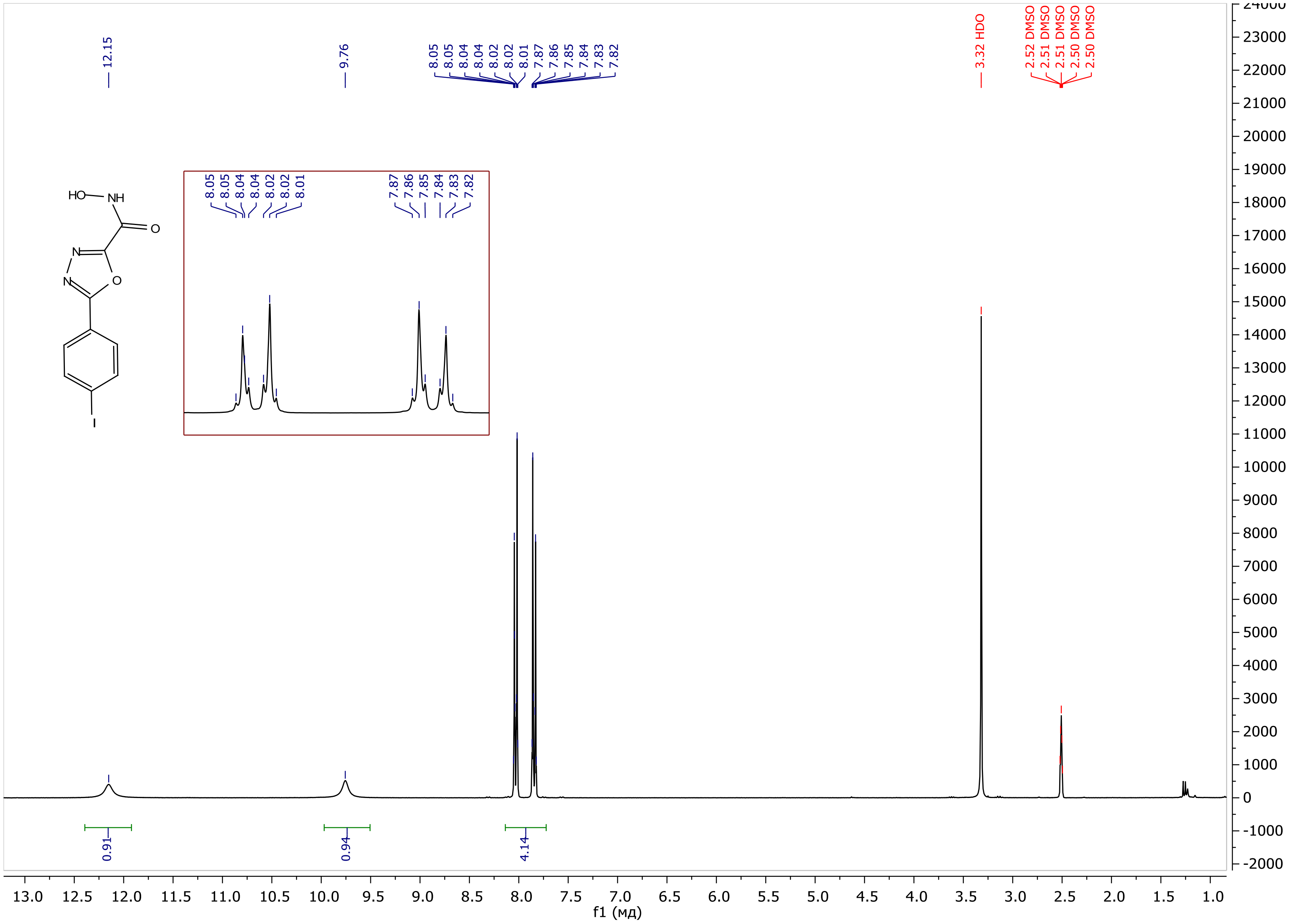

^{13}C NMR spectrum of compound **30a**

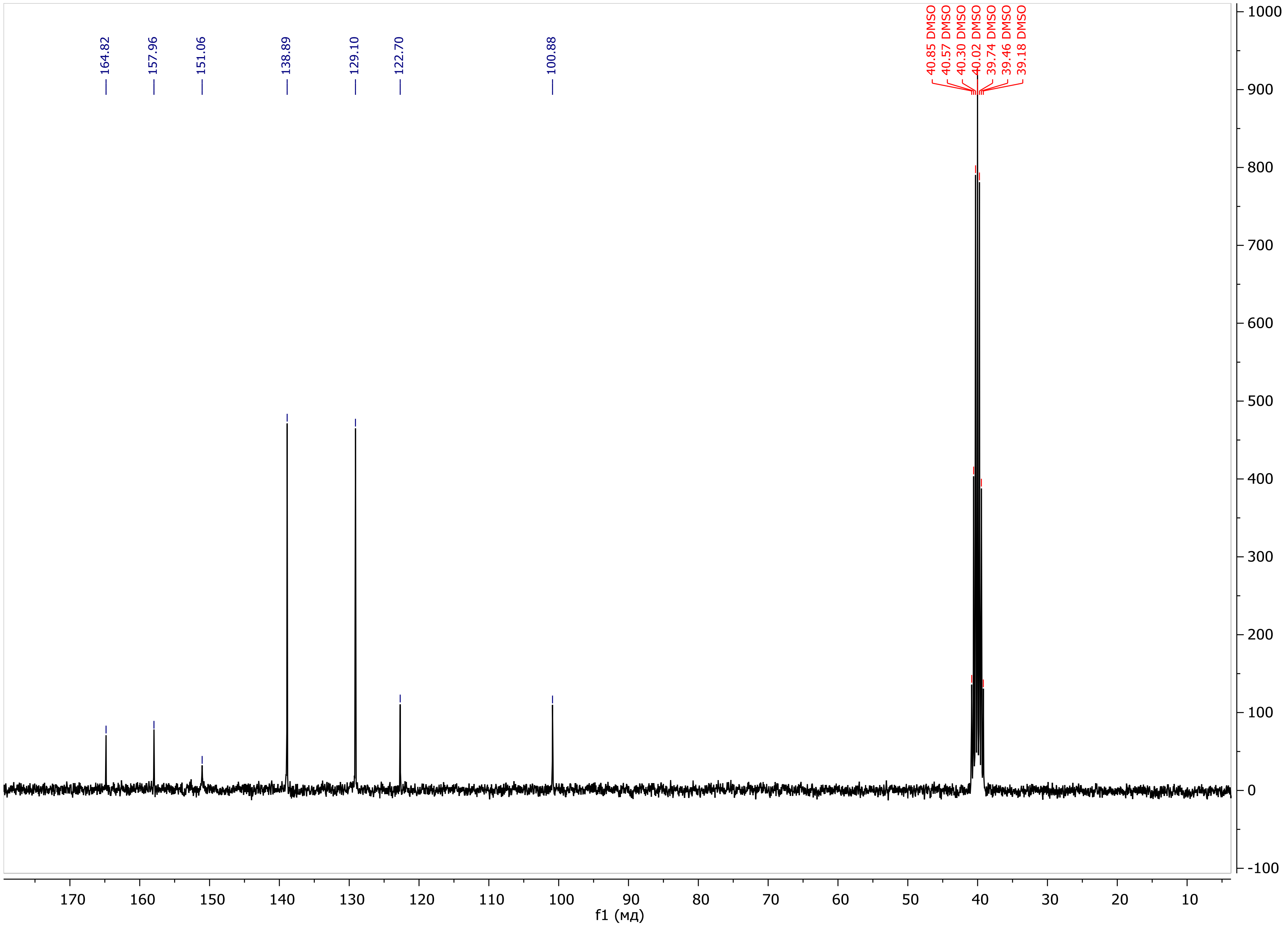

^{1}H NMR spectrum of compound **30c**

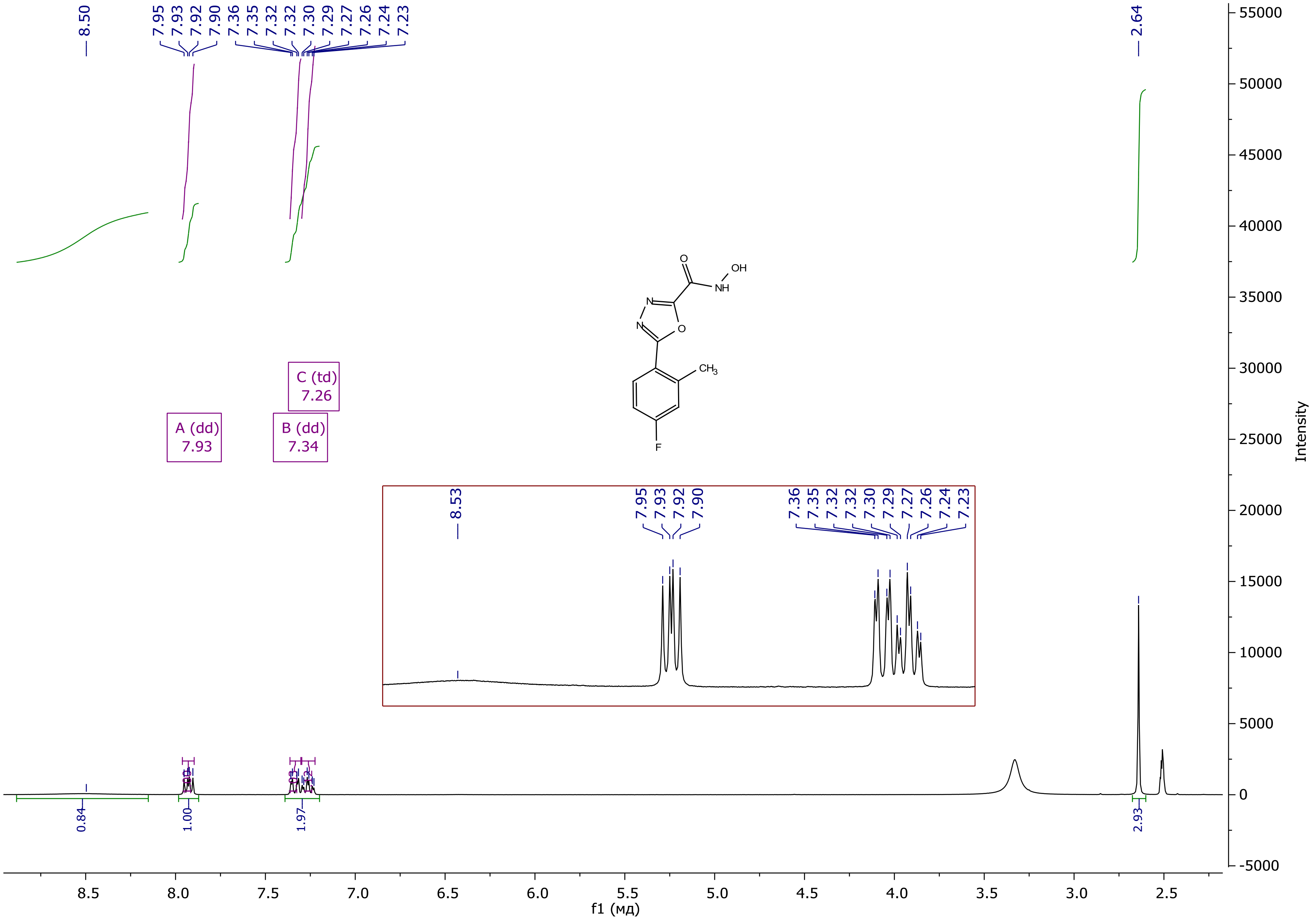

^{13}C NMR spectrum of compound **30c**

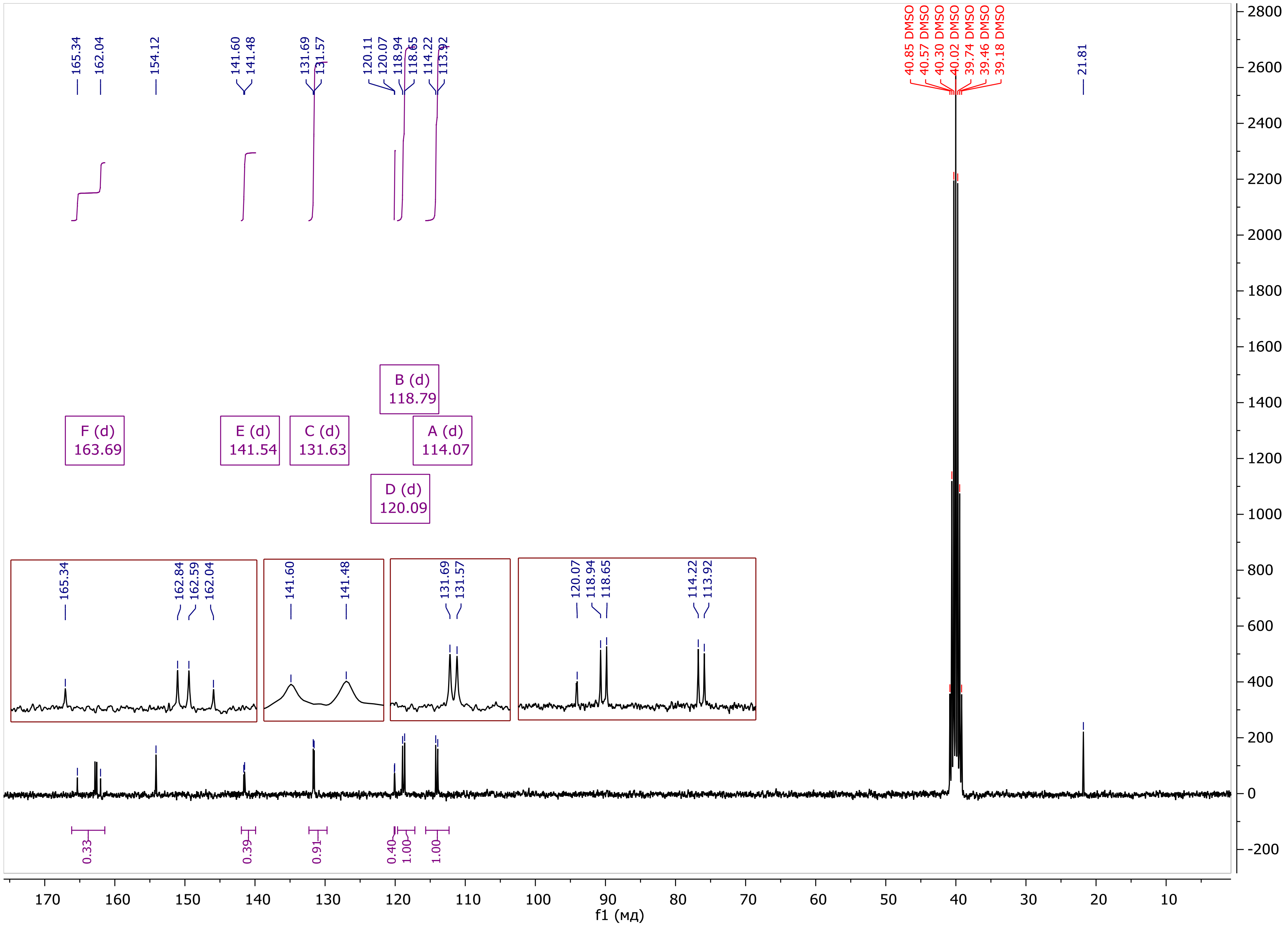

^{1}H NMR spectrum of compound **30b**

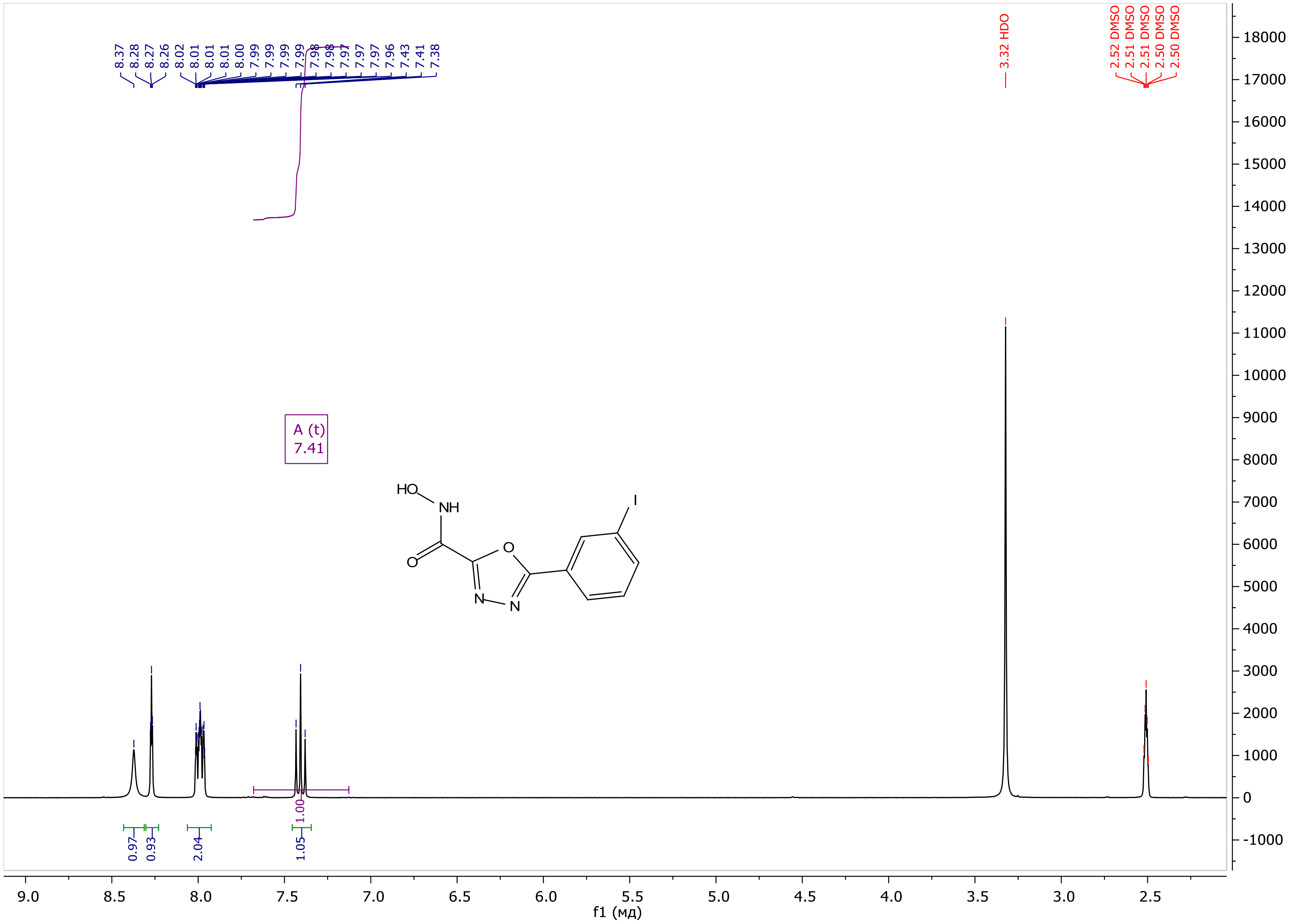

^{13}C NMR spectrum of compound **30b**

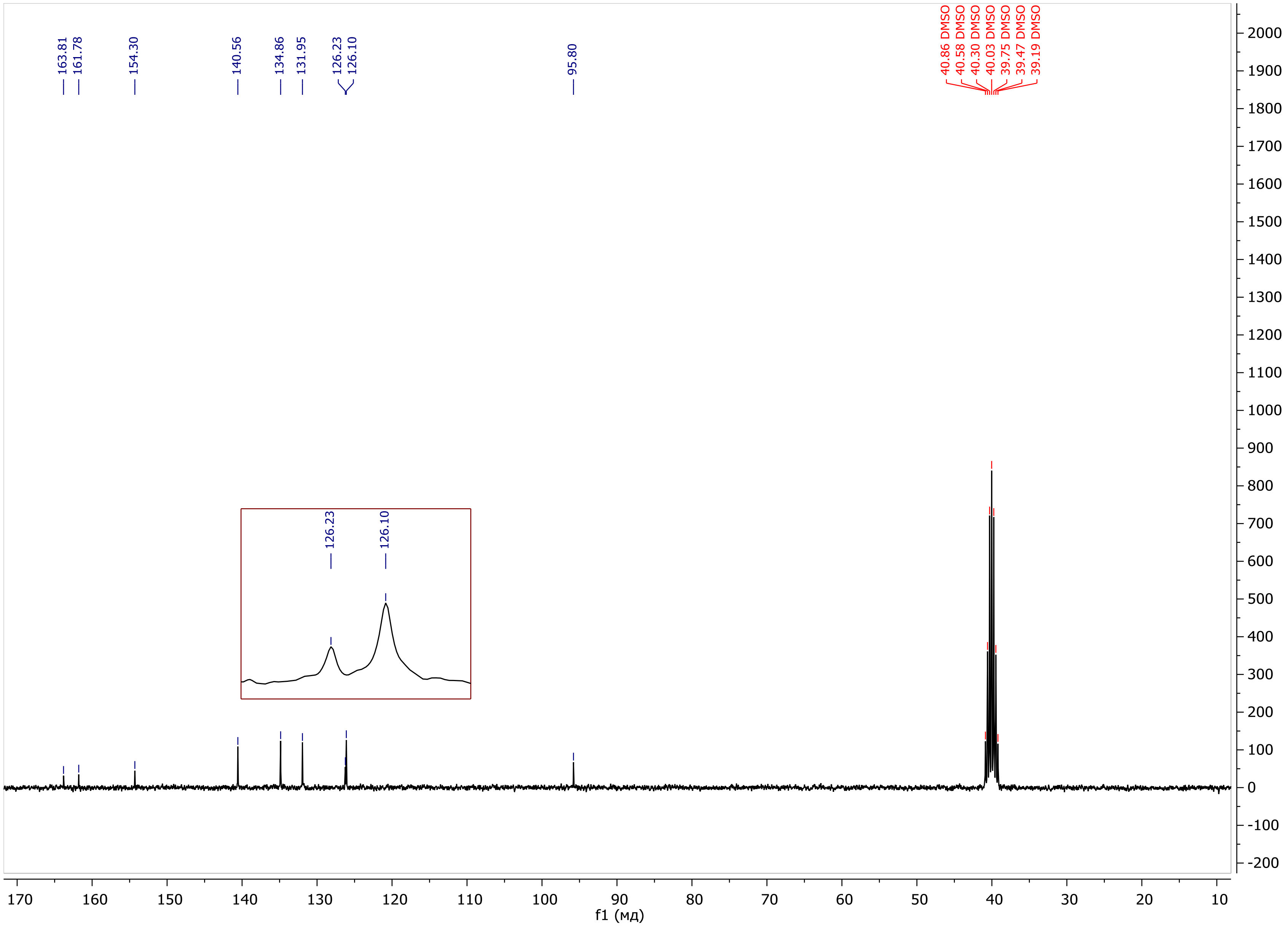

HRMS spectra for hydroxamic acids obtained

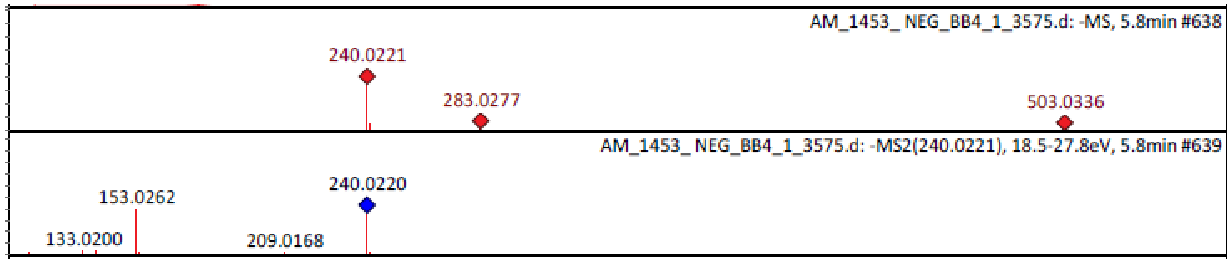

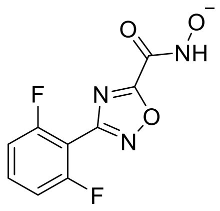

m/z: 240.0226 (100.0%), 241.0260 (9.7%), 241.0197 (1.1%)

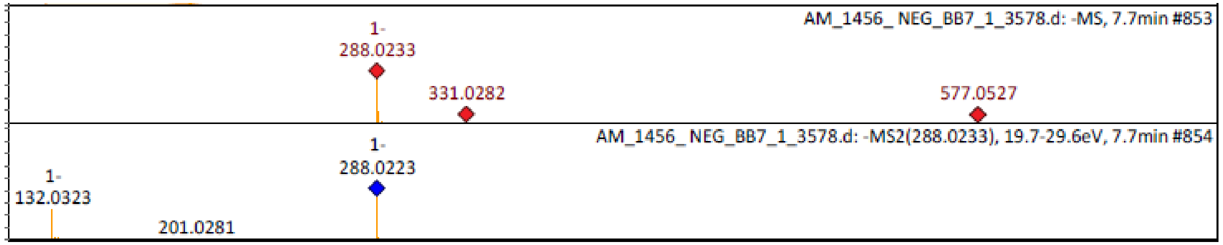

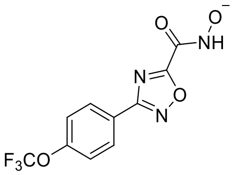

m/z: 288.0238 (100.0%), 289.0271 (10.8%), 289.0208 (1.1%)

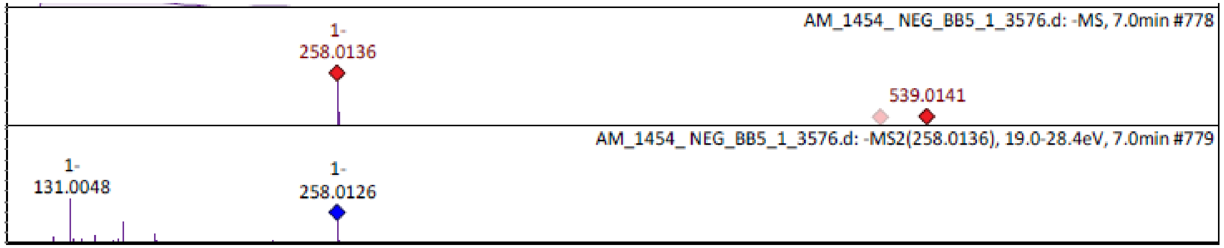

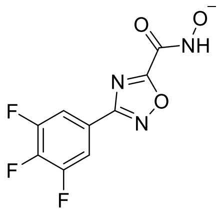

m/z: 258.0132 (100.0%), 259.0166 (9.7%), 259.0102 (1.1%)

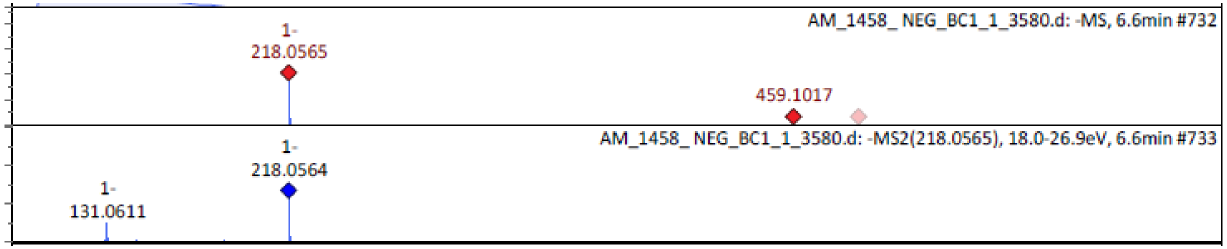

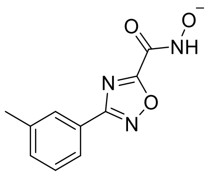

m/z: 218.0571 (100.0%), 219.0605 (10.8%), 219.0541 (1.1%)

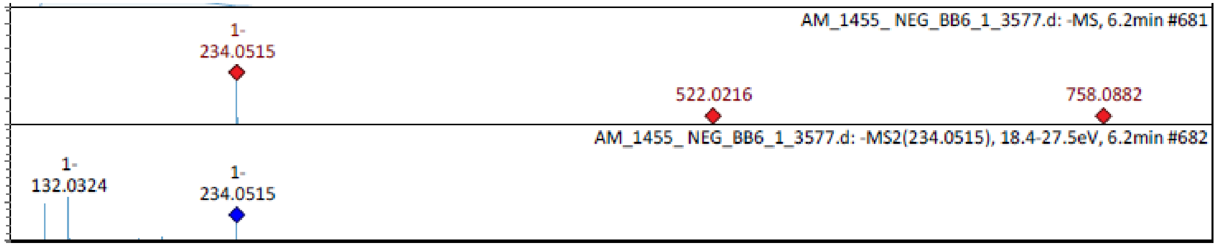

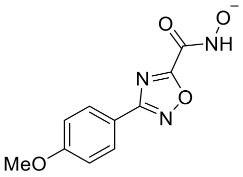

m/z: 234.0520 (100.0%), 235.0554 (10.8%), 235.0491 (1.1%)

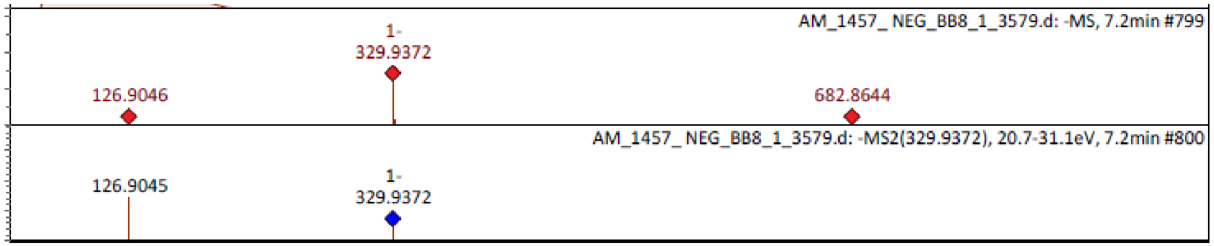

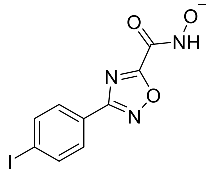

m/z: 329.9381 (100.0%), 330.9415 (9.7%), 330.9351 (1.1%)

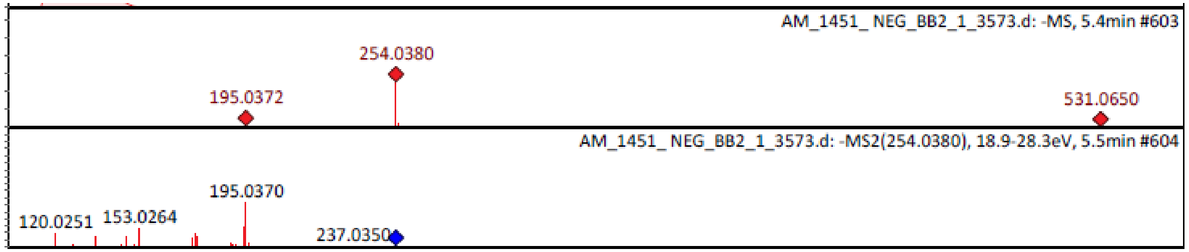

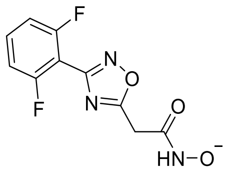

m/z: 254.0383 (100.0%), 255.0416 (10.8%), 255.0353 (1.1%)

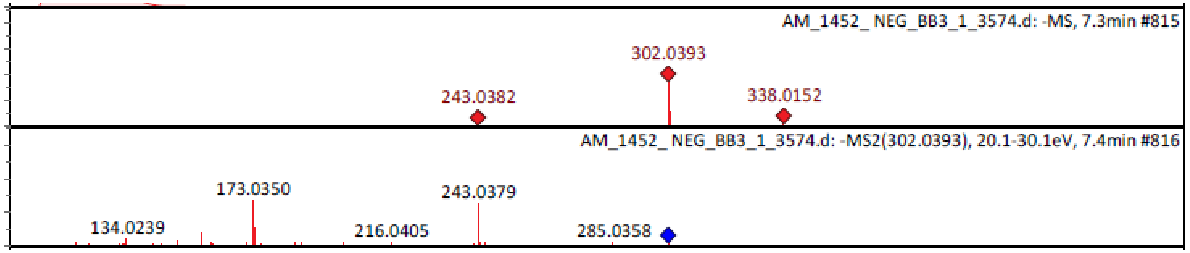

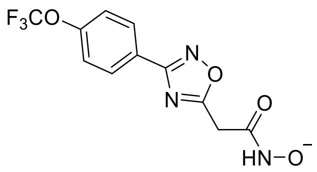

m/z: 302.0394 (100.0%), 303.0428 (11.9%), 303.0364 (1.1%)

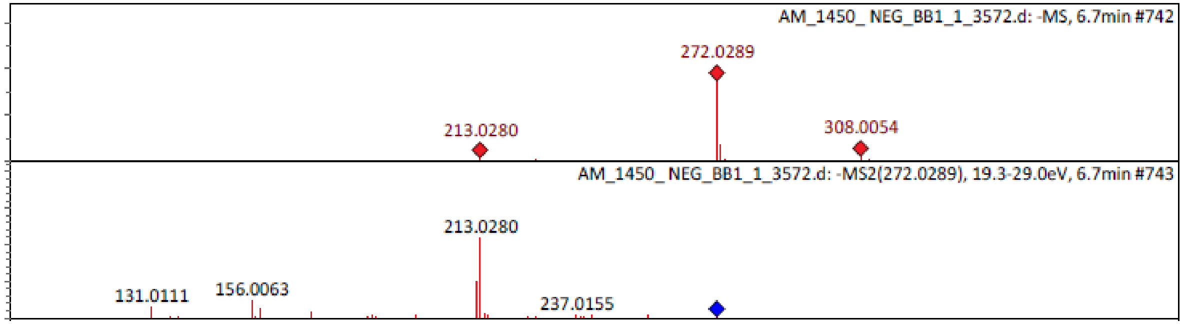

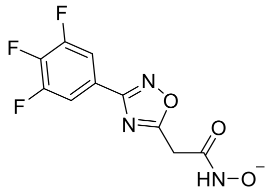

m/z: 272.0288 (100.0%), 273.0322 (10.8%), 273.0259 (1.1%)

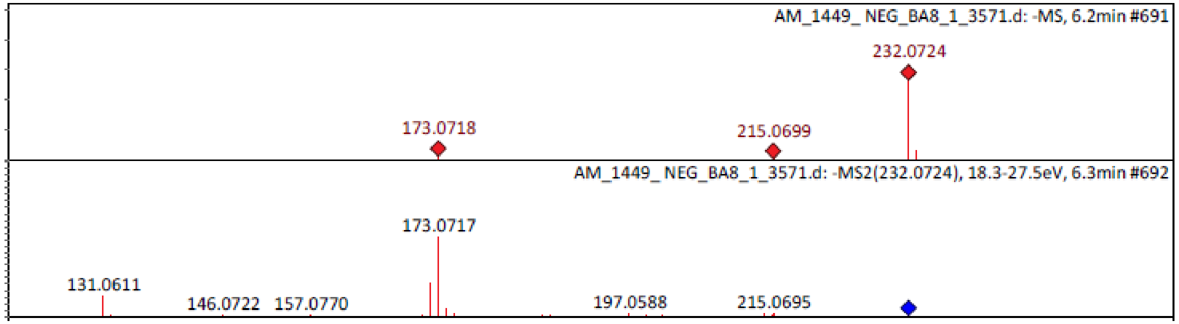

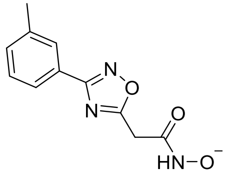

m/z: 232.0728 (100.0%), 233.0761 (11.9%), 233.0698 (1.1%)

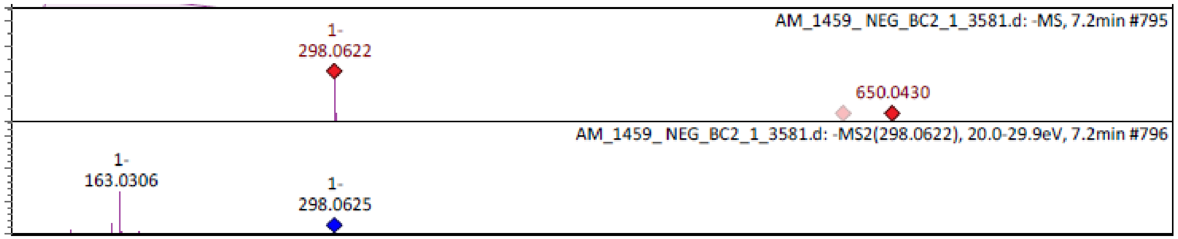

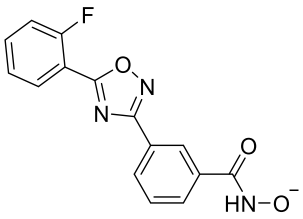

m/z: 298.0633 (100.0%), 299.0667 (16.2%), 300.0701 (1.2%), 299.0604 (1.1%)

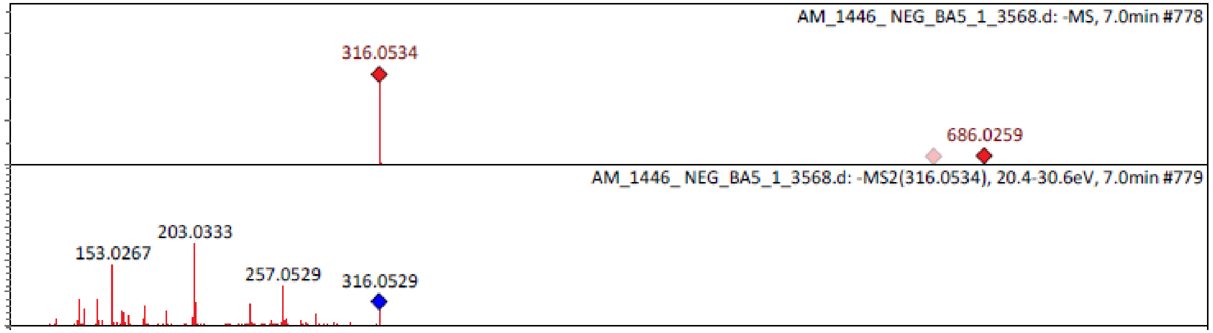

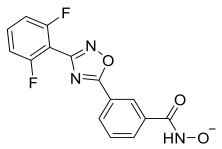

m/z: 316.0539 (100.0%), 317.0573 (16.2%), 318.0606 (1.2%), 317.0510 (1.1%)

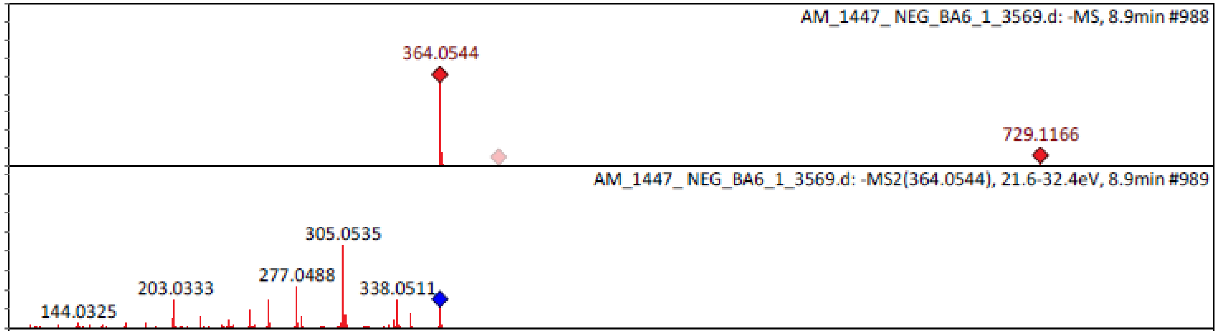

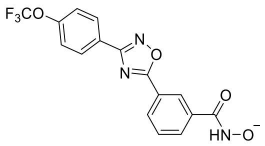

m/z: 364.0551 (100.0%), 365.0584 (17.3%), 366.0618 (1.4%), 365.0521 (1.1%)

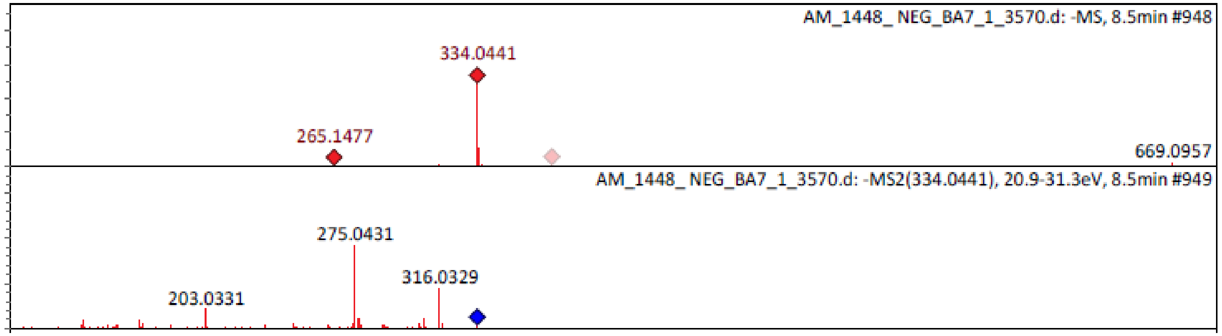

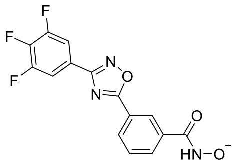

m/z: 334.0445 (100.0%), 335.0479 (16.2%), 336.0512 (1.2%), 335.0415 (1.1%)

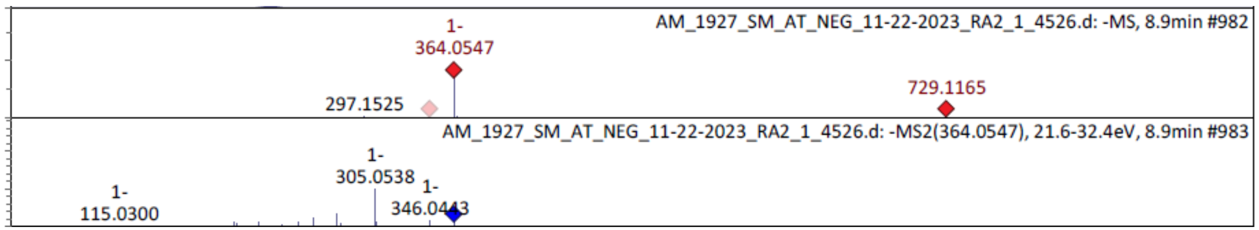

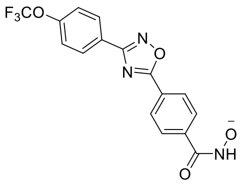

m/z: 364.0551 (100.0%), 365.0584 (17.3%), 366.0618 (1.4%), 365.0521 (1.1%)

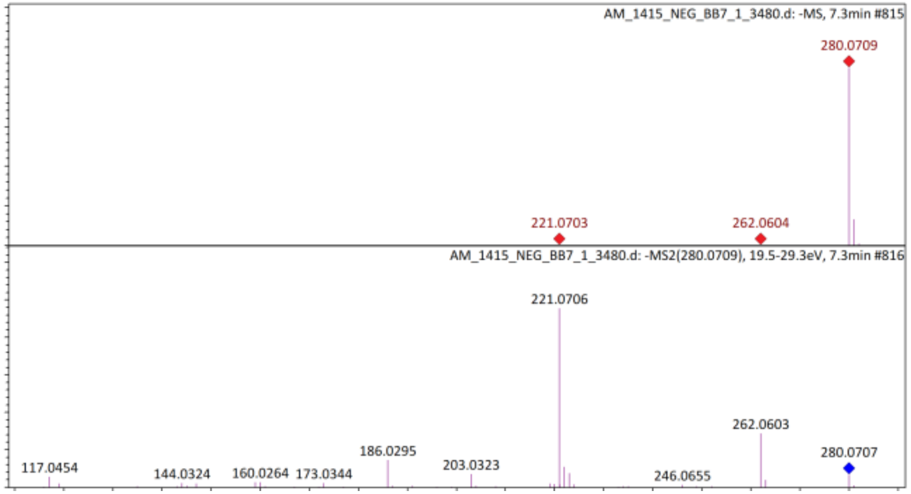

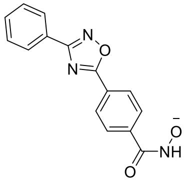

m/z: 280.0728 (100.0%), 281.0761 (16.2%), 282.0795 (1.2%), 281.0698 (1.1%)

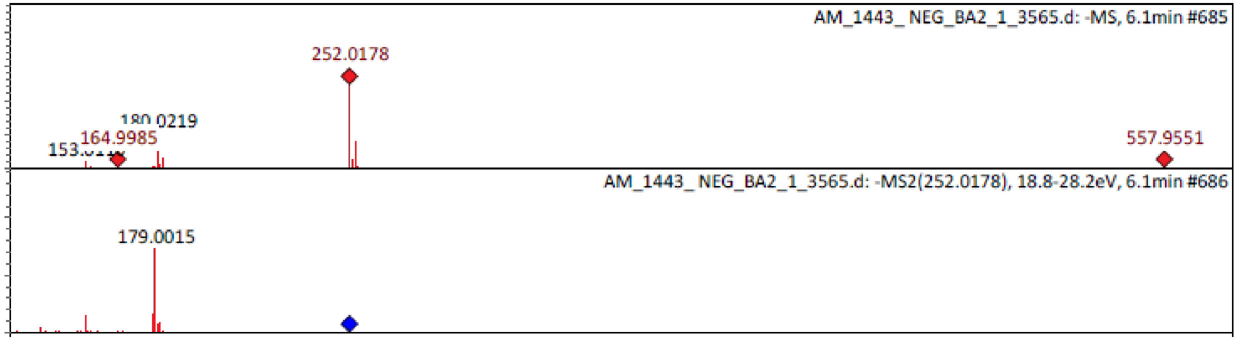

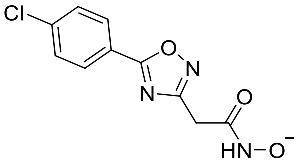

m/z: 252.0181 (100.0%), 254.0152 (32.0%), 253.0215 (10.8%), 255.0185 (3.5%), 253.0152 (1.1%)

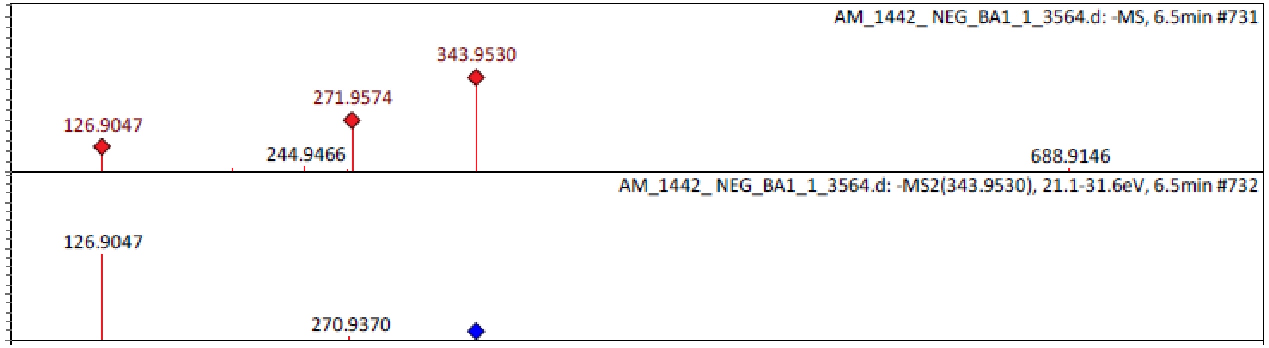

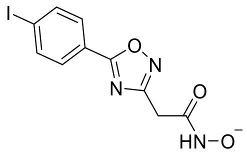

m/z: 343.9538 (100.0%), 344.9571 (10.8%), 344.9508 (1.1%)

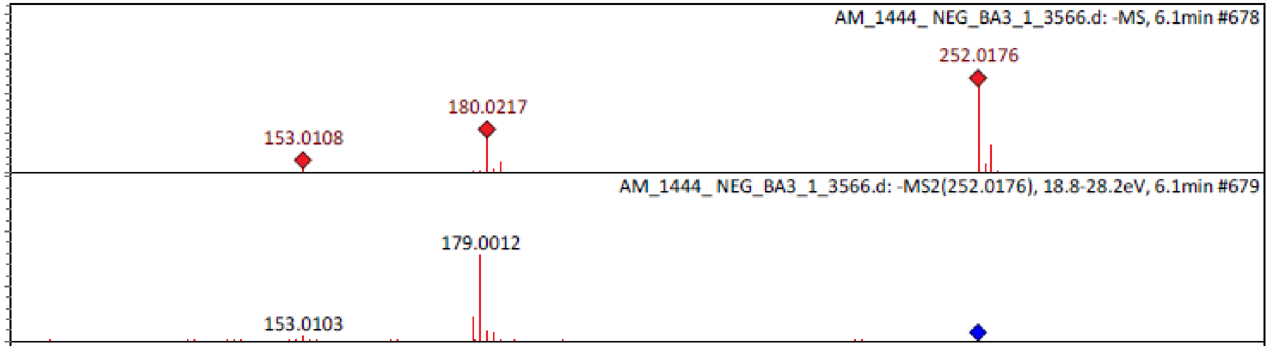

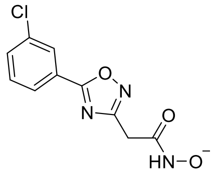

m/z: 252.0181 (100.0%), 254.0152 (32.0%), 253.0215 (10.8%), 255.0185 (3.5%), 253.0152 (1.1%)

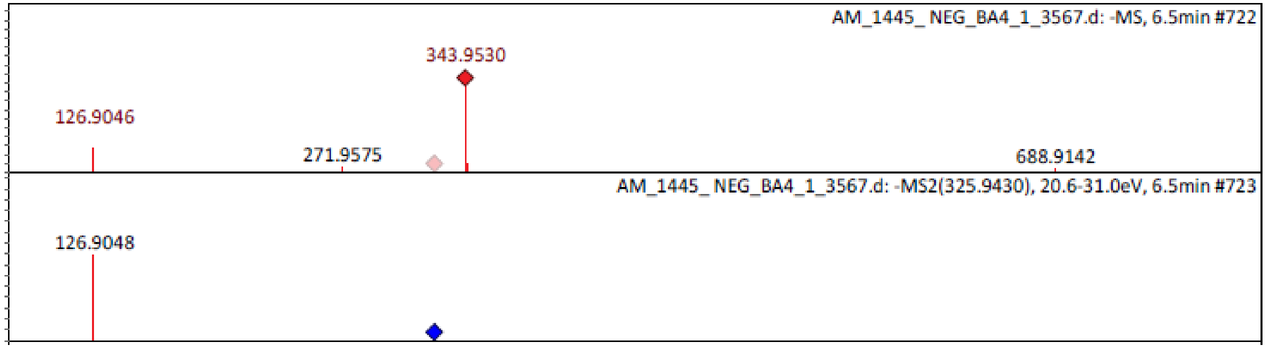

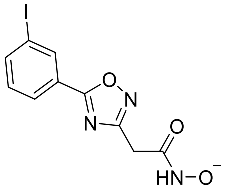

m/z: 343.9538 (100.0%), 344.9571 (10.8%), 344.9508 (1.1%)

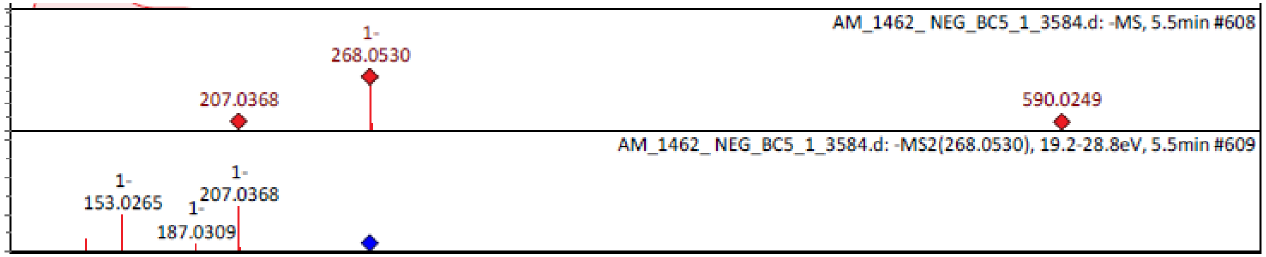

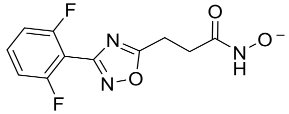

m/z: 268.0539 (100.0%), 269.0573 (11.9%), 269.0510 (1.1%)

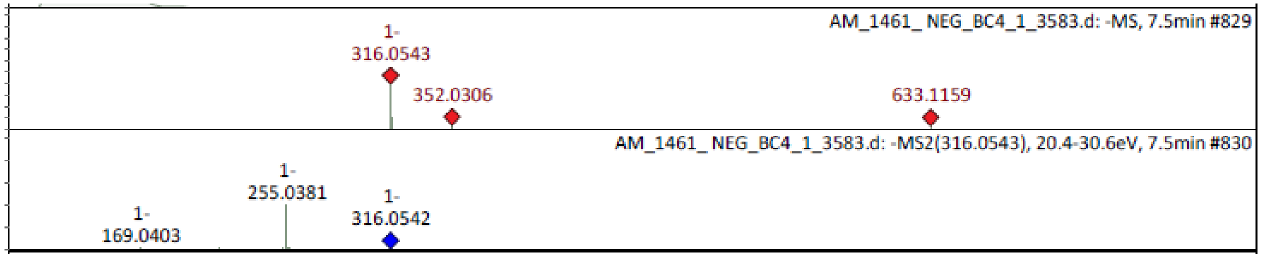

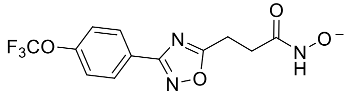

m/z: 316.0551 (100.0%), 317.0584 (13.0%), 317.0521 (1.1%)

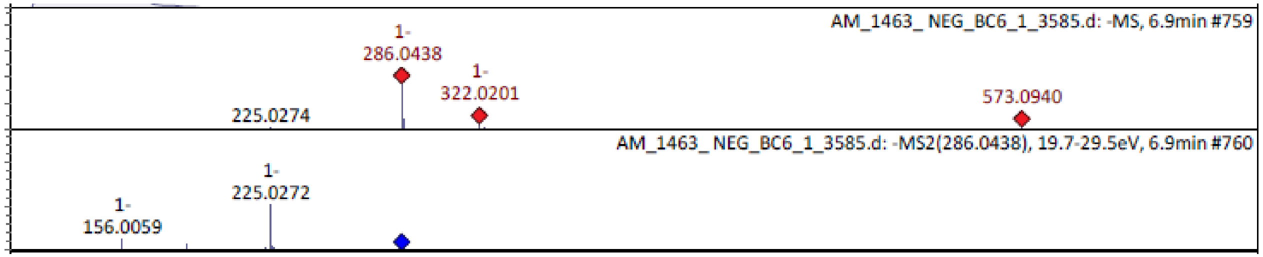

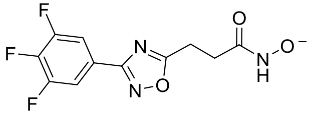

m/z: 286.0445 (100.0%), 287.0479 (11.9%), 287.0415 (1.1%)

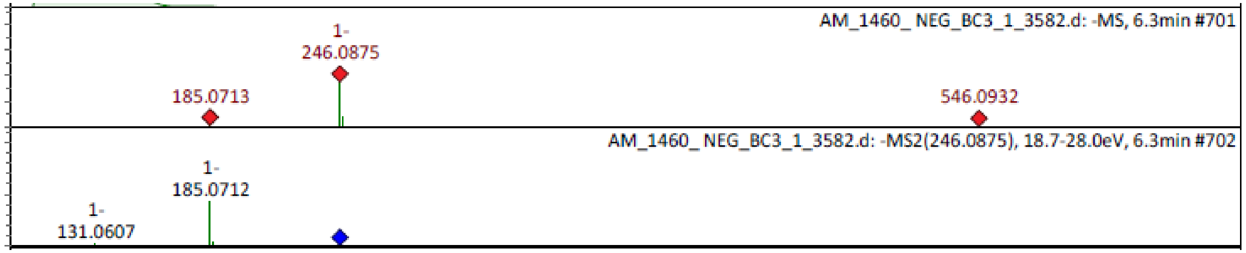

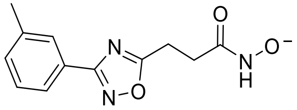

m/z: 246.0884 (100.0%), 247.0918 (13.0%), 247.0854 (1.1%)

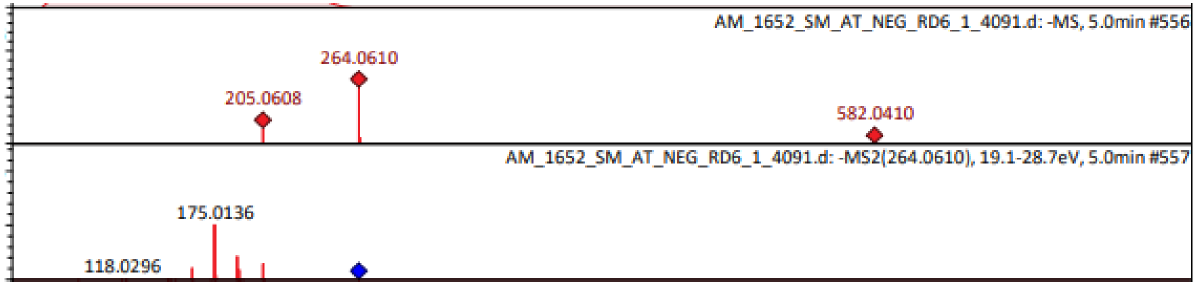

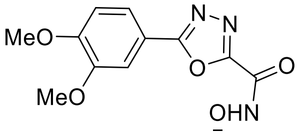

m/z: 264.0626 (100.0%), 265.0659 (11.9%), 265.0596 (1.1%), 266.0668 (1.0%)

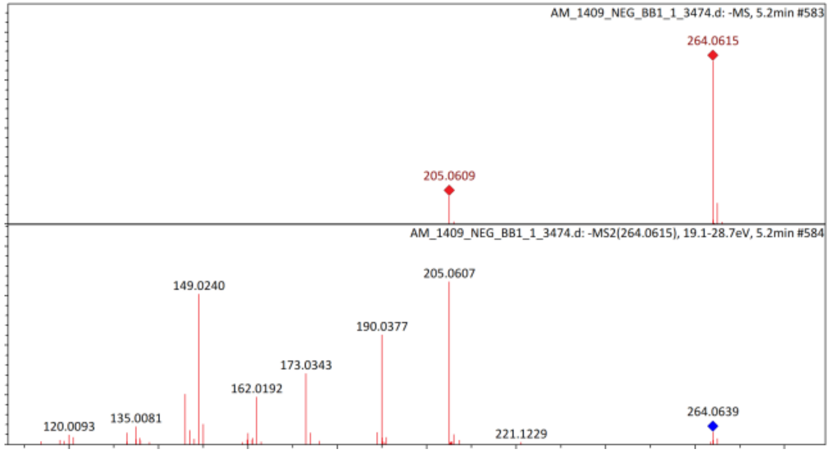

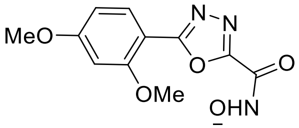

m/z: 264.0626 (100.0%), 265.0659 (11.9%), 265.0596 (1.1%), 266.0668 (1.0%)

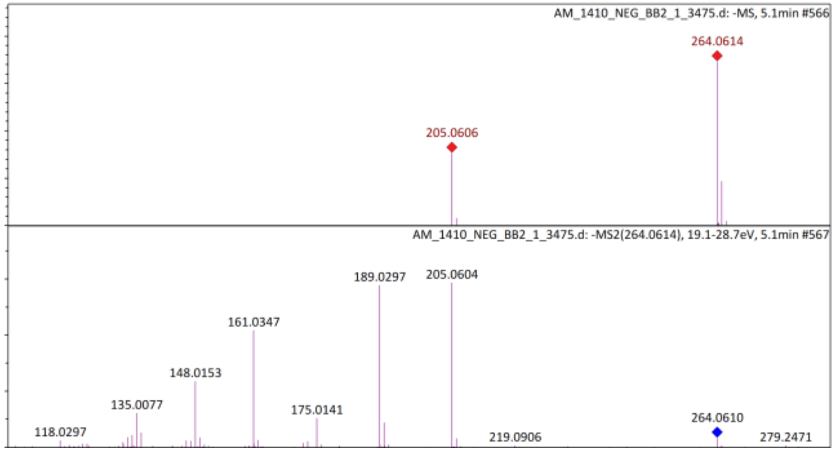

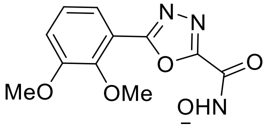

m/z: 264.0626 (100.0%), 265.0659 (11.9%), 265.0596 (1.1%), 266.0668 (1.0%)

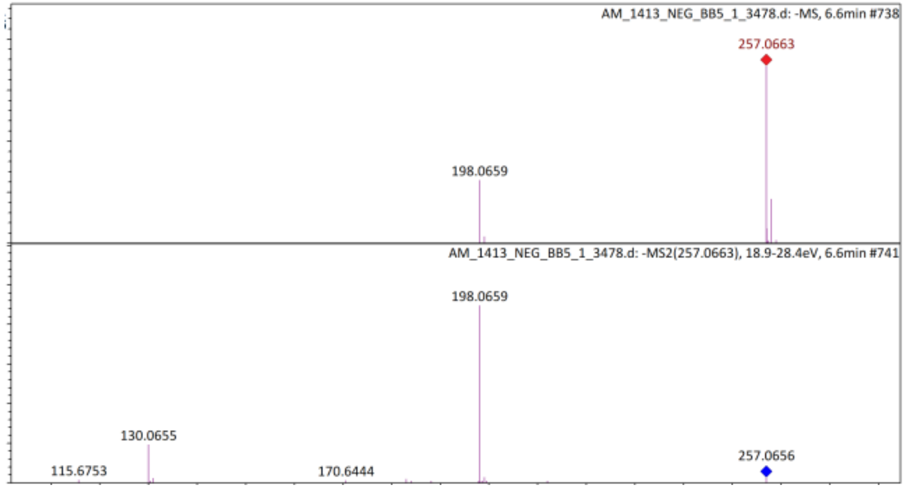

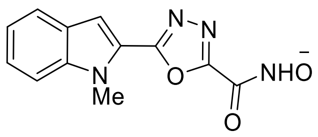

m/z: 257.0680 (100.0%), 258.0714 (13.0%), 258.0650 (1.5%)

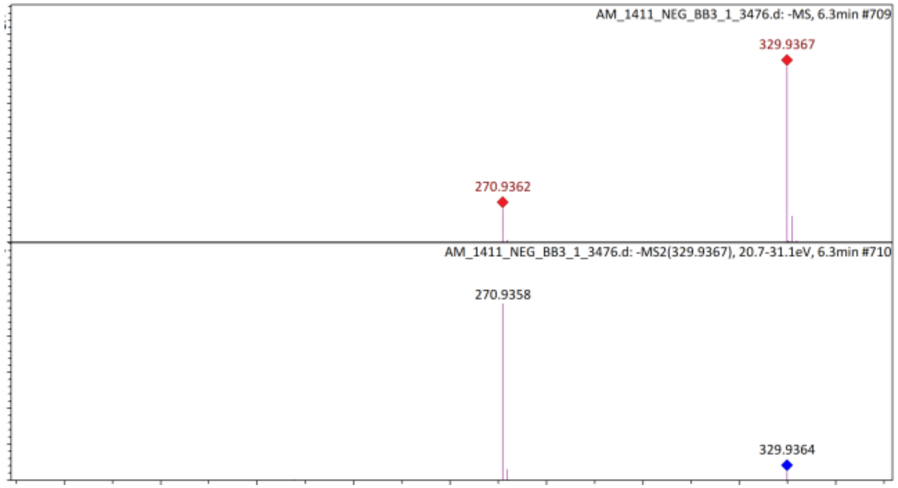

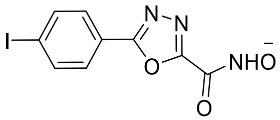

m/z: 329.9381 (100.0%), 330.9415 (9.7%), 330.9351 (1.1%)

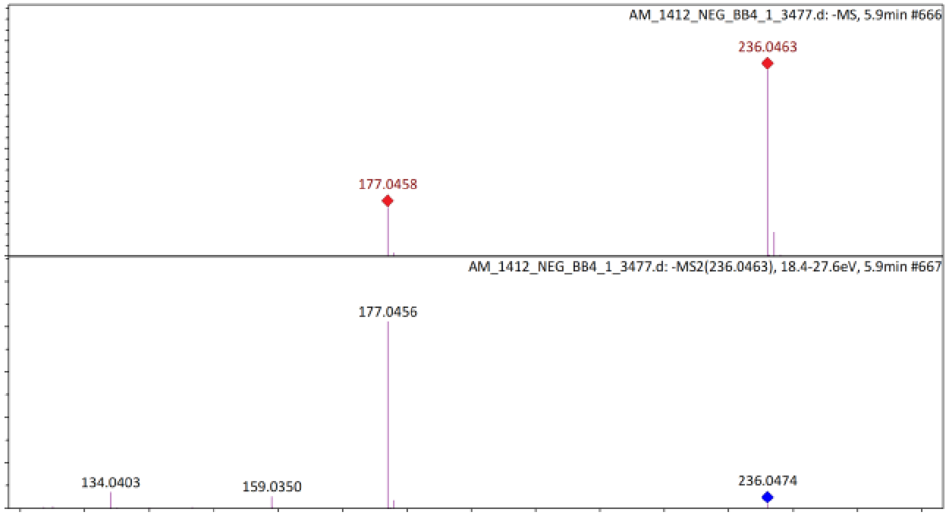

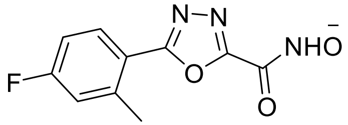

m/z: 236.0477 (100.0%), 237.0510 (10.8%), 237.0447 (1.1%)

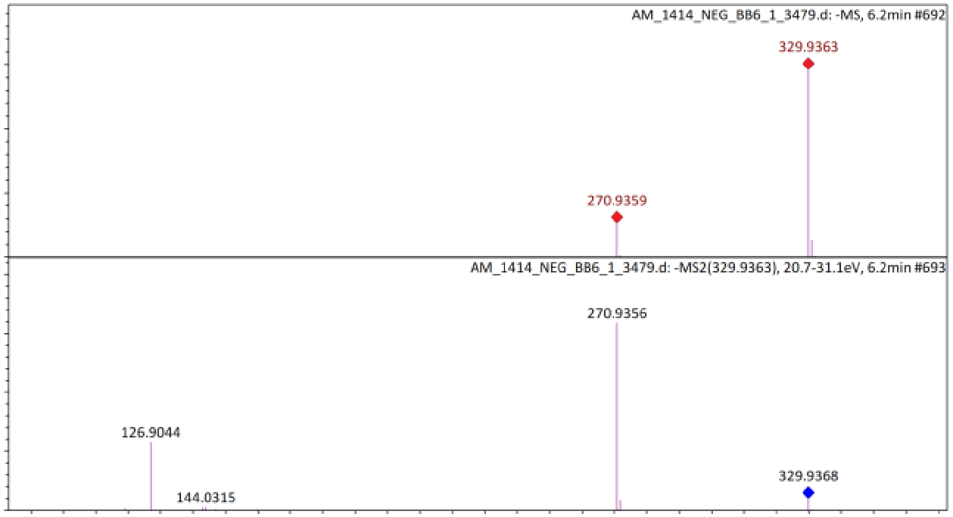

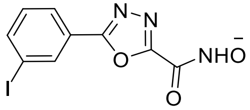

m/z: 329.9381 (100.0%), 330.9415 (9.7%), 330.9351 (1.1%)

Ligand interactions (for 6 lead compounds) with amino acid residues in the predicted binding site

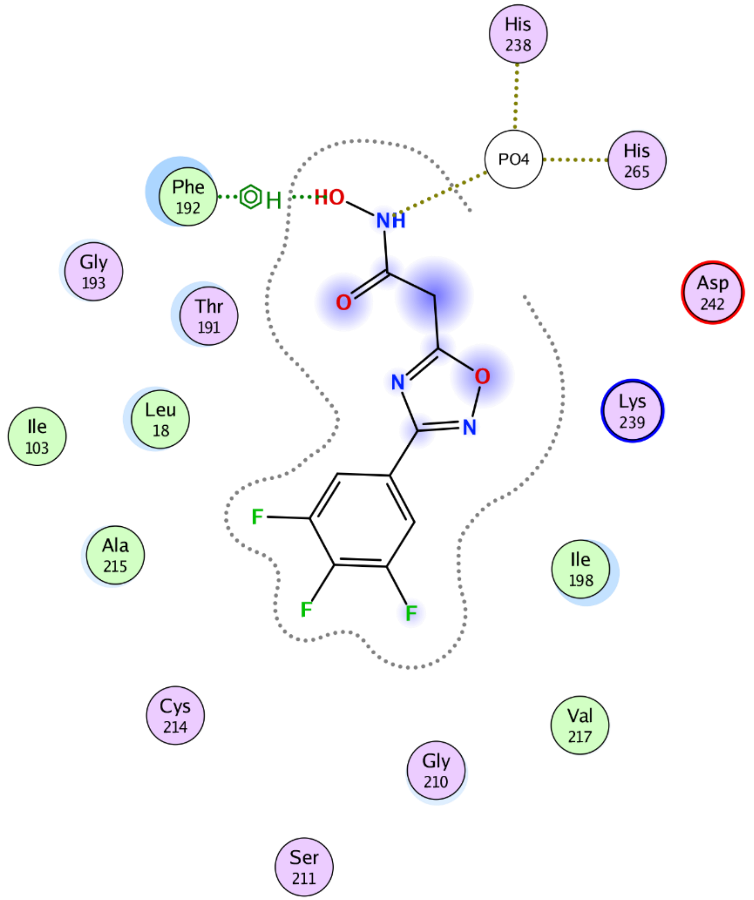

Figure S1. Interactions of ligand **9c** with amino acid residues in the predicted binding site.

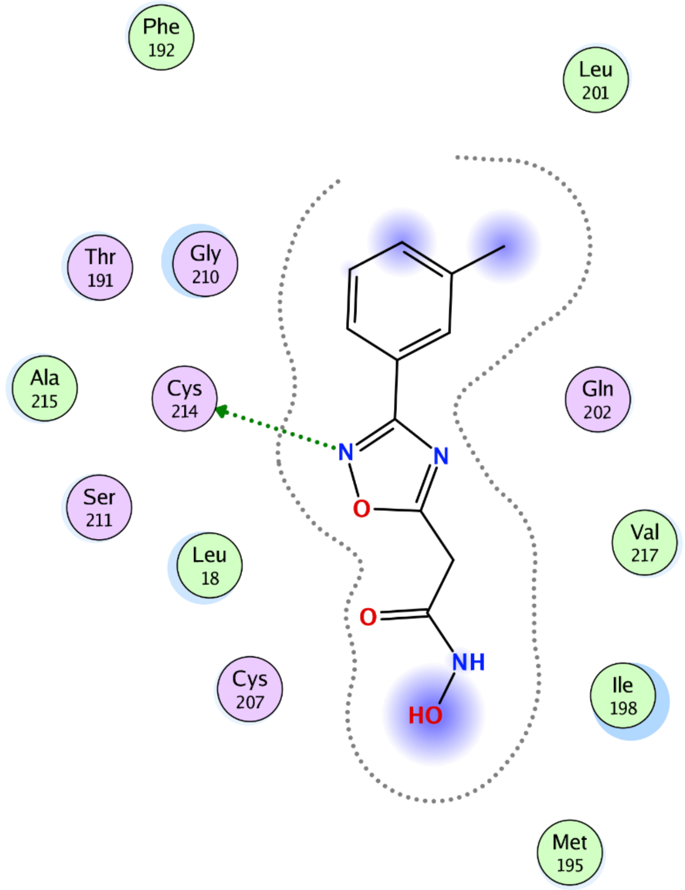

Figure S2. Interactions of ligand **9d** with amino acid residues in the predicted binding site.

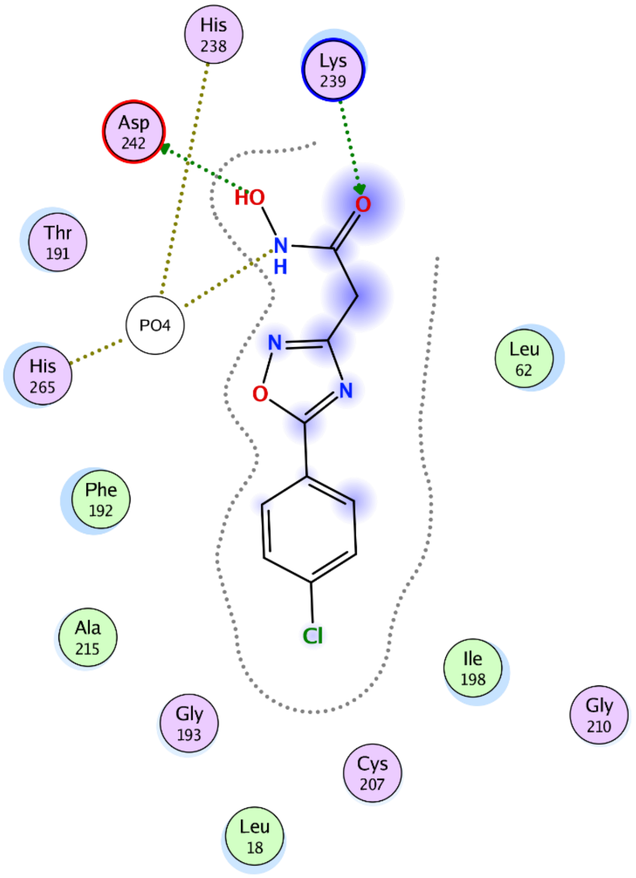

Figure S3. Interactions of ligand **23a** with amino acid residues in the predicted binding site.

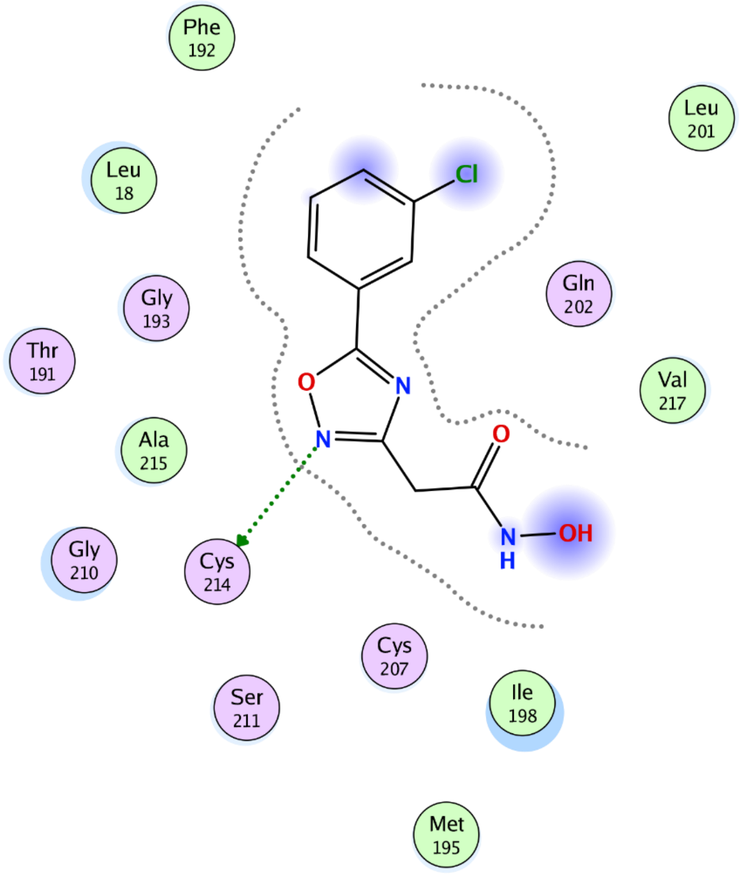

Figure S4. Interactions of ligand **23c** with amino acid residues in the predicted binding site.

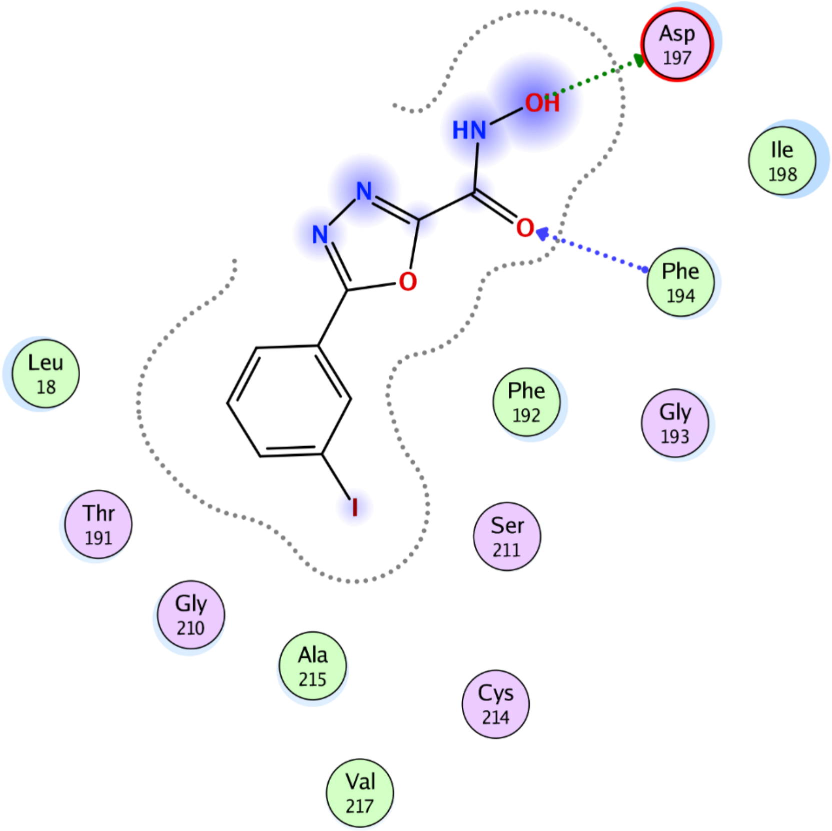

Figure S5. Interactions of ligand **30b** with amino acid residues in the predicted binding site.

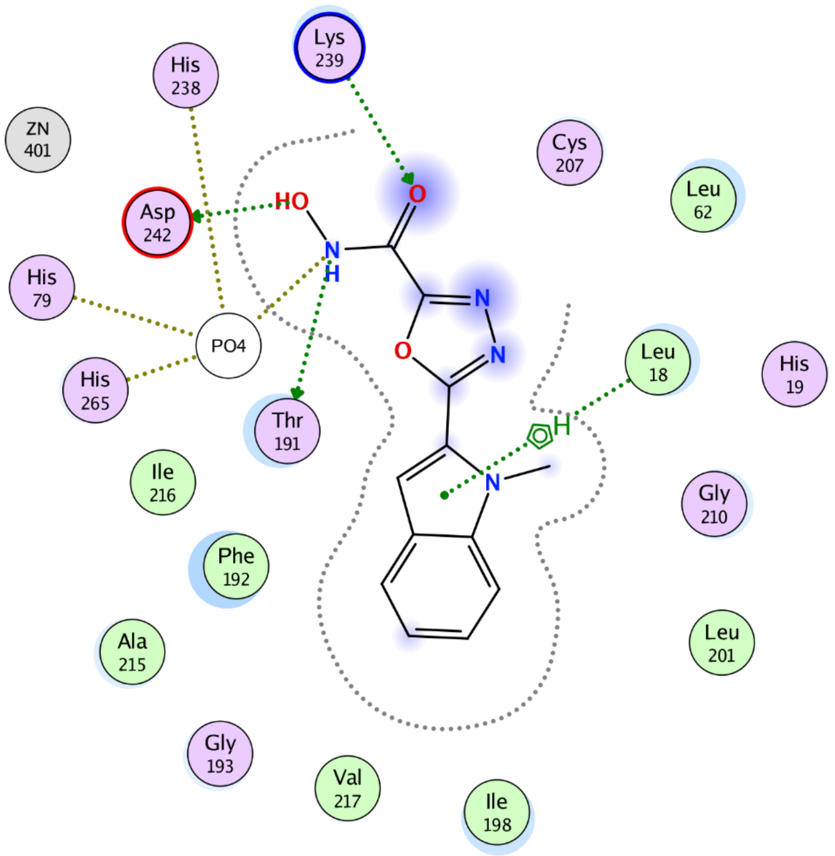

Figure S6. Interactions of ligand **36** with amino acid residues in the predicted binding site.

Table S1. Comparison 4mdt ligand binding site to residues predicted as binding sites for chemical compounds **9c**, **9d**, **23a**, **23c**, **30b**, **36**. 4mdt ligand is *UDP*-(3-*O*-(*R*-3-hydroxymyristoyl))-glucosamine. Residues Leu18, Thr191, Phe192, Ile198, Gly210 and Ala215 are common for all the sites.

**4mdt ligand**	**9c**	**9d**	**23a**	**23c**	**30b**	**36**
Leu18	Leu18	Leu18	Leu18	Leu18	Leu18	Leu18
Thr60	Ile103	Thr191	Leu62	Thr191	Thr191	His19
Leu62	Thr191	Phe192	Thr191	Phe192	Phe192	Leu62
Lys143	Phe192	Met195	Phe192	Gly193	Gly193	Thr191
Thr158	Gly193	Ile198	Gly193	Met195	Phe194	Phe192
Ile159	Ile198	Leu201	Ile198	Ile198	Asp197	Gly193
Asp160	Gly210	Gln202	Cys207	Leu201	Ile198	Ile198
Phe161	Ser211	Cys207	Gly210	Gln202	Gly210	Leu201
Thr191	Cys214	Gly210	Ala215	Cys207	Ser211	Cys207
Phe192	Ala215	Ser211	Lys239	Gly210	Cys214	Gly210
Gly193	Val217	Cys214	Asp242	Ser211	Ala215	Ala215
Phe194	His238	Ala215	His265	Cys214	Val217	Ile216
Met195	Lys239	Val217		Ala215		Val217
Asp197	Asp242			Val217		His238
Ile198						Lys239
Leu201						Asp242
Cys207						
Gly210						
Ser211						
Phe212						
Ala215						
Val217						
Lys239						
Asp242						
Lys262						
Ser263						
Gly264						
His265						

Quantitative structure–activity relationship

Table S2. List of descriptors

№	Significance	Descriptor name	Comments
1	0,22605	SMR_VSA0	Sum of v_i such that R_i is in [0,011].
2	0,224394	a_aro	Number of aromatic atoms.
3	0,223936	b_ar	Number of aromatic bonds.
4	0,223044	PEOE_VSA-6	Sum of vi where qi is less than -0.30.
5	0,222904	PEOE_RPC-	Relative negative partial charge: the smallest negative q_i divided by the sum of the negative q_i. Q_RPC - is identical to RPC - which has been retained for compatibility.
6	0,219124	SMR_VSA6	Sum of vi such that Ri is in (0.485, 0.56].
7	0,219124	chiral	The number of chiral centers.
8	0,219124	chiral_u	The number of unconstrained chiral centers.
9	0,219124	vsa_don	Approximation to the sum of VDW surface areas of pure hydrogen bond donors (not counting atoms that are both hydrogen bond donors and acceptors such as -OH) (Å2).
10	0,219124	b_double	Number of double bonds. Aromatic bonds are not considered to be double bonds.
11	0,218802	vsa_other	Approximation to the sum of VDW surface areas (Å^2) of atoms typed as "other".
12	0,218344	BCUT_SLOGP_1	The BCUT descriptors using atomic contribution to logP (using the Wildman and Crippen SlogP method) instead of partial charge.
13	0,216478	PEOE_RPC+	Relative positive partial charge: the largest positive qi divided by the sum of the positive qi. Q_RPC+ is identical to RPC + which has been retained for compatibility.
14	0,216257	KierA3	Third alpha modified shape index: $(s\text{-}1)\ (s\text{-}3)^2 / p_3^2$ for odd n, and $(s\text{-}3)\ (s\text{-}2)^2 / p_3^2$ for even n where $s = n + a$ [Hall 1991].
15	0,215308	opr_brigid	The number of rigid bonds from [Oprea 2000].
16	0,212772	BCUT_PEOE_1	The BCUT descriptors [Pearlman 1998] are calculated from the eigenvalues of a modified adjacency matrix. Each *ij* entry of the adjacency matrix takes the value 1/sqrt(b_{ij}) where b_{ij} is the formal bond order between bonded atoms *i* and *j*. The diagonal takes the value of the PEOE partial charges. The resulting eigenvalues are sorted and the smallest, 1/3-ile, 2/3-ile and largest eigenvalues are reported.
17	0,212089	h_log_pbo	Sum of log (1 + pi bond order) for all bonds.
18	0,211742	PEOE_VSA-5	Sum of v_i where q_i is in the range [-0.30,-0.25).
19	0,211292	rings	The number of rings.
20	0,211292	opr_nring	The number of rings from [Oprea 2000].
21	0,210522	GCUT_SMR_1	The GCUT descriptors using atomic contribution to molar refractivity (using the Wildman and Crippen SMR method) instead of partial charge.
22	0,210104	TPSA	Polar surface area (Å^2) calculated using group contributions to approximate the polar surface area from connection table information only. The parameterization is that of Ertl *et al.* [Ertl 2000].
23	0,209977	vsa_pol	Approximation to the sum of VDW surface areas (Å^2) of polar atoms (atoms that are both hydrogen bond donors and acceptors), such as -OH.

Table S3. Activity/predicted activity of the compounds synthesized

SMILES	№	Activity	Predicted activity
Fc1c(-c2nc(C(=O)NO)on2)c(F)ccc1	**4a**	0	2,71E-05
FC(F)(F)Oc1ccc(C2=NOC(C(=O)NO)N2)cc1	**4b**	0	2,51E-05
Fc1c(F)cc(-c2nc(C(=O)NO)on2)cc1F	**4c**	0	2,94E-07
O=C(NO)c1onc(-c2cc(C)ccc2)n1	**4d**	0	0,000454
O=C(NO)C1ON=C(c2ccc(OC)cc2)N1	**4e**	0	2,75E-06
Ic1ccc(-c2nc(C(=O)NO)on2)cc1	**4f**	0	1,31E-05
Fc1c(-c2nc(CC(=O)NO)on2)c(F)ccc1	**9a**	0	1,67E-06
FC(F)(F)Oc1ccc(C2=NOC(CC(=O)NO)N2)cc1	**9b**	0	5,39E-09
Fc1c(F)cc(-c2nc(CC(=O)NO)on2)cc1F	**9c**	**1**	0,675913
O=C(NO)Cc1onc(-c2cc(C)ccc2)n1	**9d**	**1**	0,912425
Fc1c(-c2nc(-c3cc(C(=O)NO)ccc3)on2)c(F)ccc1	**14a**	0	1,73E-08
FC(F)(F)Oc1ccc(C2=NOC(c3cc(C(=O)NO)ccc3)N2)cc1	**14b**	0	1,16E-08
Fc1c(F)cc(C2=NOC(c3cc(C(=O)NO)ccc3)N2)cc1F	**14c**	0	4,10E-10
FC(F)(F)Oc1ccc(-c2nc(-c3ccc(C(=O)NO)cc3)on2)cc1	**14d**	0	8,04E-09
O=C(NO)c1ccc(-c2onc(-c3ccccc3)n2)cc1	**14e**	0	1,63E-07
Fc1c(-c2nc(CCC(=O)NO)on2)c(F)ccc1	**17a**	0	3,61E-07
FC(F)(F)Oc1ccc(-c2nc(CCC(=O)NO)on2)cc1	**17b**	0	5,82E-09
O=C(NO)CCc1onc(-c2cc(C)ccc2)n1	**17c**	0	2,10E-05
Fc1c(F)cc(-c2nc(CCC(=O)NO)on2)cc1F	**17c**	0	1,11E-07
Clc1ccc(-c2onc(CC(=O)NO)n2)cc1	**23a**	**1**	0,999496
Ic1ccc(-c2onc(CC(=O)NO)n2)cc1	**23b**	0	2,12E-05
Clc1cc(-c2onc(CC(=O)NO)n2)ccc1	**23c**	**1**	0,99914
Ic1cc(-c2onc(CC(=O)NO)n2)ccc1	**23d**	0	1,51E-06
Fc1c(-c2onc(-c3cc(C(=O)NO)ccc3)n2)cccc1	**26**	0	2,80E-07
Ic1ccc(-c2oc(C(=O)NO)nn2)cc1	**30a**	0	0,010669
Ic1cc(-c2oc(C(=O)NO)nn2)ccc1	**30b**	**1**	0,854806
Fc1cc(C)c(-c2oc(C(=O)NO)nn2)cc1	**30c**	0	2,79E-05
O=C(NO)c1oc(-c2c(OC)cc(OC)cc2)nn1	**30d**	0	2,81E-09
O=C(NO)c1oc(-c2c(OC)c(OC)ccc2)nn1	**30e**	0	1,59E-08
O=C(NO)c1oc(-c2cc(O)c(O)cc2)nn1	**30f**	0	7,38E-05
O=C(NO)c1oc(-c2n(C)c3c(c2)cccc3)nn1	**36**	**1**	0,999296
